# An expanded molecular phylogeny of Plumbaginaceae, with emphasis on *Limonium* (sea lavenders): Taxonomic implications and biogeographic considerations

**DOI:** 10.1002/ece3.4553

**Published:** 2018-12-06

**Authors:** Konstantina Koutroumpa, Spyros Theodoridis, Ben H. Warren, Ares Jiménez, Ferhat Celep, Musa Doğan, Maria M. Romeiras, Arnoldo Santos‐Guerra, Jóse María Fernández‐Palacios, Juli Caujapé‐Castells, Mónica Moura, Miguel Menezes de Sequeira, Elena Conti

**Affiliations:** ^1^ Department of Systematic and Evolutionary Botany University of Zurich Zurich Switzerland; ^2^ Department of Biology, Faculty of Arts and Sciences Kırıkkale University Kırıkkale Turkey; ^3^ Department of Biological Sciences Middle East Technical University Cankaya, Ankara Turkey; ^4^ Linking Landscape, Environment, Agriculture and Food (LEAF) Instituto Superior de Agronomia (ISA), Universidade de Lisboa Lisboa Portugal; ^5^ Centre for Ecology, Evolution and Environmental Changes (cE3c), Faculdade de Ciências Universidade de Lisboa Lisboa Portugal; ^6^ Calle Guaidil 16 Tegueste, Tenerife Spain; ^7^ Island Ecology and Biogeography Research Group, Universitario de Enfermedades Tropicales y Salud Publica de Canarias (IUETSPC) Universidad de La Laguna Tenerife Spain; ^8^ Jardín Botánico Canario “Viera y Clavijo” – Unidad Asociada CSIC Cabildo de Gran Canaria Las Palmas de Gran Canaria Spain; ^9^ CIBIO, Research Centre in Biodiversity and Genetic Resources, InBIO Associate Laboratory, Azores Group Universidade dos Açores Ponta Delgada, Azores Portugal; ^10^ InBio, Research Network in Biodiversity and Evolutionary Biology, CIBIO‐Azores, Madeira Botanical Group (GBM) Universidade da Madeira Funchal Portugal

**Keywords:** *Limonium*, Macaronesia, Mediterranean region, Molecular systematics, Plumbaginaceae, taxonomy

## Abstract

Plumbaginaceae is characterized by a history of multiple taxonomic rearrangements and lacks a broad molecular phylogenetic framework. *Limonium* is the most species‐rich genus of the family with *ca*. 600 species and cosmopolitan distribution. Its center of diversity is the Mediterranean region, where *ca*. 70% of all *Limonium* species are endemic. In this study, we sample 201 *Limonium* species covering all described infrageneric entities and spanning its wide geographic range, along with 64 species of other Plumbaginaceae genera, representing 23 out of 29 genera of the family. Additionally, 20 species of the sister family Polygonaceae were used as outgroup. Sequences of three chloroplast (*trnL‐F*, *matK,* and *rbcL*) and one nuclear (ITS) loci were used to infer the molecular phylogeny employing maximum likelihood and Bayesian analyses. According to our results, within Plumbaginoideae, *Plumbago* forms a non‐monophyletic assemblage, with *Plumbago europaea* sister to *Plumbagella*, while the other *Plumbago* species form a clade sister to *Dyerophytum*. Within Limonioideae, *Ikonnikovia* is nested in *Goniolimon*, rejecting its former segregation as genus distinct from *Goniolimon*. *Limonium* is divided into two major clades: *Limonium *subg. *Pteroclados s.l*., including *L. *sect. *Pteroclados* and *L. anthericoides*, and *L. *subg. *Limonium*. The latter is divided into three well‐supported subclades: the monospecific *L. *sect. *Limoniodendron* sister to a clade comprising a mostly non‐Mediterranean subclade and a Mediterranean subclade. Our results set the foundation for taxonomic proposals on sections and subsections of *Limonium*, namely: (a) the newly described *L. *sect. *Tenuiramosum*, created to assign *L. anthericoides *at the sectional rank; (b) the more restricted circumscriptions of *L. *sect. *Limonium* (= *L. *sect. *Limonium* subsect. *Genuinae*) and *L. *sect. *Sarcophyllum* (for the Sudano‐Zambezian/Saharo‐Arabian clade); (c) the more expanded circumscription of *L. *sect. *Nephrophyllum* (including species of the *L. bellidifolium* complex); and (d) the new combinations for *L. *sect. *Pruinosum* and *L. *sect. *Pteroclados* subsect. *Odontolepideae *and subsect. *Nobiles*.

## 1 INTRODUCTION

Reconstructing the tree of life has represented one of the most ambitious goals of the scientific community ever since Darwin discovered the link between the diversity of organisms and their shared ancestry (Darwin, 1859; Hinchliff et al., 2015). However, some groups of organisms lack detailed phylogenies and phylogeny‐based taxonomy, and represent major gaps in our knowledge of how the diversity of life evolved and is currently partitioned; hence, they are preferred targets of modern phylogenetic analyses. Due to its remarkable taxonomic complexity and high species richness, the plant family Plumbaginaceae Juss. (leadwort family) represents one such group (e.g., Lledó, Crespo, Cameron, Fay, & Chase, 1998; Lledó, Crespo, Cox, Fay, & Chase, 2000; Lledó, Karis, Crespo, Fay, & Chase, 2001).

Plumbaginaceae form a species‐rich, highly diverse family exhibiting a world‐wide distribution, with representatives occurring predominantly in temperate regions of the Northern Hemisphere. Several species of Plumbaginaceae are used as garden ornaments and some have medicinal uses (mostly *Limonium *Mill. and *Plumbago *L. species). The family consists mainly of perennial shrubs, subshrubs, and herbs growing mostly in arid and saline habitats (Kubitzki, 1993). Embedded in the order Caryophyllales, the family is sister to Polygonaceae Juss. (e.g., Chase et al., 1993; Cuénoud et al., 2002; APG IV, 2016). Kubitzki (1993) recognized 27 genera in the family, whereas a later study on Caryophyllales identified 29 genera for the family (Hernández‐Ledesma et al., 2015). The total number of species ascribed to these genera ranges from one (e.g., *Bamiania *Lincz., *Bukiniczia *Lincz., *Ghaznianthus *Lincz.*, Saharanthus *M.B. Crespo & M.D. Lledó) to 596 (*Limonium*; Hassler, 2018), making Plumbaginaceae one of the top 20% of angiosperm families in terms of species richness (Christenhusz & Byng, 2016).

In Plumbaginaceae, some generic boundaries have been controversial and re‐arranged multiple times. For example, in *Limonium *individual species or entire sections were segregated to form new genera (such as *Afrolimon *Lincz., *Eremolimon *Lincz., and *Linczevskia *Tzelev) that were later assigned again back to *Limonium *on the basis of molecular phylogenetic analyses (Lledó, Crespo, Fay, & Chase, 2005; Malekmohammadi, Akhani, & Borsch, 2017). On the systematics of Plumbaginaceae, both Kubitzki (1993) and Hernández‐Ledesma et al. (2015) agree on the necessity of additional studies aimed at clarifying generic boundaries and relationships. The main generic diversity of the family is centered in the mountains of Central Asia (Kubitzki, 1993) in the Irano‐Turanian phytogeographic region, where many genera occur and some are endemic (e.g., *Acantholimon *Boiss., *Bamiania, Bukiniczia, Cephalorhizum *Popov & Korovin, *Chaetolimon *(Bunge) Lincz., *Dictyolimon *Rech.f.*, Ghaznianthus, Gladiolimon *Mobayen, *Ikonnikovia *Lincz., *Neogontscharovia *Lincz.*, Plumbagella *Spach, *Popoviolimon *Lincz.*, Vassilczenkoa *Lincz.). However, the circumscription and relationships of these Central Asian genera are still debated (Moharrek, Kazempour‐Osaloo, Assadi, & Feliner, 2017), further highlighting the need for detailed phylogenetic and taxonomic studies in Plumbaginaceae.

The most widely accepted classification of Plumbaginaceae divides the family into two subfamilies, Limonioideae Reveal (former Staticoideae Burnett) and Plumbaginoideae Burnett (Hernández‐Ledesma et al., 2015; Lledó et al., 1998, 2001; Table 1). The two subfamilies are well differentiated in terms of morphological (e.g., styles connation, frequency of heterostyly and pollen dimorphism), molecular, and chemical (e.g., presence of plumbagin, A‐ring methylation in flower anthocyanins, and frequency of leucoanthocyanidins) characteristics (Lledó et al., 1998, 2001). Plumbaginoideae, with four genera, exhibits a mostly pantropical distribution, with some exceptions, for example the monospecific *Plumbagella,* which occurs in temperate Central and East Asia. The most species‐rich genus of this subfamily is *Plumbago* (“leadworts”), with approximately 20 species. *Plumbago* is the only genus of Plumbaginoideae that extends its distribution out of the Old World into America. Subfamily Limonioideae is split into two tribes, the Limonieae Reveal, comprising 24 genera (sensu Hernández‐Ledesma et al., 2015), and monogeneric Aegialitideae Z.X.Peng, with *Aegialitis *R.Br., the only tropical genus of Limonioideae, which consists of two mangrove species in south‐eastern Asia and Oceania. Genera of Limonioideae are broadly distributed and diversified in the Mediterranean and Irano‐Turanian regions, but a few genera also occur in the Southern Hemisphere. Specifically, *Muellerolimon *Lincz. is a monospecific halophytic genus from Western Australia, and *Bakerolimon* Lincz. comprises two shrubby species in the deserts of Chile and Peru. Furthermore, a minority of the species in two large genera occurs in the Southern Hemisphere (*Armeria *Willd.: South America; *Limonium*: South America, South Africa and Oceania). The most species‐rich genera of Plumbaginaceae are *Limonium*, *Acantholimon, *and *Armeria*, all in subfamily Limonioideae, comprising approximately 85%–90% of all species in the family.

**Table 1:** Plumbaginaceae genera listed according to a recent taxonomic revision by Hernández‐Ledesma et al. (2015) together with a list of species used in this study. Representatives from genera in bold letters are included in the phylogeny. *Afrolimon* (in gray) was found nested in *Limonium* by Lledó, Crespo, et al. (2005) phylogeny and is currently considered a synonym of *Limonium *(Malekmohammadi et al., 2017)

Plumbaginaceae	Sampled species
**Subfamily Plumbaginoideae**	
***Ceratostigma*** **Bunge**	*Ceratostigma minus* Stapf ex Prain
	*Ceratostigma plumbaginoides* Bunge
***Dyerophytum*** **Kuntze**	*Dyerophytum africanum* (Lam.) Kuntze
	*Dyerophytum indicum* (Gibs. ex Wight) Kuntze
***Plumbagella*** **Spach**	*Plumbagella micrantha* (Ledeb.) Spach
***Plumbago*** **L.**	*Plumbago auriculata* Lam.
	*Plumbago caerulea* Kunth
	*Plumbago europaea* L.
	*Plumbago indica* L.
	*Plumbago zeylanica* L.
**Subfamily Limonioideae**	
**Tribe Aegialitideae**	
** ** ***Aegialitis*** **R.Br.**	*Aegialitis annulata* R.Br.
**Tribe Limonieae**	
** ** ***Acantholimon*** **Boiss.**	*Acantholimon acerosum* (Willd.) Boiss.
	*Acantholimon bracteatum* (Girard) Boiss.
	*Acantholimon chitralicum* Rech.f. & Schiman‐Czeika
	*Acantholimon cymosum* Bunge
	*Acantholimon demavendicum* Bornm.
	*Acantholimon diapensioides* Boiss.
	*Acantholimon echinus* (L.) Bunge
	*Acantholimon glutinosum* Rech.f. & Köie
	*Acantholimon gorganense* Mobayen
	*Acantholimon hohenackeri* (Jaub. & Spach) Boiss.
	*Acantholimon leucochlorum* Rech.f. & Schiman‐Czeika
	*Acantholimon lycopodioides* (Girard) Boiss.
	*Acantholimon pterostegium* Bunge
	*Acantholimon restiaceum* Bunge
	*Acantholimon revolutum* Rech.f. & Köie
	*Acantholimon senganense* Bunge
	*Acantholimon solidum* Rech.f. & Köie
	*Acantholimon subulatum* Boiss.
	*Acantholimon tragacanthinum* (Jaub. & Spach) Boiss.
	*Acantholimon tricolor* Rech.f. & Köie
	*Acantholimon ulicinum* (Schult.) Boiss.
	*Acantholimon venustum* Boiss.
***Afrolimon*** **Lincz.**	See Table 2‐*Limonium* sect. *Circinaria*
***Armeria*** **Willd.**	*Armeria alliacea* (Cav.) Hoffmanns. & Link
	*Armeria arenaria* (Pers.) Schult.
	*Armeria canescens* (Host) Boiss.
	*Armeria castellana* Boiss. & Reut. ex Leresche
	*Armeria maritima* (Mill.) Willd.
	*Armeria morisii* Boiss.
	*Armeria pseudarmeria* (Murray) Mansf.
	*Armeria pungens* (Link) Hoffmanns. & Link
	*Armeria splendens* (Lag. & Rodr.) Webb
***Bakerolimon*** **Lincz.**	*Bakerolimon plumosum* (F.Phil.) Lincz.
*Bamiania* Lincz.	
***Bukiniczia*** **Lincz.**	*Bukiniczia cabulica* (Boiss.) Lincz.
***Cephalorhizum*** **Popov & Korovin**	*Cephalorhizum coelicolor* (Rech.f.) Rech.f.
***Ceratolimon*** **M.B.Crespo & M.D.Lledó**	*Ceratolimon feei* (Girard) M.B.Crespo & M.D.Lledó
	*Ceratolimon migiurtinum* (Chiov.) M.B.Crespo & M.D.Lledó
	*Ceratolimon weygandiorum* (Maire & Wilczek) M.B.Crespo & M.D.Lledó
*Chaetolimon* (Bunge) Lincz.	
***Dictyolimon*** **Rech.f.**	*Dictyolimon macrorrhabdos* (Boiss.) Rech.f.
*Ghaznianthus* Lincz.	
*Gladiolimon* Mobayen	
***Goniolimon*** **Boiss.**	*Goniolimon besserianum* (Schult.) Kusn.
	*Goniolimon incanum* (L.) Hepper
	*Goniolimon italicum* Tammaro, Pignatti & G.Frizzi
	*Goniolimon speciosum* (L.) Boiss.
	*Goniolimon tataricum* (L.) Boiss.
***Ikonnikovia*** **Lincz.**	*Ikonnikovia kaufmanniana* (Regel) Lincz.
***Limoniastrum*** **Fabr.**	*Limoniastrum guyonianum* Durieu ex Boiss.
	*Limoniastrum monopetalum* (L.) Boiss.
*Limoniopsis* Lincz.	
***Limonium*** **Mill.**	See Table 2
***Muellerolimon*** **Lincz.**	*Muellerolimon salicorniaceum* (F. Muell.) Lincz.
***Myriolimon*** **Lledó, Erben & M.B.Crespo**	*Myriolimon ferulaceum* (L.) Lledó, Erben & M.B.Crespo
*Neogontscharovia* Lincz.	
***Popoviolimon*** **Lincz.**	*Popoviolimon turcomanicum* (Popov ex Lincz.) Lincz.
***Psylliostachys*** **(Jaub. & Spach) Nevski**	*Psylliostachys suvorovii* (Regel) Roshk.
	*Psylliostachys spicata* (Willd.) Nevski
***Saharanthus*** **M.B.Crespo & M.D.Lledó**	*Saharanthus ifniensis* (Caball.) M.B.Crespo & M.D.Lledó
***Vassilczenkoa*** **Lincz.**	*Vassilczenkoa sogdiana* (Lincz.) Lincz.

*Limonium *is the only genus of Plumbaginaceae with a cosmopolitan distribution, and by far the most species‐rich genus in the family with *ca.* 600 species (Catalogue of Life reports 603 species including *Afrolimon, *synonymized under *Limonium *by Malekmohammadi et al., 2017; Hassler, 2018; list compiled by reviewing online databases, floras and published studies includes 595–599 species, K. Koutroumpa pers. obs.). Its main diversity occurs in the Mediterranean region, where *ca*. 70% of the total number of species of *Limonium* are endemic. *Limonium* species are mostly perennial herbs and shrubs growing in coastal areas, from sandy beaches to maritime cliffs and salt marshes, as well as lagoons, meadows, steppes, and deserts of the continental interior. Erben (1978) characterized its species as facultative halophytes, explaining that they grow predominately on saline and metal‐rich soils because of biotic competition. The variation of reproductive systems, both sexual and asexual (apomixis), and the frequent occurrence of hybridization and polyploidy have been proposed as major explanations for the high number of species in the genus (e.g., Palacios, Rosselló, & González‐Candelas, 2000). In the Mediterranean, in particular, high levels of apomixis in addition to habitat fragmentation (most frequently in coastal areas) favor the origin of numerous “microspecies” with restricted distributions (i.e., local endemics; Cowan, Ingrouille, & Lledó, 1998). The reproductive diversity and broad distribution of *Limonium* make it one of the most interesting groups of plants, but also beget a considerable taxonomic complexity.

The taxonomic classification of the genus is rather incomplete, and in some cases obsolete (Table 2). The most comprehensive taxonomic treatment was published by Boissier (1848, 1859) under the former generic name *Statice *L. Boissier (1848, 1859, 1879) divided the genus into 13 sections and 10 subsections based on taxonomically important morphological features (e.g., habit, leaves, inflorescence and floral traits). However, his classification included only *ca*. 17% of the currently described *Limonium *species. Several authors followed and slightly modified Boissier's classification by simply assigning additional species to his infrageneric units (e.g., Linczevski, 1952; Rechinger & Schiman‐Czeika, 1974; Bokhari & Edmondson, 1982; Karis, 2004), while others segregated some species of *Limonium *(or *Statice*) and assigned them to new genera (e.g., Nevski, 1937; Linczevski, 1952, 1968, 1971, 1979, 1982, 1985; Lledó, Erben, & Crespo, 2003). Other taxonomic attempts have been made to assign *Limonium* species to groups based primarily on morphological affinities. However, these focus on specific geographic regions—such as Europe (Pignatti, 1971, 1972) or smaller areas (e.g., Sicily: Brullo, 1980; Domina & Mazzola, 2003; Croatia: Bogdanović & Brullo, 2015; Greece: Brullo & Erben, 2016)—and lack a global context. In the current paper, we follow and discuss the taxonomic classification of *Limonium *into sections and subsections from Boissier (1848, 1859, 1879), with later revisions and additions by other authors (see Table 2).

**Table 2:** Species of *Limonium* sampled for this study according to the following classifications: division into subgenera follows Lledó, Crespo, et al. (2005), with additional reference to previous subgeneric classification by Pignatti (1971, 1972); division into sections and subsections follows Boissier (1848, 1859, 1879) and later authors. Letters in bold refer to the authors that have assigned the listed species to different subgenera, sections, and subsections

**Genus ** ***Limonium***			
**Sampled species**	***L.*** **subg. ** ***Pteroclados***		
		***L.*** **sect. ** ***Pteroclados***	***L.*** **subsect. ** ***Odontolepideae***
*L. beaumierianum* (Coss. ex Maire) Maire	**L**	**K**	**K**
*L. bonduellei* (T.Lestib.) Kuntze	**P, M**	**Ba, Bo, K, M**	**Ba, Bo, K**
*L. lobatum* (L.f.) Chaz.	**L, P, M**	**B, Ba, Bo, K, R, M**	**B, Ba, Bo, K**
*L. mouretii* (Pit.) Maire	**L, M**	**K, M**	**K**
*L. sinuatum* (L.) Mill.	**L, P, M**	**B, Li, Ba, Bo, BE, I, K, M**	**B, Ba, Bo, K**
			***L.*** **subsect. ** ***Nobiles***
*L. arboreum* (Willd.) Erben, A.Santos & Reyes‐Bet.	**L**	**B, Ba, Bo, K**	**B, Ba, Bo, K**
*L. benmageci* Marrero Rodr.		**Ma**	**Ma**
*L. bourgeaui* (Webb ex Webb) Kuntze		**B, Ba, K**	**B, Ba, K**
*L. brassicifolium* (Webb & Berthel.) Kuntze	**P, M**	**B, Ba, Bo, K, M**	**B, Ba, Bo, K**
*L. frutescens* (Lem.) Erben, A.Santos & Reyes‐Bet.	**L, M**	**K, M**	**K**
*L. imbricatum* (Webb ex Girard) Hubbard ex L.H.Bailey	**M**	**B, Ba, Bo, K, M**	**B, Ba, Bo, K**
*L. macrophyllum* Kuntze	**L**	**B, Ba, Bo, K**	**B, Ba, Bo, K**
*L. macropterum* (Webb & Berthel.) Kuntze		**B, Ba, Bo, K**	**B, Ba, Bo, K**
*L. perezii* (Stapf) Hubbard ex L.H. Bailey		**Ba, Bo, K**	**Ba, Bo, K**
*L. preauxii* (Webb & Berthel.) Kuntze		**B, Ba, K**	**B, Ba, K**
*L. puberulum* (Webb) Kuntze		**B, Ba, Bo, K**	**B, Ba, Bo, K**
*L. redivivum* (Svent.) G.Kunkel & Sunding		**K**	**K**
*L. relicticum* R.Mesa & A.Santos		**Me**	**Me**
*L. spectabile* (Svent.) G.Kunkel & Sunding	**L**	**K**	**K**
*L. sventenii* A.Santos & M.L.Fernández	**L**	**K**	**K**
*L. vigaroense* Marrero Rodr. & R.S.Almeida	**M**	**Ma, M**	**Ma**
	***L.*** **subg. ** ***Limonium***		
		***L.*** **sect. ** ***Circinaria*** (Previously assigned to *Afrolimon*)	
*L. capense* (L.Bolus) L.Bolus		**Li, Ba**	
*L. peregrinum* (P.J.Bergius) R.A.Dyer	**L, M**	**B, Li, Ba, M**	
*L. purpuratum* Hubbard ex L.H.Bailey	**L**	**B, Li, Ba**	
		***L.*** **sect. ** ***Ctenostachys***	
*L. braunii* (Bolle) A.Chev.	**M**	**Ba, M**	
*L. brunneri* (Webb ex Boiss.) Kuntze		**B, Ba**	
*L. fallax* (Coss. ex Wangerin) Maire		**SV**	
*L. mucronatum* (L.f.) Chaz.		**B, SV, Ba, Bo**	
*L. papillatum* (Webb & Berthel.) Kuntze		**B, Ba**	
*L. pectinatum* var. *corculum* (Webb & Berthel.) G.Kunkel & Sunding	**L, M**	**B, Ba, Bo, M**	
*L. pectinatum* var. *divaricatum* (Pit.) G.Kunkel & Sunding	**L, M**	**B, Ba, Bo, M**	
*L. pectinatum* var. *solandri* (Webb & Berthel.) Kuntze	**L, M**	**B, Ba, Bo, M**	
		***L.*** **sect. ** ***Jovibarba***	
*L. jovibarba* (Webb) Kuntze	**L**	**B, Ba, Bo**	
		***L.*** **sect. ** ***Limoniodendron***	
*L. dendroides* Svent.	**L**	**S**	
		***L.*** **sect. ** ***Nephrophyllum***	
* *L. otolepis* (Schrenk) Kuntze	**M**	**R, A, M**	
* *L. perfoliatum* (Kar. ex Boiss.) Kuntze	**M**	**R, A, M**	
* *L. reniforme* (Girard) Lincz.	**M**	**R, A, M**	
		***L.*** **sect. ** ***Iranolimon***	
* *L. anatolicum* Hedge	**M**	**M**	
* *L. carnosum* (Boiss.) Kuntze	**L, M**	**M**	
* *L. iranicum* (Bornm.) Lincz.	**M**	**M**	
* *L. palmyrense* (Post) Dinsm.	**M**	**M**	
* *L. suffruticosum* (L.) Kuntze	**P, M**	**M**	
		***L.*** **sect. ** ***Plathymenium***	***L.*** **subsect. ** ***Chrysanthae***
*L. aureum* (L.) Hill ex Kuntze	**M**	**B, Li, Ba, Bo, M**	**B, Ba, Bo**
*L. sinense* (Girard) Kuntze	**L**	**B, Ba, Bo**	**B, Ba, Bo**
*L. tetragonum* (Thunb.) Bullock	**L**	**B**	**B**
*L. wrightii* (Hance) Kuntze		**Ba**	**Ba**
			***L.*** **subsect. ** ***Rhodanthae***
*L. flexuosum* (L.) Kuntze	**M**	**B, Li, Ba, Bo, M**	**B, Ba, Bo**
*L. tenellum* (Turcz.) Kuntze	**L**	**B, Ba**	**B, Ba**
*L. nudum* (Boiss. & Buhse) Kuntze	**M**	**Ba, R, M**	**Ba**
			(not assigned to any subsection)
*L. dichroanthum* (Rupr.) Ikonn.‐Gal.	**M**	**Li, M**	
*L. hoeltzeri* (Regel) Ikonn.‐Gal.	**M**	**Li, M**	
*L. kaschgaricum* (Rupr.) Ikonn.‐Gal.	**M**	**Li, M**	
		***L.*** **sect. ** ***Polyarthrion***	
*L. caesium* (Girard) Kuntze	**L, P** (subg. * Myriolepis* **)**	**B, Ba**	
*L. insigne* (Coss.) Kuntze	**L, M, P** (subg. *Myriolepis*)	**I, M**	
		***L.*** **sect. ** ***Sarcophyllum*** (Syn.: *L*. sect. *Limonium* subsect. *Sarcophyllae*)	
* *L. anatolicum* Hedge	**M**	**BE, Bo**	
*L. axillare* (Forssk.) Kuntze	**L, M**	**B, Ba, Bo, R, M**	
* *L. carnosum* (Boiss.) Kuntze	**L, M**	**B, Bo, R, Li**	
*L. cylindrifolium* (Forssk.) Verdc. ex Cufod.	**L, M**	**B, Ba, Bo, M**	
* *L. iranicum* (Bornm.) Lincz.	**M**	**R**	
* *L. palmyrense* (Post) Dinsm.	**M**	**Bo**	
*L. somalorum* (Vierh.) Hutch. & E.A.Bruce	**L**	**Ba, L**	
*L. stocksii* (Boiss.) Kuntze	**L, M**	**B, Ba, Bo, R, M**	
* *L. suffruticosum* (L.) Kuntze	**P, M**	**B, Ba, Bo, R, Li**	
		***L.*** **sect. ** ***Schizhymenium***	
*L. echioides* (L.) Mill.	**L, P, M**	**B, SV, Ba, Bo, BE, I, M**	
		***L.*** **sect. ** ***Siphonantha***	
*L. tubiflorum* (Del.) Kuntze	**L, P** (subg. *Myriolepis*)	**B, SV, Ba, Bo**	
		***L.*** **sect. ** ***Sphaerostachys***	
*L. globuliferum* (Boiss. & Heldr.) Kuntze	**L, M**	**B, Ba, Bo, BE, M**	
*L. lilacinum* (Boiss. & Bal.) Wagenitz	**M**	**Bo, BE, M**	
*L*. cf. *pycnanthum* (K.Koch) Kuntze	**M**	**BE, M**	
		***L.*** **sect. ** ***Siphonocalyx*** (Previously assigned to *Eremolimon*)	
*L. sogdianum* Ikonn.‐Gal.	**M**	**Li, M**	
		***L.*** **sect. ** ***Limonium***	***L.*** **subsect. ** ***Genuinae***
*L. brasiliense* (Boiss.) Kuntze	**M**	**B, Ba, M**	**B, Ba**
*L. californicum* (Boiss.) A.Heller		**B, Ba, Bo**	**B, Ba, Bo**
*L. carolinianum* (Walter) Britton	**M**	**B, Ba, Bo, M**	**B, Ba, Bo**
*L. effusum* (Boiss.) Kuntze	**M**	**B, Ba, Bo, BE, M**	**B, Bo, Ba**
*L. gmelini* (Willd.) Kuntze	**P, M**	**B, Ba, Bo, BE, Li, R, M**	**B, Ba, Bo**
*L. guaicuru* (Molina) Kuntze		**Ba**	**Ba**
*L. humile* Mill.	**P**	**B, Ba**	**B, Ba**
* *L. latifolium* (Sm.) Kuntze	**P, M**	**B, Li, Ba, Bo, M**	**Bo**
*L. limbatum* Small		**Ba**	**Ba**
*L. meyeri* (Boiss.) Kuntze	**P, M**	**B, Ba, Bo, BE, Li, R, M**	**B, Ba, Bo**
*L. narbonense* Mill.	**L, P, M**	**BE, M**	**Pa**
*L. tomentellum* (Boiss.) Kuntze	**P, M**	**B, Li, Ba, M**	**B, Li, Ba**
*L. vulgare* Mill.	**L, P, M**	**B, Ba, I, M**	**B, Ba**
			***L.*** **subsect. ** ***Densiflorae***
*L. auriculae‐ursifolium* (Pourr.) Druce	**P**	**B, I**	**B**
*L. camposanum* Erben		**Pa**	**Pa**
*L. gymnesicum* Erben		**Pa**	**Pa**
*L. dodartii* (Girard) Kuntze		**B, Bo**	**B, Bo**
*L. dufourii* (Girard) Kuntze	**L, P**	**B, Bo**	**B, Bo**
*L. gougetianum* (Girard) Kuntze	**P**	**B, Bo**	**B, Bo**
*L. ocymifolium* (Poir.) Kuntze	**P**	**B, BE**	**B**
*L. ovalifolium* (Poir.) Kuntze	**P**	**B, Bo**	**B, Bo**
			***L.*** **subsect. ** ***Dissitiflorae***
*L. aucheri* (Girard) Greuter & Burdet		**B**	**B**
*L. cossonianum* Kuntze		**Pa**	**Pa**
*L. delicatulum* (Girard) Kuntze	**L, P**	**B, SV, Bo, I**	**B, SV, Bo**
*L. graecum* (Poir.) Rech.f.	**P, M**	**B, BE, M**	**B**
*L. minutiflorum* (Guss.) Kuntze	**P**	**B, Bo**	**B, Bo**
*L. rigualii* M.B.Crespo & Erben		**Pa**	**Pa**
*L. roridum* (Sibth. & Sm.) Brullo & Guarino		**B**	**B**
*L. sieberi* (Boiss.) Kuntze		**B, Bo, BE**	**B, Bo**
*L. supinum* (Girard) Pignatti		**B**	**B**
*L. tournefortii* (Boiss.) Erben	**P**	**B**	**B**
			***L.*** **subsect. ** ***Hyalolepideae***
* *L. asparagoides* (Batt.) Maire		**Bat, Ba**	**Ba**
*L. bellidifolium* (Gouan) Dumort.	**M**	**BE, I, M**	**Ba**
*L. dichotomum* (Cav.) Kuntze	**P**	**B, Bo**	**B, Bo**
*L. iconicum* (Boiss. & Heldr.) Kuntze	**M**	**B, Bo, BE**	**B, Bo**
* *L. latifolium* (Sm.) Kuntze	**P, M**	**B, Li, Ba, M**	**B, Ba**
* *L. otolepis* (Schrenk) Kuntze	**M**	**B, Li**	**B**
* *L. perfoliatum* (Kar. ex Boiss.) Kuntze	**M**	**B, Li, Ba**	**B, Ba**
* *L. pruinosum* (L.) Chaz.	**M**	**B, Bat, Ba, Bo, M**	**B, Ba, Bo**
* *L. reniforme* (Girard) Lincz.	**M**	**B, Li**	**B**
* *L. tuberculatum* (Boiss.) Kuntze	**L**	**B, Bat, Ba**	**B, Ba**
			***L.*** **subsect. ** ***Steirocladae***
*L. articulatum* (Loisel.) Kuntze	**P**	**B, Ba, Bo**	**B, Ba, Bo**
*L. bocconei* (Lojac.) Litard.	**P**	**B**	**B**
*L. cancellatum* (Bertol.) Kuntze	**P**	**B**	**B**
*L. cordatum* (L.) Mill.	**P**	**B, Ba, Bo**	**B, Ba, Bo**
*L. cosyrense* (Guss.) Kuntze	**P**	**B**	**Ba**
*L. furfuraceum* (Lag.) Kuntze	**L, P**	**B**	**B**
*L. kraussianum* (Buchinger ex Boiss.) Kuntze		**B, Ba**	**B, Ba**
*L. minutum* (L.) Fourr.	**P**	**B, Ba, Bo, I**	**B, Ba, Bo**
*L. scabrum* (Thunb.) Kuntze		**B, Ba, Bo**	**B, Ba, Bo**
*L. virgatum* (Willd.) Fourr.	**P, M**	**B, Ba, Bo, BE, M**	**B, Ba, Bo**
			***L.*** **subsect. ** ***Pruinosae***
* *L. asparagoides* (Batt.) Maire		**Bat, Ba**	**Bat, SV**
* *L. pruinosum* (L.) Chaz.	**M**	**B, Bat, Ba, Bo, M**	**Bat, SV**
* *L. tuberculatum* (Boiss.) Kuntze	**L**	**B, Bat, Ba**	**SV**
			(not assigned to any subsection)
*L. binervosum* (G.E.Sm.) C.E.Salmon	**P**	**I**	
		(not assigned to any section)	
*L. algarvense* Erben	**M**		
*L. aragonense* (Debeaux ex Willk.) Pignatti	**P**		
*L. biflorum* (Pignatti) Pignatti	**P**		
*L. carpathum* (Rech.f.) Rech.f.	**P**		
*L. confusum* (Godr. & Gren.) Fourr.	**P**		
*L. costae* (Willk.) Pignatti	**P**		
*L. cymuliferum* (Boiss.) Sauvage & Vindt	**P**		
*L. densissimum* (Pignatti) Pignatti	**P**		
*L. frederici* (Barbey) Rech.f.	**P**		
*L. girardianum* (Guss.) Fourr.	**P**		
*L. hungaricum* Klokov	**P**		
*L. lobinii* N.Kilian & Leyens	**M**		
*L. multiflorum* Erben	**P**		
*L. multiforme* (Martelli) Pignatti	**P, M**		
*L. parvibracteatum* Pignatti	**P**		
*L. plurisquamatum* Erben	**P**		
*L. recurvum* C.E.Salmon subsp. *humile* (Girard) Ingr.	**P**		
*L. remotispiculum* (Lacaita) Pignatti	**P**		
*L. sarcophyllum* Ghaz. & J.R.Edm.	**M**		
*Limonium* species included in this study but not assigned to any infrageneric classification= 72 species (Table S2)			

A = Akhani et al. (2013); B = Boissier (1848, 1859, 1879); Ba = Baker (1953a); Bat = Battandier (1888); BE = Bokhari & Edmonson (1982); Bo = Bokhari (1973); I = Ingrouille (1984); K = Karis (2004); L = Lledó, Crespo, et al. (2005); Li = Linczevski (1952, 1979); M = Malekmohammadi et al. (2017); Ma = Marrero and Almeida (2003); Me = Mesa, Santos, Oval, and Voggenreiter (2001); P = Pignatti (1971, 1972); Pa = Palacios et al. (2000); R = Rechinger and Schiman‐Czeika (1974); S = Sventenius (1960); SV = Sauvage and Vindt (1952).

*The species are included twice in this table to represent the alternative classifications.

So far, there remain few molecular phylogenetic studies attempting to clarify relationships within *Limonium*. Most of these studies have a restricted focus either on a specific section (e.g., *Limonium *sect. *Limonium* sensu Boissier, 1848; Palacios et al., 2000) or on a specific geographic region (e.g., Irano‐Turanian region, Akhani, Malekmohammadi, Mahdavi, Gharibiyan, & Chase, 2013). Palacios et al. (2000) examined relationships among 17 sexual and asexual (polyploid) species of *L. *sect. *Limonium* from the Western Mediterranean, and concluded that this section is polyphyletic. Akhani et al. (2013) investigated relationships among Irano‐Turanian *Limonium* species, making taxonomic and biogeographic remarks based on both molecular and morphological data. Lledó, Crespo, et al. (2005) inferred a phylogeny with broader taxonomic sampling of *Limonium*, including 46 species representing all sections defined by Boissier (1848, 1859 ; at least one species per section), plus 24 species from 16 other Plumbaginaceae genera; all species were represented by one sample. According to Lledó, Crespo, et al. (2005), the genus can be divided into two subgenera (*L. *subg. *Pteroclados *and *L. *subg. *Limonium*) corresponding to the two main clades in the phylogeny, while further classification into lower taxonomic units (i.e., sections and subsections) was not possible due to insufficient taxon sampling and phylogenetic resolution. Recently, Malekmohammadi et al. (2017) sampled 76 *Limonium *species (102 accessions) covering many but not all currently accepted sections, and nine species from eight out of 27 other Plumbaginaceae genera. The latter study confirmed the subgeneric division proposed by Lledó, Crespo, et al. (2005) and described a new section in *Limonium *(*L. *sect. *Iranolimon *M. Malekm., Akhani & Borsch) segregated from *L. *sect. *Sarcophyllum* (Boiss.) Lincz. However, Malekmohammadi et al. (2017) acknowledged a lack of comprehensive sampling in terms of infrageneric entities and geographic areas (e.g., Mediterranean region) for the genus*. *Therefore, the need for extended geographic and taxonomic sampling spanning the full breadth of diversity in *Limonium* is clear.

In this study, we infer the largest Plumbaginaceae phylogeny to date in terms of number of genera and species, including more extensive taxon sampling in *Limonium*, the most species‐rich and taxonomically complex genus in the family. Phylogenetic relationships are estimated using one nuclear (ITS) and three chloroplast (*trnL‐F *region, *matK, *and *rbcL *genes) loci to address the following questions:

1. Do taxa identified in previous classifications of Plumbaginaceae and *Limonium* correspond to monophyletic groups?
2. What are the phylogenetic relationships within Plumbaginaceae and *Limonium*? Do phylogenetic clades correspond to morphologically diagnosable groups and/or reflect biogeographic patterns described in previous studies?

By providing a broad phylogenetic framework for Plumbaginaceae and *Limonium*, we improve knowledge of systematics in species‐rich taxa that have undergone multiple taxonomic rearrangements over the past decades. Furthermore, the newly generated, well‐sampled phylogeny will enable future evolutionary and ecological investigations into apomixis, hybridization, biogeography, as well as morphological and ecological specializations in this complex genus.

## 2 MATERIALS AND METHODS

### 2.1 Taxon sampling

We sampled 23 out of the 29 genera assigned to Plumbaginaceae in the latest classification by Hernández‐Ledesma et al. (2015; Table 1); the genera were represented by a variable number of samples (see below). Three monospecific genera and three oligospecific genera (with two or three species) were not sampled, because herbarium specimens are rare, and hence difficult to acquire and sample. For *Limonium*, the most species‐rich genus of Plumbaginaceae and the focus of this study, sampling was designed to cover its taxonomic and geographic diversity*. *Two hundred and three taxa (representing 201 species, one subspecies and three varieties) were sampled, including representatives from all sections and subsections described by Boissier (1848, 1859, 1879) and later authors (see Table 2), and spanning the full geographic breadth of this cosmopolitan genus (Table S1). The exact percentage of sampled *Limonium *species per section cannot be provided due to the plethora of unclassified species (e.g., see Table S2). Additionally, 64 species of Plumbaginaceae genera other than *Limonium, *representing the subfamilies Plumbaginoideae and Limonioideae, were included in the study (Table 1). Finally, 20 species from the sister family Polygonaceae (APG IV, 2016) were used as outgroups to reach a total sampling of 287 taxa.

Plant material was obtained from many different sources (Data S1). First, fieldwork was conducted in Greece, Turkey, Spain, Portugal, and Macaronesia, where many *Limonium* species were collected, as well as one species of *Myriolimon *Lledó, Erben & M.B.Crespo. Fresh leaves were stored in silica gel and/or press‐dried as part of herbarium specimens. Second, a large number of dried leaf samples were requested and acquired from collections of several herbaria (ATH, AZB, E, L, LISC, ORT, P, U, UPA, WAG, Z, ZT). Third, fresh material for several Plumbaginaceae species was obtained from living collections of Botanical Gardens, especially from the Botanical Garden of the University of Zurich. In the latter, some Plumbaginaceae species were grown from seeds obtained through the exchange program of seed collections among botanical gardens. Fourth, DNA samples were provided by the Plant DNA Bank of Kew Gardens. Finally, 60 taxa with published DNA sequences were added to complement our sampling.

### 2.2 Molecular sampling

The chloroplast *trnL‐F* region (i.e., the *trnL* intron, the 3’ *trnL *exon, and the *trnL*‐*trnF* spacer), *rbcL* and *matK* genes, and the nuclear ITS region (i.e., the internal transcribed spacer 1, 5.8S rRNA gene, and the internal transcribed spacer 2) compose the genetic dataset in this study. The choice of genetic loci was informed by the availability of pre‐existing sequences for genetic markers generated in previous Plumbaginaceae and *Limonium* studies (e.g., Lledó et al., 1998; Lledó et al., 2000; Lledó et al., 2001; Lledó, Crespo, et al., 2005; Palacios et al., 2000; Ding, Zhang, Yu, Zhao, & Zhang, 2012; Akhani et al., 2013; Moharrek, Osaloo, & Assadi, 2014) and by a pilot study that we conducted on 12 Plumbaginaceae taxa (ten *Limonium *taxa: three of *L. *subg. *Pteroclados* and seven of *L. *subg. *Limonium, *following the subgeneric division by Lledó, Crespo, et al., 2005), and two taxa from other Plumbaginaceae genera: one for each of the two subfamilies). In the pilot study, in addition to *rbcL*, *trnL‐F, *and ITS regions used frequently in existing Plumbaginaceae phylogenies, we also explored the adequacy of some DNA barcoding regions for plants (*accD*, *matK*, *ndhJ*, *rpoB*, *rpoC1*) proposed by Ford et al. (2009). We found that the short coding regions of *accD, ndhJ*, *rpoB,* and *rpoC1* had very few variable sites and provided little phylogenetic resolution. Phylogenies were better resolved when using *rbcL*, *matK, trnL‐F *and ITS regions, especially in combination. Thus, the selected sampling scheme of this study complements, significantly expands both taxon and gene sampling for Plumbaginaceae and *Limonium *in particular, with regard to previous global Plumbaginaceae/*Limonium* datasets (Lledó, Crespo, et al., 2005; Malekmohammadi et al., 2017; Table 3), and provides sufficient resolution at the taxonomic level of interest (primarily among genera of Plumbaginaceae and sections of *Limonium*).

**Table 3:** Comparison of previous large phylogenies of Plumbaginaceae with a focus on *Limonium* and the new phylogeny of this article (Koutroumpa et al.)

Taxon Sampling	Lledó, Crespo, et al. (2005)	Malekmohammadi et al. (2017)	Koutroumpa et al.
*Limonium* species	48	76	201
Other Plumbaginaceae species	22	9	64
Plumbaginaceae genera^a^	18	10	23
Polygonaceae outgroups	–	–	20

Molecular Sampling	Lledó, Crespo, et al. (2005)	Malekmohammadi et al., (2017)	Koutroumpa et al.
cpDNA markers	2 (*rbcL, trnL‐F*)	3 (*trnL‐F,* *trnK‐matK* *, petD*)	3 (*trnL‐F, rbcL, matK*)
nrDNA markers	–	1 (ITS)	1 (ITS)

a According to Hernández‐Ledesma et al. (2015) classification.

### 2.3 DNA extraction, amplification, and sequencing

Prior to DNA isolation, dried leaves were ground into a fine powder using metal beads and a Mixer Mill MM301 (Retsch GmbH, Haan, Germany). DNA was extracted using a modified cetyltrimethylammonium bromide (CTAB) method (Doyle & Doyle 1987; Data S2) and visualized in a 1% agarose gel. As expected, DNA samples from old herbarium specimens were generally more degraded than those from recently collected leaf material, which gave higher molecular weight DNA. In order to overcome problems with PCR amplification of entire loci when DNA samples of relatively low quality were used as template (e.g., degraded DNA from old herbarium specimens), we attempted to amplify some loci in two partially overlapping fragments. For this, we used two primer pairs, each pair composed of a primer hybridizing at the 5’ or 3’ end of the locus and one hybridizing within the locus (“internal” primer). For the *trnL‐F* region, we used primers *c *and *f* and the internal primers *d *and *e *(Taberlet, Gielly, Pautou, & Bouvet, 1991), and for the *rbcL *gene, we used primers *1F *and *1368R* and the internal primers *636F* and *724R* (Lledó et al., 1998). The *matK* gene was amplified with either *matK X* and *matK 5* primer pair (Ford et al., 2009) or *390F *and *CAR_R *primers (Cuénoud et al., 2002; Dunning & Savolainen, 2010). For the ITS region, we primarily used *ITS5 *and *ITS4* primers (White, Bruns, Lee, & Taylor, 1990), but due to problems of fungal contaminations for some samples, we additionally used the recently developed primers *ITS‐p5 *and *ITS‐u4 *and the internal primers *ITS‐p3 *and *ITS‐u2* (Cheng et al., 2016). Each PCR was performed in a final volume of 20 μl, containing 12 μl of ddH_2_O, 4 μl of Taq‐Buffer (5×, 7.5 mM MgCl_2_), 0.2 μl of Taq‐Polymerase (5 U/μl), 0.8 μl of dNTPs (10 mM), 1 μl of each primer (10 μM), and 1 μl of DNA template. For the *trnL‐F* region, we additionally used 1 μl DMSO (5%), and for *rbcL *and *matK, *1.2 μl MgCl_2 _(~0.025 M). The volume of any additional reagent was subtracted from ddH_2_O. The PCR amplification program for *trnL‐F *and *rbcL *had a first denaturation step of 5 min at 95°C and 35 cycles of: 1 min at 94°C, 1 min at 53°C, and 2 min at 72°C. For *matK, *there was an initial denaturation of 4 min at 95°C followed by 35 cycles of: 1 min at 94°C, 1 min at 52°C, and 1.5 min at 72°C. For the ITS amplification using the primers *ITS5 and ITS4,* we used a program of 1 min at 94°C and 40 cycles of: 30 s at 94°C, 40 s at 53°C, and 40 s at 72°C, whereas for primers *ITS‐p5 *and *ITS‐u4, *we used a program of 4 min at 94°C followed by 34 cycles of: 30 s at 94°C, 40 s at 55°C, and 1 min at 72°C. All PCR protocols included a final extension step of 10 min at 72°C. The purification of successfully amplified PCR products was performed using Exonuclease I (ExoI) and FastAP Thermosensitive Alkaline Phosphatase (FastAP) (Thermo Scientific, Waltham, USA). For cycle sequencing, BigDye Terminator Mix (Applied Biosystems, Inc., Foster City, California, USA) and the same primers listed above were used, and the PCR program consisted of 25 cycles of 10 s at 96°C, 5 s at 50°C, and 4 min at 60°C. Finally, ABI 3100 Genetic Analyzer (Applied Biosystems, Foster City, California, USA) was used to obtain both forward and reverse sequences for each PCR product of the four loci under study.

### 2.4 Data analysis and phylogenetic inference

Forward and reverse complement strands of sequences were assembled and edited with Sequencher v.5.0.1 (Gene Codes Corp., Ann Arbor, Michigan, USA), resulting in reliable consensus sequences. These sequences were compared with the public sequence database using the BLASTn‐NCBI in order to verify their identity and check for any contamination. Sequences were aligned with MAFFT v.7 (Katoh & Standley, 2016) and default parameters (“Auto”) for *rbcL*, *matK,* and ITS, and with the progressive method “G‐INS‐1” for *trnL‐F* dataset. All alignments were checked and edited manually in BioEdit v.5.0.6 (Hall, 2001). In addition, we used the Recombination Detection Program (RDPv.4; Martin, Murrell, Golden, Khoosal, & Muhire, 2015) to check for recombination in the aligned ITS sequences of *Limonium*, which could affect phylogenetic reconstruction and account for potential incongruences between chloroplast and nuclear markers; recombination was not detected in our dataset.

Datasets for the three chloroplast loci and one nuclear locus including newly generated and pre‐existing sequences were initially analyzed separately. Phylogenies were inferred for each of the four datasets using the maximum likelihood (ML) criterion as implemented in RAxML v.8.2.9 (Stamatakis, 2014). We carried out 40 ML searches using a different maximum parsimony starting tree each time, used a generalized time‐reversible model of nucleotide substitution with gamma‐distributed rates across sites (GTR +Γ, Tavaré, 1986; Yang, 1993, 1994), and performed a rapid bootstrap analysis with 1,000 replicates. The bootstrap replicate trees were then used to draw bipartition information (i.e., confidence values) on the best ML tree found among the 40 independent ML searches. Gene trees from the three chloroplast markers were inspected for conflicting relationships; in the absence of any well‐supported incongruence (i.e., bootstrap values (bs) ≥ 80%), the three datasets were combined for further analyses (hereafter, cpDNA dataset). ML analysis of the cpDNA dataset was done by partitioning the dataset per locus (*trnL‐F, rbcL,* and *matK*) and assigning a GTR +Γ model to each of the three partitions. We additionally conducted 40 ML searches and an analysis of 1,000 rapid bootstrap replicates to infer the cpDNA tree.

Furthermore, cpDNA and ITS datasets were analyzed using Bayesian MCMC inference (BI; Yang & Rannala, 1997) in MrBayes v.3.2.6 (Ronquist et al., 2012). For each dataset, we performed two independent runs with four chains (one cold and three incrementally heated). Chains were run for 10,000,000 generations each, while parameters and trees were sampled every 5,000th generation. In order to account for uncertainty of DNA substitution model, we employed a model‐averaging approach using a reversible‐jump MCMC algorithm (rjMCMC; Huelsenbeck, Larget, & Alfaro, 2004) that allows the chain to explore all the possible models of the GTR family (203 models). The number of times that a chain visits a model is relative to its marginal probability. It has been shown that this method results in less biased estimates for the parameters sampled during the MCMC, including tree topology and clade posterior probabilities (Alfaro & Huelsenbeck, 2006). This rjMCMC algorithm +Γ distributed rates (MrBayes command: “lset nst=mixed rates=gamma”) was assigned to ITS and to every partition (i.e., locus) in the cpDNA dataset. MCMC diagnostics for the independent and combined runs (e.g., convergence of parameter estimates and effective sample sizes >200) were assessed using Tracer v.1.5 (Rambaut & Drummond, 2007). The two runs of each analysis were then combined discarding the initial 25% of the sampled parameters and trees as burn‐in. The resulting BI and ML trees of the cpDNA and ITS datasets were compared to detect potential incongruences between well‐supported clades, which are here defined as: (a) clades with both high posterior probability (pp) and bootstrap values (i.e., pp ≥ 0.95 and bs ≥ 80%) and (b) clades with either one of those values high and the other moderate (i.e., pp ≥ 0.95 and bs = 70%–79%; or bs ≥ 80% and pp = 0.85–0.94).

We also employed a total evidence approach by combining sequences of all four loci into a single supermatrix. Well‐supported topological conflicts between the chloroplast and nuclear datasets were relatively few and usually at a phylogenetic scale shallower (i.e., toward the tips of the tree) than the desired level of phylogenetic inference (i.e., corresponding to the level of intergeneric relationships for Plumbaginaceae and intersectional relationships for *Limonium*). Therefore, a total evidence approach was appropriate to resolve phylogenetic relationships at the level of investigation. However, where necessary, the inferred phylogenetic relationships are presented and discussed separately in cases of well‐supported phylogenetic conflicts between cpDNA and nuclear ITS signals (see below “Mediterranean lineage”). Both ML and BI analyses were conducted on a reduced supermatrix that excluded six “rogue taxa” (i.e., taxa in different well‐supported clades of the cpDNA and ITS phylogenies; see Results). This was done in order to facilitate analyses and recover the monophyly of clades previously containing the “rogue taxa.” In ML analysis of the reduced supermatrix, we used a GTR +Γ model for every locus (four partitions) and conducted 40 ML inferences and 1,000 rapid bootstrap searches. For the BI on the same dataset and partitioning for every locus, rjMCMC algorithm was used for model averaging +Γ and two independent runs of 10,000,000 iterations were employed. All trees were visualized and rooted using FigTree v.1.4.2 (Rambaut, 2007), Dendroscope v.3.5.9 (Huson & Scornavacca, 2012), and Archaeopteryx 0.9921 (Han & Zmasek, 2009) in order to verify the correct assignment of support values on the nodes after rooting, as suggested by Czech, Huerta‐Cepas, and Stamatakis (2017).

## 3 RESULTS

### 3.1 Genetic datasets and phylogenetic analyses

Six hundred and ninety‐four sequences were generated for this study of Plumbaginaceae, comprising 189 new *trnL‐F *sequences (GenBank: MH560967–MH561155), 172 new *rbcL *sequences (GenBank: MH582667–MH582838), 179 new *matK *sequences (GenBank: MH582839–MH583017), and 154 new ITS sequences (GenBank: MH582513–MH582666; see also Data S3). Additionally, 270 pre‐existing sequences were retrieved from GenBank or provided to us by the authors of Lledó, Crespo, et al. (2005; Data S3). For some taxa, problems in amplification and sequencing resulted in either the complete absence of some sequences or the generation of only a part of the full sequences (for *trnL‐F, rbcL, *and ITS*, *where internal primers were available). Information about data matrices of individual and combined loci can be found in Table 4. The ITS region exhibits the highest amount of potentially informative characters (56%), followed by *matK *(34%), *trnL‐F* (28%), and *rbcL* (18%). The cpDNA dataset and the reduced supermatrix contained 26% and 32% potentially informative characters, respectively.

**Table 4:** Composition of multiple sequence alignment matrices for each locus separately, the combined plastid loci (cpDNA), and the combined chloroplast and nuclear loci excluding six *Limonium* ***“***rogue taxa**”** (i.e.**,** reduced supermatrix)

	*trnL‐F*	*rbcL*	*matK*	ITS	cpDNA	Reduced supermatrix
Number of sequences (= taxa)	269	241	215	238	281	281
Number of *Limonium *taxa	193	188	168	160	200	197
Number of Plumbaginaceae taxa other than *Limonium*	60	41	30	58	62	64
Number of outgroup taxa (Polygonaceae)	16	12	17	20	19	20
Number of characters in the alignment	1,472	1,267	847	896	3,586	4,481
Amount of variable characters (%)	38%	29%	42%	66%	36%	42%
Amount of informative characters (%)	28%	18%	34%	56%	26%	32%

For each dataset, phylogenies produced with ML and BI methods showed very similar topologies, with moderate (pp = 0.85–0.94 for BI and bs = 70%–79% for ML) and strongly supported nodes (pp ≥ 0.95 for BI and bs ≥ 80% for ML) being largely identical. We present the Bayesian 50% majority‐rule trees in all figures with posterior probabilities and bootstrap support values noted at the nodes. The chloroplast (cpDNA) and nuclear (ITS) phylogenetic trees were generally similar, while some topological differences were mostly found between non‐supported nodes and only a few exceptions of well‐supported incongruences were observed (see below). The ITS Bayesian tree showed better resolution (146 out of 237 nodes resolved in the 50% majority‐rule tree; Figure S1) compared to the cpDNA tree (132 out of 280 nodes resolved; Figure S2), in accordance with the higher number of informative characters in the nuclear dataset (Table 4). The tree based on the reduced supermatrix exhibited highest resolution with 166 out of 280 nodes resolved in the 50% majority‐rule Bayesian tree. Thus, phylogenetic relationships are presented and discussed based on the tree from the reduced supermatrix dataset, except for the “Mediterranean lineage” of *Limonium, *where both cpDNA and ITS phylogenies are presented to account for well‐supported topological discrepancies between the two datasets.

### 3.2 Phylogeny of Plumbaginaceae

**Figure 1:**
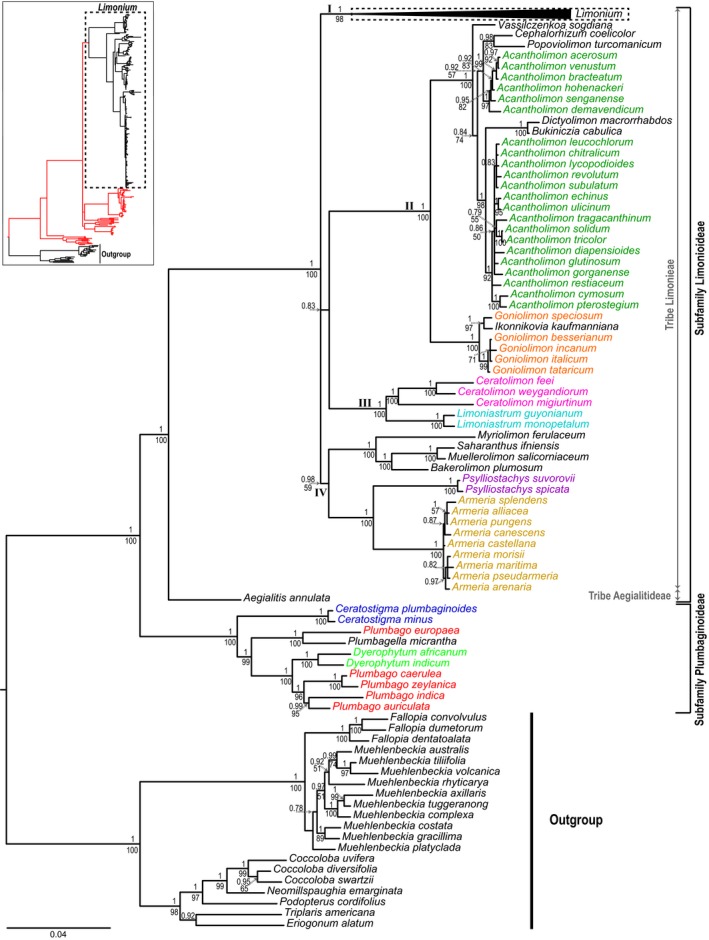
Large tree: Phylogeny of Plumbaginaceae with Polygonaceae outgroups inferred from Bayesian analysis of the reduced (excluding six “rogue taxa”; see Methods) supermatrix consisting of concatenated sequences of chloroplast and nuclear loci. In the 50% majority‐rule tree posterior probabilities above 0.7 and bootstrap support values above 50% estimated from MrBayes and RAxML analyses are reported above and below the branches, respectively. Different colors were used for Plumbaginaceae genera (other than *Limonium*) represented by more than one species and Roman numerals (I–IV) are assigned to the major clades in Limonieae. Small tree: phylogenetic framework showing the topology of *Limonium* (black branches surrounded by a dashed rectangle) in the context of Plumbaginaceae (red branches). For phylogenetic relationships within *Limonium*, see Figures 2 and 3

The Plumbaginaceae are divided into the two monophyletic subfamilies Plumbaginoideae (pp = 1, bs = 100%) and Limonioideae (pp = 1, bs = 100%; Figure 1). In Plumbaginoideae, both *Ceratostigma* Bunge and *Dyerophytum* Kuntze are monophyletic (pp = 1, bs = 100%), whereas *Plumbago* is not. Specifically, *Plumbago europaea* L. is sister to *Plumbagella* (pp = 1, bs = 100%), while other *Plumbago* species form a sister clade to *Dyerophytum* (pp = 1, bs = 100%). *Plumbago, Plumbagella, *and *Dyerophytum *form a clade (pp = 1, bs = 99%) sister to *Ceratostigma *(Figure 1).

In Limonioideae, *Aegialitis* (tribe Aegialitideae) is sister to a well‐supported clade (pp = 1, bs = 100%) consisting of genera of the tribe Limonieae (Figure 1). In this clade, *Armeria*, *Limoniastrum* Fabr., *Ceratolimon *M.B.Crespo & M.D.Lledó, *Psylliostachys* (Jaub. & Spach) Nevski and *Limonium* are monophyletic, whereas *Acantholimon* and *Goniolimon *Boiss. are not. Limonieae consist of four mostly well‐supported major clades (see clades I–IV; Figure 1), forming a tetratomy (sister relationship of clades II and III is not well‐supported, bs < 50% and pp = 0.83; Figure 1). In clade IV, *Armeria *(pp = 1, bs = 100%) is sister to *Psylliostachys *(pp = 1, bs = 100%) and together they are sister to a clade (pp = 1, bs = 100%) comprised of *Saharanthus*, *Myriolimon, Bakerolimon, *and *Muellerolimon*. In clade III, *Ceratolimon* and *Limoniastrum* are reciprocally monophyletic sister lineages (pp = 1, bs = 100%). In clade II, *Goniolimon* as currently circumscribed is paraphyletic, with *Ikonnikovia *nested in it. *Goniolimon *and *Ikonnikovia *(pp = 1, bs = 100%) are sister to a clade formed by *Acantholimon, Vassilczenkoa, Cephalorhizum, Popoviolimon, Dictyolimon,* and *Buckiniczia *(pp = 1, bs = 100%). There are two monophyletic groups of *Acantholimon *species (pp = 1, bs = 92% and pp = 1, bs = 97%, respectively): One is part of a well‐supported clade (pp = 1, bs = 98%) sister to a clade comprised of *Dictyolimon *and *Bukiniczia *(pp = 1, bs = 100%), and the other forms part of a moderately to poorly supported clade (pp = 0.92 and bs = 57%, respectively) that also contains the sister genera *Cephalorhizum *and *Popoviolimon *(pp = 0.98, bs = 93%; Figure 1)*.*

### 3.3 Phylogeny of ***Limonium***

**Figure 2:**
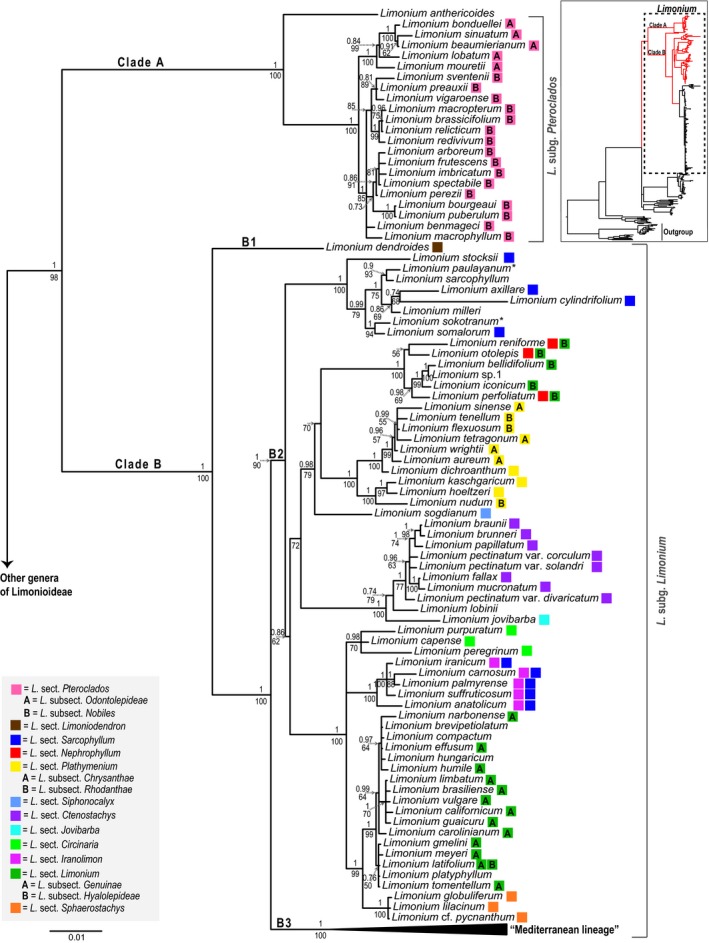
Large tree: Phylogeny of *Limonium* clades A and B inferred from Bayesian analysis of the reduced (excluding six “rogue taxa”; see methods) supermatrix consisting of concatenated sequences of chloroplast and nuclear loci. In the 50% majority‐rule tree posterior probabilities above 0.7 and bootstrap support values above 50% estimated from MrBayes and RAxML analyses are reported above and below the branches, respectively. The division into subgenera is according to Lledó, Crespo, et al. (2005). Colored squares next to species names denote sections and letters inside the squares denote subsections according to Boissier and other authors’ classification (see Table 2). Double squares next to species names indicate alternative classifications, with the left square referring to the most recent one. Species without a colored square are not assigned to any section or subsection. Asterisks next to species indicate well‐supported conflicting topologies between chloroplast and nuclear trees: *L. sokotranum* and *L. paulayanum* are sisters in the cpDNA tree (see also Figures S1 and S2). Small tree: phylogenetic framework showing relationships of clades A and B (red branches) and the Mediterranean subclade (black branches) of *Limonium* (dashed rectangle) within Plumbaginaceae

**Figure 3:**
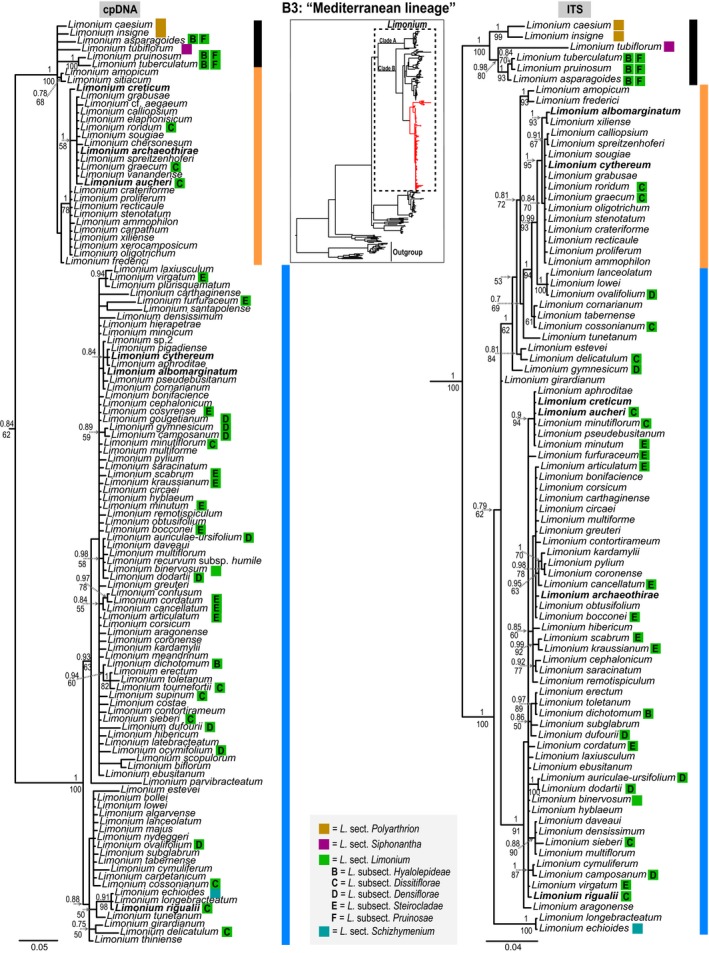
Large trees: Phylogenies of the “Mediterranean lineage” of *Limonium* inferred from Bayesian analyses of concatenated chloroplast DNA (left) and ITS (right) sequences, respectively. In the 50% majority‐rule trees posterior probabilities above 0.7 and bootstrap support values above 50% estimated from MrBayes and RAxML analyses are reported above and below the branches, respectively. Colored squares next to species names denote sections and letters inside the squares denote subsections according to Boissier and other authors’ classification (see Table 2). Double squares next to species names indicate alternative classifications, with the left square referring to the most recent one. Species without a colored square are not assigned to any section or subsection. Vertical colored bars are used to highlight well‐supported topological conflicts between clades (“rogue clades”), boldfaced species indicate well‐supported conflicting topologies for individual species (“rogue taxa”) in the two trees. Small tree: phylogenetic framework showing the position of the Mediterranean subclade (red branches) of *Limonium* (dashed rectangle) within Plumbaginaceae

*Limonium* forms a strongly supported clade (pp = 1, bs = 98%; clade I in Figure 1) with the species previously assigned to *Afrolimon, *now *L. *sect. *Circinaria* (Boiss.) M.Malekm., nested in it (Figure 2)*.* *Limonium* is divided into two major clades (A and B; Figure 2). In clade A, *L. *sect. *Pteroclados *(Boiss.) Bokhari (= *L. *subg. *Pteroclados *sensu Lledó, Crespo, et al., 2005) is sister to *L. anthericoides* (Schltr.) R.A.Dyer (pp = 1, bs = 100%)*,* and divided into the two reciprocally monophyletic subsections, *L. *sect. *Pteroclados *subsect. *Odontolepideae *and subsect. *Nobiles *sensu Boissier (1848; pp = 1, bs = 100% and pp = 1, bs = 85%, respectively). In clade B, the monotypic *L. *sect. *Limoniodendron *Svent. (*L. dendroides *Svent.; subclade B1) is sister to two well‐supported subclades (B2: pp = 1, bs = 90% and B3: pp = 1, bs = 100%; Figure 2). Clade B2 consists of taxa assigned to *L. *sect. *Sarcophyllum*, *L. *sect. *Nephrophyllum *Rech.f.*, L. *sect. *Limonium, L. *sect. *Plathymenium *(Boiss.) Lincz.*,* *L. *sect. *Siphonocalyx *Lincz., *L. *sect. *Ctenostachys *(Boiss.) Sauvage & Vindt*, L. *sect. *Jovibarba *sensu Boissier (1848)*, L. *sect. *Circinaria, L. *sect. *Iranolimon *and *L. *sect. *Sphaerostachys *(Boiss.) Bokhari (Figure 2)*. *Clade B3 comprises taxa from *L. *sect. *Polyarthrion *(Boiss.) Sauvage & Vindt*, L. *sect. *Siphonantha *(Boiss.) Sauvage & Vindt*, L. *sect. *Limonium *and *L. *sect. *Schizhymenium *(Boiss.) Bokhari (Figure 3)*.*

At the sectional level, apart from the monotypic *Limonium *sect. *Jovibarba, L. *sect.* Limoniodendron, L. *sect. *Schizhymenium* and *L. *sect. *Siphonantha,* and the *L. *sect. *Siphonocalyx* represented here by only one species, the *L. *sect. *Pteroclados, L. *sect.* Plathymenium, L. *sect.* Ctenostachys*, *L. *sect. *Circinaria, L. *sect. *Iranolimon, L. *sect.* Sphaerostachys, *and *L. *sect. *Polyarthrion *(in the ITS tree; Figure 3) are strongly supported as monophyletic (Figures 2 and 3). The remaining sections, namely *L. *sect. *Sarcophyllum, L. *sect. *Nephrophyllum, *and *L. *sect. *Limonium *are strongly supported as non‐monophyletic (Figures 2 and 3). It should be noted that slightly fewer than half of the *Limonium *taxa used in this phylogeny (72 out of 203 taxa) were not assigned to any subgenera, sections, and subsections by the authors who described them or later authors who studied the infrageneric classification of *Limonium *(see Table 2 and Supporting information Table S1)*.*

In clade B2 (Figure 2), *Limonium *sect. *Sarcophyllum* sensu Linczevski (1952) is polyphyletic with representatives in two separate and well‐supported clades (pp = 1, bs = 100%), one formed by *L. cylindrifolium *(Forssk.) Verdc. ex Cufod.*, L. axillare *(Forssk.) Kuntze*, L. somalorum *(Vierh.) Hutch. & E.A.Bruce*, L. stocksii *(Boiss.) Kuntze and the unclassified *L. sokotranum *(Vierh.) Radcl.‐Sm.*, L. paulayanum *(Vierh.) Ghaz. & J.R.Edm.*, L. sarcophyllum* Ghaz. & J.R.Edm. and *L. milleri* Ghaz. & J.R.Edm.*, *and the other formed by *L. carnosum *(Boiss.) Kuntze*, L. iranicum *(Bornm.) Lincz., *L. suffruticosum *(L.) Kuntze*, L. anatolicum *Hedge, and *L. palmyrense *(Post) Dinsm. The former clade is sister to a moderately to poorly supported clade (pp = 0.86 and bs = 62%, respectively) comprising all other *Limonium *taxa in clade B2, including the latter clade. This latter clade, which was recently assigned to the newly formed *L. *sect. *Iranolimon*, is part of a clade comprising *L. *sect. *Circinaria*, *L. *sect. *Sphaerostachys, *and *L. *sect. *Limonium *subsect. *Genuinae *sensu Boissier (1848; pp = 1, bs = 100%). *Limonium *sect. *Plathymenium *is monophyletic, but its subsections, *L. *subsect. *Chrysanthae *and *L. *subsect. *Rhodanthae *sensu Boissier (1848), are not. *Limonium *sect. *Plathymenium* is sister to a clade formed by species assigned to *L. *sect. *Nephrophyllum *and *L. *sect. *Limonium *subsect. *Hyalolepideae *sensu Boissier (1848)*,* and these sister lineages together with *L. sogdianum *Ikonn.‐Gal. (*L. *sect. *Siphonocalyx*) form a well‐supported clade (pp = 0.98, bs = 79%).* Limonium *sect. *Ctenostachys* is monophyletic and sister to *L. lobinii* N.Kilian & Leyens, and the combined clade is sister to *L. *sect. *Jovibarba*; all together form a well‐supported clade (pp = 1, bs = 100%)*. *In clade B3 (Figure 3) of the ITS tree, *L. *sect. *Polyarthrion *is monophyletic and sister to a well‐supported clade comprising *L. *sect. *Siphonantha *and *L. *sect. *Limonium *subsect. *Hyalolepideae/Pruinosae *(pp = 0.98, bs = 80%), but these relationships are not corroborated by the cpDNA tree, which leaves the relationships of *L. *sect. *Polyarthrion *unresolved. In its currently accepted circumscription (Boissier, 1848), *L. *sect. *Limonium *is polyphyletic and, of its subsections, *L. *subsect. *Genuinae *is monophyletic (considering the latest classification for *L. latifolium* (Sm.) Kuntze; see Figure 2), *L. *subsect. *Pruinosae *(Batt.) Sauvage & Vindt is well‐supported as monophyletic in the ITS tree, *L. *subsect. *Hyalolepideae *is clearly non‐monophyletic, with its representatives found in both clades B2 and B3, and *L. *subsect. *Densiflorae, L. *subsect.* Dissitiflorae, *and *L. *subsect. *Steirocladae *sensu Boissier (1848) have their representatives in a large clade with many unsupported nodes consisting almost exclusively of Mediterranean endemic species on short branches (many of them are “microspecies”; Figure 3). All three of *L. *subsect. *Densiflorae, L. *subsect. *Dissitiflorae *and *L. *subsect. *Steirocladae* are non‐monophyletic based on well‐supported nodes.

The cpDNA and ITS trees of the “Mediterranean lineage” (= clade B3; Figure 3) show incongruences between some well‐supported clades (“rogue clades”). In the cpDNA phylogeny, North African/Iberian species (black bar) are included in the same clade with species from the Aegean region (orange bar) and this clade is sister to the clade comprising the rest of the Mediterranean species (blue bar). Conversely, in the ITS tree the North African/Iberian clade (black bar) is sister to a clade consisting of the Aegean species (orange bar) and the other Mediterranean species (blue bar). In addition, six species (“rogue taxa”; in bold letters in Figure 3) show incongruences in their phylogenetic placement between strongly supported clades in trees from different organellar genomes, while another case of well‐supported conflict at a shallower phylogenetic level between sister species is noted with an asterisk in Figure 2 (details in figure legend).

## 4 DISCUSSION

Our study represents the phylogeny of Plumbaginaceae with the highest number of genera, species, and sequences sampled to date. The major findings for Plumbaginaceae genera are the confirmed lack of monophyly for *Plumbago*, and the phylogenetic positions of *Plumbagella *sister to *Plumbago europaea,* *Ikonnikovia *nested within *Goniolimon*, and *Muellerolimon *placed within a well‐supported clade comprised of *Saharanthus *(sister to *Muellerolimon*), *Bakerolimon, *and *Myriolimon*. This is also the first study to sample about one third of the currently accepted species of *Limonium*, covering all described infrageneric entities and including a large number of Mediterranean endemics that comprise 70% of the genus, but were never sampled extensively in previous phylogenetic studies (Lledó, Crespo, et al., 2005; Malekmohammadi et al., 2017). The main implications of our phylogenetic results for the infrageneric classifications of *Limonium *are: the composition of one of the two major clades in *Limonium* phylogeny (Clade A) that do not strictly match *L. *subg. *Pteroclados s.s.* (i.e., = *L. *sect. *Pteroclados*), but additionally includes *L. anthericoides *as sister to it; the subdivision of *L. *subg. *Limonium *(Clade B) into three well‐supported subclades (i.e., B1: *L. *sect. *Limoniodendron*, B2: mostly non‐Mediterranean *Limonium *species assigned to several sections, and B3: “Mediterranean lineage”); the identification of a new section of *Limonium *comprising *L. anthericoides*; and the new circumscription of *L. *sect. *Limonium *currently corresponding to *L. *sect. *Limonium *subsect. *Genuinae*. Our results confirm many previously published findings, provide new insights, and set the basis for taxonomic revisions of Plumbaginaceae and *Limonium*. Below we discuss the systematic implications of all clades supported by our analyses in light of morphological and biogeographic characteristics identified by previous authors.

### 4.1 Subfamily Plumbaginoideae

The monophyly of Plumbaginaceae and the division of the family into two subfamilies, Plumbaginoideae and Limonioideae, are confirmed in this study and are in agreement with previous phylogenetic results based on more limited taxon and molecular sampling (Lledó et al., 1998, 2001). This is the first phylogenetic study to sample all four genera of Plumbaginoideae, including the monospecific *Plumbagella*. Here, *Ceratostigma *and *Dyerophytum *are clearly monophyletic, while *Plumbago *forms a non‐monophyletic assemblage (Figure 1). *Ceratostigma* is characterized by non‐glandular, tubular, 5‐ribbed calyx, with 10‐nerved calyx base, and glabrous style (Kubitzki, 1993), while *Dyerophytum* is characterized by non‐glandular, segmented calyx, bearing sepals with strong midrib and reflexed, wide margins, and hairy style (Kubitzki, 1993). The polyphyly of *Plumbago* is confirmed in the current study, which includes six more species ascribed to Plumbaginoideae in addition to the four species of the same subfamily employed by Lledó et al. (2001) and Lledó, Crespo, et al. (2005). *Plumbago *and *Plumbagella *have glandular calyces, which is a diagnostic trait, distinct for the family (Kubitzki, 1993). However, *Plumbagella, *which is the only annual herb of Plumbaginoideae, has calyces deeply divided into five lobes bearing glands and glabrous calyx tube (eFloras, 2008). According to our results, the circumscription of *Plumbago *is challenged and its generic boundaries should be either extended to accommodate *Plumbagella *and *Dyerophytum *or a new generic name should be assigned to the *Plumbago *clade that does not include the type for the genus (*Plumbago europaea*). A more comprehensive taxon sampling and a revision of diagnostic morphological characters for *Plumbago *are needed before a formal generic revision is proposed.

Our topology newly suggests a biogeographic disjunction between temperate and tropical/subtropical taxa. Specifically, *Plumbago europaea *and *Plumbagella micrantha *(Ledeb.) Spach*, *occurring predominantly in temperate regions of Eurasia, form a clade sister to the clade comprising the other *Plumbago *species and *Dyerophytum *(Figure 1)*, *which occur in tropical and subtropical regions of the Old (*D. africanum *(Lam.) Kuntze*, D. indicum *(Gibs. ex Wight) Kuntze*, Plumbago indica *L.*, Plumbago auriculata *Lam. and *Plumbago zeylanica* L.) and New World (*Plumbago caerulea *Kunth and *Plumbago zeylanica*). All representatives of Plumbaginoideae occur in the Old World, apart from three out of ca. 20 species of *Plumbago *that occur in the New World. Based on our current sampling, the phylogenetic placement of the neotropical *Plumbago zeylanica *and *Plumbago caerulea, *embedded in a clade otherwise formed by paleotropical taxa, is consistent with a pattern of colonization of the New World from the Old World. More extensive sampling of *Plumbago* species from throughout the tropics is required to clarify the biogeographic history of the subfamily.

### 4.2 Subfamily Limonioideae

Limonioideae have a more complex taxonomic history than Plumbaginoideae, with many of the currently described genera originally assigned to the former genus *Statice. *Several new, small genera have been segregated primarily from the two most species‐rich genera (*Limonium *and *Acantholimon*). Here, we sampled 19 out of 25 genera for Limonioideae (Table 1), expanding on previous molecular phylogenetic analyses (e.g., Lledó, Crespo, et al., 2005; Malekmohammadi et al., 2017; Moharrek et al., 2017) and further clarifying intergeneric boundaries and relationships in the subfamily. In our phylogeny (Figure 1), *Aegialitis *(Aegialitideae) is sister to Limonieae, confirming the taxonomic subdivision of Limonioideae and previous phylogenetic results (Lledó et al., 2001; Lledó, Crespo, et al., 2005). Baker (1948) proposed that *Aegialitis *represents an isolated, early divergent clade of Limonioideae, because it is characterized by morphological and chemical features typical of the subfamily (Boissier, 1848; Hanson et al., 1994; Harbone, 1967; Maury, 1886), but anatomical features intermediate between the two subfamilies (Maury, 1886) and breeding system similar to Plumbaginoideae (*Plumbago‐*type pollen and monomorphic stigma). In addition, *Aegialitis *is the only genus of Limonioideae with a fully tropical distribution, similar to the great majority of Plumbaginoideae. The placement of *Aegialitis *in our phylogeny as sister to the rest of Limonioideae supports Baker's (1948) hypothesis described above.

In tribe Limonieae, our topology corroborates previous phylogenies (e.g., Lledó, Crespo, et al., 2005; Moharrek et al., 2017) in supporting the sister relationships of *Armeria *with *Psylliostachys*, *Ceratolimon *with *Limoniastrum*, and *Goniolimon *with a clade comprising *Acantholimon *and related genera (Figure 1). Using nine species of *Armeria *and two species of *Psylliostachys, *we confirmed the reciprocal monophyly of these two sister genera (see also Lledó, Crespo, et al., 2005; Moharrek et al., 2014; Moharrek et al., 2017). *Armeria *and *Psylliostachys *share a unique morphological characteristic of the calyx (i.e., the rib‐like tissue of the calyx limb is not present along the calyx tube as it fuses at the limb base; Lledó et al., 2001), but the former comprises perennial herbs with a primarily Western Mediterranean distribution, while the latter consists of annual herbs with an Irano‐Turanian distribution. Our results also agree with Lledó, Crespo, et al. (2005) findings in supporting the sister relationship of *Armeria‐Psylliostachys *clade with *Myriolimon*, *Bakerolimon, *and *Saharanthus*. However, while our dataset also placed the monospecific *Muellerolimon* in a well‐supported clade with *Myriolimon*, *Bakerolimon,* and *Saharanthus, *Lledó, Crespo, et al.'s (2005) phylogeny placed it within a well‐supported clade comprising two species of *Goniolimon*. Our results are also corroborated by Malekmohammadi et al.’s (2017) phylogenetic study. In the latter study, even though *Goniolimon *was not sampled, *Muellerolimon *was sister to *Bakerolimon *and *Myriolimon *in a clade sister to *Psylliostachys *(similar to our results) and distantly related to *Acantholimon‐Popoviolimon*, a clade representing the closest relative of *Goniolimon*. These findings suggest that the accession of *Muellerolimon *used by Lledó, Crespo, et al. (2005) might have been misidentified and/or the sequences mislabeled, incorrectly placing it in the *Goniolimon *clade. The relatively close phylogenetic relationship of *Muellerolimon *and *Bakerolimon* (specifically *Bakerolimon *is sister to *Muellerolimon *and *Saharanthus *clade; Figure 1) is consistent with Baker's (1953a) observations that these genera share distinctive pollen morphology and a shrubby habit with articulate, almost leafless (vestigial or absent leaves) stems; the same stem morphology is also present in *Myriolimon *(Lledó et al., 2003; Lledó, Erben, & Crespo, 2005). Taking into account the morphological features and distribution of *Bakerolimon *(in Chile and Peru) and *Muellerolimon *(in Western Australia), Baker (1953b) hypothesized that these genera are possibly divergent lineages (“remnants”) of an ancient stock of Limonioideae that colonized Western Australia from South America, or vice versa. Unlike *Bakerolimon *and *Muellerolimon, Saharanthus *and *Myriolimon*, also shrubby, occur in the Northern Hemisphere (northwestern Africa and Mediterranean, respectively).

The sister relationship of *Ceratolimon *and *Limoniastrum *and their reciprocal monophyly was originally presented by Lledó et al. (2000) and is confirmed in the current study (clade III; Figure 1). The morphological feature linking these two genera is the adnation of stamen filaments to the corolla up to its tube apex, a feature absent from all other Plumbaginaceae genera (Lledó et al., 2000). *Ceratolimon *species have rosulate leaves, spikelets with an entire to multifid outer bract, and a longer horned inner bract (middle bract absent), whereas *Limoniastrum *species have alternate leaves and spikelets with three smooth bracts (Crespo & Lledó, 2000; Lledó et al., 2000). *Limoniastrum *is distributed in coastal areas of the Mediterranean region (*L. monopetalum *(L.) Boiss.) and subdesert areas of northern Africa (*L. guyonianum *Durieu ex Boiss.), while *Ceratolimon *displays a disjunct distribution: *C. migiurtinum *(Chiov.) M.B.Crespo & M.D.Lledó in Somalia, Yemen and Saudi Arabia (Sudano‐Zambezian region) and its sister species *C. weygandiorum *(Maire & Wilczek) M.B.Crespo & M.D.Lledó and *C. feei *(Girard) M.B.Crespo & M.D.Lledó in Algeria, Morocco, Sahara and Mauritania (Saharan province, Saharo‐Arabian region; Crespo & Lledó, 2000).

In our study, the monospecific genus *Ikonnikovia* is embedded within *Goniolimon* in a well‐supported clade (Figure 1). This result is not completely unexpected since *Ikonnikovia kaufmanniana *(Regel) Lincz. was previously assigned to *Goniolimon *(*G. kaufmannianus* (Regel) Voss.) and was later segregated by Linczevski (1952) on the basis of morphological characteristics, such as style and ovary morphology (i.e., styles verrucose in the lower part and a narrowly linear cylindrical ovary very gradually transiting into the styles; Linczevski, 1952). However, some features link these two genera, and the combination of these features is diagnostic for Plumbaginaceae (i.e., distinguish *Goniolimon *and *Ikonnikovia* from the rest of Plumbaginaceae), namely styles not fused from the base (i.e., free) and non‐glabrous in the lower part (papillose or hairy), and capitate stigmata (Boissier, 1848; Siebert & Voss, 1896). *Goniolimon, *comprising 20 species, has a wide distribution from Italy to Mongolia, whereas *Ikonnikovia *is restricted to Central Asia (Kubitzki, 1993; Linczevski, 1952; Hassler, 2018). According to our phylogenetic results and the shared morphological characters between *Ikonnikovia *and *Goniolimon*, the status of *Ikonnikovia *as a separate genus cannot be further accepted.

The Irano‐Turanian genera *Acantholimon*, *Vassilczenkoa*, *Cephalorhizum, Popoviolimon, Dictyolimon, *and *Bukiniczia *form a well‐supported clade sister to the *Goniolimon *clade (clade II; Figure 1), confirming previous findings (Moharrek et al., 2017). The lack of monophyly for *Acantholimon *and the presence of two separate clades comprising *Acantholimon *species were presented by Moharrek et al. (2017), who sampled 121 *Acantholimon *species and two molecular markers (*trnY‐trnT *spacer and ITS region), and are in agreement with our results. The well‐supported sister relationship between one of the two *Acantholimon *lineages with the *Dictyolimon*‐*Bukiniczia *clade in our study (Figure 1) match closely that of Moharrek et al.’s (2017; see “Clade B”), which additionally included the monospecific genus *Gladiolimon *(not sampled here) within the *Acantholimon *lineage. A difference between our and Moharrek et al.’s (2017) phylogeny is the placement of the monospecific *Vassilczenkoa.* Here, *Vassilczenkoa *is sister to all other Irano‐Turanian genera of this clade (Figure 1), whereas in Moharrek et al.’s (2017) phylogeny, *Vassilczenkoa* with *Chaetolimon *(not sampled here) are sister to *Cephalorhizum*–*Popoviolimon–Bamiania*–*Acantholimon *clade (see “Clade A”; Moharrek et al., 2017). However, in both studies the sister relationship of *Vassilczenkoa* (or *Vassilczenkoa*‐*Chaetolimon*; Moharrek et al., 2017) with related genera receives mostly moderate to low support values, so the relationships of this genus remain unclear. A wide circumscription of *Acantholimon *has been already proposed (Moharrek et al., 2017), in which *Acantholimon s.s. *with all the aforementioned related genera constitute *Acantholimon s.l.*, and within it, the *Dictyolimon‐Bukiniczia*, *Cephalorhizum–Popoviolimon–Bamiania* and *Vassilczenkoa–Chaetolimon *clades are suggested to constitute distinct sections. The species‐rich *Acantholimon, *although widely distributed in Eurasia (from south‐eastern Europe to western China), has its center of diversity in the Irano‐Turanian region, where its closely related oligospecific genera are endemic (Kubitzki, 1993; Hassler, 2018).

*Limonium *forms a well‐supported monophyletic group, yet its sister group remains unresolved (clade I; Figure 1). The only genera of Limonioideae not yet included in any molecular phylogenetic analyses are the Irano‐Turanian *Ghaznianthus, Limoniopsis,* and *Neogontscharovia, *which comprise one, two, and three species, respectively. New insights into the circumscription and relationships within Limonioideae were provided by the current study with complements by the recent study of Moharrek et al. (2017; for *Acantholimon s.l.*), thus improving substantially our understanding of generic integrity and relationships within the tribe.

### 4.3 Genus ***Limonium***

In our phylogeny, the broad sampling for *Limonium *allows us to evaluate the subgeneric, sectional, and subsectional classifications previously proposed for the genus (see Table 2), and suggest revisions aimed at improving, updating, and clarifying infrageneric circumscriptions. However, we acknowledge the existence of limitations, such as low support values, lack of diagnostic morphological traits, and incomplete species sampling that sometimes hinder our systematic conclusions (e.g., “Mediterranean lineage”). To address sampling concerns in *Limonium*, we performed an exhaustive review of the taxonomic literature and used the available morphological, biogeographic, and cytological information to assign the ca. 400 species of *Limonium* that were not sampled in our molecular phylogeny to the resulting clades and their corresponding taxonomic units (see Figures 2 and 3). The results of the mentioned review are compiled in Table S3, which covers the ca. 600 named species of *Limonium*. This effort resulted in the assignment of almost all (>99%) unsampled *Limonium *species to clades supported in our molecular phylogeny and the corresponding subgenera, sections, and subsections (see Table S3). Most of the *Limonium* species were assigned to the large “Mediterranean lineage” (Figures 2, 3 and Table S3), for which further studies are needed to improve its sectional circumscriptions (see below). Below we discuss the taxonomic implications of our phylogenetic results providing additional information on geographic distributions and morphological characteristics of each group.

#### 4.3.1 Clade A—*Limonium* subg. *Pteroclados s.l.*

##### *Limonium* sect. *Pteroclados* and *L. anthericoides*

*Limonium *sect. *Pteroclados *forms a highly supported monophyletic group (Figure 2), as also found by Lledó, Crespo, et al. (2005) and Malekmohammadi et al. (2017). Here, we sampled all 21 species of *L. *sect. *Pteroclados* (Table 2) and show for the first time that the taxonomic subdivision of this section into *L. *sect. *Pteroclados *subsect. *Odontolepideae *and subsect. *Nobiles* is sound and well supported by molecular phylogenetic analyses (Figure 2). In the morpho‐anatomical study of Karis (2004) on 18 species of this section, *L. *subsect. *Odontolepideae *and *L. *subsect. *Nobiles* were monophyletic, though they received only low (50%) and moderate (73%) support values, respectively. Former phylogenetic studies on *Limonium *sampled only few species for *L. *sect. *Pteroclados *(nine species: Lledó, Crespo, et al., 2005; eight species: Malekmohammadi et al., 2017) and did not confirm the subsectional division. Lledó, Karis, Crespo, Fay, and Chase (2011) recovered the monophyly of the two subsections using the same data as in Lledó, Crespo, et al. (2005) but newly generated sequences for *L. spectabile *(Svent.) G.Kunkel & Sunding*,* yet no support values were provided in that phylogeny.

*Limonium *sect. *Pteroclados s*ubsect. *Odontolepideae*, characterized by cuspidate inner bracts and usually conspicuously winged stems (Karis, 2004), is distributed mostly in the Mediterranean region: *L. beaumierianum *(Coss. ex Maire) Maire*, L. bonduellei *(T. Lestib.) Kuntze, and *L. mouretii* (Pit.) Maire are endemic to North Africa, and *L. lobatum *(L.f.) Chaz. and *L*. *sinuatum *(L.) Mill. have a wider distribution from Macaronesia to SW Asia (Hassler, 2018). *Limonium *sect. *Pteroclados s*ubsect. *Nobiles*, characterized by truncate inner bracts and more inconspicuously winged stems than *L. *subsect. *Odontolepideae *(Karis, 2004), consists exclusively of Canarian endemics. Its well‐supported monophyly in our phylogeny postulates a single colonization event of the Canaries followed by in situ diversification. However, the placement of other Canarian endemics, as distant from the *Nobiles *clade and in separate clades of our phylogeny (see *L. *sect. *Ctenostachys*, *L. *sect. *Limoniodendron, *and “Mediterranean lineage”), suggests that *Limonium *colonized the Canarian Islands via multiple (at least four) long‐distance dispersal events (see also Caujapé‐Castells et al., 2017). Our results complement previous morphological, anatomical, chemical and phylogenetic studies on *L. *sect. *Pteroclados *(Bokhari, 1970, 1972; Hanson et al., 1994; Karis, 2004; Lledó et al., 2011; Rao & Das, 1981) providing solid support for its recognition including two well‐defined subsections.

*Limonium *sect. *Pteroclados *is sister to *L. anthericoides *in our phylogeny (Figure 2). This is a novel sister relationship, as in the absence of *L. anthericoides *in previous studies, the section was sister to all other *Limonium *species (e.g., Lledó, Crespo, et al., 2005; Lledó et al., 2011; Akhani et al., 2013; Malekmohammadi et al., 2017). *Limonium anthericoides *is endemic to the coasts of the Western Cape in South Africa and has a peculiar morphology that distinguishes it from the rest of South African species and all other *Limonium*, namely slender fragile branches, aristate calyx ribs extending over and being longer than the calyx limb and very lax inflorescences (Dyer, 1963; Schlechter, 1898). The overall morphology of this species differs substantially from that of its sister *L. *sect. *Pteroclados,* precluding its inclusion in it (see also Taxonomic Proposals). There are only few morphological similarities between *L. anthericoides *and *L. *sect. *Pteroclados*, namely fruits with circumscissile dehiscence (yet, this feature is also found in *L. *sect. *Ctenostachys *and *L. *sect. *Jovibarba*) and inconspicuous calyx with aristate ribs, which occurs in *L. mouretii *of *L. *sect. *Pteroclados *subsect. *Odontolepideae* (Boissier, 1848; Dyer, 1963; Karis, 2004; K. Koutroumpa pers. obs.). Morphological data together with phylogenetic findings suggest the placement of *L. anthericoides *into a separate, new section for *Limonium *(see Taxonomic proposals) sister to *L. *sect. *Pteroclados*. In addition, our results challenge the subgeneric division of *Limonium* proposed by Lledó, Crespo, et al. (2005) and followed by later authors (e.g., Akhani et al., 2013; Malekmohammadi et al., 2017), and postulate the extension of the limits of *L. *subg. *Pteroclados,* previously matching *L. *sect. *Pteroclados*, to include *L. anthericoides *into the newly circumscribed *Limonium *subg. *Pteroclados s.l.*

#### 4.3.2 Clade B—*Limonium* subg. ***Limonium***

##### *Limonium* sect. *Limoniodendron*

Sventenius (1960) described the monotypic *L. *sect. *Limoniodendron* to accommodate *L. dendroides, *which is endemic to La Gomera (Canary Islands), and has unique morphology within the genus, namely arborescent habit, woody stems up to 3 m and salt glands on the spikelets instead of the leaves (Sventenius, 1960). Its phylogenetic placement as an isolated lineage sister to all other *Limonium *species in clade B (Figure 2) confirms previous results (Lledó, Crespo, et al., 2005) and is in agreement with its morphological distinctiveness. Thus, *Limonium *sect. *Limoniodendron *is accepted as a separate section within *Limonium.*

##### *Limonium* sect. *Sarcophyllum* and *L.* sect. *Iranolimon*

*Limonium *sect. *Sarcophyllum* was originally a subsection of *L. *sect. *Limonium *(under *Statice*; Boissier, 1848) and was subsequently elevated to sectional rank by Linczevski (1952). It is characterized by subshrubby habit, long, leafy, woody stems with glaucous, fleshy leaves. The polyphyly of *L. *sect. *Sarcophyllum* has been supported in previous phylogenetic studies (Akhani et al., 2013; Lledó, Crespo, et al., 2005; Malekmohammadi et al., 2017) and is confirmed here (Figure 2). Lledó, Crespo, et al. (2005) recovered three different lineages for the five sampled species: *L. stocksii, L. somalorum, *and *L. axillare *were placed together, while *L. cylindrifolium *and *L. carnosum* were placed in two different clades. Our results, although contradicting those of Lledó, Crespo, et al. (2005) by identifying two instead of three different lineages for the species of *L. *sect. *Sarcophyllum *(Figure 2)*,* are similar to those presented by Akhani et al. (2013) and Malekmohammadi et al. (2017). The phylogenetic placement of *L. cylindrifolium *as sister to *L. biflorum* in a clade that includes Mediterranean species (Lledó, Crespo, et al., 2005) is not confirmed by the current study, and instead, *L. cylindrifolium *is placed with other species of *L. *sect. *Sarcophyllum *(Figure 2). The *rbcL* sequence used by Lledó, Crespo, et al. (2005) for *L. biflorum *produced an extraordinarily long branch that could bias phylogenetic inference and result in a doubtful sister relationship with *L. cylindrifolium*. Indeed, we confirmed the aforementioned bias in our preliminary analyses; hence, we replaced Lledó, Crespo, et al. (2005) *rbcL *sequence of *L. biflorum* with a recently generated one (Galmés et al., 2014), which allowed us to resolve the placement of *L. cylindrifolium*.

Malekmohammadi et al. (2017) segregated one of the two clades comprising species of *L. *sect. *Sarcophyllum *and created *L. *sect. *Iranolimon* on the basis of phylogenetic and morpho‐anatomical data (e.g., leaves with one main nerve and C, S and V‐shaped sclereids; Akhani et al., 2013; Malekmohammadi et al., 2017)*. *Our topology confirms the circumscription of this newly generated section, as five out of nine species ascribed to it form a highly supported clade closely related to *L. *sect. *Ciricinaria, L. *sect. *Sphaerostachys *and *L. *sect. *Limonium *subsect. *Genuinae* (Figure 2). Species of *L. *sect. *Iranolimon *are mostly distributed in the Irano‐Turanian region, whereas the remaining species of *L. *sect. *Sarcophyllum *are mostly found in the Sudano‐Zambezian region*.* In our phylogeny, the Sudano‐Zambezian/Saharo‐Arabian *L. axillare* and Sudano‐Zambezian *L. stocksii, L. somalorum, L. sokotranum, L. paulayanum, L. sarcophyllum, L. milleri, *and *L. cylindrifolium* form a well‐supported clade (Figure 2) and are characterized by subshrubby, sometimes cushion‐like habit, woody caudex, leaves fleshy with three main vascular bundles in cross section and relatively dense inflorescences (Bokhari, 1973; Akhani et al., 2013; K. Koutroumpa pers. obs.). Our molecular tree in combination with morpho‐anatomical features supports a change in the circumscription of *Limonium *sect. *Sarcophyllum* (sensu Linczevski, 1952) to include only species of the Sudano‐Zambezian/Saharo‐Arabian clade.

##### *Limonium *sect.* Nephrophyllum *and “*L. bellidifolium* complex”

*Limonium *sect. *Nephrophyllum *was originally designated by Rechinger in *Flora Iranica *(Rechinger & Schiman‐Czeika, 1974) and is characterized by round reniform amplexicaule stem leaves, not persistent (caducous) rosette leaves, and obconical calyces with narrow limbs. As originally circumscribed, this section includes *L. otolepis *(Schrenk) Kuntze*, L. perfoliatum *(Kar. ex Boiss.) Kuntze, and *L. reniforme* (Girard) Lincz., which are endemic to the Irano‐Turanian region (Akhani et al., 2013; Rechinger & Schiman‐Czeika, 1974). The three species of this section do not form a monophyletic group in our molecular phylogeny. *Limonium *sect. *Nephrophyllum *together with species of *L. *sect. *Limonium *subsect. *Hyalolepideae *(i.e., *L. bellidifolium *(Gouan) Dumort., *L. iconicum *(Boiss. & Heldr.) Kuntze; part of the “*L. bellidifolium *complex”) form a strongly supported clade (Figure 2) in agreement with Malekmohammadi et al.’s (2017) results. Morphological similarities linking the species of this clade include rosette leaves that dry up before the end of flowering, amplexicaule or semi‐amplexicaule stem leaves (sometimes absent), few or several sterile branches (rarely absent), numerous small spikelets with broadly membranous (hyaline) outer and inner bracts (Akhani et al., 2013; Boissier, 1848; Erdal, 2015; Malekmohammadi et al., 2017); this is a combination of diagnostic features from both *L. *sect. *Nephrophyllum *and *L. *sect. *Limonium *subsect. *Hyalolepideae*. Apart from *L. bellidifolium, *which has a wide distribution from the Irano‐Turanian to the Mediterranean and northern Europe (Pignatti, 1972; Hassler, 2018), the rest of the species included in this clade are strictly Irano‐Turanian elements. Considering both morphological and molecular evidence, and in agreement with Malekmohammadi et al. (2017), a wider circumscription of *Limonium *sect. *Nephrophyllum *(i.e., *L. *sect. *Nephrophyllum s.l.*) is proposed to accommodate all species in this clade.

##### *Limonium* sect. *Plathymenium* and *L.* sect. *Siphonocalyx*

*Limonium *sect. *Plathymenium *is sister to *L. *sect. *Nephrophyllum s.l.* and forms a well‐supported clade together with *L. *sect. *Siphonocalyx *(Figure 2), similar to previous findings (Malekmohammadi et al., 2017). *Limonium *sect. *Plathymenium *is characterized by caudex bearing hyaline to brown or black scales, cylindrical, angled or very narrowly winged branches, capitate inflorescences, funnel‐form calyces with broad limbs, strongly oblique at base (e.g., Boissier, 1848; Linczevski, 1952). Though the monophyly of this section is well‐established (Lledó, Crespo, et al., 2005; Malekmohammadi et al., 2017), the subdivision into *L. *sect. *Plathymenium *subsect. *Chrysanthae *and subsect. *Rhodanthae *proposed by Boissier (1848) on the basis of corolla color (i.e., yellow and reddish, respectively) is not confirmed by our topology (Figure 2). *Limonium *sect. *Siphonocalyx*, represented here by *L. sogdianum, *consists of species occurring in Central Asia on gypsum and saline soils and is morphologically diagnosed by the tubular calyces with straight or slightly reflexed limbs (Linczevski, 1952). *Limonium *sect. *Plathymenium *and *L. *sect. *Siphonocalyx *are well‐defined groups accepted as distinct sections of *Limonium*, whereas the subdivision of the former section into *L. *subsect. *Chrysanthae *and subsect. *Rhodanthae *is not supported by our results.

##### *Limonium* sect. *Jovibarba*, *L. sect*. ***Ctenostachys***, and *L. lobinii*

*Limonium *sect. *Jovibarba* is a monotypic section for *L. jovibarba *(Webb) Kuntze*,* an endemic species of Cape Verde (Boissier, 1848). *Limonium jovibarba *is a subshrub with branched woody caudex, funnel‐form calyces with fringed margins divided into five tooth‐like lobes, and circumscissile fruits (Lobin, Leyens, Kilian, Erben, & Lewejohann, 1995). It is sister to the clade formed by *L. lobinii* and *L. *sect. *Ctenostachys*, with which it constitute a highly supported lineage (Figure 2). The sister relationship between *L. *sect. *Jovibarba *and *L. *sect. *Ctenostachys *was originally presented in Lledó, Crespo, et al. (2005) phylogeny based on the sampling of only two species (*L. jovibarba* and *L. pectinatum *(Ait.) Kuntze). *Limonium jovibarba*, *L. lobinii, *and *L. sundingii *Leyens, Lobin, N.Kilian & Erben (not sampled in this study) are three Cape Verdean endemics with similar ecology and habit (Lobin et al., 1995), all being subshrubs restricted to steep, moist cliffs growing at 50–800 m. *Limonium lobinii *differs from the other two species by the conspicuously winged stems and the more compact spikes (Lobin et al., 1995), traits both found in *L. *sect. *Ctenostachys*. Its morphological affinities with both *L. *sect. *Jovibarba *and *L. *sect. *Ctenostachys *corroborate its placement in our phylogeny, where *L. lobinii* is sister to *L. *sect. *Ctenostachys*, with which it forms a moderately to poorly supported clade that is sister to *L. jovibarba* (Figure 2).

*Limonium *sect. *Ctenostachys *consists of perennial herbs with crispate‐winged or angled stems, rarely round, articulate branching, terminal inflorescence forming secund, mostly compact, spreading‐scorpioid spikes, and funnel‐form, often colored and shortly lobed calyces (Boissier, 1848). The species are distributed in Macaronesia and Morocco: *Limonium brunneri *(Webb ex Boiss.) Kuntze and *L. braunii *(Bolle) A.Chev. are sister species endemic to Cape Verde, *L. papillatum* (Webb & Berthel.) Kuntze and the varieties of *L. pectinatum* are endemic to the Canaries and Savage islands, and the sister *L. mucronatum *(L.f.) Chaz. and *L. fallax *(Coss. ex Wangerin) Maire are endemic to SW Morocco. Here, we show that species of *L. *sect. *Ctenostachys* constitute a well‐supported monophyletic group (Figure 2)*.*

An interesting biogeographic pattern is observed in the clade comprising *Limonium *sect. *Jovibarba, L. *sect. *Ctenostachys, *and *L. lobinii*: the Cape Verdean endemics do not form a monophyletic group, suggesting multiple colonization events (at least two) of the archipelago. The divergent ecologies of Cape Verdean species (with *L. braunii* and *L. brunneri* occurring in arid and semi‐arid coastal habitats and *L. jovibarba*, *L. lobinii*, and *L. sundingii* mainly restricted to humid, mountainous abrupt cliffs; Lobin et al., 1995; Romeiras, Monteiro, Duarte, Schaefer, & Carine, 2015) seem to agree with the hypothesis of multiple colonizations of the archipelago.

To summarize, morphological and molecular data support the recognition of *Limonium *sect. *Ctenostachys*, but the circumscription of *L. *sect. *Jovibarba *needs further clarification (i.e., whether or not to enclose *L. lobinii* and *L. sundingii*).

##### *Limonium* sect. *Circinaria*

This section is endemic to South Africa and characterized by very large flowers with circinate styles and capitate stigmata, a combination of traits unique in the genus (Baker, 1953a; Boissier, 1848). Linczevski (1979) segregated *L. *sect. *Circinaria *from *Limonium *and created genus *Afrolimon *to include seven species. Later studies rejected the generic status of *Afrolimon *since its species were confidently placed within *Limonium *in molecular phylogenies (Lledó, Crespo, et al., 2005; Malekmohammadi et al., 2017). In our study, three species of this section form a well‐supported clade within *Limonium*. This clade, in turn, is included in a well‐supported polytomy with a clade of *L. *sect. *Iranolimon* and a clade formed by *L. *sect. *Sphaerostachys *and *L. *sect. *Limonium *subsect. *Genuinae *(Figure 2), confirming the cpDNA topologies of Lledó, Crespo, et al. (2005) and Malekmohammadi et al. (2017). However, the latter study, using a single species of *L. *sect. *Circinaria *(*L. peregrinum* (P.J. Bergius) R.A. Dyer), recovered a different topology in the ITS tree (i.e., *L. peregrinum* sister to a clade formed by *L. *sect. *Nephlophyllum s.l., L. *sect. *Plathymenium *and *L. *sect. *Siphonocalyx*). In the absence of ITS sequences for this section in the current study, we are unable to confirm the latter finding. Nevertheless, both molecular and morphological evidence support the recognition of this section within *Limonium*.

##### *Limonium* sect. *Sphaerostachys* and *L. *sect. *Limonium* subsect. *Genuinae*

*Limonium *sect. *Limonium *subsect. *Genuinae *is distinguished by its large broad leaves with pinnate venation, tall stems with few or no sterile branches, large inflorescences, and calyces with short denticulate limbs bearing up to 10 lobes, with short lobes placed between larger lobes (Boissier, 1848). This subsection occupies a broad geographic range, occurring in both the Old (Irano‐Turanian, Mediterranean, Euro‐Siberian, and Macaronesian regions) and New World (North and South America), with species growing often in salt marshes and saline steppes. In this study, representatives of *L. *subsect. *Genuinae *form a monophyletic group together with *L. latifolium *(Figure 2)*, *which was originally assigned to *L. *subsect. *Hyalolepideae *(Boissier, 1848), but later transferred to *L. *subsect. *Genuinae* on the basis of morpho‐anatomical similarities (Bokhari, 1973)*. Limonium *sect. *Limonium *as circumscribed by Boissier (1848) refers to a non‐monophyletic assemblage based on current (Figures 2 and 3) and previous findings (e.g., Palacios et al., 2000; Lledó, Crespo, et al., 2005; Malekmohammadi et al., 2017). Several species have been subsequently segregated from *L. *sect. *Limonium *and transferred to *L. *sect. *Sarcophyllum, L. *sect.* Iranolimon, *and *L. *sect. *Nephrophyllum*, yet the section remains polyphyletic and morphologically very variable. The broad sampling of *L. *sect. *Limonium *in this study, with numerous representatives from all subsections, provides us with a solid framework to propose a new taxonomic circumscription for this section. Thus, based on molecular and morphological evidence, we propose a circumscription for *L. *sect. *Limonium* strictly matching the composition of *L. *sect. *Limonium *subsect. *Genuinae, *which includes *L. vulgare *Mill., the type species of *Limonium*. This implies that, apart from species formerly of *L. *sect. *Limonium* subsect. *Genuinae, *here newly comprising the entire *L. *sect. *Limonium*, the remaining species previously assigned to the same section should be placed into different sections.

*Limonium *sect. *Limonium *subsect. *Genuinae *is sister to *L. *sect. *Sphaeorostachys *(Figure 2; see also Malekmohammadi et al., 2017). The latter section, constituting of three species distributed in Turkey (Inner Anatolia) and Syria, is characterized by stems without sterile branches, leaves with undulate‐hyaline margin, inflorescences of globose or congested spikes and flowers with densely pilose, obconical calyces with ribs terminating well below the margin (Boissier, 1848; Bokhari, 1972; Bokhari & Edmondson, 1982). According to Bokhari (1973), *L. *sect. *Sphaerostachys *and *L. *sect. *Limonium *subsect. *Genuinae *share a unique anatomical trait, namely the two rings of large vascular bundles in the unbranched part of the stem and the primary branches. Therefore, both molecular and morpho‐anatomical data support the recognition of a well‐defined *Limonium *sect. *Sphaerostachys* sister to the re‐circumscribed *L. *sect. *Limonium*.

##### “Mediterranean lineage”—*Limonium* sect. *Polyarthrion*, *L.* sect. *Schizhymenium*, *L.* sect. *Siphonantha*, and *L.* sect. *Limonium* subsect. *Densiflorae*, subsect. *Dissitiflorae*, subsect. *Hyalolepideae*, subsect. *Pruinosae* and subsect. *Steirocladae*

The large “Mediterranean lineage” (Figure 2, clade B3; Figure 3) is well‐supported and sister to clade B2, which comprises species mostly occurring outside the Mediterranean region (Figure 2). The “Mediterranean lineage” comprises species assigned to *L. *sect. *Siphonantha*, *L. *sect. *Polyarthrion*, *L. *sect. *Schizhymenium, *and *L. *sect. *Limonium* sensu Boissier (1848), but also many species that are not assigned to any section of *Limonium* (Figure 3). *Limonium *sect. *Siphonantha *(originally described as monospecific by Boissier, 1848, with the only species *L. tubiflorum *(Del.) Kuntze) is characterized by densely branched stems, scorpioid‐corymbiform inflorescences formed by flowers bearing large corollas with apically rounded corolla lobes, and membranous calyx limbs deeply divided into five lobes ending with an awn (Boissier, 1848; Boulos, 2000). This morphologically distinct section occurs in North Africa and is closely related to *L. *sect. *Polyarthrion *and representatives of *L. *sect. *Limonium *in our phylogenetic analyses (i.e., ITS tree: *L. *subsect. *Hyalolepideae/Pruinosae*, cpDNA tree: *L. *subsect. *Hyalolepideae/Pruinosae *and subsect. *Dissitiflorae*; Figure 3). *Limonium *sect. *Polyarthrion, *represented by *L. caesium *(Girard) Kuntze and *L. insigne *(Coss.) Kuntze, endemic to Spain, comprises species with numerous sterile, articulate branches in the lower part of stem and large spikelets with pink corollas. The monophyly of *L. *sect. *Polyarthrion *is strongly supported in the ITS tree, which places it as sister to the clade formed by *L. *sect. *Siphonantha *and *L. *sect. *Limonium *subsect. *Hyalolepideae/Pruinosae*, while in the cpDNA tree, the two species representing this section are closely related but their sister relationship is unresolved (Figure 3). *Limonium *sect. *Schizhymenium*, represented by the widespread Mediterranean species *L. echioides *(L.) Mill.*, *encompasses annual herbs bearing characteristic subtubular calyces with limbs lacerating in maturity and ribs forming hooked barbs (Bokhari, 1972).

*Limonium *sect. *Limonium* subsect. *Hyalolepideae* sensu Boissier (1848) is non‐monophyletic (Figures 2 and 3) and its diagnostic features (i.e., sterile, multi‐divided branches in the lower part of the plant and small spikelets with broadly or entirely membranous/hyaline bracts) are inconsistent with the current phylogenetic results. In the “Mediterranean lineage,” there are four representatives of this subsection (Figure 3). Three of them, *L. tuberculatum *(Boiss.) Kuntze, *L. pruinosum* (L.) Chaz., and *L. asparagoides *(Batt.) Maire, form a strongly supported clade sister to *L. *sect. *Siphonantha* in the ITS tree, similar to the cpDNA tree although with less resolution (Figure 3). These three species comprise *L. *sect. *Limonium *subsect. *Pruinosae *according to Sauvage and Vindt (1952) that followed the classification originally proposed by Battandier (1888). This subsection is characterized by stems and branches covered by calcariferous tubercles with a punctuate depression in the center, numerous sterile branches, one‐flowered spikelets, calyces with membranous limbs, and deciduous leaves; its three representatives occur in North Africa, with *L. tuberculatum *and *L. pruinosum *extending their distributions into Macaronesia and Saharo‐Arabian regions, respectively.

*Limonium *sect. *Limonium* subsect. *Dissitiflorae,* characterized by few or no sterile branches, paniculate, often secund, inflorescences with laxly imbricate or remotely arranged spikelets, and 5‐lobed calyces (Boissier, 1848), is represented by 10 Mediterranean endemics placed in different, mostly unresolved clades (Figure 3). *Limonium *sect. *Limonium* subsect. *Densiflorae,* characterized by few or no sterile branches, distichous panicle inflorescences with many secund branches, distichous spikes, spikelets often densely imbricate, and 5‐lobed calyces (Boissier, 1848), is represented by eight mostly Mediterranean endemics that are intermingled with other species in a largely unresolved clade (Figure 3). Lastly, *Limonium *sect. *Limonium* subsect. *Steirocladae, *characterized by sterile stems, often very branched and articulate, spikelets often forming a corymb, and bracts with narrow, hyaline‐membranous margin (Boissier, 1848), is represented by 10 species that fall in the same, widely unresolved clade (Figure 3). These species are Mediterranean endemics, except for *L. scabrum *(Thunb.) Kuntze and *L. kraussianum *(Buchinger ex Boiss.) Kuntze, which are endemic to South Africa. In our study, South African species do not form a monophyletic group, but are placed in three clades corresponding to *L. anthericoides*, *L. *sect. *Circinaria*, and “Mediterranean lineage” (Figures 2 and 3) postulating at least three different immigration events of *Limonium *into South Africa.

While most species in the “Mediterranean lineage” are Mediterranean endemics, few of them extend further North (European Circumboreal region: e.g., *L. recurvum *C.E.Salmon subsp. *humile *(Girard) Ingr., *L. binervosum *(G.E.Sm.) C.E.Salmon), South (South Africa: see above), East (Saharo‐Arabian region: e.g., *L. pruinosum*), and West (Madeira: *L. lowei* R.Jardim, M.Seq., Capelo, J.C.Costa & Rivas Mart. and Canaries: *L. bollei *(Webb ex Wangerin) Erben*, L. tuberculatum*). The radiation of *Limonium *in the Mediterranean has been attributed to several factors, including apomixis, hybridization, and polyploidization (e.g., Ingrouille, 1984; Palacios et al., 2000). The incongruences detected between well‐supported clades and individual taxa in the chloroplast and nuclear trees corroborate the explanation proposed above. For example, the clade comprised of endemics in the Aegean archipelago that are usually allopolyploids with different combinations of the basic chromosome numbers *x* = 8, 9 (e.g., Artelari, 1989; Brullo & Erben, 2016) show different phylogenetic relationships in the cpDNA versus the nrDNA tree (orange bar, Figure 3), suggesting reticulate evolution. Furthermore, the low resolution, together with the presence of short branches, might indicate a recent diversification for the “Mediterranean lineage.”

According to molecular and morphological evidence, *Limonium *sect. *Polyarthrion, L. *sect. *Siphonantha *and *L. *sect. *Schizhymenium *are accepted in the current study, while *L. *sect. *Limonium *subsect. *Pruinosae *should be raised to the sectional rank, because it forms a monophyletic group with *L. *sect. *Polyarthrion *and *L. *sect. *Siphonantha* and it cannot maintain its previous rank due to the new circumscription of *L. *sect. *Limonium *proposed here (see Taxonomic proposals). The acceptance of these four sections within the “Mediterranean lineage” is an important first step toward the improvement of the circumscription of this taxonomically complex and species‐rich clade (ca. 72% of *Limonium *species are assigned to this clade, excluding species belonging to *L. *sect. *Polyarthrion, L. *sect. *Siphonantha, L. *sect. *Schizhymenium *and *L. *sect. *Pruinosum*; see Table S3). For the remaining species in the “Mediterranean lineage” (i.e., *L. *sect. *Limonium *subsect. *Hyalolepideae p.p., *subsect. *Dissitiflorae, *subsect. *Densiflorae* and subsect. *Steirocladae*, and unclassified species), additional studies aimed at improving phylogenetic resolution, clarifying evolutionary origins for taxa of hybrid origin, and reviewing diagnostic morphological characters are needed in order to propose new taxonomic classifications.

In conclusion, our molecular phylogenetic results together with a revision of morphological diagnostic characters of different genera within Plumbaginaceae and different sections and subsections within *Limonium* allowed us to propose some taxonomic changes (see Taxonomic proposals, below). In addition, the present study laid the foundations for further research on the spatiotemporal evolution of *Limonium *and the drivers of its diversification. Both issues are currently being addressed as part of ongoing studies on *Limonium*, a genus that has speciated intensively in the Mediterranean region and occupies different island systems.

### 4.4 Taxonomic Proposals

***Goniolimon*** Boiss. in DC., Prodr. 12: 632. 1848.—Type: *Goniolimon tataricum *(L.) Boiss. in DC., Prodr. 12: 632. 1848, here selected*.

= *Statice *sect. *Tropidice *Griseb. Spicil. Fl. Rumel. 2: 299. 1846.

= *Ikonnikovia *Lincz. in Kom., Fl. URSS 18: 378, 745. 1952.—Type: *Ikonnikovia kaufmanniana *(Regel) Lincz. in Kom., Fl. URSS 18: 381. t. 19. f. 3. 1952.

* *Goniolimon tataricum *is one of the validly named species in the genus protologue (Boissier, 1848), it has not been segregated from the genus or synonymized and matches the generic description. The lectotype of *Goniolimon tataricum* (designated by Edmonson in Jarvis, 2007:874, leg. “Amman s.n., Herb Linn. No. 395.12 (LINN)”) is hereafter the type of the generic name.

***Limonium*** subg. ***Pteroclados*** (Boiss.) Pignatti ***s.l. ***(**emend.** Koutroumpa)—Type: *Limonium sinuatum *(L.) Mill., Gard. Dict., ed. 8: Limonium no. 6. 1768.

= *Linczevskia* Tzvelev in Takhtajan, Konspekt Fl. Kavkaza 3(2): 283. 2012.—Type: *Linczevskia sinuata* (L.) Tzvelev in Konspekt Fl. Kavkaza 3(2): 283. 2012.

Perennial (rarely annual) herbs or shrubs with leaf rosettes; leaves entire to sinuate‐lobed; stems bearing wings, sometimes absent; inflorescence often rather lax, rarely very lax (i.e., *L. anthericoides*) or sometimes dense; spikelets distichous; calyx infundibuliform, conspicuous with broad limb and ribs below, reaching or slightly above the lobe tips, or rarely obconical, inconspicuous but with ribs extended well above the lobe tips (i.e., *L. mouretii *and *L. anthericoides*); corolla often white, sometimes yellow or light pink; fruit with circumscissile dehiscence.

*Limonium *subg. *Pteroclados s.l. *includes all 21 species of *L. *sect. *Pteroclados *(see Table 2) and *L. anthericoides* of the new *L. *sect. *Tenuiramosum *(see below).

***Limonium*** sect. ***Limonium*** (**emend**. Koutroumpa)—Type: *Limonium vulgare *Mill., Gard. Dict., ed. 8: Limonium no. 1. 1768, typ. cons.

= *Statice *sect. *Limonium* subsect. *Genuinae *Boiss. in DC., Prodr. 12: 643. 1848.

Perennial herbs 15–150 cm tall; leaves large, pinnately veined, forming rossete; sterile branches few or absent (rarely fairly numerous); inflorescence with more or less dense spikes; spikelets small, usually with 1–4 flowers; calyx obconical or very narrowly funnel‐form; calyx limb short, undulate, bearing 5–10 distinct lobes, usually with short lobes placed between larger lobes; corolla bluish‐violet, rarely lilac.

This is a new, more restricted circumscription of *Limonium *sect. *Limonium *that matches closely the composition of *L. *sect. *Limonium *subsect. *Genuinae*. Species previously assigned to *L. *sect. *Limonium *and are not part of *L. *subsect. *Genuinae *should be segregated from this section as currently circumscribed. Species of *L. *sect. *Limonium* have wide (e.g., *L. gmelini *(Willd.) Kuntze, *L. humile *Mill.*,* *L. latifolium, L. meyeri *(Boiss.) Kuntze*, L. vulgare*) or more restricted (e.g., *L. alutaceum* (Stev.) Kuntze,* L. asterotrichum* (Salmon) Salmon, *L. compactum* Erben & Brullo, *L. pagasaeum *Erben & Brullo) distributions in the Old or New World (e.g., *L. brasiliense *(Boiss.) Kuntze, *L. californicum *(Boiss.) A. Heller, *L. guaicuru *(Molina) Kuntze, *L. limbatum *Small).

***Limonium*** sect. ***Nephrophyllum*** Rech.f. ***s.l. ***(**emend**. Koutroumpa) ≡ *Statice *sect. *Limonium *subsect. *Hyalolepideae* Boiss. *p.p.* in DC., Prodr. 12: 659. 1848.—Type: *Limonium reniforme *(Girard) Lincz. in Kom., Fl. URSS 18: 456. 1952.

Perennial herbs; basal leaves forming rossete, spathulate to obovate‐spathulate, rarely oblanceolate, dying before end of flowering, rarely persistent (e.g., *L. myrianthum *(Schrenk) Kuntze); cauline leaves present, amplexicaule or semi‐amplexicaule, sometimes absent; sterile branches few to numerous mostly in lower part, rarely absent; inflorescence paniculate; spikelets small (c. 2–6 mm) with broadly membranous bracts; calyx usually obconical, sometimes funnel‐form, 5‐lobed; calyx ribs terminating bellow margin.

This is an expanded circumscription for *L. *sect. *Neprophyllum *that together with *L. otolepis, L. perfoliatum *and *L. reniforme* newly includes species from *L. bellidifolium *complex (*L. bellidifolium* and *L. iconicum *sampled in the phylogeny, and other relatives: e.g., *L. caspium *(Willd.) Gams, *L. coralloides *(Tausch) Lincz., *L. macrorrhizon *(Ledeb.) Kuntze, *L. myrianthum, L. smithii *Akaydin, *L. tamaricoides *Bokhari) many of them previously assigned to *L. *sect. *Limonium *subsect. *Hyalolepideae *sensu Boissier and are distributed in the Irano‐Turanian area, apart from *L. bellidifolium *that expands toward the Euro‐Siberian and Mediterranean regions.

***Limonium ***sect. ***Pruinosum ***(Batt.) Koutroumpa, **comb. nov.** ≡ *Statice *sect. *Limonium *subsect. *Pruinosae *Battandier in Batt. et Trabut, Fl. Algérie 1: 727. 1888 ≡ *Limonium *sect.* Limonium *subsect. *Pruinosa* (Batt.) Sauvage & Vindt, Fl. Maroc 1: 46, 58. 1952.—Type: *Limonium pruinosum *(L.) Kuntze, in Revis. Gen. Pl. 2: 396. 1891.

***Limonium*** sect. ***Sarcophyllum*** (Boiss.) Lincz. **emend. **Koutroumpa* *≡ *Statice *sect. *Limonium *subsect. *Sarcophyllae* Boiss. *p.p.* in DC., Prodr. 12: 663. 1848.—Type: *Limonium axillare *(Forssk.) Kuntze in Revis. Gen. Pl. 2: 395. 1891.

Shrublets sometimes cushion‐formed; caudex woody; leaves mostly cauline on woody branches, alternate and often spirally arranged, fleshy, oblanceolate to spathulate or cylindrical, sometimes with an auricle at the apex, and with 3 large vascular bundles (i.e., nerves) in cross section; inflorescence relatively dense paniculate, rarely lax; calyx funnel‐form, sometimes obconical.

The newly circumscribed *L. *sect. *Sarcophyllum *includes Sudano‐Zambezian/Saharo‐Arabian species (e.g., *L. cylindrifolium, L. maurocordatae *(Schweinf. & Volk.) Cufod., *L. milleri, L. paulayanum, L. sarcophyllum, L. sokotranum, L. somalorum, L. stocksii*) and it does not include the Irano‐Turanian group of species currently assigned to *L. *sect. *Iranolimon*. ***Limonium*** sect. ***Tenuiramosum ***Koutroumpa **sect. nov.**—Type: *Limonium anthericoides *(Schltr.) R. A. Dyer in Bull. Misc. Inform. Kew 1935: 155. 1932.

Perennial herbs with leaf rosettes; leaves obovate or elliptic‐spathulate; stems erect, flexuous, verrucose, very laxly branched on the upper half with slender fragile branches bearing inflorescences; spikes very laxly arranged; spikelets small usually with 2–4 flowers; calyx pilose, obconical with scarious limb, and 5 main lobes with ribs and 5 short intermediate lobes; aristate calyx ribs, well above the lobes, longer than the limb; corolla white; fruit with circumscissile dehiscence.

This is a newly described monospecific section for *L. anthericoides*, a morphologically isolated species for *Limonium,* endemic to the coastal areas of the Cape in South Africa. ***Limonium*** sect. ***Pteroclados*** subsect. ***Nobiles ***(Boiss.) Koutroumpa, **comb. nov.** ≡ *Statice *sect. *Pteroclados* subsect. *Nobiles *Boiss. in DC., Prodr. 12: 636. 1848—Type: *Limonium arboreum *(Willd.) Erben *et al.* in Fl. Medit. 22: 65. 2012. ***Limonium*** sect. ***Pteroclados*** subsect. ***Odontolepideae ***(Boiss.) Koutroumpa, **comb. nov.** ≡ *Statice *sect. *Pteroclados* subsect. *Odontolepideae *Boiss. in DC., Prodr. 12: 635. 1848—Type: *Limonium sinuatum *(L.) Mill., Gard. Dict., ed. 8: Limonium no. 6. 1768.

= *Linczevskia* Tzvelev in Takhtajan, Konspekt Fl. Kavkaza 3(2): 283. 2012.—Type: *Linczevskia sinuata* (L.) Tzvelev in Konspekt Fl. Kavkaza 3(2): 283. 2012.

### 4.5 Accepted Taxonomic Units of *Limonium* in this study

**Genus *Limonium* Mill**.

*L. *subg. *Pteroclados *(Boiss.) Pignatti *s.l. *(emend. Koutroumpa)

*L. *sect. *Pteroclados *(Boiss.) Bokhari
*L. *sect. *Pteroclados *subsect. *Odontolepideae *(Boiss.) Koutroumpa
*L. *sect. *Pteroclados *subsect. *Nobiles *(Boiss.) Koutroumpa

*L. *sect. *Tenuiramosum* Koutroumpa

*L. *subg. *Limonium*

*L. *sect. *Circinaria *(Boiss.) M. Malekm
*L. *sect. *Ctenostachys *(Boiss.) Sauvage & Vindt
*L. *sect. *Iranolimon* M.Malekm., Akhani & Borsch
*L. *sect. *Jovibarba *(Boiss. sub *Statice*)
*L. *sect. *Limoniodendron* Svent.
*L. *sect. *Limonium *(emend. Koutroumpa)
*L. *sect. *Nephrophyllum *Rech.f. *s.l. *(emend. Koutroumpa)
*L. *sect. *Plathymenium *(Boiss.) Lincz.
*L. *sect. *Polyarthrion *(Boiss.) Sauvage & Vindt
*L. *sect. *Pruinosum *(Batt.) Koutroumpa
*L. *sect. *Sarcophyllum *(Boiss.) Lincz. emend. Koutroumpa
*L. *sect. *Schizhymenium *(Boiss.) Bokhari
*L. *sect. *Siphonantha *(Boiss.) Sauvage & Vindt
*L. *sect. *Siphonocalyx* Lincz.
*L. *sect. *Sphaerostachys *(Boiss.) Bokhari

## CONFLICT OF INTEREST

None declared.

## AUTHORS CONTRIBUTIONS

All authors contributed to critical revisions of the manuscript. K. Koutroumpa, A. Jiménez, J. M. Fernández‐Palacios, and E. Conti conceived the idea and designed the project. K. Koutroumpa, A. Jiménez, F. Celep, M. Doğan, M. M. Romeiras, A. Santos‐Guerra, J. Caujapé‐Castells, M. Moura, and M. Menezes de Sequeira did fieldwork, provided leaf tissue for DNA extraction, and taxonomically identified collected samples. K. Koutroumpa, A. Jiménez, and B. H. Warren performed DNA extractions. K. Koutroumpa performed PCR amplifications, sequencing and data analyses, under the supervision of S. Theodoridis. K. Koutroumpa, B. H. Warren, S. Theodoridis, A. Jimenez, and E. Conti contributed to the interpretation of the data. K. Koutroumpa wrote the main draft used for revision by all co‐authors. All co‐authors approved the final content of the paper.

## DATA ACCESSIBILITY

Newly generated DNA sequences: GenBank accessions MH560967–MH561155 (*trnL‐F*); MH582667–MH582838 (*rbcL*); MH582839–MH583017 (matK); MH582513–MH582666 (ITS). Sequences provided to us by the authors of Lledó, Crespo, et al. (2005) and used in this study are archived at Dryad Digital Repository (https://doi.org/10.5061/dryad.961m4t8). List of species and GenBank accession numbers and codes of all pre‐existing and newly generated sequences used in this study are uploaded as online supporting information (Data S3).
