# Supplementary figures and images for: An expanded molecular phylogeny of Plumbaginaceae, with emphasis on Limonium (sea lavenders): Taxonomic implications and biogeographic considerations
Source: Ecol Evol. 2018 Dec 6;8(24):12397–424. doi: 10.1002/ece3.4553 (PMC6308857; doi:10.1002/ece3.4553)

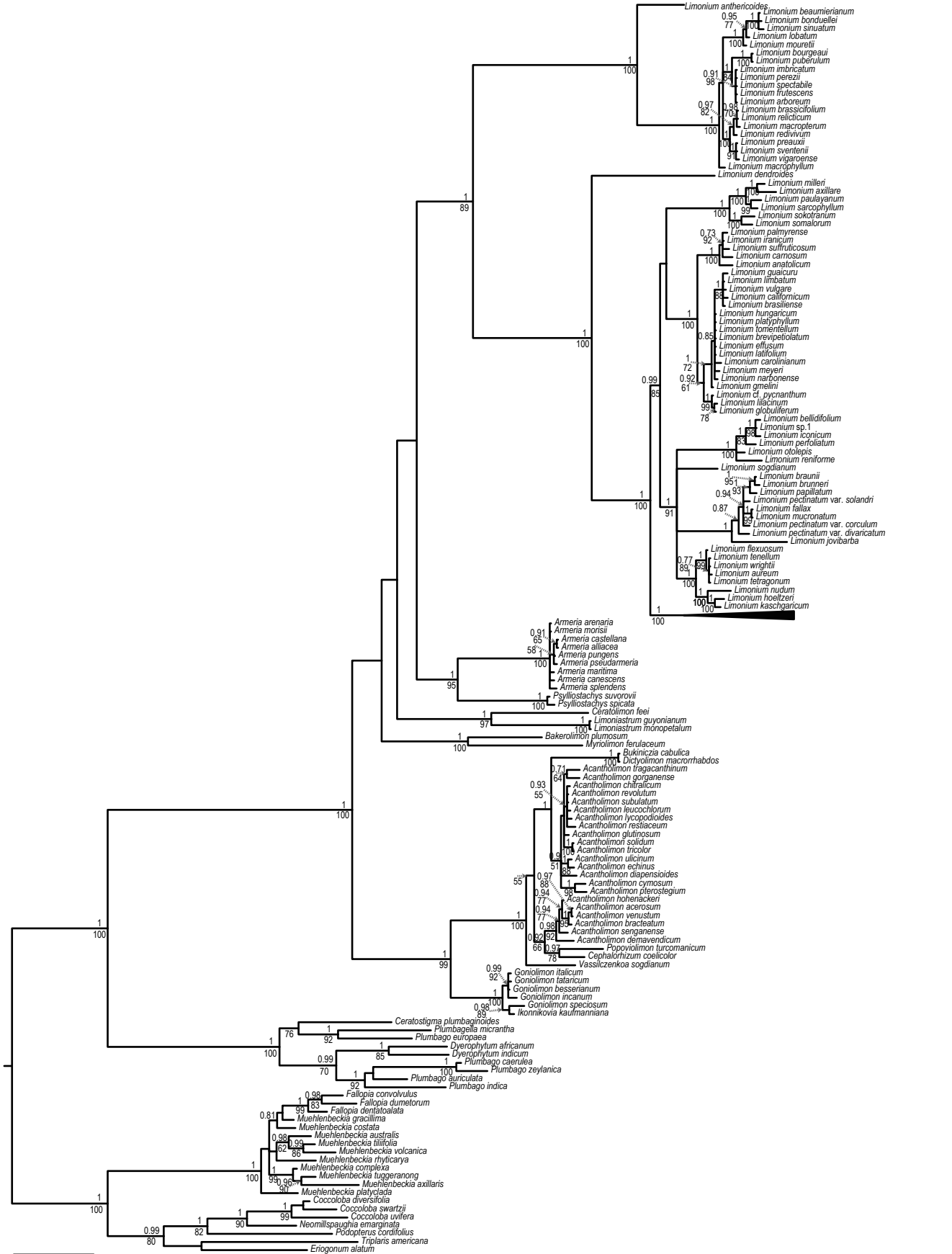

Supplement: Supplementary file 1 [file ECE3-8-12397-s001.pdf]

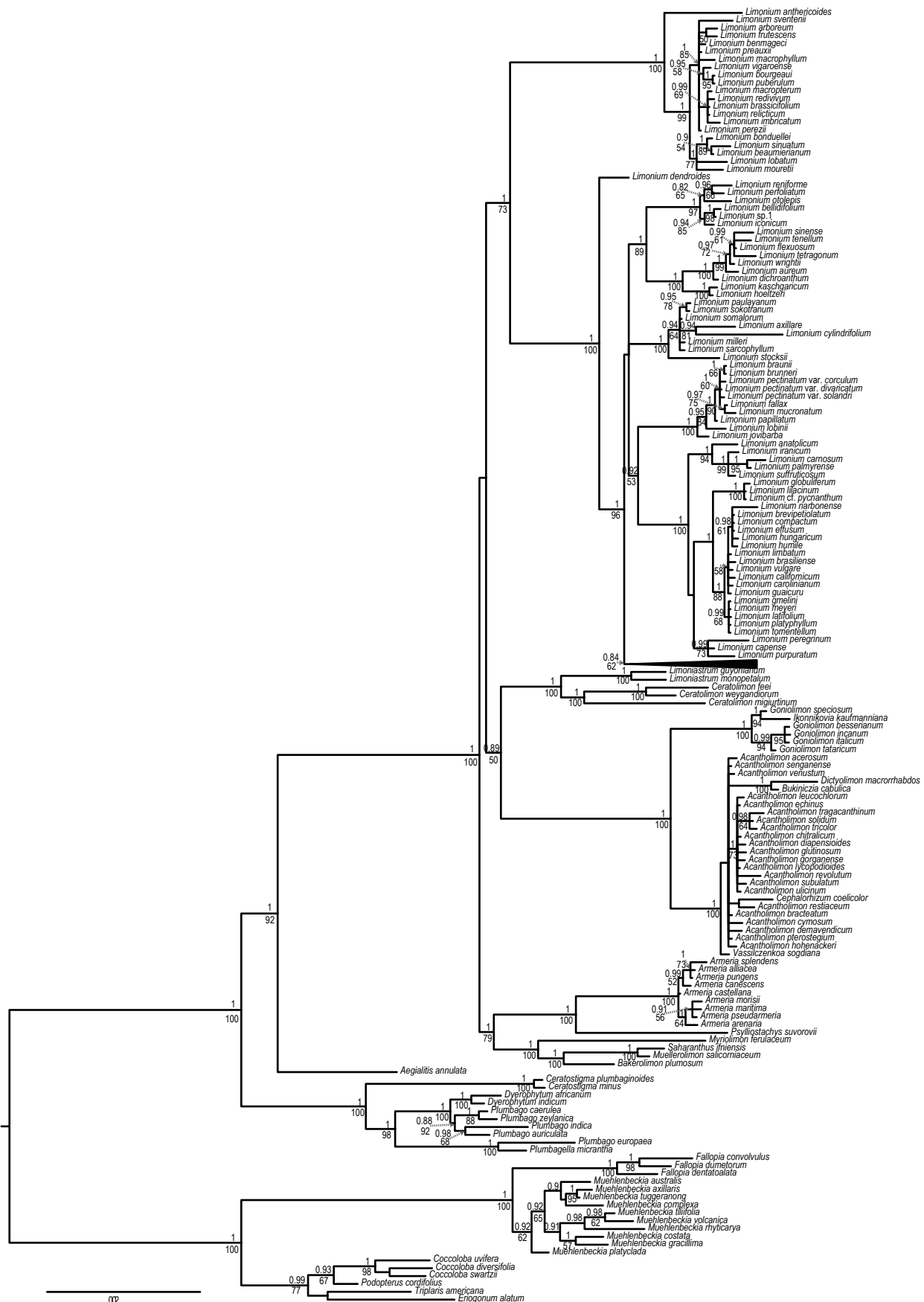

Supplement: Supplementary file 2 [file ECE3-8-12397-s002.pdf]
