# Supplementary material for: An expanded molecular phylogeny of Plumbaginaceae, with emphasis on Limonium (sea lavenders): Taxonomic implications and biogeographic considerations
Source: Ecol Evol. 2018 Dec 6;8(24):12397–424. doi: 10.1002/ece3.4553 (PMC6308857; doi:10.1002/ece3.4553)
Supplement: Supplementary file 3 [file ECE3-8-12397-s003.docx]

**Supporting Information**

**Data S1.** List of sampled species for which new sequence data were obtained, including source and voucher information; herbarium acronyms are according to Holmgren & Holmgren (1998, continuously updated), except for H.UoC: Herbarium of Department of Biology, University of Crete, Greece.

| ***Limonium* species** |
| --- |
| ***L.* cf. *aegaeum* Erben & Brullo**; Greece, Crete, Prefecture of Chania, Elafonisi, on the East side of the beach, opposite to the island, Lat. 35.27027, Long. 23.54385, August 2014; K. Koutroumpa 408 (Z). |
| ***L. albomarginatum* Brullo**; Greece. Peloponnese. Prefecture of Lakonia. Gerolimenas in Mani Peninsula; Artelari & Georgiou 1738 (UPA). |
| ***L. algarvense* Erben**; Portugal. Algarve. National Park of Ria Formosa. N 37.0295, E -8.0232, salt marshes, 17 April 2011; M.M. Romeiras 401 (LISC). |
| ***L. ammophilon* (Papatsou & Phitos) Domina**; Greece, Karpathos, Damatria, Lat. 35.27087, Long. 27.10000, August 2014; K. Koutroumpa 781 (Z). |
| ***L. amopicum* Erben & Brullo**; Greece, Karpathos, Finiki, On the rocks towards the West, next to the port, Lat. 35.45241, Long. 27.16666, 2m a.s.l., August 2014; K. Koutroumpa 796 (Z). |
| ***L. anatolicum* Hedge**; Turkey, Ankara, Şereflikoçhisar, 8-10 km before Şereflikoçhisar, Central Anatolia, Iran-Turan element, St. 2., 23 July 2015; F. Celep 3924 (GAZI). |
| ***L. anthericoides* (Schltr.) R.A.Dyer**; South Africa, Western Cape, Bredassdorp, Aasfontein, Low lying wet area near large vlei, 10 August 1983; E. Esterhuysen 36001 [E00770638] (E). |
| ***L. aphroditae* R.Artelari & Georgiou**; Greece, Kythira, Limnaria, On calcareous maritime rocks on the port and next to the beach, Lat. 36.20930, Long. 22.91664, August 2014; K. Koutroumpa 880 (Z). |
| ***L. aragonense* (Debeaux ex Willk.) Pignatti**; Spain, Prov. Teruel, Villel, vallon transversal, sur gypse en touffes denses, 14 July 1972; ipse 72/123 E [ZT-00077028] (ZT). |
| ***L. arboreum* (Willd.) Erben, A.Santos & Reyes-Bet.**; Spain, Canary Islands, Tenerife, Los Silos, road to Tierra del Trigo. Lower part of the cliff, ca. 10 m above the curve in the road, in a ledge with some humus. 28R 0323406/3138709, 256 m a.s.l. 23 May 2013; A. Jiménez AJ279-1 (Z). |
| ***L. archaeothirae* Erben & Brullo**; Greece, Santorini, Kamari; Thomas Koutroumpas 942 (Z). |
| ***L. articulatum* (Loisel.) Kuntze**; Corse, Ficaghiola, 30 April 2014, Botanical Garden of University of Zurich, living collection, accession of wild source; A. Désamoré 30/4/2014, Garden collector: K. Koutroumpa 1001 (Z). |
| ***L. asparagoides* (Batt.) Maire**; Morocco, Kariet Arkmane (= Arekmane), prov. Nador, au SE de cette ville, 35° 6' 9'' N, 2° 44' 40 '' W, 24 May 1994; J. Lambinon 94/307 & G. Van Den Sande [P05091481] (P). |
| ***L. aucheri* (Girard) Greuter & Burdet**; Turkey, Muğla: Datça, Knidos, Mediterranean, Sea level, St. 3, 17 July 2015; E. Cabi & F. Celep 3901 (GAZI). |
| ***L. aureum* (L.) Hill ex Kuntze**; China, Qinghai Province, Chindu Xian, Xiwu Xiang: S of Chumda, along east side of the Tongtian He (upper Chang Jiang). Latitude: 32° 56' N, Longitude: 97° 16' E, Habitat: Open dry rocky slopes with sparse shrubs, lower slopes cultivated, 16 August 1996; Sino-American-British Yushu Expedition (1996) 1901 [E00059615] (E). |
| ***L. auriculae-ursifolium* (Pourr.) Druce**; France, Loire-Atlantique, 3 müM La Turballe, Cote sauvage, Botanical Garden of University of Zurich, living collection, accession of wild source, donor: Muséum National d'Histoire Naturelle; Accession number: FR-0-Z-20120275, Garden collector: K. Koutroumpa 1003 (Z). |
| ***L. axillare* (Forssk.) Kuntze**; Qatar, Madinat Ash Shamal, Umm Tais, Habitat: Mangrove with Avicennia marina, 8 November 2013; Knees, Sabina Georgina 9036 (collected with R.Richer & Fran Gillespie) [E00647681] (E). |
| ***L. beaumierianum* (Coss. ex Maire) Maire**; Kew DNA bank 1932. |
| ***L. bellidifolium* (Gouan) Dumort.**; Turkey, İzmir, Çiğli, Homa Dalyanı Mediterranean, Sea level, St. 4, 19 July 2015; E. Cabi & F. Celep 3912 (GAZI). |
| ***L. benmageci* Marrero Rodr.**; Spain. Canary islands, Gran Canaria, Punta de La Aldea, plants collected from the field (wild) and transplanted in JBCVC; A. Jiménez AJben1. |
| ***L. biflorum* (Pignatti) Pignatti**; Spain, Baleares, Formentera, Estany Pudent, Les Salines, 31S CC68, 9 November 1986; M. Luceño & J. Pedrol 1618 JP [Z-000102463](Z). |
| ***L. binervosum* (G.E.Sm.) C.E.Salmon**; Spain, Estremadura, N Sâo Pedro de Moel. Alt. 30m. 14 June 2000; N. Nydegger 36863 [P05091530] (P). |
| ***L. bocconei* (Lojac.) Litard.**; Italy, Sicilia, Küstenfelsen, P Altarella, Levanzo, Isole Egadi, 7 August 1998; Jürg Röthlisberger 7/8/1998 [Z-000102464] (Z). |
| ***L. bollei* (Webb ex Wangerin) Erben**; Spain, Canary Islands, Lobos islet, Las Lagunillas, Flood area next to the path. Sandy-clay soil. 28R 0615575/3180266 7 m a.s.l. 08 May 2011; A. Jiménez, A. Santos-Guerra AJ256-1 (Z). |
| ***L. bonduellei* (T.Lestib.) Kuntze**; Morocco, 5km NW de Tazenakht sur la route d'Ourzazate (prov. Ourzazate), Alt. 1510m, 30° 36' N, 7° 14' W, 6 April 1995; D.Podlech 52610 [P05091477] (P). |
| ***L. bonifaciense* Arrigoni & Diana**; Corse, Bonifacio, Port Stagad, Botanical Garden of University of Zurich, living collection, accession of wild source, donor: Conservatoire Botanique National Méditerranéen de Porquerolles-HYERES-Frankreich; Accession number: FR-0-Z-20140897, Garden collector: K. Koutroumpa 1002 (Z). |
| ***L. bourgeaui* (Webb ex Webb) Kuntze**; Spain, Canary Islands, Lanzarote, Caleta de Famara, beyond Casas de Famara, Ravine facing the shore, Rocky soil, N 29° 07' 16,4'', W 13° 32' 00,8'', 131 m a.s.l., 5 May 2011; A. Jiménez AJ223-1 (Z). |
| ***L. brasiliense* (Boiss.) Kuntze**; Southern America, Argentina, Salina Chica, Pdo. Médanos, Prov. Buenos Aires, Muddy flats, 22 December 1981; TMP 13193 [L.2643266] (L). |
| ***L. brassicifolium* (Webb & Berthel.) Kuntze**; Spain, Canary islands, Riscos de Juel, La Gomera island, 26 March 2011; A. Santos-Guerra AS 3443-11 (ORT). |
| ***L. braunii* (Bolle) A.Chev.**; Cape Verde, Santo Antão Island, 2005; MC. Duarte 3526 (LISC). |
| ***L. brevipetiolatum* R.Artelari & Erben**; Greece, Ionian islands, Kefalonia, Argostoli, Fanari, on pebbly beach just on the right hand side in the beginning of the path leading to 'Fanari’; K. Koutroumpa 943 (Z). |
| ***L. brunneri* (Webb. ex Boiss.) Kuntze**; Cape Verde, Sal Island, 12 November 2011; C. Fernandes & M.M. Romeiras s.n. (LISC). |
| ***L. caesium* (Girard) Kuntze**; Kew DNA bank 1932, Serra & Crespo s.n. (K). |
| ***L. californicum* (Boiss.) A.Heller**; North America, Waldport, Oregon, Lint Slough across from the Waldport High School. 44° 25' 43'' N, 124° 3' 31'' W, 21 August 2002; Richard R. Halse 6313 [P05117997] (P). |
| ***L. calliopsium* Alf.Mayer**; Crete, Prefecture of Rethymno, Petre, On the rocky beach at the East of the village, Very hard-pointed, steep rocks next to the bar 'Vythos', Lat. 35.35850, Long. 24.37033, August 2014; K. Koutroumpa 668 (Z). |
| ***L. cancellatum* (Bertol.) Kuntze**; Croatia, Island of Vis south of Rukavac village, Botanical Garden of University of Zurich, living collection, accession of wild source, donor: National Botanical Garden Vacratot Hungary; Gergely Lunk 2014, Accession number: HR-0-Z-20150948, Garden collector: K. Koutroumpa 1004 (Z). |
| ***L. capense* (L.Bolus) L.Bolus**; South Africa, Western Cape: 3218 (Clanwilliam) CC, Limestone flats between Vredenberg and R27, 18 November 1995; Peter Goldblatt & J.C. Manning 10434 [WAG.1169163] (WAG). |
| ***L. carnosum* (Boiss.) Kuntze**; Kuwait, Failaka, east part of the island, 5 km from the port, 2m, very salty area, 4 Octomber 1981; A. Rawi 10838 [E00068909] (E). |
| ***L. carolinianum* (Walter) Britton**; Canada, Nova Scotia, Colchester Co., Economy, Economy Point, Dune, sable exposé, Botanical Garden of University of Zurich, living collection, accession of wild source, donor: Jardin botanique de Montréal, Montréal, Québec, Canada; Gaudette R. 07.09.2015, Accession number: CA-0-Z-20160420, Garden collector: K. Koutroumpa 1005 (Z). |
| ***L. carpathum* (Rech.f.) Rech.f.**; Greece, Karpathos, Between Ammopi and Akrotiri Volakas, on rocky terrace next to the sea, Lat. 35.48222, Long. 27.21016, 3-4m a.s.l., August 2014; K. Koutroumpa 766 (Z). |
| ***L. carpetanicum* Erben**; Spain, Ciudad Real, Alcazar de San Juan - Villafranca de los Caballeros, S Laguna de Las Yeguas, 590m, 26 July 2000; M. Nydegger 37193 [P05091517] (P). |
| ***L. carthaginense* (Rouy) C.E.Hubb. & Sandwith**; Kew DNA bank 705, M.W. Chase 705 (K). |
| ***L. cephalonicum* R.Artelari**; Greece, Ionian islands, Ithaki, Agia Ierousalim, 20 October 2014; K. Kougioumoutzis 20 Oct. 2014 (UPA). |
| ***L. chersonesum* Erben & Brullo**; Greece, Crete, Prefecture of Heraklion, Hersonissos, on a big rocky cliff in the port next to the 'Limenikos stathmos’, Lat. 35.32272, Long. 25.39288, 2m a.s.l., August 2014; K. Koutroumpa 44 (Z). |
| ***L. circaei* Pignatti**; Italy, Küstenfelsen, ca. 10müM, Grota delle Capre, Monte circeo, Lazio, 14 May 1999; Jürg Röthlisberger 14/5/99 [Z-000102468] (Z). |
| ***L. compactum* Erben & Brullo**; Greece, Peloponnese, Prefecture of Lakonia, Viglafia, Pounta beach, on salt marsh very close to the beach, Lat. 36.51805, Long. 22.98700, August 2014; K. Koutroumpa 926 (Z). |
| ***L. confusum* (Godr. & Gren.) Fourr.**; France, Aude 30 müM, Leucate, Botanical Garden of University of Zurich, living collection, accession of wild source, donor: Muséum National d'Histoire Naturelle - DJBZ  Paris cedex 05, France; Accession number: FR-0-Z-20120279, Garden collector: K. Koutroumpa 1006 (Z). |
| ***L. contortirameum* (Mabille) Erben**; Corse, Tallare, 20 April 2014, Botanical Garden of University of Zurich, living collection, accession of wild source; A. Désamoré 20/4/2014, Garden collector: K. Koutroumpa 1007 (Z). |
| ***L. cordatum* (L.) Mill.**; France, Küstenfelsen, <5müM, westlich Hafen von Nice, Dep. Alpes-Maritimes, N 43° 41' 29", E 7° 17' 33", 19 July 2002; Jürg Röthlisberger 19/7/2002 (Z). |
| ***L. cornarianum* Kypr. & R.Artelari**; Greece, Crete, Prefecture of Lasithi, Moni Kaspa gorge on the lower part towards the sea, Lat. 35.02189, Long. 26.05044, August 2014; K. Koutroumpa 751 (Z). |
| ***L. coronense* R.Artelari**; Greece, Peloponnese, Prefecture of Messinia, Kastro Koronis, On calcareous rocks, rocky beach, beneath the castle of Koroni, July 2007; Gioume Ioanna No. 1 (UPA). |
| ***L. corsicum* Erben**; Corse, Galeria, 1 May 2014, Botanical Garden of University of Zurich, living collection, accession of wild source; A. Désamoré 1/5/2014, Garden collector: K. Koutroumpa 1009 (Z). |
| ***L. cossonianum* Kuntze**; Spain, New Town, Alicante, 7 September 1994-09-07 Alt.: 5m; Kew DNA bank 1480, Lledó, M.D. & M.B. Crespo 7-IX-94, No. 10729 (ABH), [K000696171] (K). |
| ***L. costae* (Willk.) Pignatti**; Spain, Cuenca, El Hito, El Hito lagoon, 30S 0525377/4412581, 820m a.s.l., in vegetation patches on soil mounds above the water level, 1 June 2011; A. Jiménez, S. Blasco AJ287-1 (Z). |
| ***L. cosyrense* (Guss.) Kuntze**; Botanical Garden of University of Zurich, living collection, accession not of wild source, donor: Chelsea Physic Garden, London, United Kingdom; Accession number: XX-0-Z-20150592, Garden collector: K. Koutroumpa 1010 (Z). |
| ***L. crateriforme* Erben & Brullo**; Greece, Crete, Prefecture of Lasithi, Akrotiri Mavromouri, After the village 'Dionissos' on the cliff beside the road, Lat. 35.21914, Long. 26.18665, August 2014; K. Koutroumpa 151 (Z). |
| ***L. creticum* R. Artelari**; Greece, Crete, Prefecture of Heraklion, Matala caves, 11 September 2013; K.Koutroumpa, M.Megariti, S.Pirintsos 2013.09.11-01 (H.UoC). |
| ***L. cylindrifolium* (Forssk.) Verdc. ex Cufod.**; Saudi Arabia, Farasan Island, Al Husain Bay, Low sand dunes near shore, 17 February 1995; I.S. Collenette 9246 [E00121225] (E). |
| ***L. cymuliferum* (Boiss.) Sauvage & Vindt**; Algeria, Djelfa, Few km s. of Hassi BAhbah (N. of Djelfra), Saline habitat, 09 June 1971; Davis, P.H. 53418 [E00770648] (E). |
| ***L. cythereum* R.Artelari & Georgiou**; Greece, Island of Kythira, Chalkos bay, On the river dry banks and on limestone rocks next to the beach, Lat. 36.13544, Long. 23.03400, August 2014; K. Koutroumpa 902 (Z). |
| ***L. daveaui* Erben**; Portugal, Estuário do Tejo, ca. Base Aérea Montijo, 38.7299°/ -9.0104°, Salt marshes, 11 June 2011; M.M. Romeiras 410 (LISC). |
| ***L. delicatulum* (Girard) Kuntze**; Spain, Alicante, Santa Pola, against the base of the headleand, 7 September 1994; Kew DNA Bank 1478, M.D. Lledó & M.B. Crespo 7-IX-94, No 10730 (ABH), [K000696170] (K). |
| ***L. dendroides* Svent.**; Spain, Canary islands, La Gomera, El Azadoe, Reinforcement population; A. Jiménez AJden4 (Z). |
| ***L. densissimum* (Pignatti) Pignatti**; Spain; Kew DNA bank 1479, Curco et al. 15-X-94 (ABH). |
| ***L. dichotomum* (Cav.) Kuntze**; Spain, Madrid, Rivas Vaciamadrid, wasteland next to Rivas Futura, Gipsum soil, E slope, N 40° 20' 17.7", W 03° 31' 00.0", 593m a.s.l., 1 June 2011; A. Jiménez, S. Blasco AJ285 (Z). |
| ***L. dichroanthum* (Rupr.) Ikonn.-Gal.**; Kyrgyzstan, Naryn Oblasty, Between Baetov and Ugut, 41° 22' 18'' N, 74° 52' 56'' E, Eroded salt flat areas, 30 August 1998; K.R. Robertson, J.B. Taft, G.A. Lazkov 6076 [E00711141] (E). |
| ***L. dodartii* (Girard) Kuntze**; France, Vendée, Noirmoutier en l ìle, Roche Biron, Botanical Garden of University of Zurich, living collection, accession of wild source, donor: Ville de Nantes, Nantes cedex 01, France; Accession number: FR-0-Z-20150586, Garden collector: K. Koutroumpa 1011 (Z). |
| ***L. dufourii* (Girard) Kuntze**; Spain; Kew DNA bank 1476, M.D. Lledó & M.B. Crespo s.n. (ABH). |
| ***L. ebusitanum* (Font Quer) Font Quer**; Spain, Cap Regana, côte SE, prov. Mallorca, 2 June 1987; G.Sag 1987-6-2 [P05086682] (P). |
| ***L. echioides* (L.) Mill.**; Greece, Crete, Island of Gavdos, Beach of Korfos, Marls-sand in front of a tavern, on marls, Lat. 34.83064, Long. 24.11121, August 2014; K. Koutroumpa 579 (Z). |
| ***L. effusum* (Boiss.) Kuntze**; Turkey, Balıkesir, Ayvalık, Mediterranean, St. 6, 19 July 2015; E. Cabi & F. Celep 3916 (GAZI). |
| ***L. elaphonisicum* Alf.Mayer**; Greece, Crete, Island of Gavdos, at the West coast of Sarakiniko, Lat. 34.86301, Long. 24.10796, August 2014; K. Koutroumpa 589 (Z). |
| ***L. erectum* Erben**; Botanical Garden of University of Zurich, living collection, accession not of wild source, donor: Spain, Alcalá de Henares, Madrid, Real Jardin Botanico Juan Carlos I; Accession number: 20150393/ES-0-ALCA-2009-0095, Garden collector: K. Koutroumpa 1012 (Z). |
| ***L. estevei* Fern.Casas**; Spain, Mojacar, towards Carboneras; Kew DNA bank 1473, No 9231 (ABH), [K000696167] (K). |
| ***L. fallax* (Coss. ex Wangerin) Maire**; Morocco, Oued Dra between Goulimime and Tan-Tan, Stony slopes of valley, 23 March 1969; P. Davis & J.D. Davis 48593 [E00770646] (E). |
| ***L. flexuosum* (L.) Kuntze**; Russian Federation, Nerczynsk, July 1891; F. Karo 199 [E00770640] (E). |
| ***L. frederici* (Barbey) Rech.f.**; Greece. Karpathos, Sokastro (Esokastro) islet, On the north-west rocks next to *Salsola carpatha*, Lat. 35.59740, Long. 27.05827, August 2014; K. Koutroumpa 818 (Z). |
| ***L. frutescens* (Lem.) Erben, A.Santos & Reyes-Bet.**; Spain, Canary Islands, Tenerife, Buenavista, Teno, La Monja viewpoint, around the viewpoint, in rocky soil with some sand, 28R 0315325/3139271, 180 m a.s.l., 22 May 2011; A. Jiménez AJ276-1 (Z). |
| ***L. furfuraceum* (Lag.) Kuntze**; Spain, Alicante, Valencia, Playa de San Juan, Felsen am Strand, 10 May 2015, Botanical Garden of University of Zurich, living collection, accession of wild source, donor: Rayko Jonas; Rayko Jonas 10.09.2015, Accession number: ES-0-Z-20170830, Garden collector: K. Koutroumpa 1013 (Z). |
| ***L. girardianum* (Guss.) Fourr.**; Spain, Valencia, Dehesa Saler, Mallada del Garrofer, Alt.:2, 11 November 1994; Kew DNA bank 1481, M.D. Lledó & M.B. Crespo 11-XI-94, No 10732 (ABH), [K000696172] (K). |
| ***L. globuliferum* (Boiss. & Heldr.) Kuntze**; Turkey, Aksaray, between Eskil and Gölyazı, Central Anatolia, Iran-Turan element, St. 4., 25 July 2015; K. İldeniz & F. Celep 3936 (GAZI). |
| ***L. gmelini* (Willd.) Kuntze**; Turkey, Aksaray, from Aksaray to Konya, ca. 10 km, Central Anatolia, Iran-Turanian element, St. 2., 26 July 2015; K. İldeniz & F. Celep 3943 (GAZI). |
| ***L. gougetianum* (Girard) Kuntze**; Botanical Garden of University of Zurich, living collection, accession not of wild source, donor: Botanical Garden Brno, Czech Republic; Accession number: XX-0-Z-20150397, Garden collector: K. Koutroumpa 1014 (Z). |
| ***L. grabusae* Erben & Brullo**; Greece, Crete, Island of Imeri Gramvousa, Close to the 'port', Lat. 35.60727, Long. 23.58137, August 2014; K. Koutroumpa 464 (Z). |
| ***L. graecum* (Poir.) Rech.f.**; Turkey, İzmir, Çeşme, Altınkum, Mediterranean, Sea level, St. 4., 18 July 2015; E. Cabi & F. Celep 3906 (GAZI). |
| ***L. greuteri* Erben**; Corse, La Revellata, 25 April 2014, Botanical Garden of University of Zurich, living collection, accession of wild source; A. Désamoré 25/4/2014, Garden collector: K. Koutroumpa 1015 (Z). |
| ***L. guaicuru* (Molina) Kuntze**; Chile, Región Valparaíso, Provincia de Petorca, Zapallar, Lat. 32° 30' S, Long. 71° 30' W, 8 March 1992; M.F. Gardner & C.N. Page 5110 [E00215437] (E). |
| ***L. gymnesicum* Erben**; Botanical Garden of University of Zurich, living collection, accession not of wild source, donor: Banco de Germoplasma Andaluz-Cordoba; Accession number: XX-0-Z-20150438, Garden collector: K. Koutroumpa 1016 (Z). |
| ***L. hibericum* Erben**; Spain, Zaragoza, Bujaraloz, La Playa lagoon, In the bank, dry band of the lagoon, on soil with salt crust, with *Stipa*, 30T 0734109/4589966, 325 m a.s.l, 7 August 2011; A. Jiménez, S. Blasco AJ297 (Z). |
| ***L. hierapetrae* Rech.f.**; Crete, Prefecture of Lasithi, Ferma beach, 8 September 2013; K.Koutroumpa, M.Megariti, S.Pirintsos 2013.09.08-11 (H.UoC). |
| ***L. hoeltzeri* (Regel) Ikonn.-Gal.**; Kyrgyzstan, Issyk-kul. Taragay River, 13 km west-northeast of Kara-Saj, Lat. 40° 41' 40'' N, Long. 77° 49' 43'' E, 03 July 1999; Phillippe Loy R., Taft John B., Dietrich Christopher H., Warren Emily, Lazkov Georgy A. 30924 [E00714953] (E). |
| ***L. humile* Mill.**; Norway, Oslo, Gressholmen, UTM 32 V 596054 6639489 (WGS84), Botanical Garden Oslo NORWAY, wild collection, 4 October 2015; Accession number: NO-0-Z-20160477, Kristina Bjureke, Tor S. Mjaaland 04.10.2015 (Z). |
| ***L. hungaricum* Klokov**; Botanical Garden of WWU-MS Munster, Germany, living collection, accession not of wild source; Accession number: HU-0-MSTR-5727/20140257. |
| ***L. hyblaeum* Brullo**; Botanical Garden of University of Zurich, living collection, accession not of wild source, donor: Centro di Ateneo, Padova, Italia; Accession number: XX-0-Z-20160696, Garden collector: K. Koutroumpa 1017 (Z). |
| ***L. iconicum* (Boiss. & Heldr.) Kuntze**; Turkey, Ankara, Şereflikoçhisar, 30 km before Şereflikoçhisar, Central Anatolia, Iran-Turan element, St. 1, 23 July 2015; F. Celep 3919 (GAZI). |
| ***L. imbricatum* (Webb ex Girard) Hubbard ex L.H.Bailey**; Spain, Canary Islands, Tenerife, Punta del Hidalgo, below the Roque Dos Hermanos, Rocky-gravelly soil, in ledges in the cliff, 28R 0372156/3161441, 90 m a.s.l., 25 May 2011; A. Jiménez AJ283-1 (Z). |
| ***L. insigne* (Coss.) Kuntze**; Spain, Murcia, Calabardina, road to Aguilas, N 37° 25' 48.3", W 1° 31' 14.1", 8m a.s.l., 29 April 2011; A. Jiménez, S. Blasco AJ213-1 (Z). |
| ***L. iranicum* (Bornm.) Lincz.**; Iran, Tehran, Near Mardabad (South of Karaj), Plain, salty soil, partly cultivated, 20 September 1974; I.C. Hedge, P. Wendelbo & H. Foroughi 14677 [E00453664] (E). |
| ***L. jovi-barba* (Webb) Kuntze**; Cape Verde. S. Vicente, Monte Verde, 13 December 2011; C. Fernandes & M.M. Romeiras s.n. (LISC). |
| ***L. kardamylii* R.Artelari & Kamari**; Greece, Peloponnese, Prefecture of Messinia, In a small beach that you can find when entering the Kardamyli village and take a right turn next to a square, On conglomerate rocks next to the sea, Lat. 36.88827, Long. 22.23063, August 2014; K. Koutroumpa Pel.01 (Z). |
| ***L. kaschgaricum* (Rupr.) Ikonn.-Gal.**; Kyrgyzstan, Issyk-Kul, South shore of Lake Issyk Kul, 10 km east of Kadzhi-Saj, Tien Shan Flora Region of former Soviet Central Asia. Semi-desert habitat, Lat. 42° 10' 33'' N, Long. 77° 18' 55'' E, 03 July 1999; L.R. Phillippe, J.B. Taft, C.H. Dietrich, E. Warren, G.A Lazkov 30890 [E00714808] (E). |
| ***L. kraussianum* (Buchinger ex Boiss.) Kuntze**; South Africa, Ratelrivier vlei, 2 km from Die Dam/Gansbaai turnoff to Bredasdorp, 1 January 1986; G. Geraishuizen 4146 [WAG.1169568] (WAG). |
| ***L. lanceolatum* (Hoffmanns. & Link) Franco**; Portugal, SW Alentejo, Vila Nova Mil Fontes, 37.7255°/ -8.7679°, Salt marshes, 21 May 2011; M.M. Romeiras 439 (LISC). |
| ***L. latebracteatum* Erben**; Spain, Lleida, Ivars d'Urgell, Castellsera, 2 km S KreuzungToledo, La Guardia, Villanueva de Bogas bei Abzweigung, 310m, 1 August 2000; M. Nydegger 37239 [P05090887] (P). |
| ***L. latifolium* (Sm.) Kuntze**; Romania, Ruinengelände, ca. 5müM, Histria, Judetul, Constanta, 10 August 1999; Jürg Röthlisberger 10/8/1999 [Z-000102467] (Z). |
| ***L. laxiusculum* Franco**; Portugal, Estremadura, Praia de Magoito sur Ericeira, 70m, 17 June 2000; M. Nydegger 36898 [P05093581] (P). |
| ***L. lilacinum* (Boiss. & Bal.) Wagenitz**; Turkey, Ankara, Şereflikoçhisar, 8-10 km before Şereflikoçhisar, Central Anatolia, Iran-Turan element, St. 2., 23 July 2015; F. Celep 3922 (GAZI). |
| ***L. limbatum* Small**; America, United States of America, Texas. Reevs Co.: Pecos, jct. I-20 & US 285, in low alkali swale, 28 May 1990; R. D. Worthington 18488 [L.2643555] (L). |
| ***L. lobatum* (L.f.) Chaz.**; Spain, Canary Islands, Tenerife, Arico, El Viso, Sandy-gravelly soil, in path-sides and with *Cistus* in open areas, 28R 0354381/3116966, 400-450m a.s.l., 19 May 2011; A. Jiménez AJ272-3 (Z). |
| ***L. lobinii* N.Kilian & Leyens**; Cape Verde, Santiago Island, Serra Malagueta, May 2011; C. Fernandes & M.M. Romeiras s.n. (LISC). |
| ***L. longebracteatum* Erben**; Spain, Cuenca, El Hito, El Hito lagoon, Soil with salt crust around the lagoon, 30S 0525377/4412581, 820 m a.s.l., 1 June 2011; A. Jiménez, S. Blasco AJ286-1 (Z). |
| ***L. lowei* R.Jardim, M.Seq., Capelo, J.C.Costa & Rivas Mart.**; Madeira, Porto Santo Island, at the south of the capital, 7 April 2011; A. Santos-Guerra & F. Fernández AS 3565-11, ORT 42924 (ORT). |
| ***L. macrophyllum* Kuntze**; Spain, Canary Islands, Tenerife, Chamorga, Montaña Tafada, Soil with some humus accumulated in ledges, mostly with a N orientation and under the influence of the tradewinds mist, 28R 0387720/3161765, 588 m a.s.l., 21 May 2011; A. Jiménez AJ274-1 (Z). |
| ***L. macropterum* (Webb & Berthel.) Kuntze**; Spain, Canary islands, Riscos Bascos, El Hierro island, 22 March 2011; A. Santos-Guerra ORT s.n. (ORT). |
| ***L. majus* (Boiss.) Erben**; Spain, Granada, Baza, Cullar-Baza ne Venta del Peral, 930m, 16 July 2000; M. Nydegger 37115 [P05090885] (P). |
| ***L. meandrinum* Erben & Brullo**; Greece, Karpathos island, Apella, On maritime calcareous rocks, August 2014; K. Koutroumpa 834 (Z). |
| ***L. meyeri* (Boiss.) Kuntze**; Georgia, Davit Gareji, 12 miles from caves, Grassland steppe, Lat. 41° 33' 50'' N, Long. 45° 20' 0'' E, 8 August 2009; Edinburgh Tbilisi Expedition to Georgia (2009) 18 [E00375757] (E). |
| ***L. milleri* Ghaz. & J.R.Edm.**; Oman, Thamrait road near end of Gorge, Stony wadi bed by roadside, 6 October 1992; I.S. Collenette 8418 [E00046382] (E). |
| ***L. minoicum* Erben & Brullo**; Greece, Crete, Prefecture of Lasithi, Tertsa, On calcareous maritime rocks, Lat. 34.99146, Long. 25.52927, August 2014; K. Koutroumpa 313 (Z). |
| ***L. minutiflorum* (Guss.) Kuntze**; Italy, Sizilien, Prov. Messina, Capo di Milazzo, nordlich Milazzo, 21 August 1980; K. P. Buttler Nr.18747 (UPA). |
| ***L. minutum* (L.) Chaz.**; Botanical Garden of University of Zurich, living collection, accession not of wild source, donor: Utrecht University Botanic Gardens TD, Utrecht, Netherlands; Accession number: xx0u-1984BL00199/ 20160739, Garden collector: K. Koutroumpa 1018 (Z). |
| ***L. mouretii* (Pitard) Maire**; Morocco, Meknès, Bekrit, Material source: C.E. Salmon; RBGE Accession number: 19250429 A, Garden collector: P. Brownless 566 [E00706083] (E). |
| ***L. mucronatum* (L.f.) Chaz.**; Morocco, Between Tamri and Agadie, Frequent in sandy areas, February 1984; Salmon, M. & Fillan, M. 22/3 [E00770647] (E). |
| ***L. multiflorum* Erben**; Portugal, Cascais, Guincho, Cabo Raso, 38.7089°/ -9.4858°, Maritime cliffs 5m, 20 May 2011; M. M. Romeiras 422 (LISC). |
| ***L. multiforme* (Martelli) Pignatti**; Italy, Prov. of Toscana, S. part: Parco della Maremma (Monti dell'Uccellina), c. 12km SSW of Grosseto, Coastal cliffs, 28 August 1977; E. Georgiadou & A. Strid 364 (ATH). |
| ***L. nydeggeri* Erben**; Botanical Garden of University of Zurich, living collection, accession not of wild source, donor: Parque Botânico da Tapada Da Ajuda, Lisboa, Portugal; Accession number: PP-0-AJUDA-14-532/ 20150984, Garden collector: K. Koutroumpa 1019 (Z). |
| ***L. obtusifolium* (Rouy) Erben**; Corse: Bonifacio, ilot du Fazzio, Botanical Garden of University of Zurich, living collection, accession of wild source, donor: Conservatoire Botanique National Méditerranéen de Porquerolles, HYERES, France; Accession number: FR-0-Z-20140898, Garden collector: K. Koutroumpa 1020 (Z). |
| ***L. ocymifolium* (Poir.) Kuntze**; Greece, Island of Milos. Fyropotamos, Ground with gravel, 2009; O. Georgiou 11 (UPA). |
| ***L. oligotrichum* Erben & Brullo**; Greece, Crete, Prefecture of Lasithi, Xerokampos, Along the coast line, Lat. 35.04224, Long. 26.23440, August 2014; K. Koutroumpa 212 (Z). |
| ***L. otolepis* (Schrenk) Kuntze**; North America, Cultivated plants of California, 7 August 1970; E. McClintock [E00770643] (E). |
| ***L. ovalifolium* (Poir.) Kuntze**; France, Loire-Atlantique, Le Croisic, Rocher de L`Ours, Botanical Garden of University of Zurich, living collection, accession of wild source, donor: Muséum National d'Histoire Naturelle – DJBZ, Paris cedex 05, France; Accession number: FR-0-Z-20120276, Garden collector: K. Koutroumpa 1021 (Z). |
| ***L. palmyrense* (Post) Dinsm.**; Jordan, El Umari, Salt flats, 24 April 1955; Hunting Aero Survey 127D [E00453655] (E). |
| ***L. papillatum* (Webb & Berthel.) Kuntze**; Spain, Canary Islands, Fuerteventura, Jandía, track from Morro Jable to the tip of Jandía, Sandy soil in the trackside, 28R 0551246/3106263, 21 m a.s.l., 09 May 2011; A. Jiménez, A. Santos-Guerra AJ262 (Z). |
| ***L. paulayanum* (Vierh.) Ghaz. & J.R.Edm.**; Yemen, Socotra Archipelago, Socotra, Wadi Irih draining s. into Nogad plain due S of Hadiboh, Steep sided wadi with large limestone boulders at bottom, 28 February 1989; A.G. Miller, L. Guarino, N. Obadi, M. Hassan & N. Mohammed 8526 [E00453714] (E). |
| ***L. pectinatum* var. *corculum* (Webb & Berthel.) G.Kunkel & Sunding**; Spain, Canary Islands, Tenerife, Puerto de la Cruz, Cliff near the tunnel, 28R 0349311/3144356, 31 m a.s.l., 25 May 2011; A. Jiménez AJ284-1 (Z). |
| ***L. pectinatum* var. *divaricatum* (Pit.) G.Kunkel & Sunding**; Spain, Canary Islands, Tenerife, Las Galletas, 31 March 2011; A. Santos-Guerra AS 3342-11 (ORT). |
| ***L. pectinatum* var. *solandri* (Webb. & Berthel.) Kuntze**; Spain, Canary Islands, El Hiero, Arenas Blancas, 22 March 2011; A. Santos-Guerra AS 3315-11 (ORT). |
| ***L. perezii* (Stapf) Hubbard ex L.H.Bailey**; Spain, Canary islands, Tenerife, Masca, Roque Tarucho. 28R 0319805/3132508, ca. 850 m a.s.l., 13 March 2012; A. Jiménez AJ303-45 (Z). |
| ***L. perfoliatum* (Kar. ex Boiss.) Kuntze**; Iran, Golestan, Gorgan, 42 km N. of Pahlavedezh which is 15 km N. of Bandar Shah Habitat, On edge of ditch alongside *Tamarix* sp., 2 October 1977; T.F. Hewer 4044 [E00453647] (E). |
| ***L. pigadiense* (Rech.f.) Rech.f.**; Greece, Karpathos island, Damatria, Lat. 35.45241, Long. 27.16666 August 2014; K. Koutroumpa 777 (Z). |
| ***L. platyphyllum* Lincz.**; Botanical Garden of University of Zurich, living collection, accession not of wild source, donor: Botanischer Garten Krefeld, Deutschland; Accession number: XX-0-Z-20160403, Garden collector: K. Koutroumpa 1022 (Z). |
| ***L. plurisquamatum* Erben**; Portugal, S. Martinho do Porto, S. Martinho cliffs. Maritime cliffs. 65m, 39.5186°/ -9.1423°, 10 June 2011; M.M. Romeiras 445 (LISC). |
| ***L. preauxii* (Webb & Berthel.) Kuntze**; Spain, Canary Islands, Gran Canaria, Fataga ravine, near the road from Maspalomas to Fataga, 28R 0443174/3077843, 415 m a.s.l., 19 July 2012; A. Jiménez AJ316 (Z). |
| ***L. proliferum* (d'Urv.) Erben & Brullo**; Greece, Crete, Prefecture of Lasithi, Moni Faneromenis, On the East side, On calcareous rocks, Lat. 35.21959, Long. 26.07011, August 2014; K. Koutroumpa 118 (Z). |
| ***L. pruinosum* (L.) Chaz.**; Morocco, ED. 10-20 km N. of Erfoud, Sandy, subslaine desert (shrubby chenopods etc.), 06 April 1969; P. Davis & J.D. Davis 49143 [E00770644] (E). |
| ***L. pseudebusitanum* Erben**; Spain, Balearic Islands, Majorca, Cabo Blanco, 17 September 1973; D. Bramwell & Z.I. Bramwell 38 [E00240957] (E). |
| ***L. puberulum* (Webb) Kuntze**; Spain, Canary Islands, Lanzarote, Haría, on the way to Mirador del Río, Since 1 km before the viewpoint to the parking lot and in the cliff, In rills and in the steppe, Sandy-gravelly soil, N 29° 12' 18,2'', W 13° 28' 52,3''. 422 m a.s.l., 5 March 2012; A. Jiménez AJ222-2 (Z). |
| ***L.* cf. *pycnanthum* (K. Koch) Kuntze**; Turkey, Aksaray, Hamidiye village, Central Anatolia, Iran-Turan element, St. 1., 25 July 2015; K. İldeniz & F. Celep 3931 (GAZI). |
| ***L. pylium* R.Artelari**; Greece, Peloponnese, Prefecture of Ilia, Matzakoura beach, close to the village Agios Andreas, 5km from Katakolo, On karstic, calcareous rocks, July 2007; Gioume Ioanna No. 4 (UPA). |
| ***L. recticaule* Erben & Brullo**; Greece, Crete, Prefecture of Heraklion, Malia, In front of the Hotel 'Anthousa beach (Aktia)', On rocks in the sea, very rough rocks, Lat. 35.29325, Long. 25.43638, August 2014; K. Koutroumpa 26 (Z). |
| ***L. recurvum* C.E.Salmon subsp. *humile* (Girard) Ingr.**; United Kingdom, Scotland, (VC 74) Wigtownshire, Mull of Galloway, in a gully east of Lagnagatchie, Eroded vegetated cliff, 08 September 2010; McHaffie, Heather & Frachon, Natacha, Garden collection number: 3451, RBGE Accession number: 20100965 B, [E00668930] (E). |
| ***L. redivivum* (Svent.) G.Kunkel & Sunding**; Spain, Canary Islands, La Gomera, Benchijigua, slopes of Punta de Arisel, 28R 0282413/3109549, 663 m a.s.l., 25 July 2012; A. Jiménez AJ268 (Z). |
| ***L. relicticum* R.Mesa & A.Santos**; Spain, Canary islands, La Gomera, Teguerguenche, 30 March 2011; A. Santos-Guerra AS 2790-09 (ORT). |
| ***L. remotispiculum* (Lacaita) Pignatti**; Italy, Lido Macarro, Marina di Maratea, PZ, on rocky cliffs on the southernmost part of the beach; Mario Coiro s.n. (Z). |
| ***L. reniforme* (Girard) Lincz.**; Iran, Fars. Bakhteghan-lake, prope Kharameh, Salty lake, Salty clay soil, dampy, beside water, 04 October 1974; H. Foroughi & M. Assadi 15036 [E00453643] (E). |
| ***L. roridum* (Sibth. & Sm.) Brullo & Guarino**; Greece, Gavdos island, Sarakiniko, Lat. 34.85893, Long. 24.11171, August 2014; K. Koutroumpa 582 (Z). |
| ***L. santapolense* Erben**; Spain, Dehesa Saler, Mallada del Garrofer, 2m, 3 June 1994; Kew DNA bank 1484, M.D. Lledó, M.D. & M.B. Crespo 3-VI-94, No 9224 (ABH), [K000696175] (K). |
| ***L. saracinatum* R.Artelari**; Greece, Ithaki island, South Kaminia, 18 Octomber 2014; K. Kougioumoutzis 18 Oct. 2014 (UPA). |
| ***L. sarcophyllum* Ghaz. & J.R.Edm.**; Oman, Bimma, Sharquyah, Lat. 23° 1' N, Long. 59° 7' E, 3 December 1993; I.M. McLeish 3392 [E00132418] (E). |
| ***L. scabrum* (Thunb.) Kuntze**; South Africa; Kew DNA bank 5889, M.W. Chase 5889 (K). |
| ***L. scopulorum* M.B.Crespo & Lledó**; Spain, Alicante, Denia, Cova Tallada, 10m; Kew DNA bank 1467, E. Camuñas, L. Serra & M.B. Crespo 3-VIII-94 (K), No 9723 (ABH). |
| ***L. sieberi* (Boiss.) Kuntze**; Greece, Crete, Prefecture of Chania, Almyrida, West coast, On calcareous marls, Lat. 35.45055, Long. 24.20366, August 2014; K. Koutroumpa 502 (Z). |
| ***L. sinuatum* (L.) Mill.**; Greece, Crete, Prefecture of Heraklion, Kokkini Chani, On the beach in front of the Hotel 'Themis', Sandy-rocky beach, Lat. 35.33169, Long. 25.26046, August 2014; K. Koutroumpa 53 (Z). |
| ***L. sitiacum* Rech.f.**; Greece, Crete, Prefecture of Lasithi, Pacheia Ammos, On the West side of the beach, Lat. 35.11142, Long. 25.80292, August 2014; K. Koutroumpa 90 (Z). |
| ***L. sokotranum* (Vierh.) Radcl.-Sm.**; Yemen, Socotra archipelago, Socotra, Extreme W end of Nogad Plain nr Ras Qatanhin, Base of escarpment, rocky slopes with sand dunes towards the sea, Alt. 5-50m, 27 February 1989; A.G. Miller, L. Guarino, N. Obadi, M. Hassan & N. Mohammed M.8503 [E00676679] (E). |
| ***L. somalorum* (Vierh.) Hutch. & E.A.Bruce**; Kew DNA bank 1921. |
| ***L. sougiae* Erben & Brullo**; Greece, Crete, Prefecture of Chania, Sougia, On the hard rocks at the East side of the beach, Lat. 35.24816, Long. 23.81795, August 2014; K. Koutroumpa 342 (Z). |
| **L. sp.1**; Turkey, St. 2, Seyfe Lake, Central Anatolia, Iran-Turan element, 24 July 2015; K. İldeniz & F. Celep 3925 (GAZI). |
| **L. sp.2**; Greece, Crete, August 2014; K. Koutroumpa 300 (Z). |
| ***L. spectabile* (Svent.) G.Kunkel & Sunding**; Spain, Canary islands, Tenerife, Barranco (Ravine) de Natero, Teno (NW Tenerife), 5 m a.s.l. 6 May 2009; A. Santos-Guerra AS 2634-09 (ORT). |
| ***L. spreitzenhoferi* Erben & Brullo**; Greece, Kythira island, Fournoi beach, On maritime calcareous rocks, Lat. 36.35355, Long. 22.96716, August 2014; K. Koutroumpa 862 (Z). |
| ***L. stenotatum* (Rech.f.) Erben & Brullo**; Greece, Crete, Prefecture of Lasithi, Agia Fotia, Lat. 35.19531, Long. 26.15031, August 2014; K. Koutroumpa 143 (Z). |
| ***L. subglabrum* Erben**; Spain, Granada, Alhama de Granada, Granada, 600 m E La Mala, 780m, 5 July 2000; M. Nydegger 37001 [P05090886] (P). |
| ***L. suffruticosum* (L.) Kuntze**; Azerbaijan, Caucasus, Districtus Schemakha, montes Malyi Harami, 18 September 1969; G.M Proskuriakova 94 [E00453658] (E). |
| ***L. supinum* (Girard) Pignatti**; Spain, Alicante, Alcoy, above El Molinar, Roadside, on sandy marl soil, 30S 0720389/4284321, 636 m a.s.l., 19 August 2011; A. Jiménez, S. Blasco AJ300 (Z). |
| ***L. sventenii* A. Santos & M.L.Fernández**; Spain, Canary Islands, Gran Canaria, San Isidro, Montaña Amagro, Rocky soil, The population ascends along the slope of the mountain, 28R 0433483/3111408, 432 m a.s.l., 21 March 2011; A. Jiménez, E. Conti, H. Schäfer AJ197-1 (Z). |
| ***L. tabernense* Erben**; Spain, Almería, Tabernas, NW slope of the Alhamilla range, Slopes and temporal drainage basins, N 37^o^ 01' 01,1'', W 02^o^ 24' 52,5'', 377 m a.s.l., 05 June 2011; A. Jiménez, S. Blasco AJ295-1 (Z). |
| ***L. tenellum* (Turcz.) Kuntze**; Kew DNA bank 2360; Fred Mayer s.n. |
| ***L. tetragonum* (Thunb.) Bullock**; New Caledonia, Prov. Du Noumea, Pointe Maa, 7 May 2006; Kew DNA bank 24719, Y. Pillon et al. 409 (NOU). |
| ***L. toletanum* Erben**; Botanical Garden of University of Zurich, living collection, accession not of wild source, donor: Real Jardin Botanico Juan Carlos I, Alcalá de Henares, Madrid, Spain; Accession number: ES-0-ALCA-2011-0649/ 20150395, Garden collector: K. Koutroumpa 1023 (Z). |
| ***L. tomentellum* (Boiss.) Kuntze**; Living collection of the RBGE; Garden collector: P. Brownless s.n., RBGE Accession number: 19695219/ 19695219A, R31 Rock Garden. |
| ***L. tournefortii* (Boiss.) Erben**; Spain, Toledo, La Guardia, Villanueva de Bogas bei Abzweigung, 600m, 10 June 2000; M. Nydegger 36858 [P05090889] (P). |
| ***L. tuberculatum* (Boiss.) Kuntze**; Spain, Canary Islands, Lobos islet, Las Lagunillas, Flood area next to the path, Sandy-clay soil, 28R 0615575/3180266, 7m a.s.l., 8 May 2011; A. Jiménez, A. Santos-Guerra AJ257 (Z). |
| ***L. tubiflorum* (Del.) Kuntze**; Egypt, Abusir (Maryut), 15 March 1938; Boetje-van Ruyven, MRS 91 [L.2644102] (L). |
| ***L. tunetanum* (Barratte) Maire**; Tunisia, El Djerid, between Kebili and Mansoura oasis, Flat sandy desert covered with plant-bult dunes, 18 September 1968; P.H. Davis 48092 [E00770645] (E). |
| ***L. vanandense* Erben & Brullo**; Greece, Karpathos island, Tristomo, 15 June 2013; I. Bazos 4582 (UPA). |
| ***L. vigaroense* Marrero Rodr. & R.S.Almeida**; Canary islands, Gran Canaria, Barranquillo de Las Magarzas, 15 June 2011; Oscar Saturno Hernández Banco_ADN_2723, DNA bank of the Canarian Flora, Jardín Botánico Canario “Viera y Clavijo” – Unidad Asociada CSIS. |
| ***L. virgatum* (Willd.) Fourr.**; Greece, Crete, Prefecture of Heraklion, Kalamaki, 11 September 2013; K.Koutroumpa, M.Megariti, S.Pirintsos 2013.09.11-16 (H.UoC). |
| ***L. vulgare* Mill.**; Azores, Santa Maria, Maia, 676871/4090192, 37m, 11 June 2011; Mónica Moura LI-MAMA-001, AZB 1570 (AZB). |
| ***L. wrightii* (Hance) Kuntze**; Japan, Kyushu, Kagoshima, Kurio, Shimoyaku-mura, Yakushima Island, Kumage-gun, On rock near beach, 19 October 1969; H. Izumi & M. Fujimoto 78 [E00770639] (E). |
| ***L. xerocamposicum* Erben & Brullo**; Greece, Crete, Prefecture of Lasithi, Xerokampos, on the way to Agia Irini, On harsch calcareous rocks, Lat. 35.02787, Long. 26.21087, August 2014; K. Koutroumpa 215 (Z). |
| ***L. xiliense* Erben & Brullo**; Greece, Peloponnese, Prefecture of Lakonia, Archangellos, On calcareous maritime rocks, Lat. 36.62972, Long. 22.88083, August 2014; K. Koutroumpa 930 (Z). |
| **Species of other Plumbaginaceae genera** |
| ***Acantholimon echinus* (L.) Bunge**; Greece, Taygetos gebergte, op 2000m hoogte(bij top), ten ZW van Anawrouti, losliggende kalkstenen, oosthelling, hellingshoek 30Âº(T65), 14 June 1969; A. Fokkinga 1969-06-14 [L.2647181] (L). |
| ***Acantholimon hohenackeri* (Jaub. & Spach) Boiss.**; Persia, Prov. Azerbaijan, Marand c. 10km towards Tabrz, Banks between road and railway, Alt. c. 1650m, 6 June 1971; Jennifer Lamond 3744 [Z-000102460] (Z). |
| ***Acantholimon leucochlorum* Rech.f. & Schiman-Czeika**; Afganistan, Prov. Paktia, Jali, Ali Khel to Kabul, between Dre Khalla and Shinkei, Sirkei Pass, Dry grazed slopes at top of pass, Alt. c. 3000m, 11 July 1965; Jennifer Lamond 2507 [Z-000102459] (Z). |
| ***Acantholimon senganense* Bunge**; Persia, Prov. Kordestan, Kuh-e-Hamzeh Arab, between Bijar and Hamadan, Limestone mountain slopes, Alt. c. 2200-2600m, 1 July 1971; Jennifer Lamond & F. Terme 4345 [Z-000102457] (Z). |
| ***Acantholimon tragacanthinum* (Jaub. & Spach) Boiss.**; Persia, Prov. Azerbaijan, road from Marand to Zonuz, Stony slopes, Alt. c. 1500m, 7 June 1971; Jennifer Lamond 3792 [Z-000102456] (Z). |
| ***Acantholimon venustum* Boiss.**; Turkey, Swas., Ziyaret Pass, Steppe vegetation on open slopes, Alt: 2100m, 13 June 1985; J. Archibald 6700 [ZT-00077026] (ZT). |
| ***Armeria alliacea* (Cav.) Hoffmanns. & Link**; Living collection of the Botanical Garden of St. Gallen, donor: Alpengarten Parco Nationale Stelvio Bormio; Accession number: XX-0-STGAL-470/2011, Garden collector: Hanspeter Schumacher s.n. (Z). |
| ***Armeria arenaria* (Pers.) Schult.**; Botanical Garden of University of Zurich, living collection, accession not of wild source, donor: Gradina Botanica "Alexandru Borza" Cluj-Napoca, Romania; Accession number: XX-0-Z-20160803, Garden collector: K. Koutroumpa 1029 (Z). |
| ***Armeria canescens* (Host) Boiss.**; Greece, Sterea Ellas, Prefecture Etolias-Akarnanias, Region of Valtos, Mt. Ori Valtou, c. 8km E of Patiopoulo village on the way to Livadia, The area above the road and up to the crest line of Korfoula (1643m) summit, Alt. 1100-1643m, Lat. 39 05N Long. 21 18 E, Stony slopes with small meadows at the middle parts of the area, Limestone, 5 June 2005; L. Giannakos 593 (UPA). |
| ***Armeria castellana* Boiss. & Reut. ex Leresche**; Spain, Santander, Fuentes de Picos de Europa, La Colladina, Short turf over limestone, 01 July 1980; J.M. Gardiner & R.J.D McBeath R1035 [E00198386] (E). |
| ***Armeria maritima* (Mill.) Willd.**; Botanical Garden of University of Zurich, living collection, accession not of wild source, donor: National Botanical Garden Vacratot, Hungary; Accession number: XX-0-Z-20160934, Garden collector: K. Koutroumpa 1028 (Z). |
| ***Armeria morisii* Boiss.**; Botanical Garden of University of Zurich, living collection, accession not of wild source, donor: Gradina Botanica "Alexandru Borza", Cluj-Napoca, Romania; Accession number: XX-0-Z-19950245, Garden collector: K. Koutroumpa 1030 (Z). |
| ***Armeria pseudarmeria* (Murray) Mansf.**; Portugal, Lissabon, Botanical Garden of University of Zurich, living collection, accession of wild source, donor: Freie Universität zu Berlin, Berlin, Deutschland; Accession number: PT-0-Z-19840216, Garden collector: K. Koutroumpa 1031 (Z). |
| ***Armeria pungens* (Link) Hoffmanns. & Link**; Portugal, Cape St. Vincent, Sand over limestone, 30 March 2006; M.J.Y. Foley 2206 [E00246339] (E). |
| ***Armeria splendens* (Lag. & Rodr.) Webb**; Spain, Sierra Nevada; Kew DNA bank 1895, M.W. Chase 1895 (K). |
| ***Bakerolimon plumosum* (F.Phil.) Lincz.**; Chile, Región III, Atacama, Provincia de Chañaral, Parque Nacional Pan de Azúcar, Quebrada del Castillo, Lat. 26° 13' 14'' S, Long. 70° 35' 22'' W, Coastal desert fog zone, Flat valley bottom, 24 November 2008; R. Baines, M. Gardner, P. Hechenleitner, C. Morter, & D. Rae 120 [E00230461] (E). |
| ***Bukiniczia cabulica* (Boiss.) Lincz.**; Botanical Garden of University of Zurich, living collection, accession not of wild source, donor: Botanisk Have Århus C Denmark; Accession number: XX-0-Z-20070284, Garden collector: K. Koutroumpa 1024 (Z). |
| ***Ceratostigma plumbaginoides* Bunge**; Botanical Garden of University of Zurich, living collection, accession not of wild source, donor: Vogt Roger, Erlenbach, Schweiz; Accession number: XX-0-Z-19760464, Garden collector: K. Koutroumpa 1032 (Z). |
| ***Dyerophytum africanum* (Lam.) Kuntze**; Namibia, Karas, In Klinghardts main basin, Mountain slope, Alt. 656m, 27^o^ 19’ 9” S, 15 ^o^ 45’ 47” E, 17 September 2000; CA Mannheimer CM 996 [WAG.1169473] (WAG). |
| ***Dyerophytum indicum* (Gibs. ex Wight) Kuntze**; Oman, Dhofar, Jabal Samhan north facing slopes Lat. 17° 3' 11'' N, Long. 54° 38' 45'' E, Dissected limestone plateau with *Euclea* and *Cordia* spp. 7 September 2014; Knees, MacKinnon, MacLaren & Page 215 [E00695995] (E). |
| ***Goniolimon besserianum* (Schult.) Kusn.**; Botanical Garden of University of Zurich, living collection, accession not of wild source, donor: Grădina Botanică "Anastasie Fătu" IASI, Romania; Accession number: RO-0-IAGB20128983 / 20160602, Garden collector: K. Koutroumpa 1025 (Z). |
| ***Goniolimon incanum* (L.) Hepper**; Botanical Garden of University of Zurich, living collection, accession not of wild source, donor: Hortus Zoologicus Pragensis, Praha 7 – Troja, Czech Republic; Accession number: XX-0-Z-20160689, Garden collector: K. Koutroumpa 1026 (Z). |
| ***Goniolimon italicum* Tammaro, Pignatti & G.Frizzi**; Botanical Garden of University of Zurich, living collection, accession not of wild source, donor: Humboldt-Universität zu Berlin, Berlin, Deutschland; Accession number: 20160367, Garden collector: K. Koutroumpa 1027 (Z). |
| ***Goniolimon speciosum* (L.) Boiss.**; Russian Federation, Altay (Gorno-Altaysk) Bank of Kunduyak river, mountain massif Taldouair, Lat. 49° 59' 45'' N, Long. 88° 13' 31'' E, Grassy steppe, 25 August 2012; Barnaul South Siberian Botanic Garden & Edinburgh Expedition to Russian Altai 50 [E00656075] (E). |
| ***Goniolimon tataricum* (L.) Boiss.**; Living collection of the Botanical Garden of St. Gallen, donor: Botanischen Garten Stuttgart hohenheim Alpengarten; Accession number: XX-0-STGAL-67/2006, Garden collector: Hanspeter Schumacher s.n. (Z). |
| ***Limoniastrum guyonianum* Durieu ex Boiss.**; Algeria, Biskra SS2, Near Biskra, Edge of desert, 15 May 1971; P.H. Davis 52372 [E00770652] (E). |
| ***Muellerolimon salicorniaceum* (F.Muell.) Lincz.**; Australia, Western Australia, Meekatharra (S), 30 km N of Tuckanarra, E side of a gypseous dune on margin of a salt lake, Lat. -26.9167, Long. 118.25, 16 October 1981; Kew DNA bank 1635, Craven L.A. 7128 (K), [CANB 379595.1] (CANB). |
| ***Myriolimon ferulaceum* (L.) Lledó, Erben & M.B.Crespo**; Portugal, São Martinho do Porto, Salir, 39.5012° / -9.1493°, 10 June 2011; M.M. Romeiras 411 (LISC). |
| ***Plumbagella micrantha* (Ledeb.) Spach**; China, Qinghai Province, Nangqen Xian, Beca Xiang, below Gaersi monastry, SE of Becaka, Lat. 31° 50' N, Long. 96° 29' E, Gentle slopes with degraded alpine meadow, steeper slopes with groves of *Juniperus*, Growing in bare places in meadow and along edge of road, 07 September 1996; Sino-American-British Yushu Expedition (1996) 2947 [E00061458] (E). |
| ***Plumbago auriculata* Lam.**; Botanical Garden of University of Zurich, living collection, Tropical greenhouse; Accession number: XX-0-Z-19963567, Garden collector: K. Koutroumpa 1033 (Z). |
| ***Plumbago caerulea* Kunth**; Bolivia, La Paz, Inquisivi, Following the mule trail between Inquisivi and the Rio Khatu Bridge ca. 1-3 km N of Inquisivi. Semi-arid to seasonally humid forests, 16^o^ 53' S 67^o^ 09' W, 2400-2500, 29 March 1989; Marko Lewis 35396 [U.1487667] (U). |
| ***Plumbago europaea* L.**; Greece, bij Arachova aan wegkant bloemen purperrood, kelk met grote klierharen, 8 Oktober 1976; A.M.W. Mennega 917 [U.1495156] (U). |
| ***Plumbago indica* L.**; Botanical Garden of University of Zurich, living collection, Tropical greenhouse; Accession number: XX-0-Z-19966122, Garden collector: K. Koutroumpa 1000 (Z). |
| ***Plumbago zeylanica* L.**; Brasil, Fonte dos Protomartires do Brasil, Porto Seguro, Coastal rain forest with small river and clearings with disturbed ground, Alt. 0-10m, Approx. 39^o^05' N 16 ^o^ 26' S, 21 March 1974; R. M. Harley 17219 [U.1487661] (U). |
| ***Vassilczenkoa sogdiana*(Lincz.) Lincz.**; Afghanistan, Samangan, 7 miles S. of Khulm, in the Tashkurghan gorge, Grassy slope, E. facing on chalk, 15 May 1969; T.F. Hewer 1130 [E00013192] (E). |

**Data S2.** Modified CTAB DNA extraction protocol used in this study.

-Start from 20-30 mg dry leaf tissue

1. Prepare CTAB buffer, add polyvinylpyrrolidone (PVP). Heat till dissolution of PVP and then add B-mercapto-ethanol just before use.

Quantities:

No. of Samples  CTAB buffer PVP B-mercapto-ethanol

1 700µl 0.02gr 2.5µl

2. Grind the samples with two or three metal beads, when well grinded in powder add 700µl of the CTAB solution (with PVP and B-mercapto-ethanol added).

3. Incubate the samples at 65° water bath for 1h -1h30. Invert tubes each 30min.

+ Add 4μl RNAse A (100mg/μl) at 37° for 15 minutes.

4. Add 700 µl of 24 :1 Chloroform : Isoamyl Alcohol and verify that the powder is covered by the liquid. Shake slowly by vortexing or inverting the tubes gently.

5. Centrifuge 10 min at max speed. During this time prepare new 1.5ml tubes and label them.

6. Pipette the upper phase (aqueous). Be careful to not touch the middle and lower phase.

7. Centrifuge those tubes with the aqueous phase 10 min at max speed and pipette it in new tubes (in order to remove any remaining debris that you might have been transferred from the middle and lower phase).

8. Estimate the volume of the aqueous phase

9. Add 0.54 volume of cold isopropanol (2-propanol) and 0.08 volume of 7.5M ammonium or sodium acetate. Invert the tubes for about 20-30 times (do not vortex) to mix the solutions and leave at room temperature for 30 minutes (or in ice).

10. Centrifuge for 30 min at max speed.

11. Remove the liquid by inverting the tubes slowly (be careful not to lose the pellet, if you are not sure you can do it with a pipet).

12. Add 700 µl of cold 70% Ethanol, mix well and centrifuge 10 min.

13. Remove the liquid (be careful of the pellet). Add 500µl of cold 100% Ethanol, mix well and centrifuge 10 min.

14. Remove the liquid and dry the pellet by inverting the tubes on a towel paper for 5min.

15. Dry 5 min open under the fume hood or put 5 min in the speed-vac.

16. Verify that the Ethanol is well dry (if necessary leave a few more minutes to dry) and then add 30 or 50 µl of water. Then, either let the tubes for ca. 3 hours at room temp, or one night in the fridge, or 1 hour in the water bath at 65°C before checking the quantity and quality of the extracted DNA.

Data S3. Genbank accession numbers and codes of sequences provided to us by the authors of Lledó *et al*. (2005a) and used in this study, and will be deposited in Dryad. Newly generated sequences are marked with an asterisk.

| ***Limonium* species** | ***trnL*-*F*** | ***rbcL*** | ***matK*** | **ITS** |
| --- | --- | --- | --- | --- |
| ***L.* cf. *aegaeum* Erben & Brullo** | MH561010* | MH582788* | MH582872* |  |
| ***L. albomarginatum* Brullo** | MH561056* | MH582667* | MH582899* | MH582550* |
| ***L. algarvense* Erben** | MH561040* | MH582715* | MH582955* |  |
| ***L. ammophilon* (Papatsou & Phitos) Domina** | MH561021* | MH582784* | MH582855* | MH582564* |
| ***L. amopicum* Erben & Brullo** | MH561029* | MH582763* | MH582876* | MH582571* |
| ***L. anatolicum* Hedge** | MH560985* | MH582745* | MH582839* | JX983724 |
| ***L. anthericoides* (Schltr.) R.A.Dyer** | MH561136* |  |  | MH582626* |
| ***L. aphroditae* R.Artelari & Georgiou** | MH561057* | MH582669* | MH582903* | MH582574* |
| ***L. aragonense* (Debeaux ex Willk.) Pignatti** | MH561086* | MH582725* | MH582908* | MH582615* |
| ***L. arboreum* (Willd.) Erben, A.Santos & Reyes-Bet.** | MH561121* | LCFCarbor2 | MH583006* | MH582643* |
| ***L. archaeothirae* Erben & Brullo** | MH561020* | MH582764* | MH582864* | MH582592* |
| ***L. articulatum* (Loisel.) Kuntze** | MH561084* | MH582670* | MH582909* | MH582579* |
| ***L. asparagoides* (Batt.) Maire** | MH561035* |  |  | MH582620* |
| ***L. aucheri* (Girard) Greuter & Burdet** | MH561034* | MH582787* | MH582865* | MH582576* |
| ***L. aureum* (L.) Hill ex Kuntze** | MH561103* | JN187124 | MH582970* | JN187115 |
| ***L. auriculae-ursifolium* (Pourr.) Druce** | MH561077* | MH582699* | MH582907* | MH582605* |
| ***L. axillare* (Forssk.) Kuntze** | AJ391323 | AJ286362 | MH582887* | JX983660 |
| ***L. beaumierianum* (Coss. ex Maire) Maire** | MH561139* | MH582808* | MH582993* | MH582627* |
| ***L. bellidifolium* (Gouan) Dumort.** | MH561108* | MH582789* | MH582978* | MH582518* |
| ***L. benmageci* Marrero Rodr.** | MH561122* | MH582801* | MH583004* |  |
| ***L. biflorum* (Pignatti) Pignatti** |  | KJ608050 | MH582961* |  |
| ***L. binervosum* (G.E.Sm.) C.E.Salmon** | KP159566 | LCFCbiner2 |  | MH582607* |
| ***L. bocconei* (Lojac.) Litard.** | MH561076* | MH582671* | MH582910* | MH582616* |
| ***L. bollei* (Webb ex Wangerin) Erben** | MH561036* | MH582716* | MH582956* |  |
| ***L. bonduellei* (T.Lestib.) Kuntze** | MH561133* | MH582809* | MH582994* | MH582628* |
| ***L. bonifaciense* Arrigoni & Diana** | MH561058* | MH582698* | MH582911* | MH582580* |
| ***L. bourgeaui* (Webb ex Webb) Kuntze** | MH561128* | MH582812* | MH582995* | MH582630* |
| ***L. brasiliense* (Boiss.) Kuntze** | MH560981* | MH582736* |  | MH582540* |
| ***L. brassicifolium* (Webb & Berthel.) Kuntze** | MH561130* | MH582802* | MH582999* | MH582632* |
| ***L. braunii* (Bolle) A.Chev.** | MH560998* | MH582752* | MH582891* | MH582526* |
| ***L. brevipetiolatum* R.Artelari & Erben** | MH560970* | MH582731* | MH582840* | MH582544* |
| ***L. brunneri* (Webb. ex Boiss.) Kuntze** | MH560999* | MH582748* | MH582890* | MH582527* |
| ***L. caesium* (Girard) Kuntze** |  | MH582765* | MH582879* | MH582623* |
| ***L. californicum* (Boiss.) A.Heller** | MH560983* | MH582739* | MH582841* | MH582539* |
| ***L. calliopsium* Alf.Mayer** | MH561011* | MH582766* | MH582866* | MH582552* |
| ***L. camposanum* M. Erben** |  | KJ608041 |  | AJ222841 |
| ***L. cancellatum* (Bertol.) Kuntze** | MH561090* | MH582672* | MH582944* | MH582604* |
| ***L. capense* (L.Bolus) L.Bolus** | MH560967* | MH582727* |  |  |
| ***L. carnosum* (Boiss.) Kuntze** | MH560987* | LCFCcarno2 | MH582853* | JX983729 |
| ***L. carolinianum* (Walter) Britton** | MH560984* | KJ773631 | KJ772894 | MH582547* |
| ***L. carpathum* (Rech.f.) Rech.f.** | MH561025* | MH582767* | MH582856* |  |
| ***L. carpetanicum* Erben** | MH561049* | MH582713* |  |  |
| ***L. carthaginense* (Rouy) C.E.Hubb. & Sandwith** | LCFCcarth1 | LCFCcarth2 | MH582912* | MH582582* |
| ***L. cephalonicum* R.Artelari** | MH561059* | MH582673* | MH582913* | MH582599* |
| ***L. chersonesum* Erben & Brullo** | MH561015* |  | MH582867* |  |
| ***L. circaei* Pignatti** | MH561071* | MH582674* | MH582914* | MH582583* |
| ***L. compactum* Erben & Brullo** | MH560971* | MH582732* | MH582842* |  |
| ***L. confusum* (Godr. & Gren.) Fourr.** | MH561082* | MH582675* | MH582945* |  |
| ***L. contortirameum* (Mabille) Erben** | MH561098* | MH582676* | MH582938* | MH582586* |
| ***L. cordatum* (L.) Mill.** | MH561083* | MH582693* | MH582946* | MH582597* |
| ***L. cornarianum* Kypr. & R.Artelari** | MH561069* | MH582694* | MH582900* | MH582565* |
| ***L. coronense* R.Artelari** | MH561088* | MH582677* | MH582915* | MH582598* |
| ***L. corsicum* Erben** | MH561085* | MH582678* | MH582916* | MH582581* |
| ***L. cossonianum* Kuntze** | LCFCcosso1 | KJ608017 | MH582962* | AJ132331 |
| ***L. costae* (Willk.) Pignatti** | MH561097* |  |  |  |
| ***L. cosyrense* (Guss.) Kuntze** | MH561060* | MH582679* | MH582917* |  |
| ***L. crateriforme* Erben & Brullo** | MH561016* | MH582768* | MH582857* | MH582560* |
| ***L. creticum* R. Artelari** | MH561008* | MH582762* | MH582875* | MH582575* |
| ***L. cylindrifolium* (Forssk.) Verdc. ex Cufod.** |  | LCFCcylin2 | MH582888* |  |
| ***L. cymuliferum* (Boiss.) Sauvage & Vindt** | MH561047* | MH582724* |  | MH582612* |
| ***L. cythereum* R.Artelari & Georgiou** | MH561055* | MH582680* | MH582904* | MH582554* |
| ***L. daveaui* Erben** | MH561078* | MH582700* | MH582918* | MH582609* |
| ***L. delicatulum* (Girard) Kuntze** | AJ391324 | Y16903 | MH582963* | AJ222851 |
| ***L. dendroides* Svent.** | LCFCdendr1 | MH582796* | MH582883* | MH582625* |
| ***L. densissimum* (Pignatti) Pignatti** | LCFCdensi1 | LCFCdensi2 | MH582919* | MH582610* |
| ***L. dichotomum* (Cav.) Kuntze** | MH561092* | MH582704* | MH582920* | AJ222858 |
| ***L. dichroanthum* (Rupr.) Ikonn.-Gal.** | MH561104* | MH582800* | MH582971* |  |
| ***L. dodartii* (Girard) Kuntze** | KP159568 | MH582701* | MH582921* | MH582606* |
| ***L. dufourii* (Girard) Kuntze** | AJ391326 | AJ286363 | MH582922* | AJ222840 |
| ***L. ebusitanum* (Font Quer) Font Quer** |  | KJ608043 |  | MH582603* |
| ***L. echioides* (L.) Mill.** | MH561038* | KJ608051 | MH582966* | MH582618* |
| ***L. effusum* (Boiss.) Kuntze** | MH560972* | MH582733* | MH582966* | MH582545* |
| ***L. elaphonisicum* Alf.Mayer** | MH561012* | MH582769* | MH582868* |  |
| ***L. erectum* Erben** | MH561093* | MH582705* | MH582950* | MH582594* |
| ***L. estevei* Fern.Casas** | LCFCestev1 | LCFCestev2 | MH582968* | MH582570* |
| ***L. fallax* (Coss. ex Wangerin) Maire** | MH561004* | MH582753* | MH582895* | MH582530* |
| ***L. flexuosum* (L.) Kuntze** | MH561105* | MH582798* | MH582972* | MH582515* |
| ***L. frederici* (Barbey) Rech.f.** | MH561028* | MH582785* | MH582878* | MH582572* |
| ***L. frutescens* (Lem.) Erben, A.Santos & Reyes-Bet.** | MH561123* | LCFCfrute2 | MH583007* | MH582642* |
| ***L. furfuraceum* (Lag.) Kuntze** | LCFCfurfu1 | Y16902 | MH582947* | AJ222856 |
| ***L. girardianum* (Guss.) Fourr.** | LCFCgirar1 | MH582720* | MH582964* | AJ222845 |
| ***L. globuliferum* (Boiss. & Heldr.) Kuntze** | LCFCglobu1 |  | MH582850* | MH582549* |
| ***L. gmelini* (Willd.) Kuntze** | MH560975* | MH582740* | MH582844* | JX983716 |
| ***L. gougetianum* (Girard) Kuntze** | MH561061* | MH582681* | MH582923* |  |
| ***L. grabusae* Erben & Brullo** | MH561009* | MH582779* | MH582869* | MH582555* |
| ***L. graecum* (Poir.) Rech.f.** | MH561023* |  | MH582870* | MH582557* |
| ***L. greuteri* Erben** | MH561081* | MH582682* | MH582948* | MH582585* |
| ***L. guaicuru* (Molina) Kuntze** | MH560990* | MH582737* | MH582845* | MH582536* |
| ***L. gymnesicum* Erben** | MH561062* | KJ608045 | MH582939* | AJ222842 |
| ***L. hibericum* Erben** | MH561100* | MH582708* | MH582924* | MH582589* |
| ***L. hierapetrae* Rech.f.** | MH561050* | MH582668* | MH582937* |  |
| ***L. hoeltzeri* (Regel) Ikonn.-Gal.** |  | MH582794* | MH582976* | MH582513* |
| ***L. humile* Mill.** | MH560974* | JN893200 | JN894792 |  |
| ***L. hungaricum* Klokov** | MH560973* | MH582734* | MH582849* | MH582541* |
| ***L. hyblaeum* Brullo** | MH561072* | MH582683* | MH582925* | MH582608* |
| ***L. iconicum* (Boiss. & Heldr.) Kuntze** | MH561110* | MH582791* | MH582980* | MH582520* |
| ***L. imbricatum* (Webb ex Girard) Hubbard ex L.H.Bailey** | MH561132* | MH582810* |  | MH582639* |
| ***L. insigne* (Coss.) Kuntze** | LCFCinsig1 | MH582781* | MH582880* | MH582622* |
| ***L. iranicum* (Bornm.) Lincz.** | MH560986* | MH582730* | MH582854* | JX983712 |
| ***L. jovi-barba* (Webb) Kuntze** | MH561007* | MH582755* |  | MH582534* |
| ***L. kardamylii* R.Artelari & Kamari** | MH561089* | MH582684* | MH582926* | MH582587* |
| ***L. kaschgaricum* (Rupr.) Ikonn.-Gal.** | MH561107* | MH582795* | MH582977* | MH582514* |
| ***L. kraussianum* (Buchinger ex Boiss.) Kuntze** | MH561070* |  |  | MH582591* |
| ***L. lanceolatum* (Hoffmanns. & Link) Franco** | MH561041* | MH582709* | MH582957* | MH582567* |
| ***L. latebracteatum* Erben** | MH561101* | MH582710* | MH582927* |  |
| ***L. latifolium* (Sm.) Kuntze** | MH560977* | MH582741* |  | MH582546* |
| ***L. laxiusculum* Franco** | MH560991* |  |  | MH582602* |
| ***L. lilacinum* (Boiss. & Bal.) Wagenitz** | MH560968* | MH582746* | MH582851* | JX983693 |
| ***L. limbatum* Small** | MH560980* | MH582738* |  | MH582537* |
| ***L. lobatum* (L.f.) Chaz.** | MH561137* | LCFClobat2 | MH582998* | AJ132333 |
| ***L. lobinii* N.Kilian & Leyens** | MH561006* | MH582756* | MH582898* |  |
| ***L. longebracteatum* Erben** | MH561039* | MH582721* | MH582965* | MH582617* |
| ***L. lowei* R.Jardim, M.Seq., Capelo, J.C.Costa & Rivas Mart.** | MH561037* | MH582717* | MH582958* | MH582568* |
| ***L. macrophyllum* Kuntze** | MH561125* | LCFCmacro2 | MH583003* | MH582644* |
| ***L. macropterum* (Webb & Berthel.) Kuntze** | MH561127* | MH582803* | MH583002* | MH582634* |
| ***L. majus* (Boiss.) Erben** | MH561042* |  |  |  |
| ***L. meandrinum* Erben & Brullo** | MH561091* | MH582685* | MH582906* |  |
| ***L. meyeri* (Boiss.) Kuntze** | MH560976* | MH582742* | MH582846* | AB979593 |
| ***L. milleri* Ghaz. & J.R.Edm.** | MH560996* | MH582759* | MH582889* | MH582521* |
| ***L. minoicum* Erben & Brullo** | MH561051* | MH582686* | MH582928* |  |
| ***L. minutiflorum* (Guss.) Kuntze** | MH561063* | MH582696* |  | MH582577* |
| ***L. minutum* (L.) Chaz.** | MH561073* | KJ608027 | MH582929* | AJ132332 |
| ***L. mouretii* (Pitard) Maire** | MH561138* | MH582814* | AF204854 | MH582629* |
| ***L. mucronatum* (L.f.) Chaz.** | MH561005* | MH582754* | MH582896* | MH582531* |
| ***L. multiflorum* Erben** | MH561079* | MH582707* | MH582936* | MH582613* |
| ***L. multiforme* (Martelli) Pignatti** | MH561064* | MH582722* | MH582930* | MH582584* |
| ***L. narbonense* Mill.** | AJ391327 | AJ286364 |  | AJ222838 |
| ***L. nudum* (Boiss. & Buhse) Kuntze** |  |  |  | JX983672 |
| ***L. nydeggeri* Erben** | MH561043* | MH582718* | MH582959* |  |
| ***L. obtusifolium* (Rouy) Erben** | MH561075* | MH582688* | MH582931* | MH582593* |
| ***L. ocymifolium* (Poir.) Kuntze** | MH561102* | MH582780* | MH582932* |  |
| ***L. oligotrichum* Erben & Brullo** | MH561031* | MH582770* | MH582858* | MH582558* |
| ***L. otolepis* (Schrenk) Kuntze** | MH561111* | MH582792* | MH582982* | JX983682 |
| ***L. ovalifolium* (Poir.) Kuntze** | MH561044* | MH582719* | MH582960* | MH582569* |
| ***L. palmyrense* (Post) Dinsm.** | MH560988* | MH582729* |  | MH582535* |
| ***L. papillatum* (Webb & Berthel.) Kuntze** | MH561000* | MH582750* | MH582897* | MH582528* |
| ***L. parvibracteatum* Pignatti** | LCFCparvi1 | LCFCparvi2 |  |  |
| ***L. paulayanum* (Vierh.) Ghaz. & J.R.Edm.** | MH560992* | MH582757* |  | MH582522* |
| ***L. pectinatum* var. *corculum* (Webb & Berthel.) G.Kunkel & Sunding** | MH561001* |  | MH582892* | MH582532* |
| ***L. pectinatum* var. *divaricatum* (Pit.) G.Kunkel & Sunding** | MH561002* | MH582749* | MH582893* | MH582533* |
| ***L. pectinatum* var. *solandri* (Webb. & Berthel.) Kuntze** | MH561003* | MH582751* | MH582894* | MH582529* |
| ***L. peregrinum* (P.J.Bergius) R.A.Dyer** | LCFCpereg1 | JQ412383 |  |  |
| ***L. perezii* (Stapf) Hubbard ex L.H.Bailey** | MH561135* | MH582804* | MH583005* | MH582640* |
| ***L. perfoliatum* (Kar. ex Boiss.) Kuntze** | MH561112* | MH582793* | MH582981* | JX983681 |
| ***L. pigadiense* (Rech.f.) Rech.f.** | MH561053* | MH582689* | MH582905* |  |
| ***L. platyphyllum* Lincz.** | MH560978* | MH582743* | MH582847* | MH582542* |
| ***L. plurisquamatum* Erben** | MH561087* | MH582702* | MH582940* |  |
| ***L. preauxii* (Webb & Berthel.) Kuntze** | MH561124* | MH582805* |  | MH582636* |
| ***L. proliferum* (d'Urv.) Erben & Brullo** | MH561017* | MH582771* | MH582863* | MH582562* |
| ***L. pruinosum* (L.) Chaz.** | MH561032* | MH582772* | MH582881* | MH582621* |
| ***L. pseudebusitanum* Erben** | MH561065* | KJ608033 | MH582902* | MH582578* |
| ***L. puberulum* (Webb) Kuntze** | MH561134* | MH582811* | MH582996* | MH582631* |
| ***L. purpuratum* Hubbard ex L.H.Bailey** | LCFCpurpu1 | LCFCpurpu2 | AY042537 |  |
| ***L.* cf. *pycnanthum* (K. Koch) Kuntze** | MH560969* | MH582747* | MH582852* | MH582548* |
| ***L. pylium* R.Artelari** | MH561066* | MH582690* | MH582954* | MH582588* |
| ***L. recticaule* Erben & Brullo** | MH561018* | MH582773* | MH582859* | MH582561* |
| ***L. recurvum* C.E.Salmon subsp. *humile* (Girard) Ingr.** | MH561080* | MH582703* | MH582933* |  |
| ***L. redivivum* (Svent.) G.Kunkel & Sunding** | MH561129* | MH582807* | MH583001* | MH582635* |
| ***L. relicticum* R.Mesa & A.Santos** | MH561131* | MH582806* | MH583000* | MH582633* |
| ***L. remotispiculum* (Lacaita) Pignatti** | MH561074* | MH582691* | MH582953* | MH582601* |
| ***L. reniforme* (Girard) Lincz.** | MH560989* |  |  | JX983675 |
| ***L. rigualii* M.B.Crespo & Erben** | LCFCrigua1 | GQ248628 | AM889717 | AJ222854 |
| ***L. roridum* (Sibth. & Sm.) Brullo & Guarino** | MH561013* | MH582774* | MH582871* | MH582556* |
| ***L. santapolense* Erben** | LCFCsanta1 | LCFCsanta2 | MH582934* |  |
| ***L. saracinatum* R.Artelari** | MH561067* | MH582692* | MH582949* | MH582600* |
| ***L. sarcophyllum* Ghaz. & J.R.Edm.** | MH560997* | MH582760* | MH582884* | MH582523* |
| ***L. scabrum* (Thunb.) Kuntze** | MH561068* | AM235050 | MH582942* | MH582590* |
| ***L. scopulorum* M.B.Crespo & Lledó** | LCFCscopu1 | MH582695* | MH582969* |  |
| ***L. sieberi* (Boiss.) Kuntze** | MH561099* | MH582723* | MH582935* | MH582611* |
| ***L. sinense* (Girard) Kuntze** | LCFCsinen1 | JQ946306 | JQ946307 |  |
| ***L. sinuatum* (L.) Mill.** | AJ391329 | Y16900 | MH582992* | AJ222860 |
| ***L. sitiacum* Rech.f.** | MH561030* | MH582783* | MH582877* |  |
| ***L. sogdianum* Ikonn.-Gal.** |  |  |  | JX983723 |
| ***L. sokotranum* (Vierh.) Radcl.-Sm.** | MH560993* | MH582758* | MH582885* | MH582524* |
| ***L. somalorum* (Vierh.) Hutch. & E.A.Bruce** | MH560994* | MH582761* | MH582886* | MH582525* |
| ***L. sougiae* Erben & Brullo** | MH561014* | MH582782* |  | MH582553* |
| **L. sp.1** | MH561109* | MH582790* | MH582979* | MH582519* |
| **L. sp.2** | MH561052* | MH582687* | MH582901* |  |
| ***L. spectabile* (Svent.) G.Kunkel & Sunding** |  |  |  | MH582641* |
| ***L. spreitzenhoferi* Erben & Brullo** | MH561022* | MH582775* | MH582873* | MH582563* |
| ***L. stenotatum* (Rech.f.) Erben & Brullo** | MH561019* | MH582786* | MH582860* | MH582559* |
| ***L. stocksii* (Boiss.) Kuntze** | LCFCstock1 | LCFCstock2 |  |  |
| ***L. subglabrum* Erben** | MH561045* | MH582711* | MH582967* | MH582596* |
| ***L. suffruticosum* (L.) Kuntze** | MH560995* | MH582728* |  | JX983671 |
| ***L. supinum* (Girard) Pignatti** | MH561096* | MH582697* | MH582943* |  |
| ***L. sventenii* A. Santos & M.L.Fernández** | LCFCsvent1 | LCFCsvent2 |  | MH582637* |
| ***L. tabernense* Erben** | MH561046* | MH582714* |  | MH582566* |
| ***L. tenellum* (Turcz.) Kuntze** | AJ391331 | AJ286365 | MH582974* | MH582516* |
| ***L. tetragonum* (Thunb.) Bullock** | LCFCtetra1 | MH582797* | MH582975* | AB190856/AB190857 |
| ***L. thiniense* Erben** |  | GQ248629 | AM889718 |  |
| ***L. toletanum* Erben** | MH561094* | MH582726* | MH582952* | MH582595* |
| ***L. tomentellum* (Boiss.) Kuntze** | MH560979* | MH582744* | MH582848* | MH582543* |
| ***L. tournefortii* (Boiss.) Erben** | MH561095* | MH582706* | MH582951* |  |
| ***L. tuberculatum* (Boiss.) Kuntze** | LCFCtuber1 | LCFCtuber2 | MH582882* | MH582619* |
| ***L. tubiflorum* (Del.) Kuntze** | MH561033* |  |  | MH582624* |
| ***L. tunetanum* (Barratte) Maire** | MH561048* | MH582712* |  | MH582573* |
| ***L. vanandense* Erben & Brullo** | MH561024* | MH582776* | MH582874* |  |
| ***L. vigaroense* Marrero Rodr. & R.S.Almeida** | MH561126* | MH582813* | MH582997* | MH582638* |
| ***L. virgatum* (Willd.) Fourr.** | MH561054* | KJ608053 | MH582941* | MH582614* |
| ***L. vulgare* Mill.** | MH560982* | MH582735* | JN895287 | MH582538* |
| ***L. wrightii* (Hance) Kuntze** | MH561106* | MH582799* | MH582973* | MH582517* |
| ***L. xerocamposicum* Erben & Brullo** | MH561027* | MH582777* | MH582861* |  |
| ***L. xiliense* Erben & Brullo** | MH561026* | MH582778* | MH582862* | MH582551* |
| **Species of other Plumbaginaceae genera** |  |  |  |  |
| ***Acantholimon acerosum* (Willd.) Boiss.** | AJ391314 |  |  | LT714475 |
| ***Acantholimon bracteatum* (Girard) Boiss.** | LT714372 |  |  | LT714572 |
| ***Acantholimon chitralicum* Rech.f. & Schiman-Czeika** | LT714384 |  |  | LT714584 |
| ***Acantholimon cymosum* Bunge** | LT714410 |  |  | LT714600 |
| ***Acantholimon demavendicum* Bornm.** | LT714295 |  |  | LT714500 |
| ***Acantholimon diapensioides* Boiss.** | LT714386 |  |  | LT714585 |
| ***Acantholimon echinus* (L.) Bunge** | MH561146* | MH582819* |  | MH582646* |
| ***Acantholimon glutinosum* Rech.f. & Köie** | LT714304 |  |  | LT714509 |
| ***Acantholimon gorganense* Mobayen** | LT714305 |  |  | LT714510 |
| ***Acantholimon hohenackeri* (Jaub. & Spach) Boiss.** | MH561148* | MH582816* |  | AB979563 |
| ***Acantholimon leucochlorum* Rech.f. & Schiman-Czeika** | MH561145* | MH582823* |  | LT714527 |
| ***Acantholimon lycopodioides* (Girard) Boiss.** | LT714275 |  | FN597642 | LT714480 |
| ***Acantholimon pterostegium* Bunge** | LT714337 |  |  | LT714541 |
| ***Acantholimon restiaceum* Bunge** | LT714343 |  |  | LT714547 |
| ***Acantholimon revolutum* Rech.f. & Köie** | LT714344 |  |  | LT714548 |
| ***Acantholimon senganense* Bunge** | MH561141* | MH582817* |  | AB979580 |
| ***Acantholimon solidum* Rech.f. & Köie** | LT714350 |  |  | LT714553 |
| ***Acantholimon subulatum* Boiss.** | LT714355 |  |  | LT714557 |
| ***Acantholimon tragacanthinum* (Jaub. & Spach) Boiss.** | MH561147* | MH582821* |  | AB979585 |
| ***Acantholimon tricolor* Rech.f. & Köie** | LT714358 |  |  | LT714559 |
| ***Acantholimon ulicinum* (Schult.) Boiss.** | LT714224 |  |  | LT714429 |
| ***Acantholimon venustum* Boiss.** | MH561142* | MH582822* |  | LT714470 |
| ***Aegialitis annulata* R.Br.** | AJ312245 | AJ312252 |  |  |
| ***Armeria alliacea* (Cav.) Hoffmanns. & Link** | MH561113* | MH582825* | MH582985* | AJ225578 |
| ***Armeria arenaria* (Pers.) Schult.** | MH561118* | KF997272 | MH582987* | MH582653* |
| ***Armeria canescens* (Host) Boiss.** | MH561115* | MH582826* | MH582991* | AY179770 |
| ***Armeria castellana* Boiss. & Reut. ex Leresche** | MH561116* | MH582830* | MH582989* | MH582655* |
| ***Armeria maritima* (Mill.) Willd.** | MH561119* | MH582827* | HM851064 | AJ225574 |
| ***Armeria morisii* Boiss.** | MH561117* | MH582828* | MH582990* | MH582654* |
| ***Armeria pseudarmeria* (Murray) Mansf.** | MH561120* | MH582829* | MH582988* | AJ225596 |
| ***Armeria pungens* (Link) Hoffmanns. & Link** | MH561114* | MH582831* | GQ901553 | MH582656* |
| ***Armeria splendens* (Lag. & Rodr.) Webb** | AJ391316 | Y16908 | MH582986* | AJ225591 |
| ***Bakerolimon plumosum* (F.Phil.) Lincz.** | LCFCBaker1 | MH582824* | MH582983* | MH582657* |
| ***Bukiniczia cabulica* (Boiss.) Lincz.** | MH561144* | MH582818* | MH583013* | MH582645* |
| ***Cephalorhizum coelicolor* (Rech.f.) Rech.f.** | LT714368 | LCFCCepha2 |  | LT714568 |
| ***Ceratolimon feei* (Girard) M.B.Crespo & M.D.Lledó** | AJ391318 | AJ286357 | EU531681 | HE602420 |
| ***Ceratolimon migiurtinum* (Chiov.) M.B.Crespo & M.D.Lledó** | AJ391322 | AJ286360 |  |  |
| ***Ceratolimon weygandiorum* (Maire & Wilczek) M.B.Crespo & M.D.Lledó** | LCFCCerwe1 | AJ286361 |  |  |
| ***Ceratostigma minus* Stapf ex Prain** | AJ391333 |  | AY042566 |  |
| ***Ceratostigma plumbaginoides* Bunge** | MH561150* | MH582838* | MH583014* | MH582659* |
| ***Dictyolimon macrorrhabdos* (Boiss.) Rech.f.** | AJ391317 | Y16909 |  | LT714570 |
| ***Dyerophytum africanum* (Lam.) Kuntze ** | AJ312246 | LCFCDyafr2 | AY042581 | MH582661* |
| ***Dyerophytum indicum* (Gibs. ex Wight) Kuntze** | MH561151* | MH582832* | MH583015* | MH582662* |
| ***Goniolimon besserianum* (Schult.) Kusn.** | MH561140* |  | MH583009* | MH582650* |
| ***Goniolimon incanum* (L.) Hepper** |  | MH582815* | MH583010* | MH582651* |
| ***Goniolimon italicum* Tammaro, Pignatti & G.Frizzi** |  |  | MH583011* | MH582648* |
| ***Goniolimon speciosum* (L.) Boiss.** | AJ312247 | AJ312254 | MH583012* | MH582652* |
| ***Goniolimon tataricum* (L.) Boiss.** | LT714399 |  |  | MH582649* |
| ***Ikonnikovia kaufmanniana* (Regel) Lincz.** | LT714406 | KX527533 | KX526745 | LT714605 |
| ***Limoniastrum guyonianum* Durieu ex Boiss.** | AJ391319 | AJ286358 | MH583008* | HE602418 |
| ***Limoniastrum monopetalum* (L.) Boiss.** | AJ391321 | Z97642 | AY042609 | HE602419 |
| ***Muellerolimon salicorniaceum* (F.Muell.) Lincz** | MH561149* |  | MH582984* |  |
| ***Myriolimon ferulaceum* (L.) Lledó, Erben & M.B.Crespo** | LCFCMyfer1 | KJ608035 |  | MH582658* |
| ***Plumbagella micrantha* (Ledeb.) Spach** | MH561155* | MH582836* | KX526746 | MH582660* |
| ***Plumbago auriculata* Lam.** | JF831319 | EU002283 | MH583017* | MH582665* |
| ***Plumbago caerulea* Kunth** | MH561152* | MH582833* |  | MH582663* |
| ***Plumbago europaea* L.** | AJ391334 | MH582837* | AY042634 | HE602417 |
| ***Plumbago indica* L.** | MH561154* | MH582835* |  | MH582666* |
| ***Plumbago zeylanica* L.** | MH561153* | MH582834* | MH583016* | MH582664* |
| ***Popoviolimon turcomanicum* (Popov ex Lincz.) Lincz.** |  |  |  | JX983658 |
| ***Psylliostachys suvorovii* (Regel) Roshk.** | AJ391335 | Y16907 | AY042639 | AJ132446 |
| ***Psylliostachys spicata* (Willd.) Nevski** |  |  |  | JX983656 |
| ***Saharanthus ifniensis* (Caball.) M.B.Crespo & M.D.Lledó** | AJ391320 | AJ286359 |  |  |
| ***Vassilczenkoa sogdiana*(Lincz.) Lincz.** | MH561143* | MH582820* |  | MH582647* |
| **Outgroup Polygonaceae species** |  |  |  |  |
| ***Coccoloba diversifolia* Jacq.** |  | HM446781 | HM446674 | HM137431 |
| ***Coccoloba swartzii* Meisn.** |  | AF297150 | KJ012533 | FJ154469 |
| ***Coccoloba uvifera* (L.) L.** | AJ312249 | AF206753 | KJ012536 | GQ206246 |
| ***Eriogonum alatum* Torr.** | JQ352614 | EF437977 |  | FJ154472 |
| ***Fallopia convolvulus* (L.) Á.Löve** | EU024782 | KM360782 | EU749341 | AF040064 |
| ***Fallopia dentatoalata* (F.Schmidt) Holub** | EU024775 | HM357888 | EU024769 | AF040066 |
| ***Fallopia dumetorum* (L.) Holub** | EU024785 | JN890735 | AM503813 | AF040068 |
| ***Muehlenbeckia australis* (Forst.f.) Meisn.** | JF831303 | FM883618 | JF831267 | JF831208 |
| ***Muehlenbeckia axillaris* (Hook.f.) Walp.** | JF831304 |  | AY042617 | JF831209 |
| ***Muehlenbeckia complexa* (A.Cunn.) Meisn.** | JF831305 | HM850184 | HM851072 | AF040076 |
| ***Muehlenbeckia costata* K.L.Wilson & Makinson** | JF831306 |  | JF831269 | JF831210 |
| ***Muehlenbeckia gracillima* Meisn.** | JF831309 |  | JF831237 | JF831213 |
| ***Muehlenbeckia platyclada* (F.Muell.) Meisn.** | JF831311 | JN234979 | JF831239 | AF189738 |
| ***Muehlenbeckia rhyticarya* F.Muell.** | JF831312 |  |  | AF189739 |
| ***Muehlenbeckia tiliifolia* Wedd.** | JF831314 |  | JF831270 | JF831215 |
| ***Muehlenbeckia tuggeranong* Mallinson** | JF831315 |  | JF831271 | JF831216 |
| ***Muehlenbeckia volcanica* (Benth.) Endl.** | JF831317 |  | JF831241 | JF831218 |
| ***Neomillspaughia emarginata* (Gross) S.F.Blake** |  |  |  | GQ206257 |
| ***Podopterus cordifolius* Rose & Standl.** |  | FJ154455 | FJ154494 | FJ154479 |
| ***Triplaris americana* L.** | AJ312251 | Y16910 | AY042668 | FJ154486 |

**Table S1** Geographical distributions of *Limonium* species sampled in the current study.

| ***Limonium* species** | **Distributions** |
| --- | --- |
| *L.* cf. *aegaeum* Erben & Brullo | Greece, Turkey |
| *L. albomarginatum* Brullo | Greece |
| *L. algarvense* Erben | Baleares, Spain, Portugal, Morocco |
| *L. ammophilon* (Papatsou & Phitos) Domina | Greece |
| *L. amopicum* Erben & Brullo | Greece |
| *L. anatolicum* Hedge | Turkey |
| *L. anthericoides* (Schltr.) R.A.Dyer | South Africa |
| *L. aphroditae* R.Artelari & Georgiou | Greece |
| *L. aragonense* (Debeaux ex Willk.) Pignatti | Spain |
| *L. arboreum* (Willd.) Erben, A.Santos & Reyes-Bet. | Canary Islands |
| *L. archaeothirae* Erben & Brullo | Greece |
| *L. articulatum* (Loisel.) Kuntze | Corsica, Sardinia, Italy |
| *L. asparagoides* (Batt.) Maire | Algeria, Morocco |
| *L. aucheri* (Girard) Greuter & Burdet | Greece, Turkey |
| *L. aureum* (L.) Hill ex Kuntze | C-Siberia, China, Mongolia |
| *L. auriculae-ursifolium* (Pourr.) Druce | France, Spain, Portugal, Morocco, Algeria |
| *L. axillare* (Forssk.) Kuntze | Bahrain, Egypt, Kuwait, Oman, Saudi Arabia, Sinai peninsula, United Arab Emirates, Qatar, Yemen, Pakistan, Somalia, Sudan, Eritrea |
| *L. beaumierianum* (Coss. ex Maire) Maire | Algeria, Morocco, W-Sahara |
| *L. bellidifolium* (Gouan) Dumort. | Turkey, Cyprus, Iran, Spain, France, Corsica, Sardinia, Italy, Croatia, Greece, Montenegro, Tunisia, E-England, Romania, Moldavia, European Russia, Crimea, W-Siberia, Kazakhstan, Northern Caucasus |
| *L. benmageci* Marrero Rodr. | Canary Islands |
| *L. biflorum* (Pignatti) Pignatti | Baleares |
| *L. binervosum* (G.E.Sm.) C.E.Salmon | England, France, Ireland, Spain, Morocco, Channel Islands |
| *L. bocconei* (Lojac.) Litard. | Sicily, Isole Egadi |
| *L. bollei* (Webb ex Wangerin) Erben | Canary Islands |
| *L. bonduellei* (T.Lestib.) Kuntze | Algeria, Morocco, Tunisia, Libya, Mauritania, Chad, Egypt |
| *L. bonifaciense* Arrigoni & Diana | Corsica |
| *L. bourgeaui* (Webb ex Webb) Kuntze | Canary Islands |
| *L. brasiliense* (Boiss.) Kuntze | Argentina, Brazil, Uruguay |
| *L. brassicifolium* (Webb & Berthel.) Kuntze | Canary Islands |
| *L. braunii* (Bolle) A.Chev. | Cape Verde Islands |
| *L. brevipetiolatum* R.Artelari & Erben | Greece |
| *L. brunneri* (Webb. ex Boiss.) Kuntze | Cape Verde Islands |
| *L. caesium* (Girard) Kuntze | Spain |
| *L. californicum* (Boiss.) A.Heller | USA, Mexico |
| *L. calliopsium* Alf.Mayer | Greece |
| *L. camposanum* M. Erben | Baleares |
| *L. cancellatum* (Bertol.) Kuntze | Albania, Croatia, Italy, Montenegro |
| *L. capense* (L.Bolus) L.Bolus | South Africa |
| *L. carnosum* (Boiss.) Kuntze | Azerbaijan, Iran, Kuwait |
| *L. carolinianum* (Walter) Britton | Canada, USA, Bermuda |
| *L. carpathum* (Rech.f.) Rech.f. | Greece |
| *L. carpetanicum* Erben | Spain |
| *L. carthaginense* (Rouy) C.E.Hubb. & Sandwith | Spain |
| *L. cephalonicum* R.Artelari | Greece |
| *L. chersonesum* Erben & Brullo | Greece |
| *L. circaei* Pignatti | Italy |
| *L. compactum* Erben & Brullo | Greece |
| *L. confusum* (Godr. & Gren.) Fourr. | Baleares, Corsica, France, Italy, Sardinia |
| *L. contortirameum* (Mabille) Erben | Corsica |
| *L. cordatum* (L.) Mill. | France, Italy |
| *L. cornarianum* Kypr. & R.Artelari | Greece |
| *L. coronense* R.Artelari | Greece |
| *L. corsicum*Erben | Corsica |
| *L. cossonianum* Kuntze | Baleares, Spain, Morocco, Algeria |
| *L. costae* (Willk.) Pignatti | Spain |
| *L. cosyrense* (Guss.) Kuntze | Malta, Pantelleria |
| *L. crateriforme* Erben & Brullo | Greece |
| *L. creticum* R. Artelari | Greece |
| *L. cylindrifolium* (Forssk.) Verdc. ex Cufod. | Saudi Arabia, Yemen, NE-trop. Africa |
| *L. cymuliferum* (Boiss.) Sauvage & Vindt | Algeria, Morocco |
| *L. cythereum* R.Artelari & Georgiou | Greece |
| *L. daveaui* Erben | Portugal |
| *L. delicatulum* (Girard) Kuntze | Spain, Tunisia, Algeria, Morocco, Libya |
| *L. dendroides* Svent. | Canary Islands |
| *L. densissimum* (Pignatti) Pignatti | Spain, France, Sicily, Italy, ?Sardinia |
| *L. dichotomum* (Cav.) Kuntze | Spain |
| *L. dichroanthum* (Rupr.) Ikonn.-Gal. | China, Kyrgyzstan, Kazakhstan |
| *L. dodartii* (Girard) Kuntze | Portugal, Spain, France |
| *L. dufourii* (Girard) Kuntze | Spain |
| *L. ebusitanum* (Font Quer) Font Quer | Baleares |
| *L. echioides* (L.) Mill. | Portugal, Spain, Baleares, France, Corsica, Sardinia, Sicily, Italy, Greece, Libya, Tunisia, Algeria, Morocco, Turkey, Cyprus, Egypt |
| *L. effusum* (Boiss.) Kuntze | Turkey |
| *L. elaphonisicum* Alf.Mayer | Greece |
| *L. erectum* Erben | Spain |
| *L. estevei* Fern.Casas | Spain |
| *L. fallax* (Coss. ex Wangerin) Maire | Morocco |
| *L. flexuosum* (L.) Kuntze | C-Siberia, China, Mongolia |
| *L. frederici* (Barbey) Rech.f. | Greece |
| *L. frutescens* (Lem.) Erben, A.Santos & Reyes-Bet. | Canary Islands |
| *L. furfuraceum* (Lag.) Kuntze | Spain |
| *L. girardianum* (Guss.) Fourr. | France, Spain, Baleares |
| *L. globuliferum* (Boiss. & Heldr.) Kuntze | Turkey, Syria |
| *L. gmelini* (Willd.) Kuntze | Hungary, Romania, Macedonia, Montenegro, Serbia & Kosovo, Bulgaria, Crimea, C- & E-European Russia, Ukraine, Moldavia, Siberia, China, Kazakhstan, Kyrgyzstan, Mongolia, Turkey, Iran |
| *L. gougetianum* (Girard) Kuntze | Tunisia, Algeria, Baleares |
| *L. grabusae* Erben & Brullo | Greece |
| *L. graecum* (Poir.) Rech.f. | Greece, Turkey |
| *L. greuteri* Erben | Corsica |
| *L. guaicuru* (Molina) Kuntze | Chile |
| *L. gymnesicum* Erben | Baleares |
| *L. hibericum* Erben | Spain |
| *L. hierapetrae* Rech.f. | Greece |
| *L. hoeltzeri* (Regel) Ikonn.-Gal. | Kyrgyzstan |
| *L. humile* Mill. | England, Denmark, France, Germany, Ireland, Norway, Sweden, Spain, Portugal |
| *L. hungaricum* Klokov | Hungary, Romania, Slovakia |
| *L. hyblaeum* Brullo | Sicily |
| *L. iconicum* (Boiss. & Heldr.) Kuntze | Turkey |
| *L. imbricatum* (Webb ex Girard) Hubbard ex L.H.Bailey | Canary Islands |
| *L. insigne* (Coss.) Kuntze | Spain |
| *L. iranicum* (Bornm.) Lincz. | Iran, Iraq |
| *L. jovi-barba* (Webb) Kuntze | Cape Verde Islands |
| *L. kardamylii* R.Artelari & Kamari | Greece |
| *L. kaschgaricum* (Rupr.) Ikonn.-Gal. | China, Kyrgyzstan |
| *L. kraussianum* (Buchinger ex Boiss.) Kuntze | South Africa |
| *L. lanceolatum* (Hoffmanns. & Link) Franco | South Africa |
| *L. latebracteatum* Erben | Spain |
| *L. latifolium* (Sm.) Kuntze | Bulgaria, Romania, Ukraine |
| *L. laxiusculum* Franco | Portugal |
| *L. lilacinum* (Boiss. & Bal.) Wagenitz | Turkey |
| *L. limbatum* Small | USA, Mexico |
| *L. lobatum* (L.f.) Chaz. | Spain, Greece, Libya, Tunisia, Algeria, Morocco, NW-Sahara, Israel, Egypt, Iran, Iraq, Jordania, Kuwait, Saudi Arabia, Sinai peninsula, Syria, Canary Islands |
| *L. lobinii* N.Kilian & Leyens | Cape Verde Islands |
| *L. longebracteatum* Erben | Spain |
| *L. lowei* R.Jardim, M.Seq., Capelo, J.C.Costa & Rivas Mart. | Madeira |
| *L. macrophyllum* Kuntze | Canary Islands |
| *L. macropterum* (Webb & Berthel.) Kuntze | Canary Islands |
| *L. majus* (Boiss.) Erben | Spain |
| *L. meandrinum* Erben & Brullo | Greece |
| *L. meyeri* (Boiss.) Kuntze | Bulgaria, Crimea, E-European Russia, Armenia, Georgia, Azerbaijan, Turkmenistan, Ukraine, Uzbekistan, Turkey, Cyprus, Iran, Israel |
| *L. milleri* Ghaz. & J.R.Edm. | Oman |
| *L. minoicum* Erben & Brullo | Greece |
| *L. minutiflorum* (Guss.) Kuntze | Sicily |
| *L. minutum* (L.) Chaz. | Baleares |
| *L. mouretii* (Pitard) Maire | Morocco |
| *L. mucronatum* (L.f.) Chaz. | Morocco |
| *L. multiflorum* Erben | Portugal |
| *L. multiforme* (Martelli) Pignatti | Italy |
| *L. narbonense* Mill. | Turkey, Cyprus, Egypt, Lebanon, Sinai peninsula, Syria, Spain, France, Corsica, Sardinia, Sicily, Italy, Slovenia, Croatia, Albania, Bulgaria, Greece, Tunisia, Algeria, Morocco, Portugal, Azores? |
| *L. nudum* (Boiss. & Buhse) Kuntze | Iran |
| *L. nydeggeri* Erben | Portugal |
| *L. obtusifolium* (Rouy) Erben | Corsica |
| *L. ocymifolium* (Poir.) Kuntze | Greece |
| *L. oligotrichum* Erben & Brullo | Greece |
| *L. otolepis* (Schrenk) Kuntze | China, Afghanistan, Kazakhstan, Kyrgyzstan, Tajikistan, Uzbekistan, Turkmenistan |
| *L. ovalifolium* (Poir.) Kuntze | France, Spain, Portugal, Morocco |
| *L. palmyrense* (Post) Dinsm. | Jordania, Syria |
| *L. papillatum* (Webb & Berthel.) Kuntze | Islas Selvagens, Canary Islands |
| *L. parvibracteatum* Pignatti | Spain |
| *L. paulayanum* (Vierh.) Ghaz. & J.R.Edm. | Socotra |
| *L. pectinatum* var. *corculum* (Webb & Berthel.) G.Kunkel & Sunding | Canary Islands |
| *L. pectinatum* var. *divaricatum* (Pit.) G.Kunkel & Sunding | Canary Islands |
| *L. pectinatum* var. *solandri* (Webb. & Berthel.) Kuntze | Canary Islands |
| *L. peregrinum* (P.J.Bergius) R.A.Dyer | South Africa |
| *L. perezii* (Stapf) Hubbard ex L.H.Bailey | Canary Islands |
| *L. perfoliatum* (Kar. ex Boiss.) Kuntze | Afghanistan, Iran, Turkmenistan, Uzbekistan |
| *L. pigadiense* (Rech.f.) Rech.f. | Greece |
| *L. platyphyllum* Lincz. | Moldova, European Russia, Ukraine |
| *L. plurisquamatum* Erben | Portugal |
| *L. preauxii* (Webb & Berthel.) Kuntze | Canary Islands |
| *L. proliferum* (d'Urv.) Erben & Brullo | Greece |
| *L. pruinosum* (L.) Chaz. | Israel, Egypt, Jordania, Kuwait, Saudi Arabia, Sinai peninsula, Libya, Tunisia, Algeria, Morocco |
| *L. pseudebusitanum* Erben | Baleares |
| *L. puberulum* (Webb) Kuntze | Canary Islands |
| *L. purpuratum* Hubbard ex L.H.Bailey | South Africa |
| *L.* cf. *pycnanthum* (K. Koch) Kuntze | Turkey |
| *L. pylium* R.Artelari | Greece |
| *L. recticaule* Erben & Brullo | Greece |
| *L. recurvum* C.E.Salmon subsp. *humile* (Girard) Ingr. | United Kingdom |
| *L. redivivum* (Svent.) G.Kunkel & Sunding | Canary Islands |
| *L. relicticum* R.Mesa & A.Santos | Canary Islands |
| *L. remotispiculum* (Lacaita) Pignatti | Italy |
| *L. reniforme* (Girard) Lincz. | Iran |
| *L. rigualii* M.B.Crespo & Erben | Spain |
| *L. roridum* (Sibth. & Sm.) Brullo & Guarino | Greece |
| *L. santapolense* Erben | Spain |
| *L. saracinatum* R.Artelari | Greece |
| *L. sarcophyllum* Ghaz. & J.R.Edm. | Oman |
| *L. scabrum* (Thunb.) Kuntze | South Africa, Namimbia |
| *L. scopulorum* M.B.Crespo & Lledó | Spain |
| *L. sieberi* (Boiss.) Kuntze | Greece, Turkey, Lebanon, Syria |
| *L. sinense* (Girard) Kuntze | China, Taiwan, Ryukyu Islands |
| *L. sinuatum* (L.) Mill. | Moldavia, European Russia, Crimea, Georgia, Portugal, Spain, Gibraltar, France, Corsica, Sardinia, Sicily, Italy, Serbia & Kosovo, Montenegro, Albania, Greece, Slovakia, Turkey, Cyprus, Egypt, Israel, Lebanon, Sinai peninsula, Syria, Canary Islands |
| *L. sitiacum* Rech.f. | Greece |
| *L. sogdianum*Ikonn.-Gal. | Kazakhstan, Turkmenistan, Tajikistan, Uzbekistan |
| *L. sokotranum* (Vierh.) Radcl.-Sm. | Socotra, Samha Island, Abd-al-Kuri Island |
| *L. somalorum* (Vierh.) Hutch. & E.A.Bruce | Somalia |
| *L. sougiae* Erben & Brullo | Greece |
| L. sp.1 | Turkey |
| L. sp.2 | Greece |
| *L. spectabile* (Svent.) G.Kunkel & Sunding | Canary Islands |
| *L. spreitzenhoferi* Erben & Brullo | Greece |
| *L. stenotatum* (Rech.f.) Erben & Brullo | Greece |
| *L. stocksii* (Boiss.) Kuntze | Iran, India, Pakistan |
| *L. subglabrum* Erben | Spain |
| *L. suffruticosum* (L.) Kuntze | SE-European Russia, Kazakhstan, Iran, Afganistan, Mongolia, W-Siberia, China, Crimea, Ukraine, Kyrgyzstan, Uzbekistan, Azerbaijan, Northern Caucasus, Turkmenistan, Tajikistan |
| *L. supinum* (Girard) Pignatti | Spain |
| *L. sventenii* A. Santos & M.L.Fernández | Canary Islands |
| *L. tabernense* Erben | Spain |
| *L. tenellum* (Turcz.) Kuntze | China, Mongolia |
| *L. tetragonum* (Thunb.) Bullock | New Caledonia, South Korea, North Korea, Japan, Ryukyu Islands |
| *L. thiniense* Erben | Spain |
| *L. toletanum* Erben | Spain |
| *L. tomentellum*(Boiss.) Kuntze | Crimea, Romania, Ukraine, E-European Russia, Northern Caucasus |
| *L. tournefortii* (Boiss.) Erben | Spain |
| *L. tuberculatum* (Boiss.) Kuntze | Canary Island, Morocco, W-Sahara, Mauritania |
| *L. tubiflorum* (Del.) Kuntze | Libya, Egypt |
| *L. tunetanum* (Barratte) Maire | Libya, Tunisia, Algeria |
| *L. vanandense* Erben & Brullo | Greece |
| *L. vigaroense* Marrero Rodr. & R.S.Almeida | Canary Islands |
| *L. virgatum* (Willd.) Fourr. | Portugal, Spain, Baleares, France, Corsica, Sardinia, Malta, Sicily, Italy, Croatia, Albania, Greece, Libya, Tunisia, Algeria, Turkey, Cyprus, Israel, Lebanon, Syria |
| *L. vulgare* Mill. | Belgium, England, Denmark, Germany, Netherlands, Romania, Sweden, Portugal, Spain, France, Azores |
| *L. wrightii* (Hance) Kuntze | Taiwan, Japan, Ryukyu Islans, Bonin Islands |
| *L. xerocamposicum* Erben & Brullo | Greece |
| *L. xiliense* Erben & Brullo | Greece |

Table S2. *Limonium* species that have not been assigned to any broad infrageneric classification in previous studies.

| *L.* cf. *aegaeum* Erben & Brullo | *L. meandrinum* Erben & Brullo |
| --- | --- |
| *L. albomarginatum* Brullo | *L. milleri* Ghaz. & J.R.Edm. |
| *L. ammophilon* (Papatsou & Phitos) Domina | *L. minoicum* Erben & Brullo |
| *L. amopicum* Erben & Brullo | *L. nydeggeri* Erben |
| *L. anthericoides* (Schltr.) R.A.Dyer | *L. obtusifolium* (Rouy) Erben |
| *L. aphroditae* R.Artelari & Georgiou | *L. oligotrichum* Erben & Brullo |
| *L. archaeothirae* Erben & Brullo | *L. paulayanum* (Vierh.) Ghaz. & J.R.Edm. |
| *L. bollei* (Webb ex Wangerin) Erben | *L. pigadiense* (Rech.f.) Rech.f. |
| *L. bonifaciense* Arrigoni & Diana | *L. platyphyllum* Lincz. |
| *L. brevipetiolatum* R.Artelari & Erben | *L. proliferum* (d’Urv.) Erben & Brullo |
| *L. calliopsium* Alf.Mayer | *L. pseudebusitanum* Erben |
| *L. carpetanicum* Erben | *L. pylium* R.Artelari |
| *L. carthaginense* (Rouy) C.E.Hubb. & Sandwith | *L. recticaule* Erben & Brullo |
| *L. cephalonicum* R.Artelari | *L. santapolense* Erben |
| *L. chersonesum* Erben & Brullo | *L. saracinatum* R.Artelari |
| *L. circaei* Pignatti | *L. scopulorum* M.B.Crespo & Lledó |
| *L. compactum* Erben & Brullo | *L. sitiacum* Rech.f. |
| *L. contortirameum* (Mabille) Erben | *L. sokotranum* (Vierh.) Radcl.-Sm. |
| *L. cornarianum* Kypr. & R.Artelari | *L. sougiae* Erben & Brullo |
| *L. coronense* R.Artelari | *L. spreitzenhoferi* Erben & Brullo |
| *L. corsicum* Erben | *L. stenotatum* (Rech.f.) Erben & Brullo |
| *L. crateriforme* Erben & Brullo | *L. subglabrum* Erben |
| *L. creticum* R.Artelari | *L. tabernense* Erben |
| *L. cythereum* R.Artelari & Georgiou | *L. thiniense* Erben |
| *L. daveaui* Erben | *L. toletanum* Erben |
| *L. ebusitanum* (Font Quer) Font Quer | *L. tunetanum* (Barratte) Maire |
| *L. elaphonisicum* Alf.Mayer | *L. vanandense* Erben & Brullo |
| *L. erectum* Erben | *L. xerocamposicum* Erben & Brullo |
| *L. estevei* Fern.Casas | *L. xiliense* Erben & Brullo |
| *L. grabusae* Erben & Brullo | *L.* sp.1 |
| *L. greuteri* Erben | *L.* sp.2 |
| *L. hibericum* Erben |  |
| *L. hierapetrae* Rech.f. |  |
| *L. hyblaeum* Brullo |  |
| *L. kardamylii* R.Artelari & Kamari |  |
| *L. lanceolatum* (Hoffmanns. & Link) Franco |  |
| *L. latebracteatum* Erben |  |
| *L. laxiusculum* Franco |  |
| *L. longebracteatum* Erben |  |
| *L. lowei* R.Jardim, M.Seq., Capelo, J.C.Costa & Rivas Mart. |  |
| *L. majus* (Boiss.) Erben |  |

**Table S3.** List of *Limonium* species in alphabetical order (compiled by reviewing online databases, floras and published studies), their assignment to clades in the molecular phylogeny presented here (Figures 2 and 3), and justification for the phylogenetic assignment of species that were not directly included in the molecular phylogeny (in boldface) based on the reviewed literature.

*‘Mediterranean lineage’ = 'Mediterranean lineage' excluding *L.* sect. *Schizhymenium,* *L.* sect. *Siphonantha,* *L.* sect. *Polyarthrion* and *L*. sect. *Pruinosum*

| **Species of *Limonium*** | **Assignment of species to clades in the present phylogeny and corresponding taxonomic units** | **Justification for assignment of species to clades** | **References** |
| --- | --- | --- | --- |
| ***Limonium acuminatum* L. Bolus** | ‘Mediterranean lineage’* in *Limonium* subg*. Limonium* | The species has morphological affinities with *L. scabrum* (Figure 3, 'Mediterranean lineage'), *L. decumbens* and *L. equisetinum,* all of them assigned to *L.* sect. *Limonium* subsect. *Steirocladae* by Boissier. Representatives of this subsection sampled for the molecular phylogeny are all placed in the 'Mediterranean lineage' (Figure 3). | Dyer (1963) |
| ***Limonium acutifolium* (Rchb.) C.E.Salmon** | ‘Mediterranean lineage’* in *Limonium* subg. *Limonium* | The species is endemic to Corse and Sardinia, and shows morphological similarities to other Mediterranean endemics, such as *L. bonifaciense* and *L. obtusifolium* which are both placed in the Mediterranean lineage' (Figure 3). | Pignatti (1972), Arrigoni & Diana (1999) and Guarino *et al.* (2017) |
| ***Limonium adilguneri* Yıld. & Doğru-Koca** | *L.* sect. *Nephrophyllum s.l.* in *Limonium* subg. *Limonium* | The species shares morphological similarities with *L. bellidifolium* and *L. iconicum,* which are placed in the clade of *L.* sect. *Nephrophyllum s.l.* (see also Figure 2). | Yıldırımlı (2006) |
| ***Limonium admirabile* Terrones, J.Moreno, M.A.Alonso, Juan & M.B.Crespo** | ‘Mediterranean lineage’* in *Limonium* subg*. Limonium* | The species is morphologically similar to *L. latebracteatum* and *L. carpetanicum,* which are both placed in the 'Mediterranean lineage' (Figure 3). | Moreno *et al.* (2018) |
| *Limonium aegaeum* Erben & Brullo | ‘Mediterranean lineage’* in *Limonium* subg. *Limonium* | see Figure 3 |  |
| ***Limonium aegusae* Brullo** | ‘Mediterranean lineage’* in *Limonium* subg*. Limonium* | The species is part of *L. densissimum* aggregate and thus it is morphologically similar to *L. densissimum,* which is placed in the 'Mediterranean lineage'. | Pignatti (1982a) |
| ***Limonium afghanicum* Erben & Podlech** | *L.* sect. *Nephrophyllum s.l.* in *Limonium* subg. *Limonium* | The species is morphologically similar to *L*. *iconicum,* which belongs to *L.* sect. *Nephrophyllum s.l.* (see Figure 2). | Erben (1980) |
| ***Limonium afrum* (Pignatti) Domina** | ‘Mediterranean lineage’* in *Limonium* subg*. Limonium* | The species is morphologically similar to *L. delicatulum* ('Mediterranean lineage'; Figure 3) and was previously classified as subspecies of it (i.e. *L. delicatulum* subsp. a*frum* Pignatti). | Greuter & Raab-Straube (2011) |
| ***Limonium albarracinense* Pau ex Ferrer & Roselló** | ‘Mediterranean lineage’* in *Limonium* subg*. Limonium* | The species is morphologically similar to *L. ruizii,* *L. costae* and *L. longebracteatum. Limonium costae* and *L. longebracteatum* are placed in the 'Mediterranean lineage' (Figure 3). | Ferrer-Gallego *et al.* (2018) |
| ***Limonium albidum* (Guss.) Pignatti** | ‘Mediterranean lineage’* in *Limonium* subg*. Limonium* | The species is morphologically similar to other Mediterranean species of *L. albidum* group, such as *L. hyblaeum* which is placed in the 'Mediterranean lineage' (Figure 3). | Brullo & Panone (1981), Pignatti (1982a) and Domina & Mazzola (2003) |
| *Limonium albomarginatum* Brullo | ‘Mediterranean lineage’* in *Limonium* subg*. Limonium* | see Figure 3 |  |
| ***Limonium albuferae* P.P.Ferrer, R.Roselló, M.Rosato, Rosselló & E.Laguna** | ‘Mediterranean lineage’* in *Limonium* subg*. Limonium* | This is a relatively newly described polyploid species from the Eastern Iberian pensinsula that is morphologically similar to *L. girardianum, L. scopulorum* and *L. auriculae-ursifolium,* all of which are placed in the 'Mediterranean lineage'm (Figure 3). | Ferrer-Gallego *et al.* (2016) |
| ***Limonium album* (Coincy) Sennen** | ‘Mediterranean lineage’* in *Limonium* subg*. Limonium* | The species shows morphological similarities with other Mediterranean species belonging to *L. delicatulum* group, such as *L. delicatulum* and *L. supinum* that are placed in the 'Mediterranean lineage' (Figure 3). | Erben (1993) |
| ***Limonium alcudianum* Erben** | ‘Mediterranean lineage’* in *Limonium* subg*. Limonium* | The species is morhologically similar to *L. gymnesicum,* which is placed in the 'Mediterranean lineage' (Figure 3). | Erben (1989) |
| *Limonium algarvense* Erben | ‘Mediterranean lineage’* in *Limonium* subg*. Limonium* | see Figure 3 |  |
| ***Limonium algusae* (Brullo) Greuter** | ‘Mediterranean lineage’* in *Limonium* subg*. Limonium* | The species is morphologically close to *L. virgatum* (*L. virgatum* aggregate) and was formerly a subspecies of *L. virgatum* under the former species name (*L. oleifolium*). *Limonium virgatum* is placed in the 'Mediterranean lineage' (Figure 3). | Brullo & Panone (1981), Greuter *et al.* (1989), Domina & Mazzola (2003) and Guarino *et al.* (2017) |
| ***Limonium alicunense*F.Gómiz García** | ‘Mediterranean lineage’* in *Limonium* subg*. Limonium* | This species is a narrow endemic of Spain and shows morphological similarities to other Mediterranean species, such as *L. supinum* and *L. parvibracteatum*, which are both placed in the 'Mediterranean lineage' (Figure 3). | Gómiz (1995) |
| ***Limonium alleizettei* (Pau) Brullo & Erben** | *L.* sect. *Pruinosum* in *Limonium* subg. *Limonium* | The species is morphologically close to *L. pruinosum,* which is placed in the clade of *L.* sect. *Pruinosum* (see also Figure 3) and was formerly a subspecies of it (*Limonium pruinosum* subsp. *alleizettei* (Pau) Maire). | Fennane & Tattou (2005) |
| ***Limonium alutaceum* (Stev.) Kuntze** | *L.* sect. *Limonium* in *Limonium* subg. *Limonium* | The species is morphologically similar to *L. tomentellum,* which is placed in the re-circumscribed *L.* sect. *Limonium* (Figure 2). | Pignatti (1972) |
| *Limonium ammophilon (*Papatsou & Phitos) Domina | ‘Mediterranean lineage’* in *Limonium* subg*. Limonium* | see Figure 3 |  |
| ***Limonium amoenum* (C.H.Wright) R.A.Dyer** | *L.* sect. *Circinaria* in *Limonium* subg. *Limonium* | The species was previously assigned to genus *Afrolimon,* which is currently embeded in *Limonium.* All represenatives of *Afrolimon* currently comprise *L.* sect. *Circinaria* (see represenatives in Figure 2). | Malekmohammadi et al. (2017) |
| *Limonium amopicum* Erben & Brullo | ‘Mediterranean lineage’* in *Limonium* subg*. Limonium* | see Figure 3 |  |
| ***Limonium ampuriense* Arrigoni & Diana** | ‘Mediterranean lineage’* in *Limonium* subg*. Limonium* | The species is a diploid, endemic to Sardinia and shows morphological similarities to other Mediterranean endemics, such as *L. articulatum*, which is placed in the 'Mediterranean lineage' (Figure 3). | Mayer (1995) and Arrigoni & Diana (1999) |
| ***Limonium amynclaeum* Pignatti** | ‘Mediterranean lineage’* in *Limonium* subg*. Limonium* | The species is morphologically similar to other Mediterranean species, such as *L. multiforme* (*L. multiforme* aggregate-*L.cosyrense* group) which is placed in the 'Mediterranean lineage' (Figure 3). | Pignatti (1982a,b) |
| *Limonium anatolicum* Hedge | *L.* sect. *Iranolimon* in *Limonium* subg. *Limonium* | see Figure 2 |  |
| ***Limonium angustebracteatum* Erben** | ‘Mediterranean lineage’* in *Limonium* subg*. Limonium* | The species is morphologically similar to *L. delicatulum* and it was previously a subspecies of it (*Limonium delicatulum* subsp. *angustebracteatum* (Erben) Rivas Martinez & Costa). *Limonium delicatulum* and *L. angustebractetum* were assigned to *L.* sect. *Limonium* subsect. *Dissitoflorae* by Boissier and to *L. delicatulum* complex*.* Representatives of this subsection and complex are all part of the 'Mediterranean lineage' in the phylogeny (Figure 3). | Pignatti (1972), Erben (1978) and Palacios *et al.* (2000) |
| *Limonium anthericoides*(Schltr.) R.A.Dyer | *L.* sect. *Tenuiramosum* in *Limonium* subg. *Pteroclados s.l.* | see Figure 2 |  |
| ***Limonium antipaxorum* R.Artelari** | ‘Mediterranean lineage’* in *Limonium* subg*. Limonium* | The species is morphologically similar to other Mediterranean species, especially from the Ionian-Adriatic region, such as *L. cephalonicum, L. saracinatum* and *L. pylium,* which are all placed in the 'Mediterranean lineage' (Figure 3). | Artelari & Kamari (1986) |
| ***Limonium antonii-llorensii* L.Llorens** | ‘Mediterranean lineage’* in *Limonium* subg*. Limonium* | The species is morhologically similar to other Mediterranean species, such *L. biflorum* (*L. delicatulum* group), that it is placed in the 'Mediterranean lineage' (Figure 3). | Crespo (2009) |
| *Limonium aphroditae* R.Artelari & Georgiou | ‘Mediterranean lineage’* in *Limonium* subg*. Limonium* | see Figure 3 |  |
| ***Limonium apulum* Brullo** | ‘Mediterranean lineage’* in *Limonium* subg*. Limonium* | The species is morphologically similar to other Mediterranean species especially from the Adriatic region, such as *L. cancellatum,* which is placed in the 'Mediterranean lineage' (Figure 3). | Bogdanović & Brullo (2015) and https://botany.cz/cs/limonium-apulum/ |
| *Limonium aragonense* (Debeaux ex Willk.) Pignatti | ‘Mediterranean lineage’* in *Limonium* subg*. Limonium* | see Figure 3 |  |
| *Limonium arboreum* (Willd.) Erben, A.Santos & Reyes-Bet. | *L.* sect. *Pteroclados* subsect. *Nobiles* in *Limonium* subg. *Pteroclados s.l.* | see Figure 2 |  |
| *Limonium archaeothirae* Erben & Brullo | ‘Mediterranean lineage’* in *Limonium* subg*. Limonium* | see Figure 3 |  |
| ***Limonium arcuatum* R.Artelari** | ‘Mediterranean lineage’* in *Limonium* subg*. Limonium* | The species is morphologically similar to other Mediterranean species, especially from the Ionian-Adriatic region, such as *L. cephalonicum, L. saracinatum* and *L. pylium,* which are placed in the 'Mediterranean lineage' (Figure 3). | Artelari & Kamari (1986) |
| ***Limonium arenosum* Erben** | ‘Mediterranean lineage’* in *Limonium* subg*. Limonium* | The species is morphologically similar to *L. angustebracteatum* and *L. supinum*. The latter species is sampled in the phylogeny and is placed in the 'Mediterranean lineage' (Figure 3). | Erben (1978) |
| *Limonium articulatum* (Loisel.) Kuntze | ‘Mediterranean lineage’* in *Limonium* subg*. Limonium* | see Figure 3 |  |
| ***Limonium artruchium* Erben** | ‘Mediterranean lineage’* in *Limonium* subg*. Limonium* | The species is a narrow endemic of Menorca (Baleares islands) and shows morphological similarities to other Mediterranean species from the same region, such as *L. camposanum,* which is placed in the 'Mediterranean lineage' (Figure 3). | Erben (1993) |
| *Limonium asparagoides* (Batt.) Maire | *L.* sect. *Pruinosum* in *Limonium* subg. *Limonium* | see Figure 3 |  |
| ***Limonium asperrimum* Maire** | *L.* sect. *Siphonantha* in *Limonium* subg. *Limonium* | The species has been assigned to *L.* sect. *Siphonantha* (Sauvage & Vindt 1952) and is morphologically similar to *L. tubiflorum* and *L. maroccanum* (see *L. tubiflorum* in Figure 3). | Maire (1938) and Sauvage & Vindt (1952) |
| ***Limonium asterotrichum* C.E.Salmon** | *L.* sect. *Limonium* in *Limonium* subg. *Limonium* | The species is morphologically similar to *L. tomentellum* and *L. latifolium,* which are bith placed in the clade of the re-circumscribed *L.* sect. *Limonium* (Figure 2). | Pignatti (1972) |
| ***Limonium astypaleanum* Erben & Brullo** | ‘Mediterranean lineage’* in *Limonium* subg*. Limonium* | The species is morphologically similar to other Mediterranean species, especially endemics of the Aegean region, such as *L. meandrinum* which is placed in the 'Mediterranean lineage' (Figure 3). | Brullo & Erben (2016) |
| ***Limonium athinense* Erben & Brullo** | ‘Mediterranean lineage’* in *Limonium* subg*. Limonium* | The species is morphologically similar to other Mediterranean species, especially endemics of the Aegean region, such as *L. fragile, L. dolihense* and *L. xerocamposicum. Limonium xerocamposicum* is sampled in this study and is placed in the 'Mediterranean lineage' (Figure 3). | Brullo & Erben (2016) |
| ***Limonium atticum* Erben & Brullo** | ‘Mediterranean lineage’* in *Limonium* subg*. Limonium* | The species is morphologically similar to other Mediterranean species, especially endemics of the Aegean region, such as *L. aucheri* and *L. ocymifolium* which are placed in the 'Mediterranean lineage' (Figure 3). | Brullo & Erben (2016) |
| *Limonium aucheri* (Girard) Greuter & Burdet | ‘Mediterranean lineage’* in *Limonium* subg*. Limonium* | see Figure 3 |  |
| *Limonium aureum* (L.) Hill ex Kuntze | *L.* sect. *Plathymenium* in *Limonium* subg. *Limonium* | see Figure 2 |  |
| *Limonium auriculae-ursifolium* (Pourr.) Druce | ‘Mediterranean lineage’* in *Limonium* subg*. Limonium* | see Figure 3 |  |
| ***Limonium australe* (R.Br.) Kuntze** | *L.* sect. *Plathymenium* in *Limonium* subg. *Limonium* | The species is morphologically and phylogenetically related to other species of *L.* sect. *Plathymenium,* such as *L. flexuosum* and *L. aureum* (see Figure 2 and Malekmohammadi *et al.* 2017). | Malekmohammadi *et al.* (2017) |
| ***Limonium avei* (De Not.) Brullo & Erben** | *L.* sect. *Schizhymenium* in *Limonium* subg. *Limonium* | This annual species is morphologically and phylogenetically related to *L. echioides* of *L.* sect. *Schizhymenium.* | Malekmohammadi *et al.* (2017) |
| *Limonium axillare* (Forssk.) Kuntze | *L.* sect. *Sarcophyllum* in *Limonium* subg. *Limonium* | see Figure 2 |  |
| ***Limonium bahamense* Britton** | *L.* sect. *Ctenostachys* in *Limonium* subg. *Limonium* | The species is morphologically similar to *L. haitiense* which was recently placed in the clade of *L.* sect. *Ctenostachys* in the phylogentic study of Malekmohammadi *et al.* (2017). | Malekmohammadi *et al.* (2017) |
| ***Limonium balearicum* (Pignatti) Brullo** | ‘Mediterranean lineage’* in *Limonium* subg*. Limonium* | The species is morphologically similar to *L. gymnesicum* and *L. minutiflorum*, which are placed in the 'Mediterranean lineage' (Figure 3). *Limonium balearicum* was previously a subspecies of *L. minutiflorum* (*L. minutiflorum* subsp. *balearicum* Pignatti). | Pignatti (1972) and Erben (1993) |
| ***Limonium barceloi* L.Gil & L.Llorens** | ‘Mediterranean lineage’* in *Limonium* subg*. Limonium* | The species is morphologically similar to other Mediterranean species, especially from Baleares and genetically related to *L. cossonianum* and *L. minutum,* which are placed in the 'Mediterranean lineage' (Figure 3). | Rosselló (2008) |
| ***Limonium battandieri* Greuter & Burdet** | ‘Mediterranean lineage’* in *Limonium* subg*. Limonium* | The species shows morphological similarities with other Mediterranean species, such as *L. gummiferum* and *L. cossonianum.* The latter species is placed in the 'Mediterranean lineage' (Figure 3). | Greuter *et al.* (1989) |
| *Limonium beaumierianum* (Coss. ex Maire) Maire | *L.*sect. *Pteroclados* subsect. *Odontolepideae* in *Limonium* subg. *Pteroclados s.l.* | see Figure 2 |  |
| *Limonium bellidifolium* (Gouan) Dumort. | *L.* sect. *Nephrophyllum s.l.* in *Limonium* subg. *Limonium* | see Figure 2 |  |
| *Limonium benmageci* Marrero Rodr. | *L.* sect. *Pteroclados* subsect. *Nobiles* in *Limonium* subg. *Pteroclados s.l.* | see Figure 2 |  |
| ***Limonium bianorii* (Sennen & Pau) Erben** | ‘Mediterranean lineage’* in *Limonium* subg*. Limonium* | The species is endemic in the Baleares and shares morphological similarities with other Mediterranean species, such as *L. companyonis* and *L. duriusculum* (part of *L. duriusculum-companyonis* complex). Representatives of this complex (e.g. *L. thiniense* and *L. rigualii*) are placed in the 'Mediterranean lineage' (Figure 3). | Erben (1989), Erben (1993) and Sáez & Rosello (1999) |
| ***Limonium bicolor* Kuntze** | *L.* sect. *Plathymenium* in *Limonium* subg. *Limonium* | The species is morphologically similar to other representatives of *L.* sect. *Plathymenium,* such as *L. sinense* and *L. flexuosum* (see Figure 2). | Flora of China (online version): http://www.efloras.org/florataxon.aspx?flora_id=2&taxon_id=200017502 |
| *Limonium biflorum* (Pignatti) Pignatti | ‘Mediterranean lineage’* in *Limonium* subg*. Limonium* | see Figure 3 |  |
| ***Limonium billardierei* (Girard) Kuntze** | ‘Mediterranean lineage’* in *Limonium* subg*. Limonium* | The species has been assigned to *L.* sect. *Limonium* subsect. *Steirocladae* and is morphologically similar to *L. scabrum . Limonium scabrum* as well as representatives of the aforementioned subsection are all placed in the 'Mediterranean lineage' (see Figure 3). | Boissier (1848) and https://plants.jstor.org/stable/10.5555/al.ap.specimen.g00440137 |
| *Limonium binervosum* (G.E.Sm.) C.E.Salmon | ‘Mediterranean lineage’* in *Limonium* subg*. Limonium* | see Figure 3 |  |
| *Limonium bocconei* (Lojac.) Litard. | ‘Mediterranean lineage’* in *Limonium* subg*. Limonium* | see Figure 3 |  |
| ***Limonium boirae* L.Llorens & Tébar** | ‘Mediterranean lineage’* in *Limonium* subg*. Limonium* | The species is endemic to Baleares and is morphologically similar to other Mediterranean species*,* such as *L.biflorum* which is placed in the 'Mediterranean lineage' (Figure 3). | Erben (1993) |
| ***Limonium boitardii* Maire** | ‘Mediterranean lineage’* in *Limonium* subg*. Limonium* | The species is morphologically similar to *L. tunetanum* which is placed in the 'Mediterranean lineage' (Figure 3). | Examination of the Type specimen (including notes): https://plants.jstor.org/stable/10.5555/al.ap.specimen.mpu004360 |
| *Limonium bollei* (Webb ex Wangerin) Erben | ‘Mediterranean lineage’* in *Limonium* subg*. Limonium* | see Figure 3 |  |
| ***Limonium bolosii* L.Gil & L.Llorens** | ‘Mediterranean lineage’* in *Limonium* subg*. Limonium* | The species is similar morphologically to other Mediterranean species, especially from Baleares, such as *L. barceloi* and *L. gymnesicum.* The latter species is place in the 'Mediterranean lineage' (Figure 3). | Gil & García (1991) |
| ***Limonium bonafei* Erben** | ‘Mediterranean lineage’* in *Limonium* subg*. Limonium* | The species is morphologically similar to other Mediterranean species, especially from Baleares, such as *L. tenuicaule* which is part of '*L. minutum* group'. Representatives of this group are all phylogenetically placed in the 'Mediterranean lineage' (e.g. see *L. minutum* in Figure 3). | Erben (1993) and Palacios *et al.* (2000) |
| *Limonium bonduellei* (T.Lestib.) Kuntze | *L.*sect. *Pteroclados* subsect. *Odontolepideae* in *Limonium* subg. *Pteroclados s.l.* | see Figure 2 |  |
| *Limonium bonifaciense* Arrigoni & Diana | ‘Mediterranean lineage’* in *Limonium* subg*. Limonium* | see Figure 3 |  |
| ***Limonium bosanum* Arrigoni & Diana** | ‘Mediterranean lineage’* in *Limonium* subg*. Limonium* | The species is an endemic to Corse and Sardinia, and shows similarities to other Mediterranean endemics, such as *L. bonifaciense* and *L. obtusifolium,* both placed in the 'Mediterranean lineage' (Figure 3). | Arrigoni & Diana (1999) and Guarino *et al.* (2017) |
|    \| ***Limonium botschantzevii* (Lincz.) M.Malekm., Akhani & Borsch** \| \| --- \| | *L.* sect. *Siphonocalyx* in *Limonium* subg. *Limonium* | The species was previously assigned to genus *Eremolimon* (*Eremolimon botschantzevii* (Ikonn.-Gal.) Lincz.) which is currently embeded in *Limonium.* All represenatives of *Eremolimon* currently comprise *L.* sect. *Siphonocalyx* (see represenative in Figure 2). | Malekmohammadi *et al*. (2017) |
| *Limonium bourgeaui* (Webb ex Webb) Kuntze | *L.* sect. *Pteroclados* subsect. *Nobiles* in *Limonium* subg. *Pteroclados s.l.* | see Figure 2 |  |
| *Limonium brasiliense* (Boiss.) Kuntze | *L.* sect. *Limonium* in *Limonium* subg. *Limonium* | see Figure 2 |  |
| *Limonium brassicifolium* (Webb & Berthel.) Kuntze | *L.* sect. *Pteroclados* subsect. *Nobiles* in *Limonium* subg. *Pteroclados s.l.* | see Figure 2 |  |
| *Limonium braunii* (Bolle) A.Chev. | *L.* sect. *Ctenostachys* in *Limonium* subg. *Limonium* | see Figure 2 |  |
| *Limonium brevipetiolatum* R.Artelari & Erben | *L.* sect. *Limonium* in *Limonium* subg. *Limonium* | see Figure 2 |  |
| ***Limonium britannicum* Ingr.** | ‘Mediterranean lineage’* in *Limonium* subg*. Limonium* | The species is morphologically similar to *L. binervosum* and is part of *L. binervosum* aggregate. *Limonium binervosum* is placed in the 'Mediterranean lineage' (Figure 3). | Ingrouille & Stace (1986) |
| *Limonium brunneri* (Webb. ex Boiss.) Kuntze | *L.* sect. *Ctenostachys* in *Limonium* subg. *Limonium* | see Figure 2 |  |
| ***Limonium brusnicense* (Trinajstić) Bogdanović & Brullo** | ‘Mediterranean lineage’* in *Limonium* subg*. Limonium* | The species is morphologically similar to *L. cancellatum* and other species of *L. cancellatum* group. *Limonium cancellatum* is placed in the 'Mediterranean lineage' (Figure 3). | Bogdanović & Brullo (2015) |
| ***Limonium brutium* Brullo** | ‘Mediterranean lineage’* in *Limonium* subg*. Limonium* | The species is morphologically similar to *L. remotispiculum* (*L. multiforme* aggregate) which is placed in the 'Mediterranean lineage' (Figure 3). | Brullo (1992) |
| ***Limonium bulgaricum* Ančev** | *L.* sect. *Limonium* in *Limonium* subg. *Limonium* | The species is morphologically similar to *L. latifolium* which is placed in the clade of the re-circumscribed *L.* sect. *Limonium* (see *L.* sect. *Limonium* subsect. *Genuinae* in Figure 2). | Examination of the Type specimen (including notes): http://www.nmnhs.com/e-natura/types-bulgaria/type_id_en-SOMXXXXX0201.html |
| ***Limonium bungei* (Claus) Gamajun.** | *L.* sect. *Limonium* in *Limonium* subg. *Limonium* | The species is morphologically similar to other taxa of the re-circumscribed *L.* sect. *Limonium* such as *L. sareptanum* and *L. latifolium* (see *L. latifolium* in Figure 2)*.* | Pignatti (1972) and Malekmohammadi *et al*. (2017) |
| ***Limonium busianum* Bogdanović & Brullo** | ‘Mediterranean lineage’* in *Limonium* subg*. Limonium* | The species is morphologically similar to *L. cancellatum* and other species of *L. cancellatum* group. *Limonium cancellatum* is placed in the 'Mediterranean lineage' (Figure 3). | Bogdanović & Brullo (2015) |
| ***Limonium byzacium* Brullo & Erben** | ‘Mediterranean lineage’* in *Limonium* subg*. Limonium* | The species is part of *L. delicatulum* group, representatives of which are in the 'Mediterranean lineage' ( e.g. see *L. delicatulum, L. biflorum* in Figure 3). | Bartolo, Brullo & Giusso del Galdo (2003) |
| *Limonium caesium* (Girard) Kuntze | *L.* sect. *Polyarthrion* in *Limonium* subg. *Limonium* | see Figure 3 |  |
| ***Limonium calabrum* Brullo** | ‘Mediterranean lineage’* in *Limonium* subg*. Limonium* | The species is morphologically similar to *L. minutiflorum* which is placed in the 'Mediterranean lineage' (Figure 3). | Pignatti (1982a) |
| ***Limonium calanchicola* Erben** | ‘Mediterranean lineage’* in *Limonium* subg*. Limonium* | The species is morphologically similar to *L. articulatum* which is placed in the 'Mediterranean lineage' (Figure 3). | Erben (2002) |
| ***Limonium calcarae* (Janka) Pignatti** | ‘Mediterranean lineage’* in *Limonium* subg*. Limonium* | The species is part of *L. calcarae* complex which includes *L. calcarae, L. racemosum, L. multifurcatum, L. dichotomum* and *L. toletanum*. *Limonium dichotomum* and *L. toletanum* are placed in the 'Mediterranean lineage' (Figure 3). | Guarino *et al.* (2017) |
| *Limonium californicum* (Boiss.) A.Heller | *L.* sect. *Limonium* in *Limonium* subg. *Limonium* | see Figure 2 |  |
| ***Limonium callianthum* (T.X.Peng) Kamelin** | *L.* sect. *Siphonocalyx* in *Limonium* subg. *Limonium* | The species is closely related to *L. drepanostachyum* and it was previously a subspecies of it*. Limonium drepanostachyum* is assigned to *L.* sect. *Siphonocalyx* by Linczevski (1952; see a representative of this section in Figure 2)*.* | Linczevski (1952) |
| *Limonium calliopsium*Alf.Mayer | ‘Mediterranean lineage’* in *Limonium* subg*. Limonium* | see Figure 3 |  |
| *Limonium camposanum* Erben | ‘Mediterranean lineage’* in *Limonium* subg*. Limonium* | see Figure 3 |  |
| *Limonium cancellatum* (Bertol.) Kuntze | ‘Mediterranean lineage’* in *Limonium* subg*. Limonium* | see Figure 3 |  |
| ***Limonium* *capense* (L.Bolus) L.Bolus** | *L.* sect. *Circinaria* in *Limonium* subg. *Limonium* | see Figure 2 |  |
| ***Limonium capitis-eliae* Erben** | ‘Mediterranean lineage’* in *Limonium* subg*. Limonium* | The species is a narrow endemic of Sardinia and shows morphological similarities to other Mediterranean species, such as *L. virgatum* (part of *L. virgatum* aggr.) which is placed in the 'Mediterranean lineage' (Figure 3). | Greuter *et al.* (1989) and Erben (2001) |
| ***Limonium capitis-marci* Arrigoni & Diana** | ‘Mediterranean lineage’* in *Limonium* subg*. Limonium* | The species is morphologically similar to the Mediterranean taxa *L. sibthorpianum, L. optimae, L. lausianum* etc. and together with *L. mucronulatum* are part of *L. sibthorpianum* complex. *Limonium mucronulatum* was recently sampled in a phylogenetic study and is placed within the 'Mediterranean lineage' (see "*L. graecum* clade" in Malekmohammadi *et al.* 2017). | Greuter *et al.* (1989), Malekmohamadi *et al.* (2017) and Guarino *et al.* (2017) |
| ***Limonium caprariae* M.Rizzotto** | ‘Mediterranean lineage’* in *Limonium* subg*. Limonium* | The species is morphologically similar to other Mediterranean endemics, such as *L. articulatum* and *L. contortirameum,* both are placed in the 'Mediterranean lineage' (Figure 3). | Rizzotto (1999) |
| ***Limonium caprariense* (Font Quer & Marcos) Pignatti** | ‘Mediterranean lineage’* in *Limonium* subg*. Limonium* | The species is morphologically similar to other Mediterranean species, mostly from Baleares, such as *L. minutum* which is placed in the 'Mediterranean lineage' (Figure 3). *Limonium caprariense* was formerly a subspecies of *L. minutum.* | Pignatti (1972) and Erben (1993) |
| ***Limonium carisae* Erben** | ‘Mediterranean lineage’* in *Limonium* subg*. Limonium* | The species shares morphological similarities with *L. virgatum* which is placed in the 'Mediterranean lineage' in the phylogeny (Figure 3). | Erben (2001) |
| *Limonium carnosum* (Boiss.) Kuntze | *L.* sect. *Iranolimon* in *Limonium* subg. *Limonium* | see Figure 2 |  |
| *Limonium carolinianum* (Walter) Britton | *L.* sect. *Limonium* in *Limonium* subg. *Limonium* | see Figure 2 |  |
| *Limonium carpathum* (Rech.f.) Rech.f. | *‘*Mediterranean lineage’* in *Limonium* subg*. Limonium* | see Figure 3 |  |
| *Limonium carpetanicum* Erben | ‘Mediterranean lineage’* in *Limonium* subg*. Limonium* | see Figure 3 |  |
| ***Limonium carregadorense* Erben** | ‘Mediterranean lineage’* in *Limonium* subg*. Limonium* | The species is morphologically similar to *L. virgatum* which is placed in the 'Mediterranean clade' (Figure 3). | Erben (1989) |
| *Limonium carthaginense* (Rouy) C.E.Hubb. & Sandwith | ‘Mediterranean lineage’* in *Limonium* subg*. Limonium* | see Figure 3 |  |
| ***Limonium carvalhoi* Rosselló & L.Sáez** | *‘*Mediterranean lineage’* in *Limonium* subg*. Limonium* | The species is morhologically similar to other Mediterranean species of *L. delicatulum* group, such as *L. migjornense, L. magallufianum* etc. Representatives of *L. delicatulum* group such as *L. delicatulum and L. biflorum* are placed in the 'Mediterranean lineage' (Figure 3). | Erben (1993) and Rosselló *et al.* (1998) |
| ***Limonium caspium* (Willd.) Gams.** | *L.* sect. *Nephrophyllum s.l.* in *Limonium* subg. *Limonium* | The species is morphologically similar to other representatives of *L.* sect. *Nephrophyllum s.l.* especially *L. bellidifolium* (see also Figure 2 and Taxonomic proposals). | Linczevski (1952) |
| ***Limonium castellonense* Erben** | ‘Mediterranean lineage’* in *Limonium* subg*. Limonium* | The species is morphologically similar to *L. angustebracteatum* (*L. delicatulum* group). Representatives of this group, such as *L. delicatulum* and *L. tournefortii* are placed in the 'Mediterranean lineage' (Figure 3). | Erben (1980) |
| ***Limonium catalaunicum* (Willk. & Costa) Pignatti** | ‘Mediterranean lineage’* in *Limonium* subg*. Limonium* | The species is morphologically similar to *L. longebracteatum* which is placed in the 'Mediterranean lineage' (Figure 3). | Erben (1993) |
| ***Limonium catanense* (Lojac.) Brullo** | ‘Mediterranean lineage’* in *Limonium* subg*. Limonium* | The species is part of *L. densissimum* morphological aggregate. *Limonium densissimum* is placed in the 'Mediterranean lineage' (Figure 3). | Pignatti (1982a) |
| ***Limonium catanzaroi* Brullo** | ‘Mediterranean lineage’* in *Limonium* subg*. Limonium* | The species is morphologically similar to *L. virgatum* and is part of *L. virgatum* aggregate. *Limonium virgatum* is placed in the 'Mediterranean lineage' (Figure 3). | Pignatti (1982a) and Greuter *et al.* (1989) |
| ***Limonium cavanillesii* Erben** | ‘Mediterranean lineage’* in *Limonium* subg*. Limonium* | The species is part of *L. duriusculum-companyonis* complex. Representatives of this complex such as *L. thiniense* and *L. rigualii* are placed in the 'Mediterranean lineage' (Figure 3). | Sáez & Rosello (1999) |
| ***Limonium cazzae* Bogdanović & Brullo** | ‘Mediterranean lineage’* in *Limonium* subg*. Limonium* | The species is morphologically similar to *L. cancellatum* and other species of *L. cancellatum* group. *Limonium cancellatum* is placed in the 'Mediterranean lineage' (Figure 3). | Bogdanović & Brullo (2015) |
| ***Limonium cedrorum* Domina & Raimondo** | ‘Mediterranean lineage’* in *Limonium* subg*. Limonium* | The species is morphologically similar to other Eastern Mediterranean taxa, such as *L. sieberi* and *L. graecum.* Both species are placed in the 'Mediterranean lineage' (Figure 3). | Domina & Raimondo (2013) |
| *Limonium cephalonicum* R.Artelari | ‘Mediterranean lineage’* in *Limonium* subg*. Limonium* | see Figure 3 |  |
| ***Limonium cercinense* Brullo & Erben** | ‘Mediterranean lineage’* in *Limonium* subg*. Limonium* | The species is a triploid, morphologically similar to *L. rubescens* and *L. delicatulum.* The latter species is placed in the 'Mediterranean lineage' (Figure 3). | Brullo & Erben (1989) |
| ***Limonium chazaliei* (H.Boissieu) Maire** | *L.* sect. *Ctenostachys* in *Limonium* subg. *Limonium* | The species is mophologically similar to other taxa from *L.* sect. *Ctenostachys,* such as *L. fallax, L. mucronatum, L. brunneri* etc. (see clade of *L.* sect. *Ctenostachys* in Figure 2). | Maire (1936) and http://www.jardincanario.org/limonium-fitogeografia-y-ecologia |
| *Limonium chersonesum* Erben & Brullo | ‘Mediterranean lineage’* in *Limonium* subg*. Limonium* | see Figure 3 |  |
| ***Limonium chodshamumynense* Lincz. & Czukav.** | *L.* sect. *Siphonocalyx s.l.* in *Limonium* subg. *Limonium* | The species is morphologically similar to *L. ferganense. Limonium ferganense* is phylogenetically related to *L.* sect. *Siphonocalyx* and shares some morphological similarities with its representatives. In a recent study *L. ferganense* was sister to *L. sogdianum* and *L. piptopodum* of *L.* sect. *Siphonocalyx;* all three of them comprising the "*L. sogdianum* clade" (Malekmohammadi *et al.* 2017). | Linczevski & Czukavina (1984) and Malekmohammadi *et al*. (2017) |
| ***Limonium chrisianum* Brullo & Guarino** | ‘Mediterranean lineage’* in *Limonium* subg*. Limonium* | The species is related to *L. cornarianum* which is placed in the 'Mediterranean lineage' (Figure 3). | Brullo & Erben (2016) |
| ***Limonium chrysocomum* (Kar. & Kir.) Kuntze** | *L.* sect. *Plathymenium* in *Limonium* subg. *Limonium* | The species has been assigned to *L.* sect. *Plathymenium* and shows morphological similarities to representatives of this section (see representatives in Figure 2) | Linczevski (1952) |
| ***Limonium chrysopotamicum* Maire** | *L.* sect. *Ctenostachys* in *Limonium* subg. *Limonium* | The species is morphologically similar to *L. fallax* and *L. mucronatum* and is assigned to *L.* sect. *Ctenostachys* (see also Figure 2). | Sauvage & Vindt (1952) |
| *Limonium circaei* Pignatti | ‘Mediterranean lineage’* in *Limonium* subg*. Limonium* | see Figure 3 |  |
| ***Limonium clupeanum* Brullo & Erben** | ‘Mediterranean lineage’* in *Limonium* subg*. Limonium* | The species is morphologically similar to *L. densiflorum, L. confertum* and other representatives of *L. densiflorum* complex (sensu Guarino *et al.* 2017). *Limonium glomeratum* as a representative of this complex is phylogenetically related to other taxa of the 'Mediterranean lineage' (see details below in *L. glomeratum*) | Brullo & Erben (1989) |
| ***Limonium cofrentanum* Erben** | ‘Mediterranean lineage’* in *Limonium* subg*. Limonium* | The species is a diploid endemic of Spain that is part of *L. cofrentanum* complex (sensu Erben 1989) and shares morphological similarities with *L. lobetanicum, L. sucronicum* and *L. aragonense. Limonium aragonense* is placed in the 'Mediterranean lineage' in the phylogeny (Figure 3) | Erben (1989) and Erben (1993) |
| ***Limonium coincyi* Sennen** | ‘Mediterranean lineage’* in *Limonium* subg*. Limonium* | *Limonium coincyi* is supposed to be a hybrid between *L. album* and *L. supinum.* The former species is related to *L. cossonianum* which is similar to *L. supinum* and are both placed in the 'Mediterranean lineage' (Figure 3). | Erben (1993) |
| ***Limonium comosum* Erben** | ‘Mediterranean lineage’* in *Limonium* subg*. Limonium* | The species is morphologically close to *L. tunetanum* which is placed in the 'Mediterranean lineage' in the phylogeny (Figure 3). | Erben (2001) |
| *Limonium compactum* Erben & Brullo | *L.* sect. *Limonium* in *Limonium* subg. *Limonium* | see Figure 2 |  |
| ***Limonium companyonis* Kuntze** | ‘Mediterranean lineage’* in *Limonium* subg*. Limonium* | The species is part of *L. duriusculum-companyonis* complex. Representatives of this complex such as *L. thiniense* and *L. rigualii* are placed in the 'Mediterranean lineage' (Figure 3). | Sáez & Rosello (1999) |
| ***Limonium confertum* Brullo & Erben** | ‘Mediterranean lineage’* in *Limonium* subg*. Limonium* | The species is morphologically similar to *L. densiflorum, L. clupeanum* and other representatives of *L. densiflorum* complex (sensu Guarino *et al.* 2017). *Limonium glomeratum* as a representative of this complex is phylogenetically related to other taxa of the 'Mediterranean lineage' (see details below in *L. glomeratum*). | Brullo & Erben (1989) |
| *Limonium confusum* (Godr. & Gren.) Fourr. | ‘Mediterranean lineage’* in *Limonium* subg*. Limonium* | see Figure 3 |  |
| ***Limonium congestum* Kuntze** | *L.* sect. *Plathymenium* in *Limonium* subg. *Limonium* | The species has been assigned to *L.* sect. *Plathymenium* and shows morphological similarities to representatives of this section (e.g. *L. flexuosum*; see Figure 2) | Linczevski I. A. (1952) |
| ***Limonium connivens* Erben** | ‘Mediterranean lineage’* in *Limonium* subg*. Limonium* | The species is part of *L. duriusculum-companyonis* complex. Representatives of this complex such as *L. thiniense* and *L. rigualii* are placed in the 'Mediterranean lineage' in the phylogeny (Figure 3). | Erben (1989) and Sáez & Rosello (1999) |
| *Limonium contortirameum* (Mabille) Erben | ‘Mediterranean lineage’* in *Limonium* subg*. Limonium* | see Figure 3 |  |
| ***Limonium contractum* Erben & Brullo** | ‘Mediterranean lineage’* in *Limonium* subg*. Limonium* | The species is morphologically similar to other Eastern Mediterranean taxa such as *L. pusillum, L. palmare, L. graecum* etc. *Limonium graecum* is placed in the 'Mediterranean lineage' (Figure 3). | Brullo & Erben (2016) |
| ***Limonium cophanense* C.Brullo, Brullo, Cambria, Giusso & Ilardi** | ‘Mediterranean lineage’* in *Limonium* subg*. Limonium* | The species is morphologically similar to *L. bocconei* (part of *L. bocconei* aggregate) which is placed in the 'Mediterranean lineage' (Figure 3). | Brullo *et al.* (2016) |
| ***Limonium coralloides* (Tausch) Lincz.** | *L.* sect. *Nephrophyllum s.l.* in *Limonium* subg. *Limonium* | The species is morphologically similar to other representatives of *L.* section *Nephrophyllum s.l.* especially the ones of *L. bellidifolium* complex (see also Figure 2 and Taxonomic proposals). | Linczevski (1952) |
| *Limonium cordatum* (L.) Mill. | ‘Mediterranean lineage’* in *Limonium* subg*. Limonium* | see Figure 3 |  |
| ***Limonium cordovillense* Stübing & Cirujano** | ‘Mediterranean lineage’* in *Limonium* subg*. Limonium* | The species is morphologically similar to *L. supinum* and *L. toletanum*, which are both placed in the 'Mediterranean lineage' (Figure 3). | Roselló *et al.* (1997) |
| ***Limonium corinthiacum* (Boiss. & Heldr.) Kuntze** | ‘Mediterranean lineage’* in *Limonium* subg*. Limonium* | The species is rather morphologically similar to *L. aphroditae,* which is placed in the 'Mediterranean lineage' in the phylogeny (Figure 3). | Brullo & Erben (2016) |
| *Limonium cornarianum*Kypr. & R.Artelari - | ‘Mediterranean lineage’* in *Limonium* subg*. Limonium* | see Figure 3 |  |
| ***Limonium cornusianum* Arrigoni & Diana** | ‘Mediterranean lineage’* in *Limonium* subg*. Limonium* | The species is morphologically similar to *L. acutifolium,* it's part of  *"acutifolium"* group and was formerly a subspecies of *L. acutifolium* (*Limonium acutifolium* subsp. *cornusianum* (Arrigoni & Diana) Arrigoni). Representantives of this group such as *L. bonifaciense* and *L. obtusifolium* are placed in the 'Mediterranean lineage' (Figure 3). | Arrigoni & Diana (1999) and Guarino *et al*. (2017) |
| *Limonium coronense* R.Artelari | ‘Mediterranean lineage’* in *Limonium* subg*. Limonium* | see Figure 3 |  |
| *Limonium corsicum* Erben | ‘Mediterranean lineage’* in *Limonium* subg*. Limonium* | see Figure 3 |  |
| *Limonium cossonianum* Kuntze | ‘Mediterranean lineage’* in *Limonium* subg*. Limonium* | see Figure 3 |  |
| *Limonium costae* (Willk.) Pignatti | ‘Mediterranean lineage’* in *Limonium* subg*. Limonium* | see Figure 3 |  |
| *Limonium cosyrense* (Guss.) Kuntze | ‘Mediterranean lineage’* in *Limonium* subg*. Limonium* | see Figure 3 |  |
| *Limonium crateriforme* Erben & Brullo | ‘Mediterranean lineage’* in *Limonium* subg*. Limonium* | see Figure 3 |  |
| ***Limonium cretaceum* Cherkasova** | *L.* sect. *Nephrophyllum s.l.* in *Limonium* subg. *Limonium* | The species is morphologically similar to representatives of *L.* sect. *Nephrophyllum s.l.* such as the ones of *L. bellidifolium* complex (e.g. *L. macrorrhizon*; see also Figure 2 and Taxonomic proposals). | Cherkasova (1970) |
| *Limonium creticum* R. Artelari | ‘Mediterranean lineage’* in *Limonium* subg*. Limonium* | see Figure 3 |  |
| ***Limonium cumanum* (Ten.) Kuntze** | ‘Mediterranean lineage’* in *Limonium* subg*. Limonium* | The species is morphologically and phylogenetically related to other Mediterranean taxa of the 'Mediterranean lineage' (see Malekmohammadi *et al.* 2017). | Malekmohammadi *et al.* (2017) |
| ***Limonium cunicularium* Arrigoni & Diana** | ‘Mediterranean lineage’* in *Limonium* subg*. Limonium* | The species is morphologically similar to *L. acutifolium* and it's part of *"acutifolium"* group. Representantives of this group such as *L. bonifaciense* and *L. obtusifolium* are placed in the 'Mediterranean lineage' (Figure 3). | Arrigoni & Diana (1999) |
| ***Limonium cuspidatum* (Delort) Erben** | ‘Mediterranean lineage’* in *Limonium* subg*. Limonium* | The species is morphologically similar to other Mediterranean taxa such as *L. ramosissimum, L. densissimum* and *L. confusum. Limonium densissimum* and *L. confusum* are placed in the 'Mediterranean lineage' (Figure 3). | Pavon (2005) |
| *Limonium cylindrifolium* (Forssk.) Verdc. ex Cufod. | *L.* sect. *Sarcophyllum* in *Limonium* subg. *Limonium* | see Figure 2 |  |
| *Limonium cymuliferum* (Boiss.) Sauvage & Vindt | ‘Mediterranean lineage’* in *Limonium* subg*. Limonium* | see Figure 3 |  |
| ***Limonium cyprium* (Meikle) Hand & Buttler** | ‘Mediterranean lineage’* in *Limonium* subg*. Limonium* | The species is morphologically similar to *L. albidum* and is part of *L. albidum* group. *Limonium hyblaeum* which is a representative of *L. albidum* group is placed in the 'Mediterranean lineage' (Figure 3). | Kouzali *et al.* (2012) |
| ***Limonium cyrenaicum* (Rouy) Brullo** | ‘Mediterranean lineage’* in *Limonium* subg*. Limonium* | The species is part of *L. graecum* complex. Representatives of this complex such as *L. graecum* and *L. roridum* are placed in the 'Mediterranean lineage' (Figure 3). | Brullo (1978) |
| ***Limonium cyrtostachyum* (Girard) Brullo** | ‘Mediterranean lineage’* in *Limonium* subg*. Limonium* | The species is part pf *L. minutiflorum* aggregate. *Limonium minutiflorum* is placed in the 'Mediterranean lineage' (Figure 3). | Greuter et al. (1989) |
| *Limonium cythereum* R.Artelari & Georgiou | ‘Mediterranean lineage’* in *Limonium* subg*. Limonium* | see Figure 3 |  |
| ***Limonium damboldtianum* Phitos & R.Artelari** | ‘Mediterranean lineage’* in *Limonium* subg*. Limonium* | The species is morphologically similar to *L. cancellatum* and *L. cephalonicum.* Both species are placed in the 'Mediterranean lineage' (Figure 3). | Artelari (1984) and Brullo & Erben (2016) |
| ***Limonium danubiale* Klokov** | *L.* sect. *Nephrophyllum s.l.* in *Limonium* subg. *Limonium* | The species is morphologically similar to other representatives of *L.* sect. *Nephrophyllum s.l.* especially *L. bellidifolium* (see also Figure 2). | Pignatti (1972) |
| *Limonium daveaui* Erben | ‘Mediterranean lineage’* in *Limonium* subg*. Limonium* | see Figure 3 |  |
| ***Limonium decumbens* (Boiss.)Kuntze** | ‘Mediterranean lineage’* in *Limonium* subg*. Limonium* | The species shares morphological similarities with *L. scabrum* (sampled in the phylogeny and placed in the 'Mediterranean lineage'; Figure 3), *L. acuminatum* and *L. equisetinum,* all of them were assigned to *L.* sect. *Limonium* subsect. *Steirocladae* by Boissier. Representatives of this subsection are all placed in the 'Mediterranean lineage' (Figure 3). | Dyer (1963) |
| *Limonium delicatulum* (Girard) Kuntze | ‘Mediterranean lineage’* in *Limonium* subg*. Limonium* | see Figure 3 |  |
| *Limonium dendroides* Svent. | *L.* sect. *Limoniodendron* in *Limonium* subg. *Limonium* | see Figure 2 |  |
| ***Limonium densiflorum* (Guss.) Kuntze** | ‘Mediterranean lineage’* in *Limonium* subg*. Limonium* | The species is morphologically similar to other Mediterranean species, mostly form Westen Mediterranean, such as *L. glomeratum* which is also part of *L. densiflorum* group. *Limonium glomeratum* is morphologically and phylogenetically related to other taxa of the 'Mediterranean lineage' (see details below in *L. glomeratum*). | Brullo & Pavone (1981) and Brullo (1988) |
| *Limonium densissimum* (Pignatti) Pignatti | ‘Mediterranean lineage’* in *Limonium* subg*. Limonium* | see Figure 3 |  |
| ***Limonium depauperatum* (Boiss.) R.A.Dyer** | ‘Mediterranean lineage’* in *Limonium* subg*. Limonium* | The species shares morphological similarities with other South African taxa such as *L. equisetinum* (previously it was a variety of this species under the former generic name: *Statice equisetina* var. *depauperata* Boiss. ), *L. acuminatum* and *L. scabrum. Limonium scabrum* is placed in the 'Mediterranean lineage' (Figure 3). | Dyer (1963) |
| ***Limonium dianium* Pignatti** | ‘Mediterranean lineage’* in *Limonium* subg*. Limonium* | The species is part of *L. multiforme* aggregate (*L. cosyrense* group) and thus, morphologically similar to *L. multiforme,* which is placed in the 'Mediterranean lineage' (Figure 3). | Pignatti (1982a,b) |
| *Limonium dichotomum* (Cav.) Kuntze | ‘Mediterranean lineage’* in *Limonium* subg*. Limonium* | see Figure 3 |  |
| *Limonium dichroanthum* (Rupr.) Ikonn.-Gal. | *L.* sect. *Plathymenium* in *Limonium* subg. *Limonium* | see Figure 2 |  |
| ***Limonium didimense* Doğan & Akaydın** | ‘Mediterranean lineage’* in *Limonium* subg*. Limonium* | The species is morphologically similar to *L. virgatum* and *L. sieberi*. These species are placed in the 'Mediterranean lineage' (Figure 3). | Doğan & Akaydın (2017) |
| ***Limonium dielsianum* (Wangerin) Kamelin** | *L.* sect. *Plathymenium* in *Limonium* subg. *Limonium* | The species is morphologically similar to taxa from *L.* sect. *Plathymenium*and particularly *L. aureum* (see Figure 2)*. Limonium dielsianum* was formerly a variety of *L. aureum* (*Limonium aureum* L. var. *dielsianum* (Wangerin) Peng.). | Flora of China (online version): http://www.efloras.org/florataxon.aspx?flora_id=2&taxon_id=210001018 |
| ***Limonium diomedeum* Brullo** | ‘Mediterranean lineage’* in *Limonium* subg*. Limonium* | The species is morphologically similar to *L. cancellatum* (part of *L. cancellatum* group). Represenatives of this group, such as *L. cancellatum* is placed in the 'Mediterranean lineage' (Figure 3). | Brullo (1988) |
| ***Limonium divaricatum* (Rouy) Brullo** | ‘Mediterranean lineage’* in *Limonium* subg*. Limonium* | The species is morphologically similar to *L. graecum* and was previously a subspecies of it. *Limonium graecum* is placed in the 'Mediterranean lineage' (Figure 3). | Pignatti (1982a) |
| ***Limonium dodartiforme*Ingr.** | ‘Mediterranean lineage’* in *Limonium* subg*. Limonium* | The species is morphologically similar to *L. binervosum* (part of *L. binervosum* aggregate) which is placed in the 'Mediterranean lineage' (Figure 3). | Pignatti (1972) and Ingrouille & Stace (1986) |
| *Limonium dodartii* (Girard) Kuntze | ‘Mediterranean lineage’* in *Limonium* subg*. Limonium* | see Figure 3 |  |
| ***Limonium doerfleri* (Halácsy) Rech.f.** | ‘Mediterranean lineage’* in *Limonium* subg*. Limonium* | The species is morphologically similar to *L. sirinicum* and *L. albomarginatum* (*L. doerfleri* complex). The latter species is placed in the 'Mediterranean lineage' (Figure 3). | Brullo & Erben (2016) |
| ***Limonium dolcheri* Pignatti** | ‘Mediterranean lineage’* in *Limonium* subg*. Limonium* | The species is morphologically similar to *L. articulatum* (*L. articulatum* group) which is placed in the 'Mediterranean lineage' (Figure 3). | Pignatti (1982a) |
| ***Limonium dolihiense* Erben & Brullo** | ‘Mediterranean lineage’* in *Limonium* subg*. Limonium* | The species is morphologically similar to other taxa from the Aegean region (East Mediterranean) such as *L. athinense, L. fragile* and *L. xerocamposicum. Limonium xerocamposicum* is placed in the 'Mediterranean lineage' (Figure 3). | Brullo & Erben (2016) |
| ***Limonium donetzicum* Klokov** | *L.* sect. *Limonium* in *Limonium* subg. *Limonium* | The species is morphologically similar to *L. tomentellum,* which is placed in the clade of the re-circumscribed *L.* sect. *Limonium* (i.e. former *L.* sect. *Limonium* subsect. *Genuinae*; Figure 2). | Pignatti (1972) |
| ***Limonium doriae* (Sommier) Pignatti** | ‘Mediterranean lineage’* in *Limonium* subg*. Limonium* | The species is morphologically similar to other Mediterranean species, mostly form Westen Mediterranean, such as *L. glomeratum* which together with *L. doriae* is also part of *L. densiflorum* complex. *Limonium glomeratum* as a representative of this complex is phylogenetically related to other taxa of the 'Mediterranean lineage' (see details below in *L. glomeratum*). | Pignatti (1982a) and Guarino *et al.* (2017) |
| ***Limonium dragonericum* Erben** | ‘Mediterranean lineage’* in *Limonium* subg*. Limonium* | The species shows morphological similarities to *L. pseudodictyocladum* and *L. virgatum.* The latter species is part of the 'Mediterranean lineage' (Figure 3). | Erben (1989) |
| ***Limonium dregeanum* (Presl) Kuntze** | ‘Mediterranean lineage’* in *Limonium* subg*. Limonium* | The species is morphologically similar to *L. dyeri* (syn. *L. membranaceum*) and *L. scabrum.* The latter species is placed in the 'Mediterranean lineage' (Figure 3). | Dyer (1963) |
|    \| ***Limonium drepanostachyum* Ikonn.-Gal.** \| \| --- \| | *L.* sect. *Siphonocalyx* in *Limonium* subg. *Limonium* | The species was previously assigned to genus *Eremolimon* (*Eremolimon drepanostachyum* Lincz.) which is currently embeded in *Limonium.* All represenatives of *Eremolimon* currently comprise *L.* sect. *Siphonocalyx* (see represenative in Figure 2). | Malekmohammadi *et al*. (2017) |
| ***Limonium dubium* (Andrz. ex Guss.) Litard** | ‘Mediterranean lineage’* in *Limonium* subg*. Limonium* | The species is morphologically similar to *L. virgatum* and is part of *L. virgatum* aggregate. *Limonium virgatum*is placed in the 'Mediterranean lineage' (Figure 3). | Pignatti (1982a; under the synonym *L. pignattii*) and Greuter *et al.* (1989) |
| ***Limonium dubyi* (Gren. & Godron) Kuntze** | *L.* sect. *Nephrophyllum s.l.* in *Limonium* subg. *Limonium* | The species is morphologically similar to *L. bellidifolium* which is part of *L.* sect. *Nephrophyllum s.l.* (see also Figure 2). *Limonium dubyi* was formerly a subspecies of *L. bellidifolium* (*L. bellidifolium* subsp. *dubyi* (Godron & Gren.) P. Fourn.)*.* | Pignatti (1972) and Erben (1978) |
| *Limonium dufourii* (Girard) Kuntze | ‘Mediterranean lineage’* in *Limonium* subg*. Limonium* | see Figure 3 |  |
| ***Limonium duriaei* (Girard) Kuntze** | ‘Mediterranean lineage’* in *Limonium* subg*. Limonium* | The species has been assigned to *L.* sect. *Limonium* subsect. *Densiflora* by Sauvage & Vindt (1952). It is morphologically similar to *L. densiflorum* and other representatives of *L. densiflorum* complex (sensu Guarino et al. 2017). *Limonium glomeratum* as a representative of this complex is phylogenetically related to other taxa of the 'Mediterranean lineage' (see details below in *L. glomeratum*). | Sauvage & Vindt (1952) and examination of the Isosyntype of *Statice duriaei* Girard including notes: https://plants.jstor.org/stable/10.5555/al.ap.specimen.mpu021625?searchUri=filter%3Dname%26so%3Dps_group_by_genus_species%2Basc%26Query%3DStatice%2Bduriaei%2BGirard%2B |
| ***Limonium duriusculum* (Girard) Fourr.** | ‘Mediterranean lineage’* in *Limonium* subg*. Limonium* | The species is part of *L. duriusculum-companyonis* complex. Representatives of this complex such as *L. thiniense* and *L. rigualii* are placed in the 'Mediterranean lineage' (Figure 3). | Sáez & Rosello (1999) |
| ***Limonium dyeri* Lincz.** | ‘Mediterranean lineage’* in *Limonium* subg*. Limonium* | The species is morphologically similar to *L. scabrum,* which is placed in the 'Mediterranean lineage' (Figure 3). | Dyer (1961) and Dyer (1963) under *L. membranaceum* R.A.Dyer |
| *Limonium ebusitanum* (Font Quer) Font Quer | ‘Mediterranean lineage’* in *Limonium* subg*. Limonium* | see Figure 3 |  |
| *Limonium echioides* (L.) Mill. | *L.* sect. *Schizhymenium* in *Limonium* subg. *Limonium* | see Figure 3 |  |
| *Limonium effusum* (Boiss.) Kuntze | *L.* sect. *Limonium* in *Limonium* subg. *Limonium* | see Figure 2 |  |
| ***Limonium ejulabilis* Rosselló, Mus & J.X.Soler** | ‘Mediterranean lineage’* in *Limonium* subg*. Limonium* | The species is part of *L. delicatulum* complex, representatives of which (e.g. *L. delicatulum, L. majus, L. santapolense*) are placed in the 'Mediterranean lineage' (Figure 3). | Rosselló, Amézquita, & Marí (1993) |
| *Limonium elaphonisicum* Alf. Mayer | ‘Mediterranean lineage’* in *Limonium* subg*. Limonium* | see Figure 3 |  |
| ***Limonium elfahsianum* Brullo & Giusso** | ‘Mediterranean lineage’* in *Limonium* subg*. Limonium* | The species is morphologically similar to *L. todaroanum* which is part of *L. densissimum* aggregate (see Colombo 2002). A representative of this aggregate, *L. densissimum,* is placed in the 'Mediterranean lineage' (Figure 3). | Colombo (2002) and Brullo & Giusso del Galdo (2006) |
| ***Limonium emarginatum* (Willd.) Kuntze** | ‘Mediterranean lineage’* in *Limonium* subg*. Limonium* | The species is morphologically very similar to *L. spathulatum,* which has been assigned to *L.* sect. *Limonium* subsect. *Dissitiflora* (see Sauvage & Vindt 1952). Representatives of this subsection are all placed in the 'Mediterranean lineage' (Figure 3). | Sauvage & Vindt (1952) and Erben (1993) |
| ***Limonium equisetinum* (Boiss.) R.A.Dyer** | ‘Mediterranean lineage’* in *Limonium* subg*. Limonium* | The species has morphological similarities to other South African taxa such as *L. depauperatum*, *L. acuminatum* and *L. scabrum. Limonium scabrum* is placed in the 'Mediterranean lineage' (Figure 3). | Dyer (1963) |
| *Limonium erectum* Erben | ‘Mediterranean lineage’* in *Limonium* subg*. Limonium* | see Figure 3 |  |
| *Limonium estevei* Fern.Casas | ‘Mediterranean lineage’* in *Limonium* subg*. Limonium* | see Figure 3 |  |
| ***Limonium etruscum* Arrigoni & Rizzotto** | ‘Mediterranean lineage’* in *Limonium* subg*. Limonium* | The species is morphologically similar to *L. glomeratum, L. densiflorum* and *L. selinuntinum* which together with *L. etruscum* are part of *L. densiflorum* complex. *Limonium glomeratum* as a representative of this complex is phylogenetically related to other taxa of the 'Mediterranean lineage' (see details below in *L. glomeratum*). | Arrigoni & Rizzotto (1985) and Guarino *et al.* (2017) |
| ***Limonium eugeniae* Sennen** | ‘Mediterranean lineage’* in *Limonium* subg*. Limonium* | The species is morphologically similar to *L. tunetanum* and it was formerly given a subspecies ranking (i.e. *Limonium tunetanum* subsp. *eugeniae* (Sennen)). *Limonium tunenatum* is placed in the 'Mediterranean lineage' (Figure 3). | Greuter *et al.* (1989) |
| ***Limonium failachicum* Erben & Mucina** | *L.* sect. *Iranolimon* in *Limonium* subg. *Limonium* | The species is morphologically similar to *L. carnosum* and *L. iranicum,* both are placed in the *L.* sect. *Iranolimon* (see Figure 2) | Erben & Mucina (2006) |
|    \| ***Limonium fajzievii* Zakirov** \| \| --- \| | *L.* sect. *Siphonocalyx* in *Limonium* subg. *Limonium* | The species was previously assigned to genus *Eremolimon* (*Eremolimon fajzievii* (Zak. ex Lincz.) Lincz. ) which is currently embeded in *Limonium.* All represenatives of *Eremolimon* currently comprise *L.* sect. *Siphonocalyx* (see represenative in Figure 2). | Malekmohammadi *et al*. (2017) |
| *Limonium fallax* (Coss. ex Wangerin) Maire | *L.* sect. *Ctenostachys* in *Limonium* subg. *Limonium* | see Figure 2 |  |
| ***Limonium ferganense* Ikonn.-Gal.** | *L.* sect. *Siphonocalyx s.l.* in *Limonium* subg. *Limonium* | The species is phylogenetically related to *L.* sect. *Siphonocalyx* and shares some morphological similarities with its representatives. In a recent study *L. ferganense* was sister to *L. sogdianum* and *L. piptopodum* comprising the "*L. sogdianum* clade" (see Malekmohammadi *et al.* 2017). | Malekmohammadi *et al.* (2017) |
| ***Limonium fesianum* Erben** | *L.* sect. *Pruinosum* in *Limonium* subg. *Limonium* | The species is morphologically similar to *L. asparagoides* which is place in *L.* sect. *Pruinosum* (see aslo *L. asparagoides* in the phylogeny in Figure 3). | Erben (2001) |
| ***Limonium fischeri* (Trautv.) Lincz.** | *L.* sect. *Plathymenium* in *Limonium* subg. *Limonium* | The species is morphologically similar to *L. nudum* (see Figure 2) and has been assigned to *L.* sect. *Plathymenium* (Linczevski 1952) *.* | Linczevski (1952) |
| ***Limonium flagellare* (Lojac.) Brullo** | ‘Mediterranean lineage’* in *Limonium* subg*. Limonium* | The species is morphologically similar to *L. bocconei* (*L. bocconei* aggregate) which is placed in the 'Mediterranean lineage' (Figure 3). | Pignatti (1982a) and Brullo (1988) |
| *Limonium flexuosum* (L.) Kuntze | *L.* sect. *Plathymenium* in *Limonium* subg. *Limonium* | see Figure 2 |  |
| ***Limonium florentinum* Arrigoni & Diana** | ‘Mediterranean lineage’* in *Limonium* subg*. Limonium* | The species is morphologically similar to *L. corsicum* and *L. greuteri,* and is a member of *L. articulatum* aggregate. The aforementioned taxa are placed in the 'Mediterranean lineage' (Figure 3). | Erben (2006) |
| ***Limonium fontqueri* (Pau) L. Llorens** | ‘Mediterranean lineage’* in *Limonium* subg*. Limonium* | The species is morphologically similar to *L. tamarindanum* and *L. virgatum. Limonium fontqueri* was formerly ranked as subspecies of *L. virgatum* (*Limonium virgatum* subsp. *fontqueri* (Pau) O. de Bolòs, J. Vigo, R.M. Masalles & J.M. Ninot). *Limonium virgatum* is placed in the 'Mediterranean lineage' (Figure 3). | Erben (1989) and Erben (1993) |
| ***Limonium formosum* Bartolo, Brullo & Giusso** | ‘Mediterranean lineage’* in *Limonium* subg*. Limonium* | The species is part of *L. delicatulum* group and is morphologically similar to *L. byzacium* and *L. neapolense*. Representatives of *L. delicatulum* group (e.g. *L. delicatulum, L. tournefortii*, *L. biflorum* etc.) are all placed in the 'Mediterranean lineage' (Figure 3). | Bartolo, Brullo & Giusso del Galdo (2003) |
| ***Limonium fradinianum* (Pomel) Erben** | ‘Mediterranean lineage’* in *Limonium* subg*. Limonium* | The species is morphologically similar to *L. densiflorum.*  *Limonium glomeratum* which is also part of *L. densiflorum* group is morphologically and phylogenetically related to other taxa of the 'Mediterranean lineage' (see details below in *L. glomeratum*). | Quezel *et al.* (1963) |
| ***Limonium fragile* Erben & Brullo** | ‘Mediterranean lineage’* in *Limonium* subg*. Limonium* | The species is morphologically similar to other taxa from the Aegean region (East Mediterranean) such as *L. athinense* and *L. xerocamposicum* (*L. fragile* complex)*.* The latter species is placed in the 'Mediterranean lineage' (Figure 3). | Brullo & Erben (2016) |
| ***Limonium franchetii* (Debeaux) Kuntze** | *L.* sect. *Plathymenium* in *Limonium* subg. *Limonium* | The species is morphologically similar to other taxa of *L.* sect. *Plathymenium* such as *L. sinense,* sampled in the current phylogeny (see Figure 2). | Flora of China (online version): http://www.efloras.org/florataxon.aspx?flora_id=2&taxon_id=200017511 |
| *Limonium frederici* (Barbey) Rech.f. | *‘*Mediterranean lineage’* in *Limonium* subg*. Limonium* | see Figure 3 |  |
| *Limonium frutescens* (Lem.) Erben, A.Santos & Reyes-Bet. | *L.* sect. *Pteroclados* subsect. *Nobiles* in *Limonium* subg. *Pteroclados s.l.* | see Figure 2 |  |
| *Limonium furfuraceum* (Lag.) Kuntze | ‘Mediterranean lineage’* in *Limonium* subg*. Limonium* | see Figure 3 |  |
| ***Limonium furnarii* Brullo** | ‘Mediterranean lineage’* in *Limonium* subg*. Limonium* | The species is morphologically similar to *L. minutiflorum* (*L. minutiflorum* group) which is placed in the 'Mediterranean lineage' (Figure 3). | Pignatti (1982a) |
| ***Limonium galilaeum* Domina, Danin & Raimondo** | ‘Mediterranean lineage’* in *Limonium* subg*. Limonium* | The species is morphologically similar to *L. graecum* and *L. sieberi.* Both species are placed in the 'Mediterranean lineage' (Figure 3). | Domina *et al.* (2006) |
| ***Limonium gallicum* (Pignatti) Domina** | ‘Mediterranean lineage’* in *Limonium* subg*. Limonium* | The species is morphologically similar to *L. ovalifolium* which is placed in the 'Mediterranean lineage' (Figure 3). The species was previously a subspecies of *L. ovalifolium* (*L. ovalifolium* subsp. *gallicum* Pignatti). | Pignatti (1972) |
| ***Limonium gallurense* Arrigoni & Diana** | ‘Mediterranean lineage’* in *Limonium* subg*. Limonium* | The species is part of '*L. acutifolium* group'. Represenatives of this group, such as *L. obtusifolium* and *L. bonifaciense* are placed in the 'Mediterranean lineage' (Figure 3). | Greuter *et al.* (1989) and Arrigoni & Diana (1999) |
| ***Limonium geronense* Erben** | ‘Mediterranean lineage’* in *Limonium* subg*. Limonium* | The species is part of *L. duriusculum-companyonis* complex. Representatives of this complex such as *L. thiniense* and *L. rigualii* are placed in the 'Mediterranean lineage' (Figure 3). | Sáez & Rosello (1999) |
| ***Limonium gibertii* (Sennen) Sennen** | *‘*Mediterranean lineage’* in *Limonium* subg*. Limonium* | The species is part of *L. delicatulum* complex (Pgnatti 1972), representatives of which (e.g. *L. delicatulum, L. costae, L. tournefortii* etc.) are placed in the 'Mediterranean lineage' (see Figure 3). In addition, *L. gibertii* is phylogenetically related to other Mediterranean taxa in a recent study (constituing the 'Mediterranean lineage'; see Malekmohammadi *et al.* 2017). | Pignatti (1972) and Malekmohammadi *et al.* (2017) |
| ***Limonium ginzbergeri* Bogdanović & Brullo** | ‘Mediterranean lineage’* in *Limonium* subg*. Limonium* | The species is morphologically similar to *L. cancellatum* and other species of *L. cancellatum* group. *Limonium cancellatum* is placed in the 'Mediterranean lineage' (Figure 3) | Bogdanović & Brullo (2015) |
| *Limonium girardianum* (Guss.) Fourr. | ‘Mediterranean lineage’* in *Limonium* subg*. Limonium* | see Figure 3 |  |
| *Limonium globuliferum* (Boiss. & Heldr.) Kuntze | *L.* sect. *Sphaerostachys* in *Limonium* subg. *Limonium* | see Figure 2 |  |
| ***Limonium glomeratum* (Tausch) Erben** | ‘Mediterranean lineage’* in *Limonium* subg*. Limonium* | The species is morphologically similar to other mainly Western Mediterranean taxa and is placed in the *L. densiflorum* group. Some preliminary, partial unpublished sequence data place the species within the 'Mediterranean lineage'. | Pignatti (1982a) and Koutroumpa unpubl. |
| *Limonium gmelini* (Willd.) Kuntze | *L.* sect. *Limonium* in *Limonium* subg. *Limonium* | see Figure 2 |  |
| ***Limonium gobicum* Ikonn.-Gal.** | *L.* sect. *Plathymenium* in *Limonium* subg. *Limonium* | The species is assigned to *L.* sect. *Plathymenium* and is morphologically similar to *L. congestum* and *L. flexuosum* (see *L. flexuosum* in Figure 2) | Ikonnikov-Galitzky (1936) |
| ***Limonium gorgonae* Pignatti** | ‘Mediterranean lineage’* in *Limonium* subg*. Limonium* | The species is part of *L. multiforme* aggregate (*L. cosyrense* group) and thus, morphologically similar to *L. multiforme,* which placed in the 'Mediterranean lineage' (Figure 3). | Pignatti (1982a,b) |
| *Limonium gougetianum* (Girard) Kuntze | ‘Mediterranean lineage’* in *Limonium* subg*. Limonium* | see Figure 3 |  |
| *Limonium grabusae* Erben & Brullo | ‘Mediterranean lineage’* in *Limonium* subg*. Limonium* | see Figure 3 |  |
| *Limonium graecum* (Poir.) Rech.f. | ‘Mediterranean lineage’* in *Limonium* subg*. Limonium* | see Figure 3 |  |
| *Limonium greuteri* Erben | ‘Mediterranean lineage’* in *Limonium* subg*. Limonium* | see Figure 3 |  |
| ***Limonium grosii* L.Llorens** | ‘Mediterranean lineage’* in *Limonium* subg*. Limonium* | The species is morphologically similar to *L. girardianum* which is placed in the 'Mediterranean lineage' (Figure 3). | Erben (1993) |
| ***Limonium grubovii* Lincz.** | *L.* sect. *Plathymenium* in *Limonium* subg. *Limonium* | The species has been assigned to *L.* sect. *Plathymenium* (Linczevski 1971) and is morphologically similar to other taxa of this section (e.g. *L. bicolor*). | Linczevski (1971) |
| *Limonium guaicuru* (Molina) Kuntze | *L.* sect. *Limonium* in *Limonium* subg. *Limonium* | see Figure 2 |  |
| ***Limonium gueneri* Doğan, H. Duman & Akaydın** | *‘*Mediterranean lineage’* in *Limonium* subg*. Limonium* | The species is morphologically similar to *L. ocymifolium,* which is placed in the 'Mediterranean lineage' in the phylogeny (Figure 3). *Limonium gueneri* was also sampled in a recent study and is indeed phylogenetically placed in the clade consisting of Mediterranean taxa (i.e. 'Mediterranean lineage'; see Malekmohammadi *et al.* 2017). | Doğan, Duman & Akaydın (2008) and Malekmohammadi *et al.* (2017) |
| ***Limonium guigliae* Raimondo & Domina** | *L.* sect. *Sarcophyllum* in *Limonium* subg. *Limonium* | The species is morphologically similar to *L. paulayanum* which is placed in the clade of *L.* sect. *Sarcophyllum* (see Figure 2). | Raimondo & Domina (2009) |
| ***Limonium gummiferum* (Durieu ex Boiss. & Reut.) Kuntze** | ‘Mediterranean lineage’* in *Limonium* subg*. Limonium* | The species is morphologiacally similar to other Mediterranean taxa such as *L. battandieri* and *L. cossonianum.* The latter species is placed in the 'Mediterranean lineage' (Figure 3). | Greuter *et al.* (1989) |
| *Limonium gymnesicum* Erben | ‘Mediterranean lineage’* in *Limonium* subg*. Limonium* | see Figure 3 |  |
| ***Limonium halophilum* Pignatti** | ‘Mediterranean lineage’* in *Limonium* subg*. Limonium* | The species is placed in the *L. densiflorum* complex. A represenative of this complex, *Limonium glomeratum* is morphologically and phylogenetically related to other taxa of the 'Mediterranean lineage' (see details above in *L. glomeratum*). | Guarino *et al*. (2017) |
| ***Limonium helenae* Erben & Brullo** | ‘Mediterranean lineage’* in *Limonium* subg*. Limonium* | The species is morphologically similar to *L. aucheri* which is placed in the 'Mediterranean lineage' (Figure 3). | Brullo & Erben (2016) |
| ***Limonium heraionense* Erben & Brullo** | ‘Mediterranean lineage’* in *Limonium* subg*. Limonium* | The species is morphologically similar to *L. frederici* which is placed in the 'Mediterranean lineage' (Figure 3). | Brullo & Erben (2016) |
| ***Limonium hermaeum* Pignatti** | ‘Mediterranean lineage’* in *Limonium* subg*. Limonium* | The species is part of *L. cosyrense* group and thus, morphologically similar to *L. cosyrense,* which is placed in the 'Mediterranean lineage' (Figure 3). | Pignatti (1982a) |
| ***Limonium heterospicatum* Erben** | ‘Mediterranean lineage’* in *Limonium* subg*. Limonium* | The species is morphologically similar to *L. gibertii* which is phylogenetically related to other Mediterranean taxa in a recent study constituing the 'Mediterranean lineage' (" *L. graecum* clade" in Malekmohammadi *et al*. 2017) | Erben (1989) and Malekmohammadi *et al.* (2017) |
| *Limonium hibericum* Erben | ‘Mediterranean lineage’* in *Limonium* subg*. Limonium* | see Figure 3 |  |
| *Limonium hierapetrae* Rech.f. | ‘Mediterranean lineage’* in *Limonium* subg*. Limonium* | see Figure 3 |  |
| ***Limonium himariense* F.K.Mey.** | ‘Mediterranean lineage’* in *Limonium* subg*. Limonium* | The species is part of *L. cosyrense* group (sensu Pignatti 1972). Representatives of this group such as *L. cosyrense* and *L. multiforme* are placed in the 'Mediterranean lineage' (Figure 3). | Meyer (2011) |
| ***Limonium hipponense* Brullo & Erben** | *‘*Mediterranean lineage’* in *Limonium* subg*. Limonium* | The species is morphologically similar to *L. pyramidatum,* which is phylogenetically related to other Mediterranean taxa of the 'Mediterranean lineage' (see in Malekmohammadi *et al.* 2017). | Brullo & Erben (1989) and Malekmohammadi *et al.* (2017) |
| ***Limonium hirsuticalyx* Pignatti** | *L.* sect. *Limonium* in *Limonium* subg. *Limonium* | The species is morphologically similar to other taxa from the re-circumscribed *L.* sect. *Limonium* (i.e. *L.* sect. *Limonium* subsect. *Genuinae*) and is part of *L. vulgare* group (see also Figure 2). | Pignatti (1972) and Brullo & Erben (2016) |
| *Limonium hoeltzeri* (Regel) Ikonn.-Gal. | *L.* sect. *Plathymenium* in *Limonium* subg. *Limonium* | see Figure 2 |  |
| *Limonium humile* Mill. | *L.* sect. *Limonium* in *Limonium* subg. *Limonium* | see Figure 2 |  |
| *Limonium hungaricum* Klokov | *L.* sect. *Limonium* in *Limonium* subg. *Limonium* | see Figure 2 |  |
| ***Limonium hyblaeum* Brullo** | ‘Mediterranean lineage’* in *Limonium* subg*. Limonium* | see Figure 3 |  |
| ***Limonium hypanicum* Klokov** | *L.* sect. *Limonium* in *Limonium* subg. *Limonium* | The species is morphologically similar to *L. gmelini* which is placed in the newly circumscribed *L.* sect. *Limonium* (see clade of *L.* sect. *Limonium* subsect. *Genuinae* in Figure 2)*.* | Pignatti (1972) |
| *Limonium iconicum* (Boiss. & Heldr.) Kuntze | *L.* sect. *Nephrophyllum s.l.* in *Limonium* subg. *Limonium* | see Figure 2 |  |
| ***Limonium ikaricum* Erben & Brullo** | *‘*Mediterranean lineage’* in *Limonium* subg*. Limonium* | The species is morphologically similar to other Aegean endemics (East Mediterranean) such as *L. quinnii* and *L. elaphonisicum.* The latter species is placed in the 'Mediterranean lineage' (Figure 3). | Brullo & Erben (2016) |
| ***Limonium ilergabonum* López-Alvarado, Cobacho, Arán & L. Sáez** | *‘*Mediterranean lineage’* in *Limonium* subg*. Limonium* | The species is morphologically similar to *L. hibericum* which is placed in the 'Mediterranean lineage' (Figure 3). | López-Alvarado *et al.* (2017) |
| ***Limonium ilvae* Pignatti** | ‘Mediterranean lineage’* in *Limonium* subg*. Limonium* | The species is part of *L. multiforme* aggregate (*L. cosyrense* group) and thus, morphologically similar to *L. multiforme* which is part of the 'Mediterranean lineage' (Figure 3). | Pignatti (1982a,b) |
| *Limonium imbricatum* (Webb ex Girard) Hubbard ex L.H.Bailey | *L.* sect. *Pteroclados* subsect. *Nobiles* in *Limonium* subg. *Pteroclados s.l.* | see Figure 2 |  |
| ***Limonium inarimense* (Guss.) Pignatti** | ‘Mediterranean lineage’* in *Limonium* subg*. Limonium* | The species is morphologically similar to *L. minutiflorum* which is placed in the 'Mediterranean lineage' (Figure 3). | Pignatti (1982a) |
| ***Limonium inexpectans*L.Sáez & Rosselló** | ‘Mediterranean lineage’* in *Limonium* subg*. Limonium* | The species is morphologically similar to *L. gibertii* which is phylogenetically related to other Mediterranean taxa in a recent phylogenetic study (constituing the 'Mediterranean lineage' - " *L. graecum* clade" in Malekmohammadi *et al.* 2017) | Sáez & Rosselló (1996) and Malekmohammadi *et al.* (2017) |
| *Limonium insigne* (Coss.) Kuntze | *L.* sect. *Polyarthrion* in *Limonium* subg. *Limonium* | see Figure 3 |  |
| ***Limonium insulare* (Bég. & Landi) Arrigoni & Diana** | ‘Mediterranean lineage’* in *Limonium* subg*. Limonium* | The species is part of *L. virgatum* complex and is similar to representatives of this complex such as *L. virgatum, L. dubium, L. algusae* etc. *Limonium virgatum* is placed in the 'Mediterranean lineage' (Figure 3). | Guarino *et al.* (2017) |
| ***Limonium interjectum*J.X.Soler & Rosselló** | ‘Mediterranean lineage’* in *Limonium* subg*. Limonium* | The species is morphologically similar to *L. virgatum* and *L. girardianum* (both placed in the ‘Mediterranean lineage’)*,* and phylogenetically the species is related to Mediterranean taxa of the 'Mediterranean lineage' (see Figure 3 and Palacios *et al.* 2000). | Soler & Rosselló (1997) and Palacios *et al.* (2000) |
| ***Limonium intermedium* (Guss.) Brullo** | *‘*Mediterranean lineage’* in *Limonium* subg*. Limonium* | The species is morphologically similar to *L. albidum* and is part of *L. albidum* group. *Limonium hyblaeum* which is a representative of *L. albidum* group is placed in the 'Mediterranean lineage’ (Figure 3). | Brullo & Pavone (1981) and Pignatti (1982a) |
| ***Limonium intricatum* Brullo & Erben** | ‘Mediterranean lineage’* in *Limonium* subg*. Limonium* | The species is part of *L. articulatum* aggregate. *Limonium articulatum* and other related taxa are placed in the 'Medieterranean lineage' (Figure 3). | Brullo & Erben (1989) and Véla & Pavon (2013) |
| ***Limonium ionicum* Brullo** | *‘*Mediterranean lineage’* in *Limonium* subg*. Limonium* | The species is morphologically similar to *L. minutiflorum* (*L. minutiflorum* group) which is placed in the 'Mediterranean lineage' (Figure 3). | Pignatti (1982a) |
| *Limonium iranicum* (Bornm.) Lincz. | *L.* sect. *Iranolimon* in *Limonium* subg. *Limonium* | see Figure 2 |  |
| ***Limonium irtaense* P.P.Ferrer, A.Navarro, P.Pérez, R.Roselló, Rosselló, M.Rosato & E.Laguna** | ‘Mediterranean lineage’* in *Limonium* subg*. Limonium* | The species is part of *L. delicatulum* agregate. Representatives of this group such as *L. delicatulum, L. santapolense, L. biflorum, L. scopulorum* etc. are all placed in the 'Mediterranean lineage' (Figure 3). | Ferrer-Gallego *et al.* (2015) and Moreno *et al.* (2018) |
| ***Limonium isidorum* Erben & Brullo** | ‘Mediterranean lineage’* in *Limonium* subg*. Limonium* | The species is morphologically similar to *L. aucheri* which is placed in the 'Mediterranean lineage' (Figure 3). | Brullo & Erben (2016) |
| ***Limonium issaeum* Bogdanović & Brullo** | ‘Mediterranean lineage’* in *Limonium* subg*. Limonium* | The species is morphologically similar to *L. cancellatum* and other species of *L. cancellatum* group. *Limonium cancellatum* is part of the 'Mediterranean lineage' (Figure 3). | Bogdanović & Brullo (2015) |
| ***Limonium istriacum* Bogdanović & Brullo** | ‘Mediterranean lineage’* in *Limonium* subg*. Limonium* | The species is morphologically similar to *L. cancellatum* and other species of *L. cancellatum* group. *Limonium cancellatum* is placed in the 'Mediterranean lineage' (Figure 3). | Bogdanović & Brullo (2015) |
| ***Limonium ithacense* R.Artelari** | ‘Mediterranean lineage’* in *Limonium* subg*. Limonium* | The species is morphologically similar to *L. coronense* and *L. saracinatum.* Both species are placed in the 'Mediterranean lineage' (Figure 3). | Artelari (1984) and Brullo & Erben (2016) |
| ***Limonium jankae* (Lojac.) Giardina & Raimondo** | ‘Mediterranean lineage’* in *Limonium* subg*. Limonium* | The species is morphologically similar to other Mediterranean taxa, such as *L. inarimese* and *L. lojaconoi* (species similar to *L. minutiflorum*), *L.dubium* and *L. virgatum. Limonium virgatum* and *L. minutiflorum* are both placed in the 'Mediterranean lineage' (Figure 3). | Lojacono (1907) |
| ***Limonium japygicum* (H. Groves) Pignatti** | ‘Mediterranean lineage’* in *Limonium* subg*. Limonium* | The species is part of *L. cancellatum* group and is morphologically similar to *L. cancellatum* which is placed in the 'Mediterranean lineage' (Figure 3). | Guarino *et al*. (2017) |
|    \| ***Limonium jarmolenkoi* (Lincz.) M.Malekm., Akhani & Borsch** \| \| --- \| | *L.* sect. *Siphonocalyx* in *Limonium* subg. *Limonium* | The species was previously assigned to genus *Eremolimon* (*Eremolimon jarmolenkoi* Lincz.) which is currently embeded in *Limonium.* All represenatives of *Eremolimon* currently comprise *L.* sect. *Siphonocalyx* (see represenative in Figure 2). | Malekmohammadi *et al*. (2017) |
| ***Limonium johannis* Pignatti** | ‘Mediterranean lineage’* in *Limonium* subg*. Limonium* | The species is morphologically similar to *L. cumanum* which is phylogenetically closely related to other Mediterranean taxa in a recent phylogenetic work ( *L. cumanum* belongs to the 'Mediterranean lineage'/”*L. graecum* clade” in Malekmohammadi *et al.* 2017). | Pignatti (1982a) and Malekmohammadi *et al.* (2017) |
| *Limonium jovibarba* (Webb) Kuntze | *L.* sect. *Jovibarba* in *Limonium* subg. *Limonium* | see Figure 2 |  |
| ***Limonium kairouanum* Brullo & Erben** | ‘Mediterranean lineage’* in *Limonium* subg*. Limonium* | The species is morphologically similar to *L. menigense, L. cercinense,* *L. rubescens* and *L. delicatulum. Limonium delicatulum* is placed in the 'Mediterranean lineage' (Figure 3). | Brullo & Erben (1989) |
| *Limonium kardamylii* R.Artelari & Kamari | ‘Mediterranean lineage’* in *Limonium* subg*. Limonium* | see Figure 3 |  |
| *Limonium kaschgaricum* (Rupr.) Ikonn.-Gal. | *L.* sect. *Plathymenium* in *Limonium* subg. *Limonium* | see Figure 2 |  |
| ***Limonium kimmericum* (Lipsky) Klokov** | *L.* sect. *Nephrophyllum s.l.* in *Limonium* subg. *Limonium* | The species was formerly *Statice caspia* Willd. var. *kimmerica* and is morphologically similar to *L. caspium,* which is part of the clade of *L.* sect. *Nephrophyllum s.l.* (see Figure 2). | Tzvelev (2012) |
| ***Limonium kirikosicum* Erben & Brullo** | ‘Mediterranean lineage’* in *Limonium* subg*. Limonium* | The species is morphologically similar to *L. meandrinum,* which is placed in the 'Mediterranean lineage' (Figure 3). | Brullo & Erben (2016) |
| ***Limonium klementzii* Ikonn.-Gal.** | *L.* sect. *Plathymenium* in *Limonium* subg. *Limonium* | The species has been assigned to *L.* sect. *Plathymenium* (Ikonnikov-Galitzky 1936) and is morphologically similar to *L. chrysocomum*(of the same section*.* | Ikonnikov-Galitzky (1936) |
| ***Limonium kobstanicum* Tzvel.** | *L.* sect. *Iranolimon Limonium* subg. *Limonium* | The species is assigned to *L.* sect. *Iranolimon* as it shares morphological similarities with its representatives. | Malekmohammadi *et al*. (2017) |
| ***Limonium komarovii* Ikonn.-Gal. ex Lincz. & Czukav.** | *L.* sect. *Siphonocalyx s.l.* in *Limonium* subg. *Limonium* | The species is morphologically similar to *L. ferganense. Limonium ferganense* is phylogenetically related to *L.* sect. *Siphonocalyx* and shares some morphological similarities with its representatives. In a recent study *L. ferganense* was sister to *L. sogdianum* and *L. piptopodum* of *L.* sect. *Siphonocalyx,* all three of them comprising the "*L. sogdianum* clade" (see Malekmohammadi *et al.* 2017). | Linczevski & Czukavina (1984) and Malekmohammadi *et al*. (2017) |
| ***Limonium korakonisicum* R.Artelari & Valli** | ‘Mediterranean lineage’* in *Limonium* subg*. Limonium* | The species is closely related to *L. spreitzenhoferi* which is placed in the 'Mediterranean lineage' (Figure 3). | Valli & Artelari (2015) |
| ***Limonium korbousense* Brullo & Erben** | *‘*Mediterranean lineage’* in *Limonium* subg*. Limonium* | The species is a triploid, morphologically similar to other Mediterranean taxa, such as *L. cercinense, L. punicum,* *L. delicatulum* etc*. Limonium delicatulum* is placed in the 'Mediterranean lineage' in the phylogeny (Figure 3). | Brullo & Erben (1989) |
| *Limonium kraussianum* (Buchinger ex Boiss.) Kuntze | ‘Mediterranean lineage’* in *Limonium* subg*. Limonium* | see Figure 3 |  |
|    \| ***Limonium kurgantjubense* (Lincz.) M.Malekm., Akhani & Borsch** \| \| --- \| | *L.* sect. *Siphonocalyx* in *Limonium* subg. *Limonium* | The species was previously assigned to genus *Eremolimon* (*Eremolimon kurgantjubense* Lincz.) which is currently embeded in *Limonium.* All represenatives of *Eremolimon* currently comprise *L.* sect. *Siphonocalyx* (Malekmohammadi et al. 2017; see also represenative in Figure 2). | Malekmohammadi et al. (2017) |
| ***Limonium lacertosum* Brullo & Erben** | ‘Mediterranean lineage’* in *Limonium* subg*. Limonium* | The species is morphologically similar to *L. densiflorum, L. clupeanum* and other representatives of *L. densiflorum* complex (sensu Guarino *et al.* 2017). *Limonium glomeratum* as a representative of this complex is phylogenetically related to other taxa of the 'Mediterranean lineage' (see details above in *L. glomeratum*). | Brullo & Erben (1989) |
| ***Limonium lacinium* Arrigoni** | ‘Mediterranean lineage’* in *Limonium* subg*. Limonium* | The species is part of *L. cancellatum* group and is morphologically similar to *L. cancellatum* which is placed in the 'Mediterranean lineage' (Figure 3). | Guarino *et al*. (2017) |
| ***Limonium lacostei* (Danguy) Kamelin** | *L.* sect. *Plathymenium* in *Limonium* subg. *Limonium* | The species is assigned to *L.* sect. *Plathymenium* and is morphologically similar to *L. tenellum* and *L. kaschgaricum* of the same section (see Figure 2). | Ikonnikov-Galitzky (1936) under syn. *L. roborowskii* |
| ***Limonium laetum* (Nyman) Pignatti** | *‘*Mediterranean lineage’* in *Limonium* subg*. Limonium* | The species is morphologically similar to *L. inarimense* and *L. minutiflorum.* The latter species is placed in the 'Mediterranean lineage' (Figure 3). | Pignatti (1982a) and Iamonico, Vallariello, & Guacchio (2017) |
| ***Limonium lagostanum* Bogdanović & Brullo** | ‘Mediterranean lineage’* in *Limonium* subg*. Limonium* | The species is morphologically similar to *L. cancellatum* and other species of *L. cancellatum* group. *Limonium cancellatum* is placed in the 'Mediterranean lineage' (Figure 3). | Bogdanović & Brullo (2015) |
| ***Limonium lambinonii* Erben** | ‘Mediterranean lineage’* in *Limonium* subg*. Limonium* | The species is morphologically similar to *L. multiforme* and *L. gorgonae* (*L. multiforme* aggregate). The former species and other representatives from the same aggregate are placed in the 'Mediterranean lineage' (Figure 3). | Erben (2002) |
| *Limonium lanceolatum* (Hoffmanns. & Link) Franco | ‘Mediterranean lineage’* in *Limonium* subg*. Limonium* | see Figure 3 |  |
| *Limonium latebracteatum* Erben | ‘Mediterranean lineage’* in *Limonium* subg*. Limonium* | see Figure 3 |  |
| *Limonium latifolium* (Sm.) Kuntze | *L.* sect. *Limonium* in *Limonium* subg. *Limonium* | see Figure 2 |  |
| ***Limonium lausianum* Pignatti** | ‘Mediterranean lineage’* in *Limonium* subg*. Limonium* | The species is morphologically similar to the Mediterranean taxa *L. sibthorpianum, L. optimae, L. capitis-marci* etc. and together with *L. mucronulatum* are part of *L. sibthorpianum* complex. *Limonium mucronulatum* was recently sampled in a phylogenetic study and is confidently placed within the 'Mediterranean lineage' (see "*L. graecum* clade" in Malekmohammadi *et al.* 2017). | Malekmohammadi *et al.* (1989) and Guarino *et al.* (2017) |
| *Limonium laxiusculum* Franco | ‘Mediterranean lineage’* in *Limonium* subg*. Limonium* | see Figure 3 |  |
| ***Limonium legrandii* (Gaut. & Timb.-Lagr.) Erben** | ‘Mediterranean lineage’* in *Limonium* subg*. Limonium* | The species is morphologically similar to *L. auriculae-ursifolium, L. dodartii* and is part of *L. dodartii* group. The aforementioned taxa are all placed in the 'Mediterranean lineage' (Figure 3). | Pavon (2005) and Tison *et al.* (2014) |
| ***Limonium leonardi-llorensii* L.Sáez, Á.C.Carvalho & Rosselló** | ‘Mediterranean lineage’* in *Limonium* subg*. Limonium* | The species is morphologically similar to *L. marisolii* that together with *L. giberii* are part of *L. gibertii* aggregate. The latter species is phylogenetically related to other Mediterranean taxa in a recent study constituing the 'Mediterranean lineage' (" *L. graecum* clade" in Malekmohammadi *et al.* 2017). | Sáez & Rosselló (1996), Sáez *et al.* (1998a) and Malekmohammadi *et al.* (2017) |
| ***Limonium leprosorum* Bogdanović & Brullo** | ‘Mediterranean lineage’* in *Limonium* subg*. Limonium* | The species is morphologically similar to *L. cancellatum* and other species of *L. cancellatum* group. *Limonium cancellatum* is placed in the 'Mediterranean lineage' (Figure 3). | Bogdanović & Brullo (2015) |
| ***Limonium leptolobum* (Regel) Kuntze** | *L.* sect. *Plathymenium* in *Limonium* subg. *Limonium* | The species is morphologically similar to *L. dichroanthum* (see Figure 2) and is assigned to *L.* sect. *Plathymenium.* | Linczevski (1952) |
| ***Limonium leptophyllum* (Schrenk) Kuntze** | *L.* sect. *Iranolimon* in *Limonium* subg. *Limonium* | The species is assigned to *L.* sect. *Iranolimon* as it shares morphological similarities to its representatives. | Malekmohammadi *et al*. (2017) |
| ***Limonium letourneuxii* (Batt.) Greuter & Burdet** |  | The species is a narrow endemic in Algeria in the North Mediterranean coast, growing on rocks and growing in very dense clumps. This particular branchy leafy habit is also present in *L. acutifolium* complex (sensu Guarino *et al.* 2017). However, the inflorescence is not similar to the ones of the aforementioned complex. In general, the species show morphological similarities to other Mediterranean endemics but we were not able to find a very close relative. The species is possible assigned to the 'Mediterranean lineage', but it needs further examination to be more or less confident about this assignement. | |
| ***Limonium liberianum* Bogdanović & Brullo** | ‘Mediterranean lineage’* in *Limonium* subg*. Limonium* | The species is morphologically similar to *L. cancellatum* and other species of *L. cancellatum* group. *Limonium cancellatum* is placed in the 'Mediterranean lineage' (Figure 3). | Bogdanović & Brullo (2015) |
| ***Limonium liburnicum* Lovrić ex Bogdanović & Brullo** | ‘Mediterranean lineage’* in *Limonium* subg*. Limonium* | The species is morphologically similar to *L. cancellatum* and other species of *L. cancellatum* group. *Limonium cancellatum* is placed in the 'Mediterranean lineage' (Figure 3). | Bogdanović & Brullo (2015) |
| *Limonium lilacinum* (Boiss. & Bal.) Wagenitz | *L.* sect. *Sphaerostachys* in *Limonium* subg. *Limonium* | see Figure 2 |  |
| ***Limonium lilybaeum* Brullo** | ‘Mediterranean lineage’* in *Limonium* subg*. Limonium* | The species is part of *L. densissimum* morphological aggregate. *Limonium densissimum* is placed in the 'Mediterranean lineage' (Figure 3). | Pignatti (1982a) |
| *Limonium limbatum* Small | *L.* sect. *Limonium* in *Limonium* subg. *Limonium* | see Figure 2 |  |
| ***Limonium linifolium* Kuntze** | ‘Mediterranean lineage’* in *Limonium* subg*. Limonium* | The species is morphologically similar to *L. scabrum* which is placed in the 'Mediterranean lineage' (Figure 3). | Dyer (1963) |
| *Limonium lobatum* (L.f.) Chaz. | *L.*sect. *Pteroclados* subsect. *Odontolepideae* in *Limonium* subg. *Pteroclados s.l.* | see Figure 2 |  |
| ***Limonium lobetanicum* Erben** | ‘Mediterranean lineage’* in *Limonium* subg*. Limonium* | The species is a diploid endemic species of Spain that is part of *L. cofrentanum* complex (sensu Erben 1989) and shares morphological similarities with *L. cofrentanum, L. sucronicum* and *L. aragonense*. *Limonium aragonense* is placed in the 'Mediterranean lineage' (Figure 3). | Erben (1989) and Erben (1993) |
| *Limonium lobinii* N.Kilian & Leyens | *L.* sect. *Jovibarba* or *Ctenostachys* in *Limonium* subg. *Limonium* | see Figure 2 |  |
| ***Limonium loganicum* Ingr.** | ‘Mediterranean lineage’* in *Limonium* subg*. Limonium* | The species is morphologically similar to *L. binervosum* (part of *L. binervosum* aggregate) which is placed in the 'Mediterranean lineage' (Figure 3). | Pignatti (1972) and Ingrouille & Stace (1986) |
| ***Limonium lojaconoi* Brullo** | ‘Mediterranean lineage’* in *Limonium* subg*. Limonium* | The species is part of *L. minutiflorum* aggregate. *Limonium minutiflorum* is placed in the 'Mediterranean lineage' (Figure 3). | Greuter *et al.* (1989) and Domina & Mazzola (2003) |
| *Limonium longebracteatum* Erben | ‘Mediterranean lineage’* in *Limonium* subg*. Limonium* | see Figure 3 |  |
| ***Limonium longifolium* (Thunb.) R.A.Dyer** | *L.* sect. *Circinaria* in *Limonium* subg. *Limonium* | The species was previously assigned to genus *Afrolimon* (*Afrolimon longifolium* (Thunb.) Lincz.) which is currently embeded in *Limonium.* All represenatives of *Afrolimon* currently comprise *L.* sect. *Circinaria* (Malekmohammadi *et al*. 2017; see represenatives in Figure 2). | Malekmohammadi *et al*. (2017) |
| ***Limonium lopadusanum* Brullo** | ‘Mediterranean lineage’* in *Limonium* subg*. Limonium* | The species is morphologically similar to *L. albidum* and is part of *L. albidum* group. *Limonium hyblaeum* which is a representative of *L. albidum* group is placed in the 'Mediterranean lineage' (Figure 3). | Brullo & Pavone (1981) and Pignatti (1982a) |
| ***Limonium lovricii* Bogdanović & Brullo** | ‘Mediterranean lineage’* in *Limonium* subg*. Limonium* | The species is morphologically similar to *L. cancellatum* and other species of *L. cancellatum* group. *Limonium cancellatum* is placed in the 'Mediterranean lineage' (Figure 3) | Bogdanović & Brullo (2015) |
| *Limonium lowei* R.Jardim, M.Seq., Capelo, J.C.Costa & Rivas Mart. | ‘Mediterranean lineage’* in *Limonium* subg*. Limonium* | see Figure 3 |  |
| ***Limonium lucentinum* Pignatti & Freitag** | ‘Mediterranean lineage’* in *Limonium* subg*. Limonium* | The species is morphologically similar to *L. furfuraceum* and it was formerly a subspecies of it (*Limonium furfuraceum* subsp. *lucentinum* (Pignatti & Freitag) O. de Bolòs & J. Vigo). *Limonium furfuraceum* is placed in the 'Mediterranean lineage' (Figure 3). | Pignatti (1972) and Erben (1978) |
| *Limonium macrophyllum* Kuntze | *L.* sect. *Pteroclados* subsect. *Nobiles* in *Limonium* subg. *Pteroclados s.l.* | see Figure 2 |  |
| *Limonium macropterum* (Webb & Berthel.) Kuntze | *L.* sect. *Pteroclados* subsect. *Nobiles* in *Limonium* subg. *Pteroclados s.l.* | see Figure 2 |  |
| ***Limonium macrorrhizon* (Ldb.) Kuntze** | *L.* sect. *Nephrophyllum s.l.* in *Limonium* subg. *Limonium* | The species is morphologically similar to other representatives of *L.* sect. *Nephrophyllum s.l.* especially from *L. bellidifolium* complex (see also Figure 2 and Taxonomic proposals). | Linczevski (1952) |
| ***Limonium magallufianum* L.Llorens** | ‘Mediterranean lineage’* in *Limonium* subg*. Limonium* | The species is morphologically similar to other Mediterranean taxa of *L. delicatulum* complex such as *L. marisolii, L. ejulabilis, L. cossonianum* etc*.* Representatives of this complex are all placed in the 'Mediterranean lineage' (see e.g. *L. biflorum, L. delicatulum, L. cossonianum* in Figure 3). | Rosselló, Amézquita, & Marí (1993) and Erben (1993) |
| ***Limonium majoricum* Pignatti** | ‘Mediterranean lineage’* in *Limonium* subg*. Limonium* | The species is morphologically similar to *L. gymnesicum* which is placed in the 'Mediterranean lineage' (Figure 3). | Erben (1993) |
| *Limonium majus* (Boiss.) Erben | ‘Mediterranean lineage’* in *Limonium* subg*. Limonium* | see Figure 3 |  |
| ***Limonium malacitanum* B.Díez** | ‘Mediterranean lineage’* in *Limonium* subg*. Limonium* | The species is assigned to *L. auriculae-ursifolium* aggregate and is morphologically closer to *L. auriculae-ursifolium* and *L. algarvense*; both taxa are placed in the 'Mediterranean lineage' (Figure 3). | Diez Garretas (1981) and Greuter (1989) |
| ***Limonium malfatanicum* Erben** | ‘Mediterranean lineage’* in *Limonium* subg*. Limonium* | The species is morphologically similar to *L. duriusculum.* Representatives of *L. duriusculum* complex such as *L. thiniense* and *L. rigualii* are placed in the 'Mediterranean lineage' in the phylogeny (Figure 3). | Erben (2001) |
|  |  |  | |
| ***Limonium mansanetianum* M.B.Crespo & Lledó** | ‘Mediterranean lineage’* in *Limonium* subg*. Limonium* | The species is morphologically and phylogenetically related to other taxa especesially from Western Mediterranean (Lledó *et al.* 2005; currently assigned to 'Mediterranean lineage'). | Lledó *et al.* (2005) |
| ***Limonium marisolii* L.Llorens** | ‘Mediterranean lineage’* in *Limonium* subg*. Limonium* | The species is morphologically similar to *L. gibertii* which is phylogenetically related to other Mediterranean taxa in a recent study (constituing the 'Mediterranean lineage'; see "*L. graecum* clade" in Malekmohammadi *et al.* 2017). | Sáez & Rosselló (1996) and Malekmohammadi *et al.* (2017) |
| ***Limonium marmarisense* Doğan & Akaydın** | *L.* sect. *Limonium* in *Limonium* subg. *Limonium* | The species is morphologically similar to *L. narbonense* (syn. *L. angustifolium* (Tausch)Turril), which is placed in the re-circumscribed *L.* sect. *Limonium* (see also Figure 2). | Doğan & Akaydın (2017) |
| ***Limonium maroccanum* (Batt. & Trab.) Domina** | *L.* sect. *Siphonantha* in *Limonium* subg. *Limonium* | The species is morphologically similar to *L. tubiflorum* (*L.* sect. *Siphonantha,* see Figure 3) and it was formerly ranked as a subspecies of *L. tubiflorum* (i.e. *Limonium tubiflorum* subsp. *maroccanum* (Batt. & Trabut) Maire & Weiller). | Maire (1936) and Greuter & Raab-Straube (2011) |
| ***Limonium mateoi* Erben & Arán** | ‘Mediterranean lineage’* in *Limonium* subg*. Limonium* | The species is morphologically similar to *L. dichotomum* and *L. erectum,* which are placed in the 'Mediterranean lineage' (Figure 3) | Erben & Arán (2005) |
| ***Limonium maurocordatae*(Volkens & Schweinf.) Cufod.** | *L.* sect. *Sarcophyllum* in *Limonium* subg. *Limonium* | The species is morphologically similar to other representatives of *L.* sect. *Sarcophyllum* such as *L. cylindrifolium* (see also Figure 2 and Taxonomic proposals). | Thulin (2006) |
| ***Limonium mazarae* Pignatti** | ‘Mediterranean lineage’* in *Limonium* subg*. Limonium* | The species is morphologically similar to *L. albidum* and is part of *L. albidum* group. *Limonium hyblaeum* which is a representative of *L. albidum* group that is placed in the 'Mediterranean lineage' in the phylogeny (Figure 3). | Brullo & Pavone (1981) and Pignatti (1982a) |
| *Limonium meandrinum* Erben & Brullo | ‘Mediterranean lineage’* in *Limonium* subg*. Limonium* | see Figure 3 |  |
| ***Limonium melancholicum* Brullo, Marcenò & S.Romano** | ‘Mediterranean lineage’* in *Limonium* subg*. Limonium* | The species is part of *L. minutiflorum* aggregate and morphologically similar to *L. minutiflorum, L. lojaconoi* etc. *Limonium minutiflorum* is placed in the 'Mediterranean lineage' (Figure 3). | Brullo *et al.* (1996), Colombo (2002) and Domina & Mazzola (2003) |
| ***Limonium melitense* Brullo** | ‘Mediterranean lineage’* in *Limonium* subg*. Limonium* | The species is morphologically similar to *L. bocconei* (part of *L. bocconei* aggregate) which is placed in the 'Mediterranean lineage' (Figure 3). | Brullo (1988) |
| ***Limonium menigense* Brullo & Erben** | ‘Mediterranean lineage’* in *Limonium* subg*. Limonium* | The species is morphologically similar to *L. cercinense,* *L. rubescens* and *L. delicatulum. Limonium delicatulum* is placed in the 'Mediterranean lineage' (Figure 3). | Brullo & Erben (1989) |
| ***Limonium merxmuelleri* Erben** | ‘Mediterranean lineage’* in *Limonium* subg*. Limonium* | The species is morphologically similar to other Mediterranean taxa especially the diploids *L. retirameum, L. sulcitanum* etc. and is part of *L. virgatum* aggregate. *Limonium virgatum* is placed in the 'Mediterranean lineage' (Figure 3). | Greuter *et al.* (1989) |
| ***Limonium messeniacum* R.Artelari & Kamari** | ‘Mediterranean lineage’* in *Limonium* subg*. Limonium* | The species is morphologically similar to *L. kardamylii* which is placed in the 'Mediterranean lineage' (Figure 3). | Artelari & Kamari (2000) and Brullo & Erben (2016) |
| *Limonium meyeri* (Boiss.) Kuntze | *L.* sect. *Limonium* in *Limonium* subg. *Limonium* | see Figure 2 |  |
| ***Limonium michelsonii* Lincz.** | *L.* sect. *Plathymenium* in *Limonium* subg. *Limonium* | The species is morphologically similar to *L. leptolobum* and *L. dichroanthum* (see Figure 2) and is assigned to *L.* sect. *Plathymenium* (Linczevski 1952)*.* | Linczevski (1952) |
| ***Limonium microcycladicum* Erben & Brullo** | ‘Mediterranean lineage’* in *Limonium* subg*. Limonium* | The species is morphologically similar to other Mediterranean species, especially endemics of the Aegean region, such as *L. fragile, L. dolihense* and *L. xerocamposicum. Limonium xerocamposicum* is placed in the 'Mediterranean lineage' (Figure 3). | Brullo & Erben (2016) |
| ***Limonium migjornense* L.Llorens** | ‘Mediterranean lineage’* in *Limonium* subg*. Limonium* | The species is morphologically similar to *L. magallufianum* and other Mediterranean taxa of *L. delicatulum* complex. Representatives of this complex (e.g. *L. delicatulum, L. biflorum* etc.) are all placed in the 'Mediterranean lineage' (Figure 3) | Erben (1993) and Sáez & Rosselló (1996) |
| *Limonium milleri*Ghaz. & J.R.Edm. | *L.* sect. *Sarcophyllum* in *Limonium* subg. *Limonium* | see Figure 2 |  |
| ***Limonium milovicii* Bogdanović & Brullo** | ‘Mediterranean lineage’* in *Limonium* subg*. Limonium* | The species is morphologically similar to *L. cancellatum* and other species of *L. cancellatum* group. *Limonium cancellatum* is placed in the 'Mediterranean lineage' (Figure 3). | Bogdanović & Brullo (2015) |
| *Limonium minoicum* Erben & Brullo | ‘Mediterranean lineage’* in *Limonium* subg*. Limonium* | see Figure 3 |  |
| ***Limonium minoricense* Erben** | ‘Mediterranean lineage’* in *Limonium* subg*. Limonium* | The species is morphologically similar to other Mediterranean taxa especially from Western Mediterranean such as *L. majoricum* and *L. gymnesicum.* The latter species is placed in the 'Mediterranean lineage' (Figure 3). | Erben (1993) |
| ***Limonium minus* (Boiss.) Erben** | ‘Mediterranean lineage’* in *Limonium* subg*. Limonium* | The species is morphologically similar to other Mediterranean taxa such as *L. salsuginosum, L. supinum* and *L. subglabrum. Limonium supinum* and *L. subglabrum* are placed in the 'Mediterranean lineage' (Figure 3). | Greuter *et al.* (1989) and Erben (1993) |
| *Limonium minutiflorum* (Guss.) Kuntze | ‘Mediterranean lineage’* in *Limonium* subg*. Limonium* | see Figure 3 |  |
| *Limonium minutum* (L.) Chaz. | ‘Mediterranean lineage’* in *Limonium* subg*. Limonium* | see Figure 3 |  |
| ***Limonium monolithicum* Erben & Brullo** | ‘Mediterranean lineage’* in *Limonium* subg*. Limonium* | The species is morphologically close to *L. sitiacum* and *L. amopicum.* Both species are placed in the 'Mediterranean lineage' (Figure 3). | Brullo & Erben (2016) |
| ***Limonium montis-christi* M.Rizzotto** | ‘Mediterranean lineage’* in *Limonium* subg*. Limonium* | The species is part of *L. multiforme* aggregate. Representatives of this group (e.g. *L. multiforme* and *L. remotispiculum*) are placed in the 'Mediterranean lineage' (Figure 3). | Rizzotto, M. (1999) and and Bogdanović & Brullo (2015) |
| ***Limonium morisianum* Arrigoni** | *‘*Mediterranean lineage’* in *Limonium* subg*. Limonium* | The species is part of *L. cosyrense* group and thus, morphologically similar to *L. cosyrense* which is placed in the 'Mediterranean lineage' (Figure 3). | Pignatti (1982a) |
| *Limonium mouretii* (Pitard) Maire | *L.*sect. *Pteroclados* subsect. *Odontolepideae* in *Limonium* subg. *Pteroclados s.l.* | see Figure 2 |  |
| ***Limonium mouterdei* Domina, Erben & Raimondo** | ‘Mediterranean lineage’* in *Limonium* subg*. Limonium* | The species is morphologically similar to *L. graecum* and *L. galilaeum. Limonium graecum* is placed in the 'Mediterranean lineage' (Figure 3). | Domina *et al.* (2008) |
| *Limonium mucronatum* (L.f.) Chaz. | *L.* sect. *Ctenostachys* in *Limonium* subg. *Limonium* | see Figure 2 |  |
| ***Limonium mucronulatum* (H.Lindb.) Greuter & Burdet** | *‘*Mediterranean lineage’* in *Limonium* subg*. Limonium* | The species is morphologically similar to the Mediterranean taxa *L. sibthorpianum, L. raddianum, L. teuchirae,* *L. vaccari* etc. and together with *L. mucronulatum* are part of *L. sibthorpianum* aggregate. *Limonium mucronulatum* was recently sampled in a phylogenetic study and is confidently placed within the 'Mediterranean lineage' (see "*L. graecum* clade" in Malekmohammadi *et al.* 2017). | Kouzali *et al*. (2012) and Malekmohammadi *et al.* (2017) |
| ***Limonium multiceps* (Pomel) Erben** | ‘Mediterranean lineage’* in *Limonium* subg*. Limonium* | The species is morphologically similar to *L. gougetianum* which is placed in the 'Mediterranean clade' (Figure 3). *Limonium multiceps* was formerly a subspecies of *L. gougetianum* (*Limonium gougetianum* subsp. *multiceps* (Pomel) Greuter & Burdet). | Greuter *et al.* (1989) and Erben (2012) |
| *Limonium multiflorum* Erben | ‘Mediterranean lineage’* in *Limonium* subg*. Limonium* | see Figure 3 |  |
| *Limonium multiforme* (Martelli) Pignatti | *‘*Mediterranean lineage’* in *Limonium* subg*. Limonium* | see Figure 3 |  |
| ***Limonium multifurcatum* Erben** | ‘Mediterranean lineage’* in *Limonium* subg*. Limonium* | The species is part of *L. calcarae* complex which includes *L. calcarae, L. racemosum, L. multifurcatum, L. dichotomum* and *L. toletanum*. *Limonium dichotomum* and *L. toletanum* are placed in the 'Mediterranean lineage' (Figure 3). | Guarino *et al.* (2017) |
| ***Limonium muradense* Erben** | ‘Mediterranean lineage’* in *Limonium* subg*. Limonium* | The species is morphologically similar to other Mediterranean species, mostly from Baleares, such as *L. caprariense* and *L. minutum.* The latter species is placed in the 'Mediterranean lineage' (Figure 3). | Erben (1993) |
| ***Limonium myrianthum* (Schrenk) Kuntze** | *L.* sect. *Nephrophyllum s.l.* in *Limonium* subg. *Limonium* | The species is morphologically similar to other representatives of *L.* sect. *Nephrophyllum s.l.* (see also Figure 2 and Taxonomic proposals). | Linczevski (1952) |
| ***Limonium namaquanum* L. Bolus** | *L.* sect. *Circinaria* in *Limonium* subg. *Limonium* | The species was previously assigned to genus *Afrolimon* (*Afrolimon namaquanum* (L.Bolus) Lincz.) which is currently embeded in *Limonium.* All represenatives of *Afrolimon* currently comprise *L.* sect. *Circinaria* (Malekmohammadi *et al.* 2017; see represenatives in Figure 2). | Malekmohammadi *et al*. (2017) |
| *Limonium narbonense* Mill. | *L.* sect. *Limonium* in *Limonium* subg. *Limonium* | see Figure 2 |  |
| ***Limonium narynense* Lincz.** | *L.* sect. *Siphonocalyx s.l.* in *Limonium* subg. *Limonium* | The species is morphologically similar to *L. ferganense. Limonium ferganense* is phylogenetically related to *L.* sect. *Siphonocalyx* and shares some morphological similarities with its representatives. In a recent study *L. ferganense* was sister to *L. sogdianum* and *L. piptopodum*, all three of them comprising the "*L. sogdianum* clade". | Linczevski (1985) and Malekmohammadi et al. (2017) |
| ***Limonium neapolense* Brullo & Erben** | ‘Mediterranean lineage’* in *Limonium* subg*. Limonium* | The species is part of *L. delicatulum* group and is morphologically similar to *L. byzacium*. Representatives of *L. delicatulum* group (e.g. *L. delicatulum, L. tournefortii*, *L. biflorum* etc.) are placed in the 'Mediterranean lineage' (Figure 3). | Bartolo, Brullo & Giusso del Galdo (2003) |
| ***Limonium normannicum* Ingr.** | ‘Mediterranean lineage’* in *Limonium* subg*. Limonium* | The species is morphologically similar to *L. auriculae-ursifolium* ( *L. auriculae-ursifolium* complex) which is placed in the 'Mediterranean lineage' (Figure 3). | Ingrouille (1985) |
| *Limonium nudum*  (Boiss. & Buhse) Kuntze | *L.* sect. *Plathymenium* in *Limonium* subg. *Limonium* | see Figure 2 |  |
| *Limonium nydeggeri* Erben | ‘Mediterranean lineage’* in *Limonium* subg*. Limonium* | see Figure 3 |  |
| ***Limonium nymphaeum* Erben** | ‘Mediterranean lineage’* in *Limonium* subg*. Limonium* | The species is part of '*L. acutifolium* gorup' and is morphologically similar to representatives of this group, such as *L. obtusifolium* and *L. bonifaciense*. The aforementioned species are part of the 'Mediterranean lineage' (Figure 3). | Pignatti (1982a) |
| ***Limonium oblanceolatum* Brullo & Erben** | ‘Mediterranean lineage’* in *Limonium* subg*. Limonium* | The species is morphologically similar to *L. densiflorum, L. glomeratum* and other representatives of *L. densiflorum* complex (sensu Guarino *et al.* 2017). *Limonium glomeratum* as a representative of this complex is phylogenetically related to other taxa of the 'Mediterranean lineage' (see details above in *L. glomeratum*). | Brullo & Erben (1989) |
| *Limonium obtusifolium* (Rouy) Erben | ‘Mediterranean lineage’* in *Limonium* subg*. Limonium* | see Figure 3 |  |
| *Limonium ocymifolium* (Poir.) Kuntze | *‘*Mediterranean lineage’* in *Limonium* subg*. Limonium* | see Figure 3 |  |
| *Limonium oligotrichum* Erben & Brullo | ‘Mediterranean lineage’* in *Limonium* subg*. Limonium* | see Figure 3 |  |
| ***Limonium omissae* Bogdanović & Brullo** | ‘Mediterranean lineage’* in *Limonium* subg*. Limonium* | The species is morphologically similar to *L. cancellatum* and other species of *L. cancellatum* group. *Limonium cancellatum* is placed in the 'Mediterranean lineage' (Figure 3). | Bogdanović & Brullo (2015) |
| ***Limonium optimae* Raimondo** | ‘Mediterranean lineage’* in *Limonium* subg*. Limonium* | The species is morphologically similar to the Mediterranean taxa *L. sibthorpianum, L. lausianum, L. vaccarii* etc. and together with *L. optimae* are part of *L. sibthorpianum* aggregate. *Limonium optimae* was recently sampled in a phylogenetic study and is confidently placed within the 'Mediterranean lineage' (see "*L. graecum* clade" in Malekmohammadi *et al.* 2017). | Kouzali *et al*. (2012), Malekmohammadi *et al.* (2017) and Guarino *et al.* (2017) |
| ***Limonium opulentum* (Lojac.) Brullo** | ‘Mediterranean lineage’* in *Limonium* subg*. Limonium* | The species is part of *L. virgatum* aggregate and is similar to representatives of this aggregate such as *L. virgatum, L. dubium, L. algusae* etc. *Limonium virgatum* is placed in the 'Mediterranean lineage' (Figure 3). | Greuter *et al.* (1989) and Guarino *et al.* (2017) |
| ***Limonium orellii* Erben** | ‘Mediterranean lineage’* in *Limonium* subg*. Limonium* | The species is part of *L. duriusculum-companyonis* complex. Representatives of this complex such as *L. thiniense* and *L. rigualii* are placed in the 'Mediterranean lineage' (Figure 3). | Erben (1991) and Sáez & Rosello (1999) |
| ***Limonium oristanum* Alf.Mayer** | ‘Mediterranean lineage’* in *Limonium* subg*. Limonium* | The species is morphologically similar to *L. merxmuelleri, L. sulcitanum, L. retirameum* etc.; part of *L. virgatum* aggregate (sensu Greuter *et al.* 1989). *Limonium virgatum* is placed in the 'Mediterranean lineage' (Figure 3). | Greuter *et al.* (1989) |
| ***Limonium ornatum* (Ball) Kuntze** | *L.* sect. *Polyarthrion* in *Limonium* subg. *Limonium* | The species has been assigned to *L.* sect. *Polyarthrion* and shares morphological similarities with *L. caesium* and *L. insigne* of the same section (see also Figure 3). | Sauvage & Vindt (1952) |
| *Limonium otolepis* (Schrenk) Kuntze | *L.* sect. *Nephrophyllum s.l.* in *Limonium* subg. *Limonium* | see Figure 2 |  |
| ***Limonium oudayense* Sauvage & Vindt** | *L.* sect. *Pruinosum* in *Limonium* subg. *Limonium* | The species is ascribed to *L.* sect. *Pruinosum* (under the former *Statice* sect. *Limonium* subsect. *Pruinosa*) and is morphologically similar to other representatives of this section (see Figure 3). | Sauvage & Vindt (1954) |
| *Limonium ovalifolium* (Poir.) Kuntze | ‘Mediterranean lineage’* in *Limonium* subg*. Limonium* | see Figure 3 |  |
| ***Limonium ovczinnikovii* Lincz. & Czukav.** | *L.* sect. *Siphonocalyx s.l.* in *Limonium* subg. *Limonium* | The species is morphologically similar to *L. ferganense. Limonium ferganense* is phylogenetically related to *L.* sect. *Siphonocalyx* and shares some morphological similarities with its representatives. In a recent study *L. ferganense* was sister to *L. sogdianum* and *L. piptopodum* of *L.* sect. *Siphonocalyx,* all three of them comprising the "*L. sogdianum* clade" (Malekmohammadi *et al.* 2017). | Linczevski & Czukavina (1984) and Malekmohammadi et al. (2017) |
| ***Limonium pachynense* Brullo** | ‘Mediterranean lineage’* in *Limonium* subg*. Limonium* | The species is part of *L. densissimum* morphological aggregate. *Limonium densissimum* is placed in the 'Mediterranean lineage' (Figure 3). | Pignatti (1982a) |
| ***Limonium pagasaeum* Erben & Brullo** | *L.* sect. *Limonium* in *Limonium* subg. *Limonium* | The species is morphologically similar to *L. brevipetiolatum* and *L. narbonense*; both species are part of the re-circumscribed *L.* sect. *Limonium* (see Figure 2). | Brullo & Erben (2016) |
| ***Limonium palmare* (Sm.) Rech.f.** | ‘Mediterranean lineage’* in *Limonium* subg*. Limonium* | The species is morphologically similar to other Eastern Mediterranean taxa, such as *L. graecum* and *L. roridum*, both taxa are placed in the 'Mediterranean lineage' in the phylogeny (Figure 3). | Georgakopoulou *et al.* (2006) |
| *Limonium palmyrense* (Post) Dinsm. | *L.* sect. *Iranolimon* in *Limonium* subg. *Limonium* | see Figure 2 |  |
| ***Limonium pandatariae* Pignatti** | ‘Mediterranean lineage’* in *Limonium* subg*. Limonium* | The species is part of *L. cosyrense* group and thus, morphologically similar to *L. cosyrense,* which is placed in the 'Mediterranean lineage' (Figure 3). | Pignatti (1982a,b) |
| ***Limonium panormitanum* (Tod.) Pignatti** | ‘Mediterranean lineage’* in *Limonium* subg*. Limonium* | The species is morphologically similar to *L. albidum* and is part of *L. albidum* group. *Limonium hyblaeum* which is a representative of *L. albidum* group is placed in the 'Mediterranean lineage' (Figure 3). | Brullo & Pavone (1981) and Pignatti (1982a) |
| *Limonium papillatum* (Webb & Berthel.) Kuntze | *L.* sect. *Ctenostachys* in *Limonium* subg. *Limonium* | see Figure 2 |  |
| ***Limonium paradoxum* Pugsley** | ‘Mediterranean lineage’* in *Limonium* subg*. Limonium* | The species is morphologically similar to *L. binervosum* (part of *L. binervosum* aggregate) which is placed in the 'Mediterranean lineage' (Figure 3). | Pignatti (1972) and Ingrouille & Stace (1986) |
| ***Limonium parosicum* Erben & Brullo** | ‘Mediterranean lineage’* in *Limonium* subg*. Limonium* | The species is morphologically similar to other Eastern Mediterranean taxa such as *L. palmare, L. microcycladicum, L. graecum* etc. *Limonium graecum* is placed in the 'Mediterranean lineage' (Figure 3). | Brullo & Erben (2016) |
| *Limonium parvibracteatum* Pignatti | ‘Mediterranean lineage’* in *Limonium* subg*. Limonium* | see Figure 3 |  |
| ***Limonium parvifolium* (Tineo) Pignatti** | ‘Mediterranean lineage’* in *Limonium* subg*. Limonium* | The species is assigned to *L. bocconei* complex and thus is morphlogically related to *L. bocconei, L. cosyrense* etc. These species are placed in the 'Mediterranean lineage' (Figure 3). | Guarino et al. (2017) |
| ***Limonium parvum*Ingr.** | ‘Mediterranean lineage’* in *Limonium* subg*. Limonium* | The species is morphologically similar to *L. binervosum* (part of *L. binervosum* aggregate) which is placed in the 'Mediterranean lineage' (Figure 3). | Pignatti (1972) and Ingrouille & Stace (1986) |
| ***Limonium patrimoniense* Arrigoni & Diana** | ‘Mediterranean lineage’* in *Limonium* subg*. Limonium* | The species is morphologically similar to *L. glomeratum, L. densiflorum* and *L. etruscum,* all of them part of *L. densiflorum* complex. *Limonium glomeratum* as a representative of this complex is phylogenetically related to other taxa of the 'Mediterranean lineage' (see details above in *L. glomeratum*). | Arrigoni & Diana (1993) |
| ***Limonium paui* Cámara & Sennen** | ‘Mediterranean lineage’* in *Limonium* subg*. Limonium* | The species is morphologically similar to other Mediterranean species, especially from Western Mediterranean, such as *L. viciosoi, L. catalaunicum* and *L. longebracteatum. Limonium longebracteatum* is placed in the 'Mediterranean lineage' (Figure 3). | Erben (1993) |
| *Limonium paulayanum* (Vierh.) Ghaz. & J.R.Edm. | *L.* sect. *Sarcophyllum* in *Limonium* subg. *Limonium* | see Figure 2 |  |
| ***Limonium pavonianum* Brullo** | ‘Mediterranean lineage’* in *Limonium* subg*. Limonium* | The species is morphologically similar to *L. glomeratum* and *L. densiflorum,* both part of *L. densiflorum* complex. *Limonium glomeratum* is morphologically and phylogenetically related to other taxa of the 'Mediterranean lineage' (see details above in *L. glomeratum*). | Greuter et al. (1989) and Guarino et al. (2017) |
| *Limonium pectinatum* Kuntze | *L.* sect. *Ctenostachys* in *Limonium* subg. *Limonium* | see Figure 2 |  |
| ***Limonium pelagosae* Bogdanović & Brullo** | ‘Mediterranean lineage’* in *Limonium* subg*. Limonium* | The species is morphologically similar to *L. cancellatum* and other species of *L. cancellatum* group. *Limonium cancellatum* is placed in the 'Mediterranean lineage' (Figure 3). | Bogdanović & Brullo (2015) |
| *Limonium peregrinum* (P.J.Bergius) R.A.Dyer | *L.* sect. *Circinaria* in *Limonium* subg. *Limonium* | see Figure 2 |  |
| *Limonium perezii* (Stapf) Hubbard ex L.H.Bailey | *L.* sect. *Pteroclados* subsect. *Nobiles* in *Limonium* subg. *Pteroclados s.l.* | see Figure 2 |  |
| *Limonium perfoliatum* (Kar. ex Boiss.) Kuntze | *L.* sect. *Nephrophyllum s.l.* in *Limonium* subg. *Limonium* | see Figure 2 |  |
| ***Limonium pericotii* (O.Bolòs & Vigo) Greuter & Burdet** | ‘Mediterranean lineage’* in *Limonium* subg*. Limonium* | The species is part of *L. minutum* aggregate. *Limonium minutum* is placed in the 'Mediterranean lineage' (Figure 3). | Greuter *et al.* (1989) |
| ***Limonium perplexum* L.Sáez & Rosselló** | ‘Mediterranean lineage’* in *Limonium* subg*. Limonium* | The species is part of *L. duriusculum-companyonis* complex. Representatives of this complex such as *L. thiniense* and *L. rigualii* are placed in the 'Mediterranean lineage' (Figure 3). | Sáez & Rosello (1999) |
| ***Limonium pescadense* Greuter & Burdet** | ‘Mediterranean lineage’* in *Limonium* subg*. Limonium* | The species is morphologically similar to *L. zembra,* part of L. albidum group. *Limonium hyblaeum* which is a representative of *L. albidum* group is placed in the 'Mediterranean lineage' (Figure 3). | Domina & El Mokni (2012) |
| ***Limonium peucetium* Pignatti** | *‘*Mediterranean lineage’* in *Limonium* subg*. Limonium* | The species is part of *L. divaricatum* aggregate, which is morphologically similar to *L. graecum. Limonium graecum* belongs to the 'Mediterranean lineage' (Figure 3). | Pignatti (1982a,b) |
| ***Limonium pharosianum* Bogdanović & Brullo** | ‘Mediterranean lineage’* in *Limonium* subg*. Limonium* | The species is morphologically similar to *L. cancellatum* and other species of *L. cancellatum* group. *Limonium cancellatum* is placed in the 'Mediterranean lineage' (Figure 3). | Bogdanović & Brullo (2015) |
| ***Limonium phitosianum* R.Artelari** | ‘Mediterranean lineage’* in *Limonium* subg*. Limonium* | The species is morphologically similar to *L. cephalonicum* which is placed in the 'Mediterranean lineage' (Figure 3). | Artelari (1984) and Brullo & Erben (2016) |
| *Limonium pigadiense* (Rech.f.) Rech.f. | ‘Mediterranean lineage’* in *Limonium* subg*. Limonium* | see Figure 3 |  |
| ***Limonium pinillense* Roselló & Peris** | ‘Mediterranean lineage’* in *Limonium* subg*. Limonium* | The species is morphologically similar to *L. tournefortii,* which is placed in the 'Mediterranean lineage' (Figure 3). | Roselló *et al.* (1997) |
|    \| ***Limonium piptopodum* Nevski** \| \| --- \| | *L.* sect. *Siphonocalyx* in *Limonium* subg. *Limonium* | The species was previously assigned to genus *Eremolimon* (*Eremolimon piptopodum* (Nevski) Lincz.) which is currently embeded in *Limonium.* All represenatives of *Eremolimon* currently comprise *L.* sect. *Siphonocalyx* (Malekmohammadi et al. 2017; see represenative in Figure 2). | Malekmohammadi et al. (2017) |
| ***Limonium planesiae* Pignatti** | ‘Mediterranean lineage’* in *Limonium* subg*. Limonium* | The species is part of *L. multiforme* aggregate (*L. cosyrense* group) and thus, it is morphologically similar to *L. multiforme,* which is placed in the 'Mediterranean lineage' (Figure 3). | Pignatti (1982a,b) |
| *Limonium platyphyllum* Lincz. | *L.* sect. *Limonium* in *Limonium* subg. *Limonium* | see Figure 2 |  |
| *Limonium plurisquamatum* Erben | ‘Mediterranean lineage’* in *Limonium* subg*. Limonium* | see Figure 3 |  |
| ***Limonium poimenum* Ilardi, Brullo, D.Cusimano & Giusso** | ‘Mediterranean lineage’* in *Limonium* subg*. Limonium* | The species is morphologically similar to *L. todaroanum*. The latter species is part of *L. densissimum* aggregate. A representative of this aggregate, *L. densissimum,* is placed in the 'Mediterranean lineage' (Figure 3). | Colombo (2002) and Ilardi *et al.* (2014) |
| ***Limonium pomelianum* (Rouy) Erben** | ‘Mediterranean lineage’* in *Limonium* subg*. Limonium* | The species is morphologically similar to *L. delicatulum* and it was formerly a variety of this species (*Limonium delicatulum* var. *leptostachys* (Pomel) Maire (1934)). *Limonium delicatulum* is placed in the 'Mediterranean lineage' (Figure 3). | Erben (2012) |
| ***Limonium pomoense* Bogdanović & Brullo** | ‘Mediterranean lineage’* in *Limonium* subg*. Limonium* | The species is morphologically similar to *L. cancellatum* and other species of *L. cancellatum* group. *Limonium cancellatum* is placed in the 'Mediterranean lineage' (Figure 3) | Bogdanović & Brullo (2015) |
| ***Limonium pontium* Pignatti** | ‘Mediterranean lineage’* in *Limonium* subg*. Limonium* | The species is part of *L. cosyrense* group and thus, morphologically similar to represenatives of this group, such as *L. cosyrense* and *L. multiforme.* The latter species is placed in the 'Mediterranean lineage' (Figure 3). | Pignatti (1982a) |
| ***Limonium ponzoi* (Fiori & Béguinot) Brullo** | ‘Mediterranean lineage’* in *Limonium* subg*. Limonium* | The species is morphologically similar to *L. bocconei* (*L. bocconei* aggregate) which is placed in the 'Mediterranean lineage' (Figure 3). | Pignatti (1982a) and Brullo (1998) |
| ***Limonium popovii* Kubanskaya** | *L.* sect. *Nephrophyllum s.l.* in *Limonium* subg. *Limonium* | The species is morphologically similar to *L. myrianthum* part of *L.* sect. *Nephrophyllum s.l.* (see also Figure 2 and Taxonomic proposals for *L. myrianthum* and related taxa). | Linczevski (1952) |
| ***Limonium portopetranum* Erben** | ‘Mediterranean lineage’* in *Limonium* subg*. Limonium* | The species is morphologically similar to other Mediterranean species, mostly from Baleares, such as *L. muradense,* *L. caprariense* and *L. minutum. Limonium minutum* is placed in the 'Mediterranean lineage' (Figure 3). | Erben (1993) |
| ***Limonium portovecchiense* Erben** | ‘Mediterranean lineage’* in *Limonium* subg*. Limonium* | The species is morphologically similar to *L. glomeratum,* which is phylogenetically related to other taxa of the 'Mediterranean lineage' (see details above in *L. glomeratum*). | Erben (2001) |
| ***Limonium postii* Domina, Erben & Raimondo** | ‘Mediterranean lineage’* in *Limonium* subg*. Limonium* | The species is morphologically similar to *L. sieberi* and *L. galilaeum. Limonium sieberi* is placed in the 'Mediterranean lineage' (Figure 3). | Domina *et al.* (2008) |
| ***Limonium potaninii* Ikonn.-Gal.** | *L.* sect. *Plathymenium* in *Limonium* subg. *Limonium* | The species has been assigned to *L.* sect. *Plathymenium* Ikonnikov-Galitzky 1936) and is morphologically similar to *L. aureum* of the same section (see Figure 2). | Ikonnikov-Galitzky (1936) |
| *Limonium preauxii* (Webb & Berthel.) Kuntze | *L.* sect. *Pteroclados* subsect. *Nobiles* in *Limonium* subg. *Pteroclados s.l.* | see Figure 2 |  |
| ***Limonium procerum* (C.E.Salmon) Ingr.** | ‘Mediterranean lineage’* in *Limonium* subg*. Limonium* | The species is morphologically similar to *L. binervosum* (part of *L. binervosum* aggregate) which is placed to the 'Mediterranean lineage' (Figure 3). | Pignatti (1972) and Ingrouille & Stace (1986) |
| *Limonium proliferum* (d'Urv.) Erben & Brullo | ‘Mediterranean lineage’* in *Limonium* subg*. Limonium* | see Figure 3 |  |
| ***Limonium protohermaeum* Arrigoni & Diana** | ‘Mediterranean lineage’* in *Limonium* subg*. Limonium* | The species is part of *L. multiforme* complex. Representatives of this group (e.g. *L. multiforme, L. circaei* and L*. remotispiculum*) are placed in the 'Mediterranean lineage' (Figure 3). | Guarino *et al.* (2017) |
| *Limonium pruinosum* (L.) Chaz. | *L.* sect. *Pruinosum* in *Limonium* subg. *Limonium* | see Figure 3 |  |
| ***Limonium pseudarticulatum* Erben** | ‘Mediterranean lineage’* in *Limonium* subg*. Limonium* | The species is morphologically close to *L. articulatum* which is placed in the 'Mediterranean lineage' (Figure 3). | Erben (1989) |
| ***Limonium pseudebusitanum* Erben** | ‘Mediterranean lineage’* in *Limonium* subg*. Limonium* | see Figure 3 |  |
| ***Limonium pseudodictyocladum* (Pignatti) L.Llorens** | ‘Mediterranean lineage’* in *Limonium* subg*. Limonium* | The species shows morphological similarities to *L. virgatum* which is placed in the 'Mediterranean lineage' (Figure 3). *Limonium pseudodictyocladum* was previously a subspecies of *L. virgatum.* | Pignatti (1972) |
| ***Limonium pseudolaetum* Arrigoni & Diana** | ‘Mediterranean lineage’* in *Limonium* subg*. Limonium* | The species is part of the *L. densiflorum* complex. A represenative of this complex, *Limonium glomeratum* is morphologically and phylogenetically related to other taxa of the 'Mediterranean lineage' (see details above in *L. glomeratum*). | Guarino *et al*. (2017) |
| ***Limonium pseudominutum* Erben** | ‘Mediterranean lineage’* in *Limonium* subg*. Limonium* | The species is morphologically similar to *L. minutum* (*L. minutum* group) which is placed in the 'Mediterranean lineage' in the phylogeny (Figure 3). | Erben (1988) |
| *Limonium puberulum* (Webb) Kuntze | *L.* sect. *Pteroclados* subsect. *Nobiles* in *Limonium* subg. *Pteroclados s.l.* | see Figure 2 |  |
| ***Limonium pujosii* Sauvage & Vindt** | ‘Mediterranean lineage’* in *Limonium* subg*. Limonium* | The species has been assigned to *L.* sect. *Limonium* subsect. *Dissitiflora* sensu Boissier (Sauvauge & Vindt 1953). Representatives of this subsection are all placed in the 'Mediterranean lineage' (see Figure 3). | Sauvage & Vindt (1953) |
| ***Limonium pulviniforme* Arrigoni & Diana** | ‘Mediterranean lineage’* in *Limonium* subg*. Limonium* | The species is morphologiacally related to *L. contortirameum* and is part of *L. articulatum* complex. *Limonium contortirameum* and *L. articulatum* are placed in the 'Mediterranean lineage' (Figure 3). | Guarino *et al*. (2017) |
| ***Limonium punicum* Brullo & Erben** | ‘Mediterranean lineage’* in *Limonium* subg*. Limonium* | The species is a triploid, morphologically similar to other Mediterranean taxa such as *L. cercinense, L. zeugitanum* and *L. delicatulum. Limonium delicatulum* is placed in the 'Mediterranean lineage' (Figure 3). | Brullo & Erben (1989) |
| *Limonium purpuratum* Hubbard ex L.H.Bailey | *L.* sect. *Circinaria* in *Limonium* subg. *Limonium* | see Figure 2 |  |
| ***Limonium pusillum* Erben & Brullo** | ‘Mediterranean lineage’* in *Limonium* subg*. Limonium* | The species is morphologically similar to other Eastern Mediterranean taxa such as *L. contractum, L. palmare, L. graecum* etc. *Limonium graecum* is placed in the 'Mediterranean lineage' (Figure 3). | Brullo & Erben (2016) |
| *Limonium pycnanthum* (K. Koch) Kuntze | *L.* sect. *Sphaerostachys* in *Limonium* subg. *Limonium* | see Figure 2 |  |
| *Limonium pylium* R. Artelari | ‘Mediterranean lineage’* in *Limonium* subg*. Limonium* | see Figure 3 |  |
| ***Limonium pyramidatum* Brullo & Erben** | ‘Mediterranean lineage’* in *Limonium* subg*. Limonium* | The species is morphologically and phylogenetically related to other Mediterranean taxa of the 'Mediterranean lineage' (see Malekmohammadi *et al.* 2017). | Brullo & Erben (1989) and Malekmohammadi *et al.* (2017) |
| ***Limonium quesadense* Erben** | ‘Mediterranean lineage’* in *Limonium* subg*. Limonium* | The species is morphologically similar to *L. delicatulum* and *L. carpetanicum* (part of *L.. delicatulum* complex). These species are placed in the 'Mediterranean lineage' (Figure 3). | Erben (1980) |
| ***Limonium quinnii* M.B.Crespo & Pena-Martín** | ‘Mediterranean lineage’* in *Limonium* subg*. Limonium* | The species is morphologically similar to other Eastern Mediterranean taxa such as *L. ikaricum* and *L. xiliense. Limonium xiliense* is placed in the 'Mediterranean lineage' (Figure 3) | Brullo & Erben (2016) |
| ***Limonium racemosum* (Lojac.) Diana** | ‘Mediterranean lineage’* in *Limonium* subg*. Limonium* | The species is part of *L. calcarae* complex which includes *L. calcarae, L. racemosum, L. multifurcatum, L. dichotomum* and *L. toletanum*. *Limonium* dichotomum and L*. toletanum* are placed in the 'Mediterranean lineage' (Figure 3). | Guarino *et al.* (2017) |
| ***Limonium raddianum* (Boiss.) Pignatti ex Brullo** | ‘Mediterranean lineage’* in *Limonium* subg*. Limonium* | The species is morphologically similar to the Mediterranean taxa *L. sibthorpianum, L. mucronulatum, L. teuchirae,* *L. vaccari* etc. and together with *L. raddianum* are part of *L. sibthorpianum* aggregate. *Limonium mucronulatum* was recently sampled in a phylogenetic study and is confidently placed within the 'Mediterranean lineage' (see "*L. graecum* clade" in Malekmohammadi *et al.* 2017) | Kouzali *et al*. (2012) and Malekmohammadi *et al.* (2017) |
| ***Limonium ramosissimum* (Poir.) Maire** | ‘Mediterranean lineage’* in *Limonium* subg*. Limonium* | The species is part of *L. densiflorum* complex (*L. ramossisimum* aggr. sensu Greuter *et al.* 1989). A represenative of this complex, *Limonium glomeratum* is morphologically and phylogenetically related to other taxa of the 'Mediterranean lineage' (see details above in *L. glomeratum*). | Greuter *et al.* (1989) and Guarino et al. (2017) |
| *Limonium recticaule* Erben & Brullo | ‘Mediterranean lineage’* in *Limonium* subg*. Limonium* | see Figure 3 |  |
| *Limonium recurvum* C.E.Salmon | ‘Mediterranean lineage’* in *Limonium* subg*. Limonium* | see Figure 3 |  |
| *Limonium redivivum* (Svent.) G.Kunkel & Sunding | *L.* sect. *Pteroclados* subsect. *Nobiles* in *Limonium* subg. *Pteroclados s.l.* | see Figure 2 |  |
| *Limonium relicticum* R.Mesa & A.Santos | *L.* sect. *Pteroclados* subsect. *Nobiles* in *Limonium* subg. *Pteroclados s.l.* | see Figure 2 |  |
| *Limonium remotispiculum* (Lacaita) Pignatti | ‘Mediterranean lineage’* in *Limonium* subg*. Limonium* | see Figure 3 |  |
| *Limonium reniforme* (Girard) Lincz. | *L.* sect. *Nephrophyllum s.l.* in *Limonium* subg. *Limonium* | see Figure 2 |  |
| ***Limonium retirameum* Greuter & Burdet** | ‘Mediterranean lineage’* in *Limonium* subg*. Limonium* | The species is morphologically similar to other Mediterranean taxa especially the diploids *L. oristanum, L. sulcitanum* and is part of *L. virgatum* aggregate. *Limonium virgatum* is placed in the 'Mediterranean lineage' (Figure 3). | Greuter *et al.* (1989) |
| ***Limonium retusum* L.Llorens** | ‘Mediterranean lineage’* in *Limonium* subg*. Limonium* | The species is part of *L. delicatulum* group. Representatives of this group such as *L. latebracteatum, L. biflorum, L. scopulorum* etc. are all placed in the 'Mediterranean lineage' (Figure 3). | Crespo (2009) and Moreno *et al.* (2018) |
| ***Limonium revolutum* Erben** | ‘Mediterranean lineage’* in *Limonium* subg*. Limonium* | The species is morphologically similar to *L. minutum* (*L. minutum* aggregate) which is placed in the 'Mediterranean lineage' (Figure 3). | Erben (1978) and Greuter *et al.* (1989) |
| ***Limonium rezniczenkoanum* Lincz.** | *L.* sect. *Plathymenium* in *Limonium* subg. *Limonium* | The species has been assigned to *L.* sect. *Plathymenium* (Linczevski 1952) and shows morphological similarities to representatives of this section (see representatives in Figure 2) | Linczevski (1952) |
| *Limonium rigualii* M.B.Crespo & Erben | ‘Mediterranean lineage’* in *Limonium* subg*. Limonium* | see Figure 3 |  |
| *Limonium roridum* (Sibth. & Sm.) Brullo & Guarino | ‘Mediterranean lineage’* in *Limonium* subg*. Limonium* | see Figure 3 |  |
| ***Limonium rosselloi* P.P.Ferrer, Roselló & E.Laguna** | ‘Mediterranean lineage’* in *Limonium* subg*. Limonium* | The species is morphologically closer to *L. erectum* which is placed in the 'Mediterranean lineage' in the phylogeny (Figure 3). | Ferrer-Gallego *et al.* (2013) |
| ***Limonium rubescens* Brullo & Erben** | ‘Mediterranean lineage’* in *Limonium* subg*. Limonium* | The species is morphologically similar to *L. delicatulum* which is placed in the 'Mediterranean lineage' (Figure 3). | Brullo & Erben (1989) |
| ***Limonium ruizii* (Font Quer) Fern.Casas** | ‘Mediterranean lineage’* in *Limonium* subg*. Limonium* | The species is morphologically similar to *L. aragonense* and it was previously a subspecies of it (*L. aragonense* subsp. *ruizii* (Font Quer) Fernández Casas & Mufioz Garmendia). *Limonium aragonense* is placed in the 'Mediterranean lineage' (Figure 3). | Erben (1978) and Erben (1993) |
| ***Limonium rungsii* Sauvage & Vindt** | ‘Mediterranean lineage’* in *Limonium* subg*. Limonium* | The species has been assigned to *L.* sect. *Limonium* subsect. *Steiroclada* (see Sauvage & Vindt 1952). Representatives assigned to this subsection are all placed in the 'Mediterranean lineage' (Figure 3). | Sauvage & Vindt (1952) |
| ***Limonium salmonis* (Sennen & Elias) Pignatti** | ‘Mediterranean lineage’* in *Limonium* subg*. Limonium* | The species is morphologically similar to *L. binervosum* (part of *L. binervosum* aggregate) which is placed in the 'Mediterranean lineage' (Figure 3). | Pignatti (1972) and Ingrouille & Stace (1986) |
| ***Limonium samium* Erben & Brullo** | ‘Mediterranean lineage’* in *Limonium* subg*. Limonium* | The species is morphologically similar to *L. vanandense* which is placed in the 'Mediterranean lineage' (Figure 3). | Brullo & Erben (2016) |
| *Limonium santapolense* Erben | ‘Mediterranean lineage’* in *Limonium* subg*. Limonium* | see Figure 3 |  |
| *Limonium saracinatum* R.Artelari | ‘Mediterranean lineage’* in *Limonium* subg*. Limonium* | see Figure 3 |  |
| *Limonium sarcophyllum* Ghaz. & J.R.Edm. | *L.* sect. *Sarcophyllum* in *Limonium* subg. *Limonium* | see Figure 2 |  |
| ***Limonium sardoum* (Pignatti) Erben** | ‘Mediterranean lineage’* in *Limonium* subg*. Limonium* | The species is part of *L. virgatum* aggregate. *Limonium virgatum* is placed in the 'Mediterranean lineage' (Figure 3). | Pignatti (1982a) |
| ***Limonium sareptanum* (Becker) Gams** | *L.* sect. *Limonium* in *Limonium* subg. *Limonium* | The species is morphologically similar to other taxa of the re-circumscribed *L.* sect. *Limonium* (e.g. *L. gmelini* and *L. bungei*) and is phylogenetically part of this section according to a recent study (Malekmohammadi *et al.* 2017). | Pignatti (1972) and Malekmohammadi *et al.* (2017) |
| ***Limonium sartorianum* Erben & Brullo** | ‘Mediterranean lineage’* in *Limonium* subg*. Limonium* | The species is morphologically similar to *L. sieberi* which is placed in the 'Mediterranean lineage' (Figure 3). | Brullo & Erben (2016) |
| ***Limonium savianum* Pignatti** | ‘Mediterranean lineage’* in *Limonium* subg*. Limonium* | The species is part of *L. multiforme* aggregate (*L. cosyrense* group) and thus, morphologically similar to *L. multiforme. Limonium multiforme* is part of the 'Mediterranean lineage' (Figure 3). | Pignatti (1982a) |
| ***Limonium saxicola* Erben** | ‘Mediterranean lineage’* in *Limonium* subg*. Limonium* | The species is morphologically similar to *L. alcudianum,* *L. gymnesicum* and *L. camposanum. Limonium gymnesicum* and *L. camposanum* are placed in the 'Mediterranean lineage' (Figure 3). | Erben (1989) |
| *Limonium scabrum* (Thunb.) Kuntze | ‘Mediterranean lineage’* in *Limonium* subg*. Limonium* | see Figure 3 |  |
| ***Limonium schinousae* Erben & Brullo** | *‘*Mediterranean lineage’* in *Limonium* subg*. Limonium* | The species is morphologically similar to *L. crateriforme* which is placed in the 'Mediterranean lineage' in the phylogeny (Figure 3). | Brullo & Erben (2016) |
| *Limonium scopulorum* M.B.Crespo & Lledóó | ‘Mediterranean lineage’* in *Limonium* subg*. Limonium* | see Figure 3 |  |
| ***Limonium scorpioides* Erben** | ‘Mediterranean lineage’* in *Limonium* subg*. Limonium* | The species is morphologically similar to other Mediterranean species, mostly from Baleares, such as *L. caprariense* and *L. minutum. Limonium minutum* is placed in the 'Mediterranean lineage' (Figure 3). | Erben (1989) and Erben (1993) |
| ***Limonium secundirameum* (Lojac.) Brullo** | ‘Mediterranean lineage’* in *Limonium* subg*. Limonium* | The species is part of *L. densissimum* morphological aggregate. *Limonium densissimum* is placed in the 'Mediterranean lineage' (Figure 3). | Pignatti (1982a) |
| ***Limonium selinuntinum* Brullo** | ‘Mediterranean lineage’* in *Limonium* subg*. Limonium* | The species is part of *L. densissimum* morphological aggregate. *Limonium densissimum* is placed in the 'Mediterranean lineage' (Figure 3). | Pignatti (1982a) |
| ***Limonium senkakuense* Yamazaki** | *L.* sect. *Plathymenium* in *Limonium* subg. *Limonium* | The species is morphologically similar to *L. sinense* and *L. tetragonum,* which are both placed in *L.* sect. *Plathymenium* (see Figure 2). | Yamazaki (1991) |
| ***Limonium serpentinicum* R.Pino, Silva Pando & J.J.Pino** | ‘Mediterranean lineage’* in *Limonium* subg*. Limonium* | The species is assigned to *L. binervosum* group. Representatives of this group such as *L. binervosum* and *L. recurvum* are placed in the 'Mediterranean lineage' (Figure 3). | Pérez *et al.* (2016) |
| ***Limonium serratum* Brullo & Erben** | ‘Mediterranean lineage’* in *Limonium* subg*. Limonium* | The species is morphologically similar to *L. pyramidatum* with which they also share the same chromosome number. *Limonium pyramidatum* is phylogenetically related to other Mediterranean taxa of the 'Mediterranean lineage' (see "*L. graecum* clade" in Malekmohammadi *et al.* 2017). | Brullo & Erben (1989) and Malekmohammadi *et al.* (2017) |
| ***Limonium sibthorpianum* (Guss.) Kuntze** | ‘Mediterranean lineage’* in *Limonium* subg*. Limonium* | The species is morphologically similar to the Mediterranean taxa *L. mucronulatum, L. raddianum, L. teuchirae,* *L. vaccari, L. optimae* etc. and are part of *L. sibthorpianum* aggregate. *Limonium mucronulatum* and *L. optimae* were recently sampled in a phylogenetic study and are confidently placed within the 'Mediterranean lineage' (see "*L. graecum* clade" in Malekmohammadi *et al.* 2017) | Kouzali *et al*. (2012) and Malekmohammadi *et al.* (2017) |
| *Limonium sieberi* (Boiss.) Kuntze | ‘Mediterranean lineage’* in *Limonium* subg*. Limonium* | see Figure 3 |  |
| ***Limonium silvestrei* Aparicio** | ‘Mediterranean lineage’* in *Limonium* subg*. Limonium* | The species shows morphological similarities (habit) with *L. virgatum* which is placed in the 'Mediterranean lineage' (Figure 3). | Aparicio (2005) |
| *Limonium sinense* (Girard) Kuntze | *L.* sect. *Plathymenium* in *Limonium* subg. *Limonium* | see Figure 2 |  |
| *Limonium sinuatum* (L.) Mill. | *L.*sect. *Pteroclados* subsect. *Odontolepideae* in *Limonium* subg. *Pteroclados s.l.* | see Figure 2 |  |
| ***Limonium sirinicum* Erben & Brullo** | ‘Mediterranean lineage’* in *Limonium* subg*. Limonium* | The species is morphologically similar to *L. doerfleri* and *L. albomarginatum* (*L. doerfleri* complex). The latter species is placed in the 'Mediterranean lineage' (Figure 3). | Brullo & Erben (2016) |
| *Limonium sitiacum* Rech.f. | ‘Mediterranean lineage’* in *Limonium* subg*. Limonium* | see Figure 3 |  |
| ***Limonium smithii* Akaydın** | *L.* sect. *Nephrophyllum s.l.* in *Limonium* subg. *Limonium* | The species is morphologically similar to other representatives of *L.* sect. *Nephrophyllum s.l.,* especially *L. caspium* and *L. bellidifolium* (see also Figure 2 and Taxonomic proposals). | Akaydın (2007) |
| ***Limonium soboliferum* Erben** | ‘Mediterranean lineage’* in *Limonium* subg*. Limonium* | The species is morphologically similar to other Mediterranean endemics and shares the same habit with *L. minus* which is placed in the 'Mediterranean lineage' (Figure 3). | Erben (1989) |
|    \| *Limonium sogdianum* Ikonn.-Gal. \| \| --- \| | *L.* sect. *Siphonocalyx* in *Limonium* subg. *Limonium* | see Figure 2 |  |
| *Limonium sokotranum* (Vierh.) Radcl.-Sm. | *L.* sect. *Sarcophyllum* in *Limonium* subg. *Limonium* | see Figure 2 |  |
| ***Limonium solanderi*Lincz.** | *L.* sect. *Plathymenium* in *Limonium* subg. *Limonium* | The species is morphologically similar to *L. australe* and are both part of *L.* sect. *Plathymenium* (see also placement of *L. australe* in Malekmohammadi *et al.* 2017)*.* | Linczevski (1986) |
| *Limonium somalorum* (Vierh.) Hutch. & E.A.Bruce | *L.* sect. *Sarcophyllum* in *Limonium* subg. *Limonium* | see Figure 2 |  |
| ***Limonium sommierianum* (Fiori) Arrigoni** | ‘Mediterranean lineage’* in *Limonium* subg*. Limonium* | The species is part of *L. multiforme* aggregate (*L. cosyrense* group) and thus, morphologically similar to *L. multiforme,* which is placed in the 'Mediterranean lineage' (Figure 3). | Pignatti (1982a) |
| *Limonium sougiae* Erben & Brullo | ‘Mediterranean lineage’* in *Limonium* subg*. Limonium* | see Figure 3 |  |
| ***Limonium spathulatum* (Desf.) Kuntze** | ‘Mediterranean lineage’* in *Limonium* subg*. Limonium* | The species has been assigned to *L.* sect. *Limonium* subsect. *Dissitiflora* (see Sauvage & Vindt 1952). Representatives assigned to this subsection are all placed in the 'Mediterranean lineage' in the phylogeny (Figure 3). | Sauvage & Vindt (1952) |
| *Limonium spectabile* (Svent.) G.Kunkel & Sunding | *L.* sect. *Pteroclados* subsect. *Nobiles* in *Limonium* subg. *Pteroclados s.l.* | see Figure 2 |  |
| *Limonium spreitzenhoferi* Erben & Brullo | ‘Mediterranean lineage’* in *Limonium* subg*. Limonium* | see Figure 3 |  |
| ***Limonium squarrosum* Erben** | ‘Mediterranean lineage’* in *Limonium* subg*. Limonium* | The species is a triploid, narrow endemic of Spain and shows morphological similarities to other Mediterranean endemic taxa. It is similar in habit to *L. carthaginense* which is placed in the 'Mediterranean lineage' (Figure 3). | Erben (1993) |
| ***Limonium stenophyllum* Erben** | ‘Mediterranean lineage’* in *Limonium* subg*. Limonium* | The species is a diploid endemic species of Spain and shares morphological similarities with *L. aragonense* which is placed in the 'Mediterranean lineage' (Figure 3) | Erben (1989) and Erben (1993) |
| *Limonium stenotatum* (Rech.f.) Erben & Brullo | ‘Mediterranean lineage’* in *Limonium* subg*. Limonium* | see Figure 3 |  |
| ***Limonium stephanii*Sennen** | ‘Mediterranean lineage’* in *Limonium* subg*. Limonium* | The species is morphologically similar to *L. aragonense. Limonium aragonense* is placed in the 'Mediterranean lineage' (Figure 3). | Erben (1993) |
| *Limonium stocksii* (Boiss.) Kuntze | *L.* sect. *Sarcophyllum* in *Limonium* subg. *Limonium* | see Figure 2 |  |
| ***Limonium strictissimum* (Salzmann) Arrigoni** | ‘Mediterranean lineage’* in *Limonium* subg*. Limonium* | The species is morphologiacally similar to *L. contortirameum* and part of *L. articulatum* complex. *Limonium contortirameum* and *L. articulatum* are placed in the 'Mediterranean lineage’ (Figure 3). | Guarino *et al*. (2017) |
| ***Limonium subanfractum* Trinajstić** | ‘Mediterranean lineage’* in *Limonium* subg*. Limonium* | The species is morphologically similar to *L. cancellatum* and other species of *L. cancellatum* group. *Limonium cancellatum* is placed in the 'Mediterranean lineage' (Figure 3). | Bogdanović & Brullo (2015) |
| *Limonium subglabrum* Erben | ‘Mediterranean lineage’* in *Limonium* subg*. Limonium* | see Figure 3 |  |
| ***Limonium subnudum* Bogdanović & Brullo** | ‘Mediterranean lineage’* in *Limonium* subg*. Limonium* | The species is morphologically similar to *L. cancellatum* and other species of *L. cancellatum* group. *Limonium cancellatum* is placed in the 'Mediterranean lineage' (Figure 3). | Bogdanović & Brullo (2015) |
| ***Limonium subrotundifolium* (Bég. & Vacc.) Brullo** | ‘Mediterranean lineage’* in *Limonium* subg*. Limonium* | The species is morphologically similar to *L. delicatulum* and *L. cyrenaicum.* The former species is placed in the 'Mediterranean lineage' (Figure 3). | Brullo (1978) and Jafri & El-Gadi (1984). |
| ***Limonium sucronicum* Erben** | ‘Mediterranean lineage’* in *Limonium* subg*. Limonium* | The species is a diploid endemic species of Spain that is part of *L. cofrentanum* complex (sensu Erben 1989) and shares some morphological similarities with *L. lobetanicum, L. cofrentanum, L. aragonense* etc. *Limonium aragonense* is placed in the 'Mediterranean lineage' (Figure 3). | Erben (1989) and Erben (1993) |
| *Limonium suffruticosum* (L.) Kuntze | *L.* sect. *Iranolimon* in *Limonium* subg. *Limonium* | see Figure 2 |  |
| ***Limonium sulcitanum* Arrigoni** | ‘Mediterranean lineage’* in *Limonium* subg*. Limonium* | The species is morphologically similar to other Mediterranean taxa especially the diploids *L. retirameum, L. merxmuelleri* etc. and is part of *L. virgatum* aggregate. *Limonium virgatum* is placed in the 'Mediterranean lineage' (Figure 3). | Greuter *et al.* (1989) |
| ***Limonium sundingii* Leyens, Lobin, N.Kilian & Erben** | *L.* sect. *Jovibarba* (or *Ctenostachys*) in *Limonium* subg. *Limonium* | The species is morphologically similar to *L. jovibarba.* However, similarly to *L. lobinii* which shares similar ecology and habit, the position of *L. sundingii* either within *L.* sect *Jovibarba* or *Ctenostachys* needs further investigation (see Figure 2 and Discussion). | Lobin *et al.* (1995) |
| *Limonium supinum* (Girard) Pignatti | ‘Mediterranean lineage’* in *Limonium* subg*. Limonium* | see Figure 3 |  |
| *Limonium sventenii* A. Santos & M.L.Fernández | *L.* sect. *Pteroclados* subsect. *Nobiles* in *Limonium* subg. *Pteroclados s.l.* | see Figure 2 |  |
| ***Limonium syracusanum* Brullo** | ‘Mediterranean lineage’* in *Limonium* subg*. Limonium* | The species is morphologically similar to *L. bocconei* (*L. bocconei* aggregate) which is placed in the 'Mediterranean lineage' (Figure 3). | Pignatti (1982a) and Brullo (1988) |
| *Limonium tabernense* Erben | ‘Mediterranean lineage’* in *Limonium* subg*. Limonium* | see Figure 3 |  |
| ***Limonium tabulare* Bogdanović & Brullo** | ‘Mediterranean lineage’* in *Limonium* subg*. Limonium* | The species is morphologically similar to *L. cancellatum* and other species of *L. cancellatum* group. *Limonium cancellatum* is placed in the 'Mediterranean lineage' (Figure 3). | Bogdanović & Brullo (2015) |
| ***Limonium tacapense* Brullo & Erben** | ‘Mediterranean lineage’* in *Limonium* subg*. Limonium* | The species is morphologically similar to *L. rubescens* and *L. delicatulum.* The latter species is placed in the 'Mediterranean lineage' (Figure 3). | Brullo & Erben (1989) |
| ***Limonium taenari* Erben & Brullo** | ‘Mediterranean lineage’* in *Limonium* subg*. Limonium* | The species is morphologically similar to *L. proliferum* which is placed in the 'Mediterranean lineage' (Figure 3). | Brullo & Erben (2016) |
| ***Limonium tamaricoides* Bokhari** | *L.* sect. *Nephrophyllum s.l.* in *Limonium* subg. *Limonium* | The species is morphologically similar to *L. bellidifolium* which is part of *L.* sect. *Nephrophyllum s.l.* (see also Figure 2). Indeed, *L. tamaricoides* is placed in the "*L. bellidifolium* clade" in a recent study (Malekmohhamadi *et al.* 2017). | Fazlioğlu (2011) and Malekmohammadi *et al*. (2017) |
| ***Limonium tamarindanum* Erben** | ‘Mediterranean lineage’* in *Limonium* subg*. Limonium* | The species is morphologically similar to *L. fontquerii* and *L. virgatum. Limonium virgatum* is placed in the 'Mediterranean lineage' (Figure 3). | Erben (1989) and Erben (1993) |
| ***Limonium tarcoense* Arrigoni & Diana** | ‘Mediterranean lineage’* in *Limonium* subg*. Limonium* | The species is part of *L. articulatum* complex. Representatives of this complex such as *L. articulatum, L. contortirameum* and *L. corsicum* are placed in the 'Mediterranean lineage' in the phylogeny (Figure 3). | Domina *et al.* (2014) |
| ***Limonium tauromenitanum* Brullo** | ‘Mediterranean lineage’* in *Limonium* subg*. Limonium* | The species is morphologically similar to *L. minutiflorum* (*L. minutiflorum* aggregate) which is placed in the 'Mediterranean lineage' (Figure 3). | Brullo & Pavone (1981) and Greuter *et al.* (1989) |
| *Limonium tenellum* (Turcz.) Kuntze | *L.* sect. *Plathymenium* in *Limonium* subg. *Limonium* | see Figure 2 |  |
| ***Limonium tenoreanum* (Guss.) Pignatti** | ‘Mediterranean lineage’* in *Limonium* subg*. Limonium* | The species is morphologically similar to *L. inarimense* and *L. minutiflorum.* The latter species is placed in the 'Mediterranean lineage' (Figure 3). | Pignatti (1982a) and Iamonico, Vallariello, & Guacchio (2017) |
| ***Limonium tenuicaule* Erben** | ‘Mediterranean lineage’* in *Limonium* subg*. Limonium* | The species is morphologically similar to *L. minutum* (*L. minutum* group) which is placed in the 'Mediterranean lineage' (Figure 3). | Erben (1989) |
| ***Limonium tenuiculum* (Tineo ex Guss.) Pignatti** | ‘Mediterranean lineage’* in *Limonium* subg*. Limonium* | The species is morphologically similar to *L. bocconei* (*L. bocconei* aggregate) which is placed in the 'Mediterranean lineage' (Figure 3). | Brullo (1988) |
| ***Limonium tenuifolium*  (Bertol. ex Moris) Erben** | ‘Mediterranean lineage’* in *Limonium* subg*. Limonium* | The species is part of *L. acutifolium* complex, which includes representatives such as *L. obtusifolium* and *L. bonifaciense*. These species are placed in the 'Mediterranean lineage' (Figure 3). | Pignatti (1982a) and Guarino *et al.* (2017) |
| ***Limonium teretifolium* L. Bolus** | *L.* sect. *Circinaria* in *Limonium* subg. *Limonium* | The species was previously assigned to genus *Afrolimon* (*Afrolimon teretifolium* (L.Bolus) Lincz.) which is currently embeded in *Limonium.* All represenatives of *Afrolimon* currently comprise *L.* sect. *Circinaria* (Malekmohammadi *et al.* 2017; see represenatives in Figure 2). | Malekmohammadi *et al.* (2017) |
| ***Limonium tetragonum* (Thunb.) Bullock** | *L.* sect. *Plathymenium* in *Limonium* subg. *Limonium* | see Figure 2 |  |
| ***Limonium teuchirae* Brullo** | ‘Mediterranean lineage’* in *Limonium* subg*. Limonium* | The species is morphologically similar to the Mediterranean taxa *L. sibthorpianum, L. mucronulatum, L. raddianum,* *L. vaccari* etc. and together with *L. teuchirae* are part of *L. sibthorpianum* aggregate. *Limonium mucronulatum* was recently sampled in a phylogenetic study and is confidently placed within the 'Mediterranean lineage' (see "*L. graecum* clade" in Malekmohammadi *et al.* 2017). | Kouzali *et al*. (2012) and Malekmohammadi *et al.* (2017) |
| ***Limonium thaenicum* Brullo & Erben** | ‘Mediterranean lineage’* in *Limonium* subg*. Limonium* | The species is morphologically similar to *L. rubescens* and *L. delicatulum.* The latter species is placed in the 'Mediterranean lineage' (Figure 3) | Brullo & Erben (1989) |
| ***Limonium tharrosianum* Arrigoni & Diana** | ‘Mediterranean lineage’* in *Limonium* subg*. Limonium* | The species is part of '*L. acutifolium* group'. Represenatives of this group, such as *L. obtusifolium* and *L. bonifaciense* that are morphologically similar to *L. tharrosianum,* are placed in the 'Mediterranean lineage' (Figure 3). | see "acutifolium" group in Arrigoni & Diana (1999) |
| *Limonium thiniense* Erben | ‘Mediterranean lineage’* in *Limonium* subg*. Limonium* | see Figure 3 |  |
| ***Limonium thirae* Erben & Brullo** | ‘Mediterranean lineage’* in *Limonium* subg*. Limonium* | The species is morphologically similar to *L. stenotatum* which is placed in the 'Mediterranean lineage' (Figure 3). | Brullo & Erben (2016) |
| ***Limonium tianschanicum* Lincz.** | *L.* sect. *Nephrophyllum s.l.* in *Limonium* subg. *Limonium* | The species is morphologically closely related to *L. myrianthum* and other representatives of *L* sect. *Nephrophyllum s.l.* (see also Taxonomic proposals). | Linczevski (1971) |
| ***Limonium tibulatium* Pignatti** | ‘Mediterranean lineage’* in *Limonium* subg*. Limonium* | The species is morphologically similar to *L. articulatum* (*L. articulatum* group) which is placed in the 'Mediterranean lineage' (Figure 3). | Pignatti (1982a) |
| ***Limonium tineoi* (Lojac.) Giardina & Raimondo** | ‘Mediterranean lineage’* in *Limonium* subg*. Limonium* | The species is morphologically similar to *L. pescadense* (syn. *Statice psiloclada*)*,* part of *L. albidum* group. *Limonium hyblaeum* which is a representative of *L. albidum* group is placed in the 'Mediterranean lineage' (Figure 3). | Lojacono (1907) |
| ***Limonium tobarrense* J.Moreno, Terrones, M.Á.Alonso, Juan & M.B.Crespo** | ‘Mediterranean lineage’* in *Limonium* subg*. Limonium* | The species is placed in *L. girardianum* complex and is morphologically similar to *L. girardianum* and *L. dufourii,* which are both placed in the 'Mediterranean lineage' (Figure 3). | Moreno *et al.* (2016) |
| ***Limonium todaroanum* Raimondo & Pignatti** | ‘Mediterranean lineage’* in *Limonium* subg*. Limonium* | The species is part of *L. densissimum* aggregate. *Limonium densissimum* is placed in the 'Mediterranean lineage' (Figure 3). | Colombo (2002) |
| *Limonium toletanum* Erben | ‘Mediterranean lineage’* in *Limonium* subg*. Limonium* | see Figure 3 |  |
| *Limonium tomentellum* (Boiss.) Kuntze | *L.* sect. *Limonium* in *Limonium* subg. *Limonium* | see Figure 2 |  |
| *Limonium tournefortii* (Boiss.) Erben | ‘Mediterranean lineage’* in *Limonium* subg*. Limonium* | see Figure 3 |  |
| ***Limonium trachycladum* Maire & Wilczek** | *L.* sect. *Ctenostachys* in *Limonium* subg. *Limonium* | The species is assigned to *L.* sect. *Ctenostachys* and was previously considered as variety of *L. fallax* (*Limonium fallax* var. *trachycladum* (Maire & Wilczek) Maire). | Maire (1936) |
| ***Limonium transwallianum* (Pugsley) Pugsley** | ‘Mediterranean lineage’* in *Limonium* subg*. Limonium* | The species is morphologically similar to *L. binervosum* (part of *L. binervosum* aggregate) which is placed in the 'Mediterranean lineage' (Figure 3). | Pignatti (1972) and Ingrouille & Stace (1986) |
| ***Limonium tremolsii* (Rouy) Erben** | ‘Mediterranean lineage’* in *Limonium* subg*. Limonium* | The species is morphologically similar to *L. virgatum* and *L. laxiusculum.* Both species are placed in the 'Mediterranean lineage' (Figure 3). | Erben (1978) and Erben (1993) |
| ***Limonium trinajsticii* Bogdanović & Brullo** | ‘Mediterranean lineage’* in *Limonium* subg*. Limonium* | The species is morphologically similar to *L. cancellatum* and other species of *L. cancellatum* group. *Limonium cancellatum* is placed in the 'Mediterranean lineage' (Figure 3). | Bogdanović & Brullo (2015) |
| ***Limonium tritonianum* Brullo & Erben** | ‘Mediterranean lineage’* in *Limonium* subg*. Limonium* | The species is morphologically similar to *L. rubescens* and *L. delicatulum.* The latter species is placed in the 'Mediterranean lineage' (Figure 3). | Brullo & Erben (1989) |
| *Limonium tuberculatum* (Boiss.) Kuntze | *L.* sect. *Pruinosum* in *Limonium* subg. *Limonium* | see Figure 3 |  |
| *Limonium tubiflorum* (Del.) Kuntze | *L.* sect. *Siphonantha* in *Limonium* subg. *Limonium* | see Figure 3 |  |
| *Limonium tunetanum* (Barratte) Maire | ‘Mediterranean lineage’* in *Limonium* subg*. Limonium* | see Figure 3 |  |
| ***Limonium tyrrhenicum* Arrigoni & Diana** | ‘Mediterranean lineage’* in *Limonium* subg*. Limonium* | The species is part of *L. virgatum* complex and is similar to representatives of this complex such as *L. virgatum, L. dubium, L. algusae* etc. *Limonium virgatum* is placed in the 'Mediterranean lineage' in the phylogeny (Figure 3). | Guarino *et al.* (2017) |
| ***Limonium ugijarense* Erben** | ‘Mediterranean lineage’* in *Limonium* subg*. Limonium* | The species is morphologically similar to *L. supinum* which is placed in the 'Mediterranean lineage' in the phylogeny (Figure 3). | Erben (1989) and Erben (1993) |
| ***Limonium ursanum* Erben** | ‘Mediterranean lineage’* in *Limonium* subg*. Limonium* | The species is part of *L. articulatum* aggregate*. Limonium articulatum* and other related taxa (e.g. *L. contortirameum*) are all placed in the 'Medieterranean lineage' (Figure 3). | Guarino *et al*. (2017) |
| ***Limonium usticanum* Giardina & Raimondo** | ‘Mediterranean lineage’* in *Limonium* subg*. Limonium* | The species is morphologically similar to *L. bocconei* which is placed in the 'Mediterranean lineage' (Figure 3). | Giardina *et al.* (2007) |
| ***Limonium vaccarii* Brullo** | ‘Mediterranean lineage’* in *Limonium* subg*. Limonium* | The species is morphologically similar to the Mediterranean taxa *L. sibthorpianum, L. mucronulatum, L. raddianum,* and *L. teuchirae,* and together with *L. vaccarii* are part of *L. sibthorpianum* aggregate. *Limonium mucronulatum* was recently sampled in a phylogenetic study and is confidently placed within the 'Mediterranean lineage' (see "*L. graecum* clade" in Malekmohammadi *et al.* 2017). | Kouzali *et al*. (2012) and Malekmohammadi *et al.* (2017) |
| ***Limonium validum* Erben** | ‘Mediterranean lineage’* in *Limonium* subg*. Limonium* | The species is part of *L. duriusculum-companyonis* complex. Representatives of this complex such as *L. thiniense* and *L. rigualii* are placed in the 'Mediterranean lineage' (Figure 3). | Erben (1989) and Sáez & Rosello (1999) |
| *Limonium vanandense* Erben & Brullo | ‘Mediterranean lineage’* in *Limonium* subg*. Limonium* | see Figure 3 |  |
| ***Limonium vanense* Kit Tan & Sorger** | *L.* sect. *Nephrophyllum s.l.* in *Limonium* subg. *Limonium* | The species is morphologically similar to other representatives of *L.* sect. *Nephrophyllum s.l.,* especially *L. bellidifolium* complex (see also Figure 2). | Fazlioğlu (2011) and Erdal (2015) |
| ***Limonium velutinum* Bogdanović & Brullo** | ‘Mediterranean lineage’* in *Limonium* subg*. Limonium* | The species is morphologically similar to *L. cancellatum* and other species of *L. cancellatum* group. *Limonium cancellatum* is part of the 'Mediterranean lineage' (Figure 3). | Bogdanović & Brullo (2015) |
| ***Limonium vestitum* (C.E. Salmon) C.E. Salmon** | ‘Mediterranean lineage’* in *Limonium* subg*. Limonium* | The species is morphologically similar to *L. cancellatum* and part of *L. cancellatum* group with its represenatives placed in the 'Mediterranean lineage' (see Figure 3). | Pignatti (1972) and Bogdanović & Brullo (2015) |
| ***Limonium viciosoi* (Pau) Erben** | ‘Mediterranean lineage’* in *Limonium* subg*. Limonium* | The species is morphologically similar to *L. catalaunicum* (*L. viciosoi* was formerly a subspecies it) and *L. longebracteatum* which is placed in the 'Mediterranean lineage' (Figure 3). | Erben (1993) |
| *Limonium vigaroense* Marrero Rodr. & R.S.Almeida | *L.* sect. *Pteroclados* subsect. *Nobiles* in *Limonium* subg. *Pteroclados s.l.* | see Figure 2 |  |
| ***Limonium vigoi* L.Sáez, Curcó & Rosselló** | ‘Mediterranean lineage’* in *Limonium* subg*. Limonium* | The species is morphologically similar to *L. girardianum* and *L. grosii.* The former species is placed in the 'Mediterranean lineage' (Figure 3). | Sáez *et al.* (1998b) |
| ***Limonium viniolae* Arrigoni & Diana** | ‘Mediterranean lineage’* in *Limonium* subg*. Limonium* | The species is part of *L. acutifolium* complex, which includes representatives such as *L. obtusifolium* and *L. bonifaciense*. These species are placed in the 'Mediterranean lineage' (Figure 3). | Guarino *et al.* (2017) |
| *Limonium virgatum* (Willd.) Fourr. | ‘Mediterranean lineage’* in *Limonium* subg*. Limonium* | see Figure 3 |  |
| ***Limonium vravronense* Erben & Brullo** | ‘Mediterranean lineage’* in *Limonium* subg*. Limonium* | The species is morphologically similar to other Mediterranean species, especially endemics of the Aegean region, such as *L. aucheri* and *L. ocymifolium* which are placed in the 'Mediterranean lineage' (Figure 3). | Brullo & Erben (2016) |
| *Limonium vulgare* Mill. | *L.* sect. *Limonium* in *Limonium* subg. *Limonium* | see Figure 2 |  |
| ***Limonium wiedmannii* Erben** | ‘Mediterranean lineage’* in *Limonium* subg*. Limonium* | The species is part of *L. delicatulum* group. Representatives of this group such as *L. latebracteatum, L. biflorum, L. scopulorum* etc. are all placed in the 'Mediterranean lineage' (Figure 3). | Crespo (2009) and Moreno *et al.* (2018) |
| *Limonium wrightii* (Hance) Kuntze | *L.* sect. *Plathymenium* in *Limonium* subg. *Limonium* | see Figure 2 |  |
| *Limonium xerocamposicum* Erben & Brullo | ‘Mediterranean lineage’* in *Limonium* subg*. Limonium* | see Figure 3 |  |
| ***Limonium xerophilum* Brullo & Erben** | ‘Mediterranean lineage’* in *Limonium* subg*. Limonium* | The species is morphologically similar to other Mediterranean taxa. Specifically, it is very similar in habit to *L. virgatum*, which is placed in the 'Mediterranean lineage' in the phylogeny (Figure 3). | Brullo & Erben (1989) |
| *Limonium xiliense* Erben & Brullo | ‘Mediterranean lineage’* in *Limonium* subg*. Limonium* | see Figure 3 |  |
| ***Limonium xipholepis* (Baker) Hutch. & E.A.Bruce** |  | The species is endemic to Somalia, where three other *Limonium* taxa occur there (i.e. *L. axillare, L. cylindrifolium* and *L. maurocordatae*). All these three species are assigned to the re-circumscribed *L.* sect. *Sarcophyllum* (see Figure 2 and Taxonomic Proposals). *Limonium xipholepis* shares a lot of morphological similarities with the aforementioned taxa, namely flat, rigid leaves with 3 nerves present also in *L. axillare,* and spikelets with funnel-form calyces that are similar to the ones of *L. maurocordatae*. However, unlike the other representatives the leaves are spirally arranged in dense basal rossetes, whereas in the other representatives of *L.* sect. *Sarcophyllum* leaves are mostly cauline. *Limonium xipholepis* is possibly assigned to the *L.*sect *Sarcophyllum*, but it needs further examination before we can confidently assign it to a clade/section. | |
| ***Limonium zacynthium* R.Artelari** | ‘Mediterranean lineage’* in *Limonium* subg*. Limonium* | The species is morphologically similar to *L. coronense* which is placed in the 'Mediterranean lineage' (Figure 3). | Brullo & Erben (2016) |
| ***Limonium zankii* Bogdanović & Brullo** | ‘Mediterranean lineage’* in *Limonium* subg*. Limonium* | The species is morphologically similar to *L. cancellatum* and other species of *L. cancellatum* group. *Limonium cancellatum* is placed in the 'Mediterranean lineage' (Figure 3). | Bogdanović & Brullo (2015) |
| ***Limonium zanonii* (Pamp.) Domina** | *L.* sect. *Siphonantha* in *Limonium* subg. *Limonium* | The species is morphologically similar to *L. tubiflorum* ( placed in *L.* sect. *Siphonantha*) and it was formerly ranked as a subspecies of *L. tubiflorum* (i.e. *Limonium tubiflorum* subsp. *zanonii* (Pamp.) Brullo). | Brullo (1978) and Greuter & Raab-Straube (2011) |
| ***Limonium zembrae* Pignatti** | ‘Mediterranean lineage’* in *Limonium* subg*. Limonium* | The species is morphologically similar to *L. albidum* and is part of *L. albidum* group. *Limonium hyblaeum* which is a representative of *L. albidum* group is placed in the 'Mediterranean lineage' in the phylogeny (Figure 3). | Pignatti (1982a,b) |
| ***Limonium zeraphae* Brullo** | ‘Mediterranean lineage’* in *Limonium* subg*. Limonium* | The species is part of *L. divaricatum* aggregate, which is morphologically similar to *L. graecum. Limonium graecum* is placed in the 'Mediterranean lineage' (Figure 3). | Pignatti (1982a) |
| ***Limonium zeugitanum* Brullo & Erben** | ‘Mediterranean lineage’* in *Limonium* subg*. Limonium* | The species is morphologically similar to other Mediterranean taxa such as *L. cercinense, L. punicum* and *L. delicatulum. Limonium delicatulum* is placed in the 'Mediterranean lineage' (Figure 3). | Brullo & Erben (1989) |

**Supplementary Figure legends**

**Figure S1.** Phylogeny of Plumbaginaceae with Polygonaceae outgroups inferred from Bayesian analysis of the ITS dataset. In the 50% majority-rule tree posterior probabilities above 0.7 and bootstrap support values above 50% estimated from MrBayes and RAxML analyses are reported above and below the branches, respectively. The collapsed node corresponds to the ‘Mediterranean lineage’ presented in detail in Figures 3.

**Figure S2.** Phylogeny of Plumbaginaceae with Polygonaceae outgroups inferred from Bayesian analysis of the cpDNA dataset. In the 50% majority-rule tree posterior probabilities above 0.7 and bootstrap support values above 50% estimated from MrBayes and RAxML analyses are reported above and below the branches, respectively. The collapsed node corresponds to the ‘Mediterranean lineage’ presented in detail in Figures 3.

**Supplementary References**

Akaydın, G. (2007). A new species of Limonium from the central Anatolian salt steppe. *Turkey. World Appl Sci J, 2*(4), 406-411.

Aparicio, A. (2005). Limonium silvestrei (Plumbaginaceae), a new agamospecies from southern Spain. In *Annales Botanici Fennici* (pp. 371-377). Finnish Zoological and Botanical Publishing Board.

Arrigoni, P. V., & Diana, S. (1999). Karyology, chorology and bioecology of the genus Limonium (Plumbaginaceae) in Sardinia. *Plant Biosystem, 133*(1), 63-71.

Arrigoni, P. V., & Rizzotto, M. (1985). Limonium etruscum (Plumbaginaceae), specie nuova, del Parco Naturale della Maremma in Toscana. *Webbia, 39*(1), 129-133.

Arrigoni, P.V. & Diana, S. (1993). Contribution a la connaissance du genre Limonium en Corse*. Candollea 48*(2), 631-677.

Artelari, P. (1984). Viosystematiki meleti tou genous Limonium (Plumbaginaceae) stin periohi tou Ioniou pelagous/Βιοσυστηματική μελέτη του γένους Limonium (Plumbaginaceae) στην περιοχή του Ιονίου πελάγους. – Doctoral dissertation, Department of Biology, University of Patras, Greece.

Artelari, R., & Kamari, G. (1986). A karyological study of ten Limonium species (Plumbaginaceae) endemic in the Ionian area, Greece. *Willdenowia*, 497-513.

Artelari, R., & Kamari, G. (2000). Limonium messeniacum (Plumbaginaceae), a new species from S. Peloponnisos (Greece). *Botanika Chronika, 13*, 45-49.

Bartolo, G., Brullo, S., & Giusso del Galdo, G. (2003). Limonium formosum (Plumbaginaceae), a new species from the island of Jerba (Tunisia). *Bocconea 16*(2), 537-541.

Bogdanović, S., & Brullo, S. (2015). Taxonomic revision of the Limonium cancellatum group (Plumbaginaceae) in Croatia. *Phytotaxa, 215*(1), 1-87.

Boissier, E. (1848) *Plumbaginaceae*. In A. P. de Candolle [ed.], Prodromus systematis naturalis regni vegetabilis, 12: 617–696. Treuttel et Wurz, Paris, France.

Brullo, C., Brullo, S., Cambria, S., Del Galdo, G. G., & Ilardi, V. (2016). Limonium cophanense (Plumbaginaceae), a new species from Sicily. *Phytotaxa, 255*(2), 153-159.

Brullo, S. & Erben, M. (1989). The genus Limonium (Plumbaginaceae) in Tunisia. *Mitteilungen der Bo-tanischen Staatssammlung München, 28*, 419-500.

Brullo, S. & Pavone, P. (1981). Chromosome numbers in the Sicilian species of" Limonium" Miller (" Plumbaginaceae"). In *Anales del Jardín Botánico de Madrid* (Vol. 37, No. 2, pp. 535-555). Real Jardín Botánico.

Brullo, S. (1978). Il genere «Limonium» Miller in Cirenaica. *Webbia, 33*(1), 137-158.

Brullo, S. (1988). Miscellaneous notes on the genus Limonium (Plumbaginaceae). *Willdenowia*, 11-18.

Brullo, S. (1992). Limonium brutium, a new species from S. Italy. *Flora Mediterranea, 2*, 109-112.

Brullo, S., & Erben, M. (2016). The genus Limonium (Plumbaginaceae) in Greece*. Phytotaxa, 240*(1), 1-212.

Brullo, S., & Giusso del Galdo, G. (2006). Limonium elfahsianum (Plumbaginaceae), a new species from Tunisia. *Bocconea 19,* 143-146.

Brullo, S., Marceno, C., Romano, S. (1996) Limonium melancholicum Brullo, Marceno et Romano (Plumbaginaceae), a new species from Sicily. *Candollea 51*(1), 99-102.

Cherkasova, G. I. (1970). Limonium cretaceum Tscherk. sp. n. from the chalk outcrops of western Kazakhstan. *Moskov Obshch Ispytatelei Prirody Biul Otd Biol, 4*, 100-103.

Colombo, P. (2002). Morpho-anatomical and taxonomical remarks on Limonium (Plumbaginaceae) in Sicily. *Flora Mediterranea 12*, 389-412.

Crespo, M. B. (2009). A new coastal species of Limonium (Plumbaginaceae) from southeastern Spain. *Folia Geobotanica, 44*(2), 177-190.

Diez Garretas, B. (1981). Limonium malacitanum Diez Garretas nueva especie. *Trab. Monogr. Dep. Bot. Malaga, 2,* 123-130.

Doğan, M. & Akaydın, G. (2017). Two new species of Lionium Mill. (plumbaginaceae) from southwest Anatolia, Turkey. *Ot Sistematik Botanic Dergisi 24*(2), 9-26.

Doğan, M., Duman, H., & Akaydın, G. (2008). Limonium gueneri (Plumbaginaceae), a new species from Turkey. In *Annales Botanici Fennici* (Vol. 45, No. 5, pp. 389-393). Finnish Zoological and Botanical Publishing Board.

Domina, G. & El Mokni, R. (2012). Suivi floristique de l’archipel de Zembra et Zembretta (Tunisie), Note naturaliste PIM. Aix-en-Provence.

Domina, G., & Mazzola, P. (2003). A phenetic approach to the genus Limonium (Plumbaginaceae) in Sicily. *Bocconea, 16*(2), 597-606.

Domina, G., & Raimondo, F. M. (2013). Limonium cedrorum sp. nov.(Plumbaginaceae) from Lebanon. *Nordic Journal of Botany, 31*(2), 194-197.

Domina, G., Danin, A., & Raimondo, F. M. (2006). A new species of Limonium (Plumbaginaceae) from Israel. *Flora Mediterranea 16,* 133-138.

Domina, G., Erben, M., & Raimondo, F. M. (2008). Two new species of Limonium (Plumbaginaceae) from Lebanon. *Flora Mediterranea, 18*, 333-339.

Domina, G., Scafidi, F., & Bartolucci, F. (2014). Osservazioni sulle strategie riproduttive dei taxa appartenenti al gruppo di Limonium articulatum (Plumbaginaceae). BI Gruppo per la Floristica, Sistematica ed Evoluzione.

Dyer, R. A. (1961) *Plumbaginaceae*. In Notes and New Records of African Plant, *Bothalia, 7,* 488-491.

Dyer, R. A. (1963) in Flora of Southern Africa (edited by R. A. Dyer, L. E. Codd and H. B. Rycroft), Vol. 26: 15-31, The Government Printer, Pretoria, Republic of South Africa.

Erben, M. (1978). Die gattung Limonium im südwestmediterranen raum. *Mitteilungen der Botanischen Staatssammlung München, 14*, 361-631.

Erben, M. (1980). Bemerkungen zur Taxonomie der Gattung Limonium. I. *Mitteilungen der Botanischen Staatssammlung Munchen, 16*, 547-563.

Erben, M. (1988). Bemerkungen zur Taxonomie der Gattung Limonium IV. *Mitteilungen der Botanischen Staatssammlung München*, *27*, 381-406.

Erben, M. (1989). Bemerkungen zur Taxonomie der Gattung Limonium V. *Mitteilungen der Botanischen Staatssammlung München, 28*, 313-417.

Erben, M. (1991). Bemerkungen zur Taxonomie der Gattung Limonium VI. *Mitteilungen der Botanischen Staatssammlung München, 30*, 459-478.

Erben, M. (1993). *Limonium*. In: Castroviejo S, Aedo C, Cirujano S et al. eds. *Flora Iberica 3*, 2–143. Madrid: Real Jardín Botánico-C.S.I.C.

Erben, M. (2001). Bemerkungen zur taxonomie der Gattung Limonium VII. *Sendtnera-Mitteilungen der Botanischen Staatssammlung und des Instituts fuer Systematische Botanik der Universitaet Muenchen, 7*, 53-84.

Erben, M. (2002). Limonium lambinonii und Limonium calanchicola: zwei neue Arten aus Korsika. *Sendtnera, 8,* 25-33.

Erben, M. (2006). Limonium greuteri (Plumbaginaceae), a new species from the island of Corsica (France). *Willdenowia, 36*(1), 145-148.

Erben, M. (2012). Contributions to the taxonomy of the genus Limonium IX. *Flora Mediterranea, 22*, 63-66.

Erben, M., & Arán, V. J. (2005). Limonium mateoi (Plumbaginaceae), a new species from Central Spain. In *Anales del Jardín Botánico de Madrid* (Vol. 62, No. 1). Consejo Superior de Investigaciones Científicas.

Erben, M., & Mucina, L. (2006). Limonium failachicum (Plumbaginaceae)—New and so far the only endemic plant from Kuwait. *Folia Geobotanica, 41*(2), 229-235.

Erdal, J. (2015). A numerical taxonomic study on the family Plumbaginaceae in Turkey (Doctoral dissertation, Middle East Technical University).

Fazlioğlu, F. (2011). A phenetics study for infrageneric grouping of Limonium Mill. Genus (Plumbaginaceae) In Turkey (Doctoral dissertation, Middle East Technical University).

Fennane, M., & Tattou, M. I. (2005). Flore vasculaire du Maroc: Inventaire et chorologie. Vol 1, 316. Pteridophyta, Gymnospermae, Angiospermae.

Ferrer-Gallego, P. P., Laguna, E., Roselló, R., Peris, J. B., & Gómez-Navarro, J. (2018). Limonium albarracinense (Plumbaginaceae), una Nueva Especie para la Flora Ibérica (España). *Novon: A Journal for Botanical Nomenclature, 26*(1), 28-36.

Ferrer-Gallego, P. P., Navarro, A., Pérrez, P., Roselló, R., Roselló, J., Rosato, M., & Laguna, E. (2015). A new polyploid species of Limonium (Plumbaginaceae) from the Western Mediterranean basin. *Phytotaxa, 234*(3), 263-270.

Ferrer-Gallego, P. P., Roselló, R., & Laguna, E. (2013). Limonium rosselloi (Plumbaginaceae), nueva especie para la Península Ibérica*. Collectanea Botanica, 32*, 33-41.

Ferrer-Gallego, P. P., Roselló, R., Rosato, M., Roselló, J. A., & Laguna, E. (2016). Limonium albuferae (Plumbaginaceae), a new polyploid species from the Eastern Iberian Peninsula. *Phytotaxa, 252*(2), 114-122.

Georgakopoulou, A., Manousou, S., Artelari, R., & Georgiou, O. (2006). Breeding systems and cytology in Greek populations of five Limonium species (Plumbaginaceae). *Willdenowia, 36*(2), 741-750.

Giardina, G., Raimondo, F. M., & Spadaro, V. (2007). A catalogue of plants growing in Sicily. Herbarium Mediterraneum Panormitanum.

Gil, L., & García, L. L. (1991). " Limonium barceloi" y" L. bolosii" Gil & Llorens, nuevas especies de la isla de Mallorca (Baleares). In *Anales del Jardín Botánico de Madrid* (Vol. 49, No. 1, pp. 51-56). Real Jardín Botánico.

Gómiz, F. (1995). Limonium alicunense (Plumbaginaceae), unanueva especie para la flora ibérica. *Anales del Jardín Botánico de Madrid 53*(2): 255-257.

Greuter, W., & Raab-Straube, E. V. (2011). Euro+ Med Notulae, 5. *Willdenowia, 41*(1), 129-138.

Greuter, W., Burdet, H. M. & Long, G. (ed.) (1989): Med-Checklist 4, 321-344 - Genève: Conservatoire et Jardin botaniques; Berlin: Botanischer Garten and Botanisches Museum Berlin-Dahlem.

Guarino, R., La Rosa, M. & Pignatti S. (2017) *Limonium*. In Flora d'Italia,Vol. 2, 18-48, Edagricole-New Business Media.

Holmgren, P., K., & Holmgren N., H. (1998, continuously updated). [*Index Herbariorum: A global directory of public herbaria and associated staff*](http://sciweb.nybg.org/science2/IndexHerbariorum.asp). New York: New York Botanical Garden’s Virtual Herbarium. <http://sweetgum.nybg.org/science/ih/>.

Iamonico, D., Vallariello, R., & Guacchio, E. D. (2017). Nomenclatural and distributional remarks on Limonium tenoreanum (Plumbaginaceae), a narrow endemic from southern Italy. *Nordic Journal of Botany, 35*(4), 445-448.

Ikonnikov-Galitzky, N. P. (1936) Limonium In Trudy Botanicheskogo Instituta Akademii Nauk SSSR,Ser. 1, Flora i Sistematika Vyssikh Rastenii. *Acta Instituti Botanici Academiae Scientiarum URPSS. 2*, 255-273. Moscow & Leningrad [St. Petersburg].

Ilardi, V., Brullo, S., Cusimano, D. & Giusso, G. (2014). Limonium poimenum (Plumbaginaceae), a new chasmophyte species from Sicily. *Phytotaxa, 188*(5), 268-274.

Ingrouille, M. J. (1985). The Limonium auriculae-ursifolium (POURRET) DRUCE group (Plumbaginaceae) in the Channel Isles. *Watsonia, 15*(3), 221-229.

Ingrouille, M. J., & Stace, C. A. (1986). The Limonium binervosum aggregate (Plumbaginaceae) in the British Isles. *Botanical journal of the Linnean Society, 92*(3), 177-217.

Jafri, S. M. H., & El-Gadi, A. (1984). Flora of Libya, vol. 109, 8-28. Al Faatheh University, Faculty of Science Publication, Tripoli.

Kouzali, I., Artelari, R., & Georgiou, O. (2012). Breeding systems and cytology in Cyprian populations of six Limonium species (Plumbaginaceae). *Willdenowia, 42*(2), 307-313.

Linczevski I. A. (1952). *Plumbaginaceae*. In: Shishkin BK, Bobrov EG, eds. Flora of the USSR. Moskva-Leningrad: Izdatel’stvo Akademii Nauk SSSR, 18: 292–474.

Linczevski I. A. (1971). Notes on Limoniaceae 3. *Botanicheskii Zhurnal, 56*, 633–1635.

Linczevski, I. A. (1985). Notulae de Limoniaceis, 6. *Novosti Sistematiki Vysshikh Rastenii, 22*, 200–207.

Linczevski, I., & Czukavina, A. (1984). New species of genus Limonium Mill.(Limoniaceae) from the Pamir-Alai [Limonium komarovii, Limonium ovczinnikovii, Limonium chodshamumynense]. *Novosti sistematiki vysshikh rastenii-Akademiia nauk SSSR, Botanicheskii institut 21*, 131-136.

Linczevski, J, I. (1986) Generis Limonium Mill. (Limoniaceae) species novae ex Australia. *Novosti Sistematiki Vysshikh Rastenii 23*, 101-110.

Linczevski, J. I. (1971). Notulae de Limoniaceis, 2. *Novosti Sistematiki Vysshikh Rastenii 8*, 209-214.

Lledó, M. D., Crespo, M. B., Fay, M. F., & Chase, M. W. (2005). Molecular phylogenetics of Limonium and related genera (Plumbaginaceae): biogeographical and systematic implications. *American Journal of Botany, 92*(7), 1189-1198.

Lobin, W., Leyens, T., Kilian, N., Erben, M., & Lewejohann, K. (1995). The genus Limonium (Plumbaginaceae) on the Cape Verde Islands, W Africa. *Willdenowia*, 197-214.

Lojacono, M. P. (1907). Flora sicula o descrizione delle piante vascolari spontanee o indigenate in *Sicilia, 2* (2), 14-30.

López-Alvarado, J., Cobacho, I., Arán, V. J., Rosato, M., Rosselló, J. A., & Sáez, L. (2017). Limonium ilergabonum (Plumbaginaceae), a new species from northeastern Iberian Peninsula. *Phytotaxa, 331*(2), 199-212.

Maire, R. (1936). Contributions à l’étude de la flore de l’Afrique du Nord. Fascicule 24. *Bulletin de la Société d’Histoire Naturelle de l’Afrique du Nord, 27*, 241-270.

Maire, R. (1938). Contributions à l’étude de la flore de l’Afrique du Nord. Fascicule 26. *Bulletin de la Société d’Histoire Naturelle de l’Afrique du Nord, 29*, 203-258.

Malekmohammadi, M., Akhani, H., & Borsch, T. (2017). Phylogenetic relationships of Limonium (Plumbaginaceae) inferred from multiple chloroplast and nuclear loci. *Taxon, 66*(5), 1128-1146.

Mayer, A. (1995). Comparative study of the coastal vegetation of Sardinia (Italy) and Crete (Greece) with respect to the effects of human influence. Ihw-Verlag.

Meyer, F. K. (2011). Beiträge zur flora von Albanien. Thüringische Botanische Gesellschaft e. V.

Moreno, J., Terrones, A., Alonso, M. Á., Juan, A., & Crespo, M. B. (2016). Limonium tobarrense (Plumbaginaceae), a new species from the southeastern Iberian Peninsula. *Phytotaxa, 257*(1), 61-70.

Moreno, J., Terrones, A., Alonso, M. Á., Juan, A., & Crespo, M. B. (2018). Taxonomic revision of the Limonium latebracteatum group (Plumbaginaceae), with the description of a new species. *Phytotaxa, 333*(1), 41-57.

Palacios, C., Rosselló, J. A., & González-Candelas, F. (2000). Study of the evolutionary relationships among Limonium species (Plumbaginaceae) using nuclear and cytoplasmic molecular markers. *Molecular phylogenetics and evolution, 14*(2), 232-249.

Pavon, D. (2005). Note sur le genre Limonium Miller dans le département des Bouches-du-Rhône. *Bulletin de la Société Linnéenne de Provence, 56*, 135-139.

Pérez, R. P., Silva-Pando, F. J., & Pérez, J. J. P. (2016). Notas sobre Limonium (Plumbaginaceae) en el Noroeste Ibérico, I: Limonium serpentinicum, Nueva Especie. *Novon: A Journal for Botanical Nomenclature, 24*(4), 380-388.

Pignatti, S. (1972) *Limonium*. In T. G. Tutin, V. H. Heywood, N. A. Burges, D. A. Valentine, S. M. Walters, D. A. Webb, and D. M. Moore [eds.], Flora Europaea, vol. 3, 38–50. Cambridge University Press, Cambridge, UK.

Pignatti, S. (1982a). *Limonium.* In Flora d’ Italia, vol. 2, 115-319. Edagricole. Bologna.

Pignatti, S. (1982b). New species of Limonium from Italy and Tunesia. *Webbia, 36*(1), 47-56.

Quezel, P., Santa, S., & Schotter, O. (1963). Nouvelle flore de l'Algerie et des regions desertiques meridionales-v. 2, 731-737.

Raimondo, F. M., & Domina, G. (2009). A new species of Limonium (Plumbaginaceae) from Soqotra (Yemen). *Plant Biosystems, 143*(3), 504-508.

Rizzotto, M. (1999). Research on the genus Limonium (Plumbaginaceae) in the Tuscan archipelago (Italy). *Webbia, 53*(2), 241-282.

Roselló, R., Stubing, G., Peris, J. B., & Cirujano, S. (1997). Limonium cordovillense* y* L. pinillense (Plumbaginaceae), dos nuevas especies de la flora Espanola. *Anales del Jardín Botánico de Madrid* *, 55*(2), 471-475.

Rosselló, J. A., Amézquita, M. M., & Marí, J. X. S. (1993). Limonium ejulabilis, a new endangered endemic species from Majorca (Balearic Islands, Spain). In *Anales del Jardín Botánico de Madrid* (Vol. 51, No. 2, pp. 199-204). Real Jardín Botánico.

Rosselló, J. A., Saez, L., & Carvalho, A. C. (1998). Limonium carvalhoi (Plumbaginaceae), a new endemic species from the Balearic Islands. In *Anales del Jardín Botánico de Madrid*(Vol. 56, No. 1, pp. 23-31). Real Jardín Botánico.

Rosselló, J.A. (2008) Relacions taxonòmiques i evolutives entre diverses espècies de saladines endèmiques de Balears. Jardí Botànic, Universitat de Valencia, Spain.

Sáez, L., & Rosello, J. A. (1999). Is Limonium cavanillesii Erben (Plumbaginaceae) really an extant species?. In *Anales del Jardín Botánico de Madrid* (Vol. 57, No. 1, pp. 47-55).

Sáez, L., & Rosselló, J. A. (1996). Limonium inexpectans (Plumbaginaceae), a new apomictic species from Mallorca (Balearic Islands). In *Anales del Jardín Botánico de Madrid* (Vol. 54, No. 1, pp. 285-289). Real Jardín Botánico.

Sáez, L., Carvalho, A. C. & Rosselló, J. A. (1998a). Limonium marisolii L. Llorens (Plumbaginaceae) revisited. In *Anales del Jardín Botánico de Madrid*(Vol. 56, No. 1, pp. 33-41). Real Jardín Botánico.

Sáez, L., Curco, A., L., & Rosselló, J. A. (1998b). Limonium vigoi (Plumbaginaceae), a new tetraploid species from the northeast of the Iberian Peninsula. In *Anales del Jardín Botánico de Madrid* (Vol. 56, No. 2, pp. 269-278). Real Jardín Botánico.

Sauvage, C. & Vindt, J. 1952. Flore de Maroc: Analytique, descriptive et illustrée, vol. 1., 42-77. Rabat: Editions Internationales.

Sauvage, Ch. & Vindt, J. (1954). Un nouvel endémique du Maroc central : Limonium,oudayense nov. sp . Comptes Rendus des Seances Mensuelles, *Societe des Sciences Naturelles et Physiques du Maroc 20*(3), 75.

Sauvage, Ch., & Vindt, J. (1953) Un nouvel endémique du Maroc oriental : Limonium pujosii nov*.. Mémoires de la Société des sciences naturelles du Maroc 19*, 115-116.

Soler, J. X. & Rosselló, J. A. (1997). On the status of Statice dianiae Pau (Plumbaginaceae). In *Anales del Jardín Botánico de Madrid* (Vol. 55, No. 1, pp. 9-16). Real Jardín Botánico.

Thulin, M. (2006). *Limonium.* In Flora of Somalia, Vol. 3,28-30. Kew Royal Botanic Gardens.

Tison, J. M., Jauzein, P., Michaud, H., & Michaud, H. (2014). Flore de la France méditerranéenne continentale (p. 1080-1085). Turriers: Naturalia publications.

Tzvelev, N.N. (2012). *Plumbaginaceae*. In: Takhtajan, A.L. (ed.), Konspekt flory Kavkaza, vol. 3(2), 272–284. St. Petersburg & Moscow: KMK Scientific Press.

Valli, A. T., & Artelari, R. (2015). Limonium korakonisicum (Plumbaginaceae), a new species from Zakynthos Island (Ionian Islands, Greece). *Phytotaxa, 217*(1), 63-72.

Véla, E., & Pavon, D. (2013). The vascular flora of Algerian and Tunisian small islands: if not biodiversity hotspots, at least biodiversity hotchpotchs. *Biodiversity Journal, 3*, 343-362.

Yamazaki, T. (1991). A new species of Limonium from Ryukyu. *Journal of Japanese Botany, 66*(3), 131-133.

Yıldırımlı, Ş. (2006). A new species Limonium adilguneri Yıld. & Doğru-Koca (Plumbaginaceae) from edge of Tuz Gölü, inner Anatolia Turkey. *Ot Sistematik Botanik Dergisi, 13*, 11-16.
